# Specific microRNA library of IFN-τ on bovine endometrial epithelial cells

**DOI:** 10.18632/oncotarget.18470

**Published:** 2017-06-14

**Authors:** Haichong Wu, Tao Zhang, Xiaofei Ma, Kangfeng Jiang, Gan Zhao, Changwei Qiu, Ganzhen Deng

**Affiliations:** ^1^ Department of Clinical Veterinary Medicine, College of Veterinary Medicine, Huazhong Agricultural University, Wuhan, People’s Republic of China

**Keywords:** microRNA, bovine endometrial epithelial cell, IFN-τ, pregnancy immunity

## Abstract

IFN-τ is specifically secreted by the conceptus in ruminants during early pregnancy, and it plays a vital role in the immunological function of pregnancy. However, its mechanism involving microRNA (miRNA) is still not well understood. Deep sequencing was used to explore the specific miRNA library of IFN-τ on bovine endometrial epithelial cells (bEECs). The results showed that 574 known bovine miRNAs and 109 novel miRNAs were identified. We found 74 differentially expressed miRNAs, including 30 commonly expressed miRNAs in the experiment. Then, qPCR verification of six selected miRNAs showed that they corresponded with the sequencing data. Gene Ontology (GO) and Kyoto Encyclopedia of Genes and Genomes (KEGG) pathway analysis revealed significant enrichment of predicted target genes of differentially expressed miRNAs, including influenza A, herpes simplex infection, antigen processing and presentation, viral myocarditis, TNF signaling pathway, graft-versus-host disease, and allograft rejection. These results may provide important contributions to the immune response during early pregnancy in ruminants, but further studies are need to verify the proposed cellular/immunological effects and role of specific miRNA as biomarkers *in vivo*.

## INTRODUCTION

IFN-τ, a novel member of type I interferon family, is specifically secreted by the conceptus in ruminants during early pregnancy, signaling maternal recognition of pregnancy and then embryonic implantation [1–4]. IFN-τ is secreted by the developing embryo in the restricted timeframe around implantation, and its production significantly increased from days 14 to 21 of cow pregnancy, which indicates transient control of the developing embryo and is responsible for developmental biology and reproductive immunology [5, 6]. It is well known that the type I interferon displays multiple immunemodulatory properties, which are also exhibited with IFN-τ [1, 7, 8]. In addition, it has been reported that IFN-τ plays a vital role in the early immunological interactions between the maternal-fetal interface [9]. Several studies have suggested that IFN-τ exerts the immunological function during early pregnancy in ruminants [6, 9]. Ozato et al. reported that the major histocompatibility complex class I (MHC-I) antigens play an essential role in immune responses during murine embryonic development [10]. Moreover, some studies have shown that the cellular expression of MHC-I molecules was modulated by type I interferon [11–13]. During pregnancy, MHC-I molecules are thought to regulate the maternal immune response in the placenta of eutherian mammals [14]. Although several reports have demonstrated that IFN-τ can contribute to the conceptus during pregnancy in ruminants [4, 15], the immunological mechanisms of IFN-τ that allow a semi-allogeneic fetus to develop in the maternal immune system remain unknown.

MicroRNA (miRNA), a small, non-coding RNA of approximately 21 nucleotides in length, is vital for controlling many processes in the immune system through targeting mRNAs for degradation or translational repression, affecting the output of many protein-coding genes [16–18]. Studies have demonstrated that miRNAs have emerged as primary bio-regulatory molecules during peri-implantation and pregnancy [19, 20]. For instance, miR-148a and miR-152 down-regulate human leukocyte antigen-G (HLA-G) expression, contributing to a healthy pregnancy [21].

To date, however, fewer studies have indicated that immune-related miRNAs from bovine endometrial epithelial cells (bEECs) are stimulated by IFN-τ. The advent of deep sequencing technology has made it possible to provide a framework to explore the physiological characteristics of miRNAs [22]. Therefore, in this study, bEECs were stimulated by IFN-τ, revealing a miRNA expression profile with Solexa high-throughput sequencing technology and allowing for exploration of the involved molecular mechanisms.

## RESULTS

### Cell identification and MTT assay

**Figure 1:**
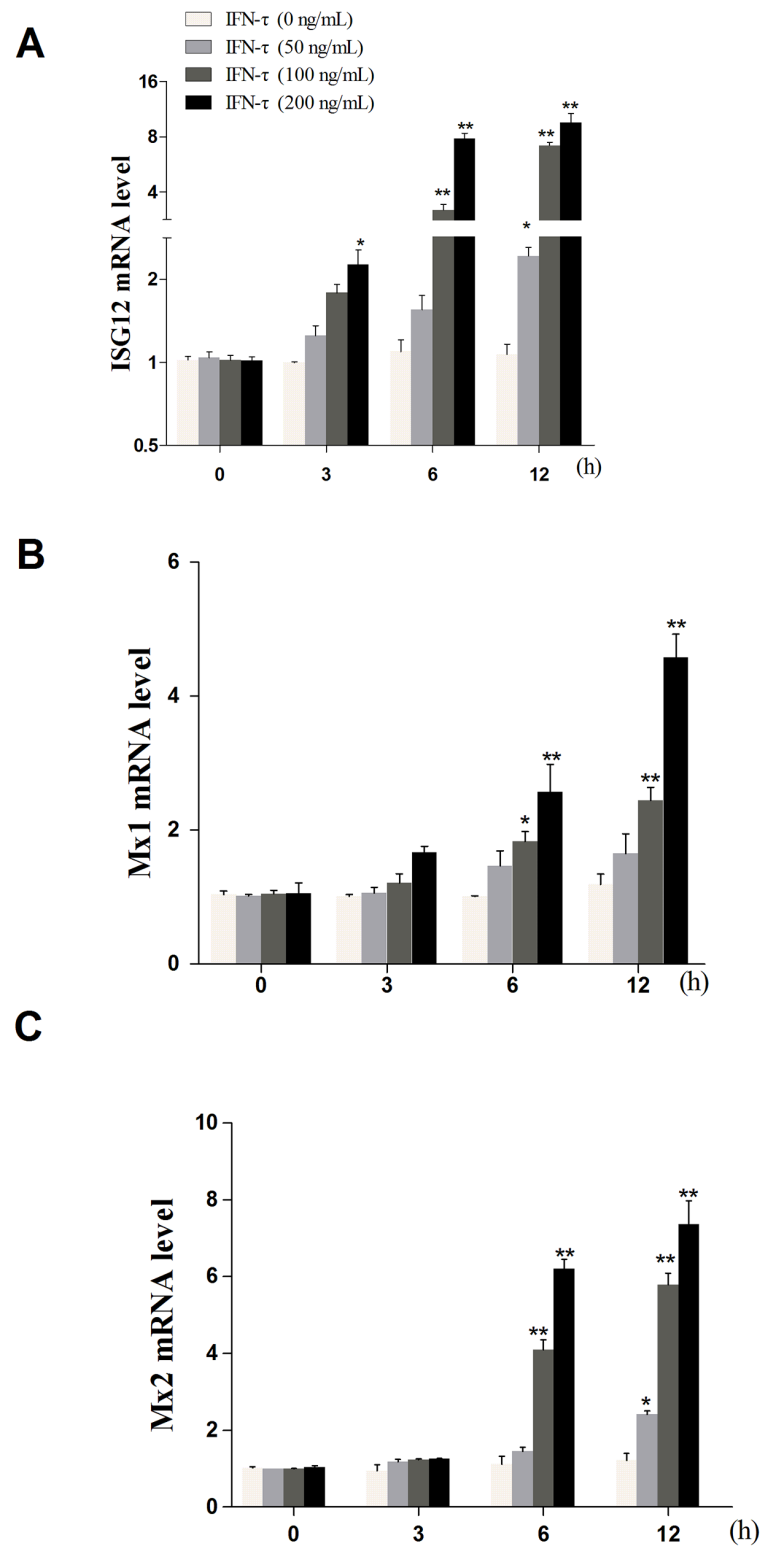
The effect of IFN-τon the expression of ISG12 (A), Mx1 (B), and Mx2 (C) mRNA Primary bEECs were treated with different concentrations of IFN-τ (0, 50, 100, and 200 ng/mL) and then harvested at 0, 3, 6, and 12 h, respectively. The expression of target genes was determined by qPCR. β-actin was used as a control. Data represent the mean ± S.E.M. of three independent experiments. *p<0.05 *vs.* IFN-τ (0 ng/mL). **p<0.01 *vs.* IFN-τ (0 ng/mL).

Cytokeratin 18 is an epithelial-specific marker to identify the bEEC integrity. bEECs were pretreated with DAPI to identify the cell nucleus and with cytokeratin 18 labeled with a red fluorochrome to observe the cell integrity. The results are shown in Figure 1A. The effect of IFN-τ on cell viability was evaluated by the MTT assay, and the results showed that the cell viability was unaffected by IFN-τ (200 ng/mL) treatment (Figure 1B).

### Sequence analysis

From the four small RNA (sRNA) libraries, the total reads reached more than one hundred million, and approximately 98.05% clean reads remained in Supplementary Table 1. The clean reads were clustered into unique sequences, and the general length of the miRNAs was 21-22 nt.

On average, 8.04 percent of the total reads corresponded to unique reads in each group. Additionally,87.06 percent of the total sRNA reads of 18-35 nt were mapped to the *bovine* genome. Furthermore, 88.66 percent of the total reads were identified as known miRNA sequences, while 0.02 percent of the total reads were unannotated, which required further analysis for novel miRNA candidates (Supplementary Table 2).

### Identification of miRNA and category analysis of specific miRNAs

A total of 574 unique mature miRNAs (467 from the CS group, 483 from the TS group, 463 from the CT group, and 457 from the TT group) were identified from the sRNA libraries. Among the known miRNAs, 338 miRNAs overlapped in each group (Supplementary Table 3). A total of 109 novel miRNAs (79 from the CS group, 66 from the TS group, 59 from the CT group, and 69 from the TT group) were predicted through miREvo and miRDeep2 software (Supplementary Table 4). Fourteen were co-expressed in each group from novel mature miRNAs. The read counts of these novel miRNAs ranged from 1 to 1011.

The number of differentially expressed miRNAs between each group were analyzed using DESeq2 (Supplementary Table 5). In detail, there were 66 (42 up-regulated and 24 down-regulated) and 38 (32 up-regulated and 6 down-regulated) miRNAs with significant expression variance identified in the TS *vs.* CS and TT *vs.* CT groups, respectively.

### Differential expression analysis and qRT-PCR verification

**Figure 2:**
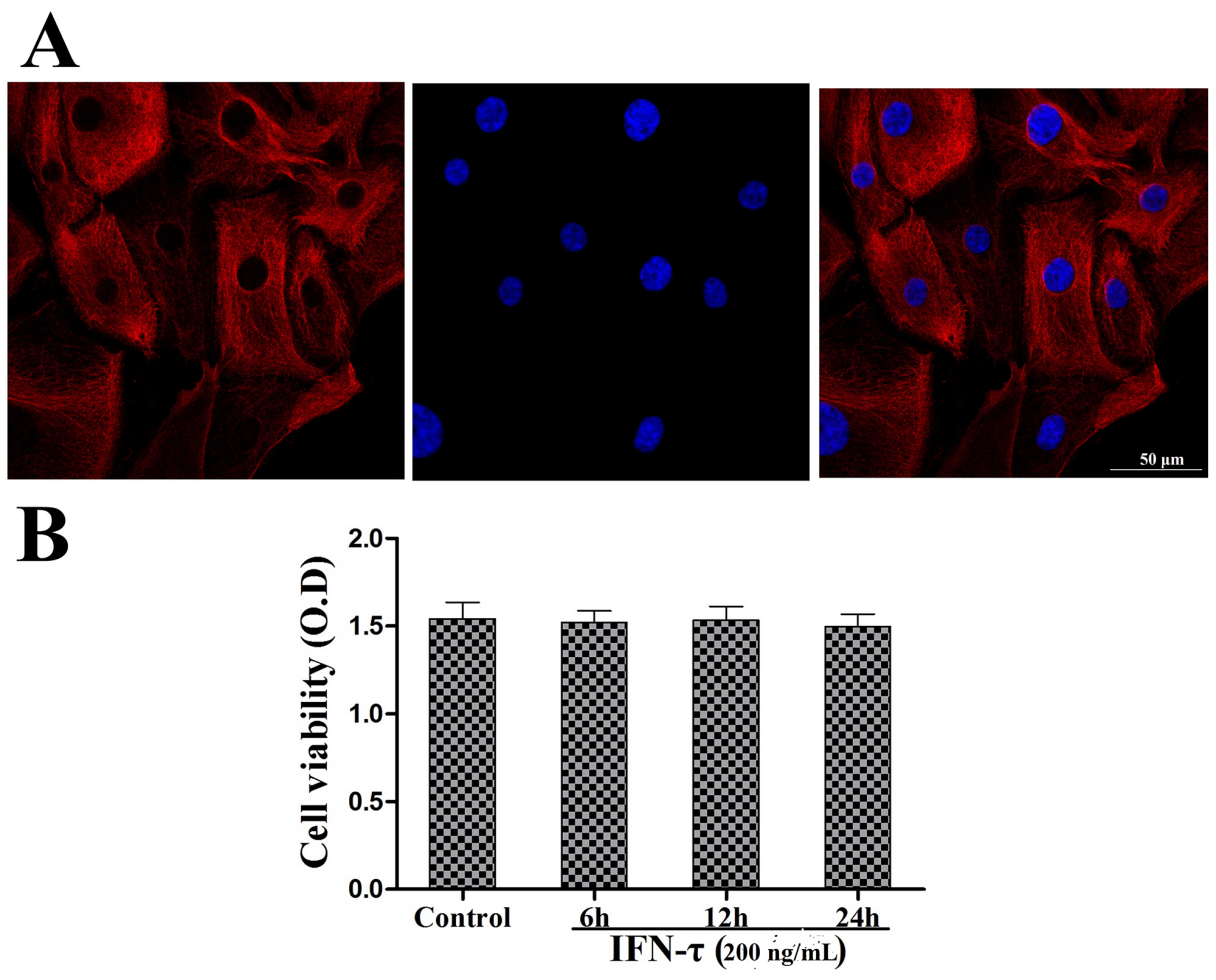
Bovine endometrial epithelial cell identification and viability **(A)** Bovine endometrial epithelial cells were pretreated with fluorochrome to observe the endometrial epithelial cell integrity. The cell nucleus was marked with blue fluorescence. Cytokeratin 18 was labeled with a red fluorochrome label (magnification ×400). **(B)** The effect of IFN-τ on the cell viability of bovine endometrial epithelial cells. Cells were cultured with IFN-τ (200 ng/mL) for 6, 12, and 24 h and then measured by the MTT assay. The values represent the means ± S.E.M of three replicates.

Selecting out some key miRNAs from the library, we performed expression pattern analysis for these miRNA groups. The 30 commonly expressed miRNAs (29 conserved miRNAs and 1 novel miRNA) are shown in Figure 2A, (Supplementary Table 6). We then processed the clustering for each group by hierarchical cluster, and the results of the hierarchical cluster are shown by heatmap (Figure 2B). Among them, 3 miRNAs were down-regulated and 27 miRNAs were up-regulated. These commonly expressed miRNAs were sequenced at varying frequencies. Some miRNAs, such as bta-miR-10a, bta-miR-184, and bta-miR-200a, were detected with relatively high read counts in both groups, while other miRNAs were detected with low read counts, containing novel_3 andbta-miR-135a.

**Figure 3:**
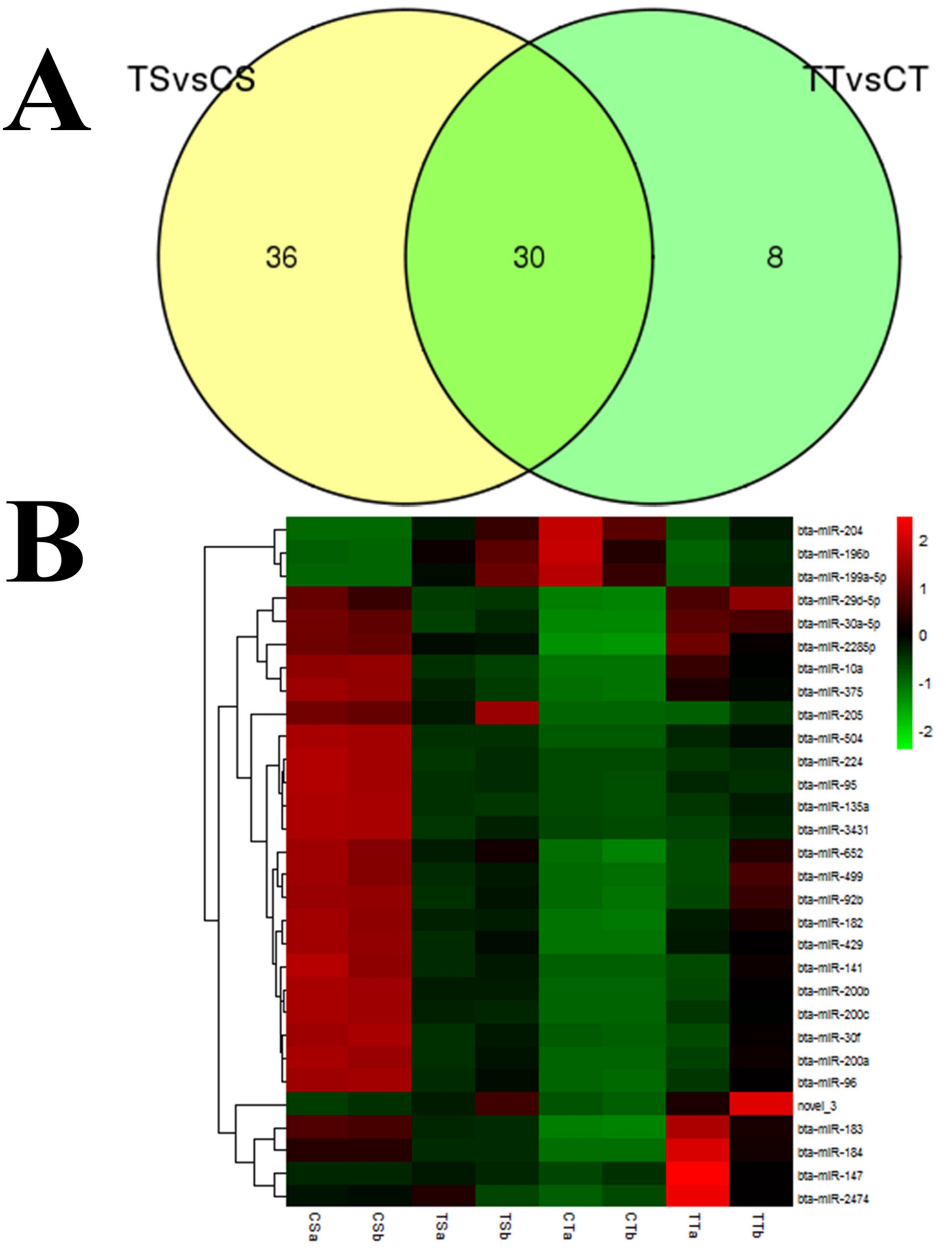
Differential expression of miRNAs analyzed in bovine endometrial epithelial cells treated with IFN-τ **(A)** Venn diagram indicating exclusively and commonly expressed miRNAs in the TS *vs.* CS group and TT *vs.* CT group. **(B)** The heatmap for the commonly expressed miRNAs with significant expression variance. The color scale indicated the relative expression level of miRNAs; red denotes expression > 0 and green denotes expression < 0.

To validate the reliability of the sequencing data with the stem-loop PCR assay, we selected six abundantly differentially expressed miRNAs. The result is shown in Figure 3, which was consistent with the deep sequencing results.

### GO enrichment and KEGG pathway analysis in differentially expressed miRNAs

**Figure 4:**
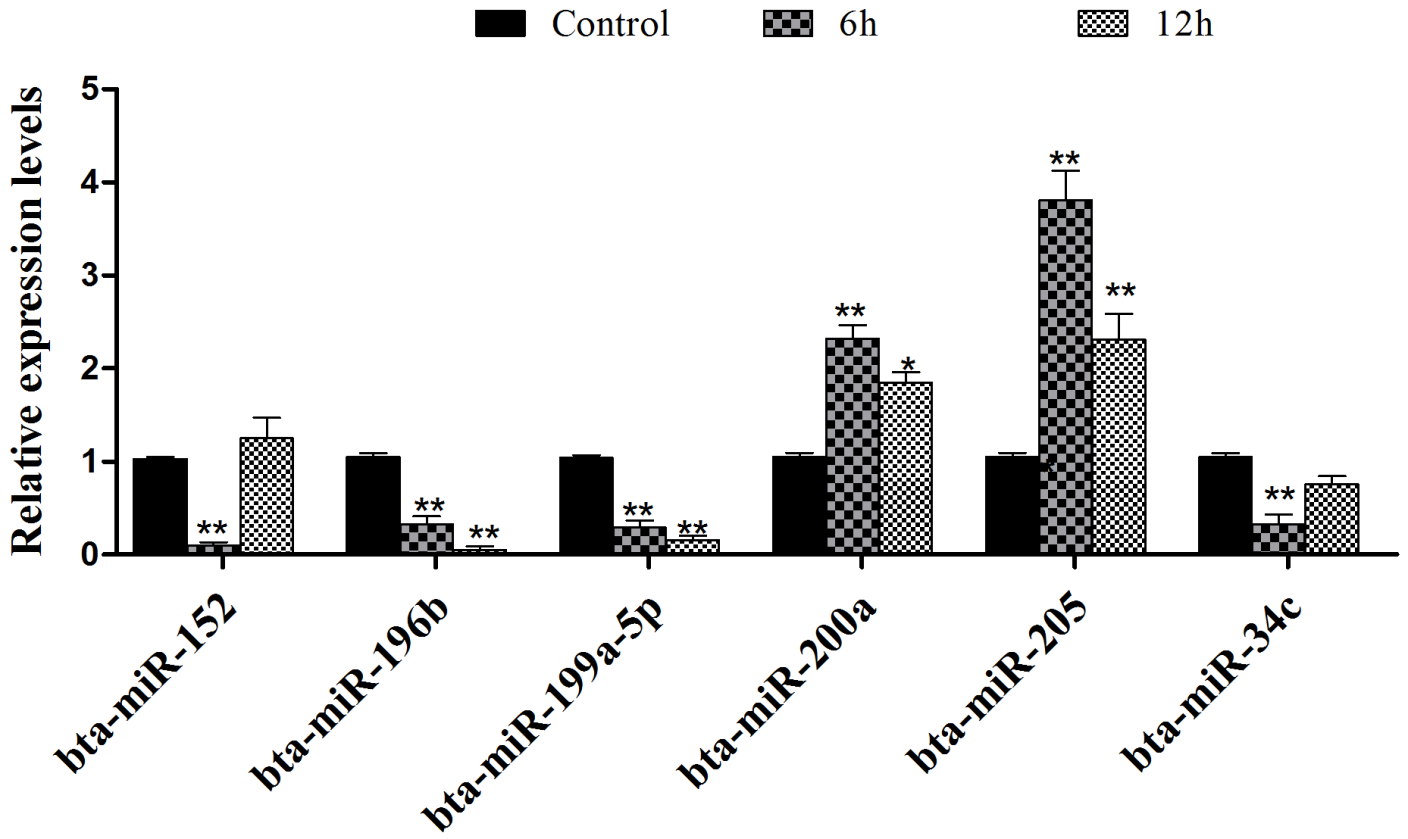
Validation of differentially expressed miRNAs by qPCR Selection of six differentially expressed miRNAs to qualify the reliability of the sequencing data using qPCR assay. The result was consistent with the sequencing data. Data represent the mean ± S.E.M. of three independent experiments. *p<0.05 *vs.* Control group. **p<0.01 *vs.* Control group.

Target gene prediction of differentially expressed miRNAs indicated that approximately 8891 (TS *vs.* CS group) and 6693 (TT *vs.* CT group) mRNA transcripts may be regulated by these miRNAs (Supplementary Table 7). Gene ontology (GO) functional annotation showed that the target genes of differentially expressed miRNAs were significantly enriched in different groups (*p*<0.05), including 14 molecular function terms, 8 cellular component terms, and 33 biological process terms in the TS *vs.* CS group, while there was only 1 molecular function term and there were 9 biological process terms in the TT *vs.* CT group (Supplementary Table 8). The metabolic process, immune system process, and cytokine activity were the most enriched terms in the biological processes, cellular components, and molecular functions, respectively (Figure 4).

Several Kyoto Encyclopedia of Genes and Genomes (KEGG) pathways were significantly enriched by target genes of differentially expressed miRNAs (*p*<0.05), which mainly contained the following seven pathways: influenza A, herpes simplex infection, antigen processing and presentation, viral myocarditis, TNF signaling pathway, graft-versus-host disease, and allograft rejection (Table 1). These pathways may be involved in the immune-related regulation of IFN-τ.

**Table 1:** Primers used for qPCR

Name	Sequence (5’→3’): forward and reverse	GenBank accession no.	Product size (bp)
**ISG12**	CTTCACCAGTGCAGGAATCA	NM_001038050	195
	CCCAAAAATTTGGACACGAG		
**Mx1**	GTCCCTGCTAACGTGGACAT	NM_173940	155
	ACCAGGTTTCTCACCACGTC		
**Mx2**	GCAGATCAAGGCACTCATCA	NM_173941.2	168
	ACCAGGTCTGGTTTGGTCAG		
**β-actin**	CTCTTCCAGCCTTCCTTCCT	BC102948	124
	GGGCAGTGATCTCTTTCTGC		

## DISCUSSION

IFN-τ, a key cytokine in ruminants, is produced at between 12-21 days and inhibits luteolysis through decreasing endometrial oxytocin receptors to maintain pregnancy [6, 23]. However, the immunological mechanism by which IFN-τ contributes to pregnancy remains unknown. Next-generation sequencing technology has transformed many areas of biological and translational research, and it has obtained accurate profiling of the expression of miRNAs at a high-throughput level [24]. Using this technology, we obtained the miRNA library in bovine endometrial epithelial cells treated with IFN-τ. It is well-known that not all miRNAs are equally important; different miRNAs emerge as major regulators that control cell functions in various physiological and pathophysiological process [22]. Therefore, analysis of the miRNA change in the bEECs treated with IFN-τ will be important to identify the miRNAs involved in the immunological regulation of IFN-τ during early pregnancy in ruminants.

The present study aimed to systematically describe the variation of miRNAs obtained from deep sequencing. Of note, only two biological replicates were used for each group in this study design because of the limited budget. It has been demonstrated that with most methods, over 90% of differentially expressed genes at the top expression levels could be detected using two replicates [25]. We selected the differentially expressed miRNAs and used the qPCR method to identify the sequencing result. The qPCR result suggested that the accuracy of deep sequencing was reliable, which has been supported by many previous studies [26]. We found the expression levels of bta-miR-184, novel-3, bta-miR-200a, bta-miR-200b, and bta-miR-200c were increased, but the expression of bta-miR-152 was significantly down-regulated in IFN-τ treatment. It could be that IFN-τ was produced by the developing embryo in the restricted timeframe around implantation, which indicated its transient control of the developing embryo [6].

To further explore the potential functions of the differentially expressed miRNAs regulating IFN-τ treatment bEECs, the predicted targets of these miRNAs were analyzed by GO and KEGG pathway annotation. The GO annotation provides ontology of defined terms representing gene product properties that were divided into the following three main domains: Biological process, Molecular function, and Cellular component. Additionally, KEGG pathway annotation contains systematic analysis of inner-cell metabolic pathways and functions of gene products, which facilitate study of the complex biological processes of genes [27].

The GO annotation of special miRNAs was related to chemokine receptor binding, cellular metabolic processes, immune system processes, and establishment of protein localization, demonstrating that the functions of differentially expressed miRNAs have a particularly close relationship with IFN-τ and its receptors. In the present study, bta-miR-200a, bta-miR-200b, and bta-miR-200c were predicted to mediate the cellular metabolic process. Liu et al. reported that miR-200acan suppress the differentiation of mouse embryonic stem cells into endoderm and mesoderm by directly regulating Grb2 expression and Erk signaling [28]. During embryo implantation, estrogen and progesterone directly and indirectly promote distinct cycles of cell proliferation and differentiation in the uterus tissues[29], and estrogen and progesterone secretion was regulated by IFN-τ in the bovine endometrium [30]. Therefore, bta-miR-200 may regulate embryo implantation through affecting IFN-τ, affecting the cytokine levels. Moreover, many reports have demonstrated that miR-152 played a vital role in immune regulation [31, 32], which was also observed in our study (Supplementary Table 8).

KEGG analysis showed that distinct biological processes and significant pathways may be involved in the early pregnancy of ruminants. These pathways, including influenza A, herpes simplex infection, antigen processing and presentation, viral myocarditis, tumor necrosis factor (TNF) signaling, graft-versus-host disease, and allograft rejection pathways, were significantly enriched in the present study. Although few reports have shown that the influenza A, herpes simplex infection, and herpes simplex infection pathways are directly regulated by IFN-τ in bEECs, many studies have demonstrated the critical role of IFN-τ in antiviral immunity [33, 34]. In our results, the antigen processing and presentation and allograft rejection pathways were targeted by miRNAs including bta-miR-148a, bta-miR-148b, bta-miR-152, bta-miR-375, bta-miR-3431, novel_3, bta-miR-224, bta-miR-199a-5p, bta-miR-504, bta-miR-200b, bta-miR-200c, and bta-miR-429. A study demonstrated that HLA-G, an immunomodulatory molecule, is mainly expressed by extravillous cytotrophoblasts and controlled by miR-148a and miR-152 in humans [21]. The homology of humans and cattle has been demonstrated in a previous study, indicating the possible involvement of the immunological pathways is affected by IFN-τ in bEECs.

The TNF pathway is very important for inducing a wide range of intracellular signal pathways, including inflammatory immune pathways [35]. Additionally, it has been reported that miR-224participates in the inflammatory immune process [36]. In our previous study, we have also demonstrated that IFN-τ plays a key role in the inflammatory response [37]. Therefore, enrichment of the TNF signaling pathway in the present study may suggest its critical role in the pregnancy immune response.

In summary, we showed that IFN-τ stimulation activated a wide variety of miRNAs in a time specific manner. Using deep sequencing, we characterized the miRNome of bovine endometrial epithelial cells challenged with or without IFN-τ, and we detected 574 known bovine miRNAs and 109 novel miRNAs. We found 74 differentially expressed miRNAs with 30 commonly expressed miRNAs. GO and KEGG pathway analysis revealed significant enrichment of predicted target genes of differentially expressed miRNAs, including those involved in influenza A, herpes simplex infection, antigen processing and presentation, viral myocarditis, TNF signaling, graft-versus-host disease, and allograft rejection. These results may provide important contributions to the immune response during early pregnancy in ruminants, but further studies are needed to verify the proposed cellular/immunological effects and role of specific miRNA as biomarkers *in vivo*.

## MATERIALS AND METHODS

### Reagents

Recombinant bovine interferon-tau (IFN-τ, HPLC >97%) was purchased from Creative Biomart (NY, USA).

### Cell culture and identification

The bovine primary endometrial epithelial cells (bEECs) were isolated and cultured as previously described [38]. Briefly, uteruses of Holstein cows were obtained from a local slaughterhouse and immediately returned to the laboratory in pre-cooled phosphate buffer solution (PBS). The uterine lumen was washed 3 times with 30-50 mL of sterile Ca^2+^- and Mg^2+^- free Hanks’ balanced salt solution supplemented with 100 IU/mL penicillin and streptomycin and containing 0.1% BSA. Then, 0.05% collagenase I (Sigma, USA) was then infused into the uterine lumen through the catheter. Epithelial cells were isolated by incubation twice at 37°C for 45 min and 30 min with gentle shaking. Cells were cultured in DMEM/F12 containing 10% fetal bovine serum and incubated at 37°C in air with 5% CO_2_.

Cells spread to the third generation were used for the experiments. The cells were passaged in twelve-well plates with a cover slip and analyzed for the expression of epithelial-specific marker cytokeratin 18 (Abcam, UK). Cells were grown to 60-70% confluence and fixed with paraformaldehyde at room temperature for 10 min; then, the cells were washed three times with PBS. The cells were blocked with 10% normal goat serum (Invitrogen, USA) at room temperature for 30 min and then incubated with primary antibody cytokeratin 18 (diluted 1:300 in PBS) overnight at 4°C. The secondary fluorescently labeled antibodies Dylight 594 antibodies (Bioss, China) were incubated for 45 min at room temperature and washed three times in PBS. DAPI was used to stain the cell nuclei (Roche, Germany). Fluorescent images were observed using laser scanning confocal microscopy (Leica, Germany).

### Cell treatment protocol

**Figure 5:**
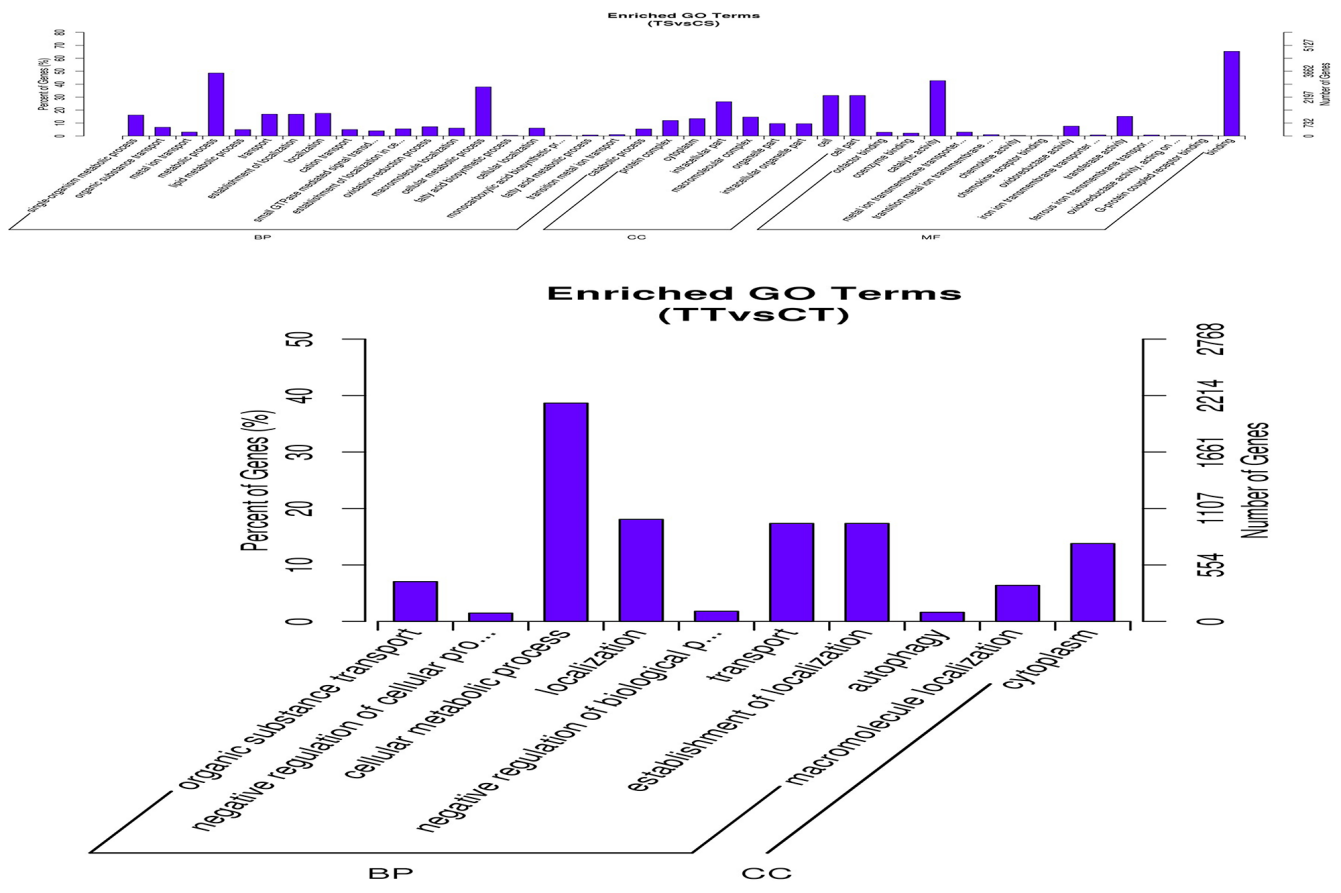
GO term of differentially expressed genes in bovine endometrial epithelial cells treated with IFN-τ The Top 20 GO (biological process) term analyses of differentially expressed genes of the TS *vs.* CS group and TT *vs.* CT group. MF indicates molecular function; BP indicates biological process; and CC indicates cellular component.

bEECs were seeded at a density of 1 × 10^6^ cells/mL in six-well plates and cultured for 12 h. The concentration of IFN-τ was chosen according to the effect of IFN-τ on the expression of several key genes during early pregnancy, including interferon-stimulated gene (ISG) 12, *Mx1*, and *Mx2*, which were influenced by IFN-τ in the uterus of ruminants [39]. The results indicated that IFN-τ at a concentration of 200 ng/mL better regulated bEECs (shown in Figure 5). The primers were displayed in Table 2. This experiment was divided into the following four groups: cells were treated with IFN-τ (200 ng/mL) for 6 h (TS group) or 12 h (TT group) and untreated cells were used as control groups at the corresponding time points of 6h and 12 h (CS and CT groups, respectively). There were two biological replicates in each group. To exclude the effect of IFN-τ on cell viability, the 3-[4,5-dimethylthiazol-2-yl]-2,5 diphenyl tetrazolium bromide (MTT) assay was performed according to the manufacturer’s protocol using the MTT kit. The cells (1×10^4^ cell/well) were treated with IFN-τ (200 ng/mL) for 6, 12, and 24 h. The absorbance was read at 570 nm with a microplate reader (Thermo, USA).

**Table 2:** Sequence of primers used for qPCR

MicroRNA	Primer names	Sequences
bta-miR-152	Stem-loop	CTCAACTGGTGTCGTGGAGTCGGCAATTCAGTTGAGcccaagtt
	Forward	TCGGCAtcagtgcatgacag
	Reverse	CTCAACTGGTGTCGTGGA
bta-miR-196b	Stem-loop	CTCAACTGGTGTCGTGGAGTCGGCAATTCAGTTGAGtcccaa
	Forward	GCCGAGtaggtagtttcctg
	Reverse	TGGTGTCGTGGAGTCGGCAAT
bta-miR-199a-5p	Stem-loop	CTCAACTGGTGTCGTGGAGTCGGCAATTCAGTTGAGaacagg
	Forward	TGCGGAcccagtgttcagacta
	Reverse	CTCAACTGGTGTCGTGGAG
bta-miR-200a	Stem-loop	CTCAACTGGTGTCGTGGAGTCGGCAATTCAGTTGAGcatcgt
	Forward	GCCGAGtaacactgtctggt
	Reverse	CTCAACTGGTGTCGTGGAGT
bta-miR-205	Stem-loop	CTCAACTGGTGTCGTGGAGTCGGCAATTCAGTTGAGcagact
	Forward	TCGGAtccttcattccaccgg
	Reverse	CTCAACTGGTGTCGTGGAGT
bta-miR-34c	Stem-loop	CTCAACTGGTGTCGTGGAGTCGGCAATTCAGTTGAGcaatcagc
	Forward	TCGGAaggcagtgtagttagc
	Reverse	CTCAACTGGTGTCGTGGAGT
U6	Stem-loop	CGCTTCACGAATTTGCGTGTCAT
	Forward	GCTTCGGCAGCACATATACTAAAAT
	Reverse	CGCTTCACGAATTTGCGTGTCAT

### RNA isolation and qualification

To explore the molecular mechanisms, bEECs exposed to IFN-τ at different times were collected fortranscriptomic analysis. Total RNA was extracted from bEECs using Trizol reagent (Invitrogen, USA), and its integrity, purity and concentration were determined using an RNA Nano 6000 Assay Kit of the Agilent Bioanalyzer 2100 system (Agilent Technologies, CA, USA), NanoPhotometer® spectrophotometer (IMPLEN, CA, USA), and Qubit® RNA Assay Kit in Qubit® 2.0 Flurometer (Life Technologies, CA, USA), respectively.

### Library construction and sequencing data analysis

Then, sequencing was performed by Novogene Bioinformatics Technology Co., Ltd. (Beijing, China). Four small RNA libraries were established using NEBNext® Multiplex Small RNA Library Prep Set for Illumina® (NEB, USA) according to the manufacturer’s instructions, and index codes were added to attribute sequences to each sample. RNA sequencing was performed on an Illumina Hiseq 2500/2000 platform and 50-bp, single-end reads were generated.

Clean reads (clean data) were obtained from raw data by removing glow quality and contaminated reads. Then, clean reads of 18-35 nt sRNA were mapped to a reference sequence by Bowtie [40]. Mapped sRNA tags were used to look for known miRNA, and miRBase20.0 was used for reference. miREvo and mirdeep2 software were integrated to predict novel miRNA targets [41, 42].

### Differential expression and quantification of miRNA

The miRNA expression levels were estimated by TPM (transcript per million) through the following criteria [43]: Normalized expression = mapped readcount/Total reads*1,000,000. Differential expression analysis of two groups was conducted using the DESeq R package (1.8.3). The *p*-value was adjusted using the Benjamini and Hochberg methods, and *p* < 0.05 was considered statistically significant.

Differential expression of miRNAs was further confirmed using the stem-loop qRT-PCR method. In the present study, we selected six miRNAs to identify the RNA sequencing results. A separate treatment experiment was performed using the same treatment protocol as described above. Then, the total RNA of bEECs was extracted by Trizol reagent according to the manufacturer’s recommendation (Invitrogen, USA). One microgram of total RNA from each sample was reverse-transcribed into cDNA using the Reverse Transcriptase M-MLV (TaKaRa) and Hairpin-itTM microRNA qPCR Quantitation Kit (GenePharma, Shanghai, China). The miRNA and U6 primers are listed in Table 3. The qPCR was performed using the SYBR^®^ Select Master Mix kit and standard protocols on the Step One Real-Time PCR System (Applied Biosystems, USA). U6 was used as an internal control. The PCR conditions were as follows: 95 °C for 10 min and then 40 cycles of 95 °C for 15 s, 60 °C for 60 s, and 72 °C for 60 s. The experiment was conducted in three biological and two technical replicates. The 2^-ΔΔCt^ comparative method was used to analyze the expression levels.

**Table 3:** Comparison of hemodynamic variables and echocardiographic parameters between control group and liraglutide group

Enriched pathways by target genes of differentially expressed miRNAs			
(TS *vs.* CS group)			
ID	KEGG_Term	Genes	*p*-Value
bta05164	Influenza A	70	0.001738256
bta05168	Herpes simplex infection	100	0.001738256
bta04612	Antigen processing and presentation	36	0.002566128
bta05416	Viral myocarditis	35	0.014447409
bta04668	TNF signaling pathway	72	0.016962605
bta05332	Graft-versus-host disease	18	0.022359168
bta05330	Allograft rejection	17	0.047396265

Enriched pathways by target genes of differentially expressed miRNAs			
(TT *vs.* CT group)			
ID	KEGG_Term	Genes	*p*-Value
bta05164	Influenza A	70	0.006594627
bta05168	Herpes simplex infection	74	0.009257507
bta04612	Antigen processing and presentation	26	0.015602785
bta05416	Viral myocarditis	28	0.015871283
bta04668	TNF signaling pathway	63	0.027027971
bta05332	Graft-versus-host disease	14	0.040435867
bta05330	Allograft rejection	14	0.046060936

*p*-value was adjusted using the Benjamini method.

### Target gene prediction, GO and KEGG enrichment analysis

Prediction of the target gene of differentially expressed miRNAs was performed by miRanda [44]. GOseq based Wallenius non-central hyper-geometric distribution was performed for Gene Ontology (GO) enrichment analysis [45]. KOBAS (v2.0) software was implemented for Kyoto Encyclopedia of Genes and Genomes (KEGG) pathway analysis and the corrected *p* value (FDR) cut-off was set at 0.05 [46].

### Statistical analyses

Data were analyzed by SPSS 15.0 software and presented as the mean ± S.E.M. The comparisons between the groups were performed by ANOVA followed by Dunnett’s test. *p*< 0.05 was considered statistically significant.

## SUPPLEMENTARY MATERIALS TABLES














