# Supplementary material for: Specific microRNA library of IFN-τ on bovine endometrial epithelial cells
Source: Oncotarget. 2017 Jun 14;8(37):61487–98. doi: 10.18632/oncotarget.18470 (PMC5617439; doi:10.18632/oncotarget.18470)
Supplement: Supplementary file 1 [file oncotarget-08-61487-s001.pdf]

Specific microRNA library of IFN-τ on bovine endometrial epithelial cells

SUPPLEMENTARY MATERIALS

Supplementary Table 1: Quality and data filtering of small RNA sequencing data.

See Supplementary File 1

Supplementary Table 2: Classification and annotation of small RNAs

Types	CSa (percent)	CSb (percent)	TSa (percent)	TSb (percent)	CTa (percent)	CTb (percent)	TTa (percent)	TTb (percent)
Total	8763846 100.00%	9312576 100.00%	6342501 100.00%	6599719 100.00%	6030710 100.00%	6747805 100.00%	8340739 100.00%	4778573 100.00%
Known_ miRNA	7679899 87.63%	8107189 87.06%	5723962 90.25%	5827939 88.31%	5435906 90.14%	6306567 93.46%	7294219 87.45%	4058853 84.94%
rRNA	219934 2.51%	239425 2.57%	97001 1.53%	131212 1.99%	80551 1.34%	55776 0.83%	58685 0.70%	100784 2.11%
tRNA	1936 0.02%	2082 0.02%	5244 0.08%	6019 0.09%	6247 0.10%	3452 0.05%	3358 0.04%	3768 0.08%
snRNA	5947 0.07%	6383 0.07%	1637 0.03%	2622 0.04%	1819 0.03%	1020 0.02%	1504 0.02%	1627 0.03%
snoRNA	161354 1.84%	189856 2.04%	69961 1.10%	74854 1.13%	56995 0.95%	54990 0.81%	185261 2.22%	73997 1.55%
Repeat	92006 1.05%	115494 1.24%	80057 1.26%	95860 1.45%	90410 1.50%	63310 0.94%	114032 1.37%	95201 1.99%
Novel_ miRNA	1686 0.02%	1543 0.02%	699 0.01%	1673 0.03%	1932 0.03%	754 0.01%	690 0.01%	809 0.02%
exon:+	56462 0.64%	70657 0.76%	65028 1.03%	78296 1.19%	69216 1.15%	53602 0.79%	103502 1.24%	68635 1.44%
exon:-	1086 0.01%	1031 0.01%	1712 0.03%	1592 0.02%	2303 0.04%	1600 0.02%	2104 0.03%	1243 0.03%
intron:+	140601 1.60%	152230 1.63%	75922 1.20%	76095 1.15%	52528 0.87%	55607 0.82%	153869 1.84%	72883 1.53%
intron:-	53609 0.61%	52864 0.57%	54882 0.87%	67744 1.03%	48362 0.80%	29005 0.43%	80254 0.96%	99991 2.09%
other	349326 3.99%	373822 4.01%	166396 2.62%	235813 3.57%	184441 3.06%	122122 1.81%	343261 4.12%	200782 4.20%

Supplementary Table 3: Read counts of known mature miRNA in each sample.

See Supplementary File 2

Supplementary Table 4: Novel mature miRNA sequence and read counts.

See Supplementary File 3

Supplementary Table 5: Differentially expressed miRNA in each group.

See Supplementary File 4

Supplementary Table 6: Commonly expressed miRNAs

Name
bta-miR-10a
bta-miR-135a
bta-miR-141
bta-miR-147
bta-miR-182
bta-miR-183
bta-miR-184
bta-miR-196b
bta-miR-199a-5p
bta-miR-200a
bta-miR-200b
bta-miR-200c
bta-miR-204
bta-miR-205
bta-miR-224
bta-miR-2285p
bta-miR-2474
bta-miR-29d-5p
bta-miR-30a-5p
bta-miR-30f
bta-miR-3431
bta-miR-375
bta-miR-429
bta-miR-499
bta-miR-504
bta-miR-652
bta-miR-92b
bta-miR-95
bta-miR-96

Supplementary Table 7: Target gene prediction of differentially expressed miRNAs.

See Supplementary File 5

Supplementary Table 8: Gene ontology enrichment of predicted targets.

See Supplementary File 6
