# Supplementary material for: Specific microRNA library of IFN-τ on bovine endometrial epithelial cells
Source: Oncotarget. 2017 Jun 14;8(37):61487–98. doi: 10.18632/oncotarget.18470 (PMC5617439; doi:10.18632/oncotarget.18470)
Supplement: Supplementary file 2 [file oncotarget-08-61487-s002.doc]

**Supplementary Table 1: Quality and data filtering of small RNA sequencing data**

| **Sample** | **CSa** |  | **CSb** | **TSa** | **TSb** | **CTa** | **CTb** | **TTa** | **TTb** |
| --- | --- | --- | --- | --- | --- | --- | --- | --- | --- |
| Total reads 14692097 14305759 10274174 12809542 12846077 11403178 14013955 10549807  Bases 0.735G 0.715G 0.514G 0.640G 0.642G 0.570G 0.701G 0.527G  Error rate 0.01% 0.01% 0.01% 0.01% 0.01% 0.01% 0.01% 0.01%  Q20 97.40% 97.18% 97.23% 97.24% 96.78% 96.76% 96.78% 96.73%  Q30 95.10% 94.80% 94.54% 94.51% 93.49% 93.60% 93.63% 93.17%  GC content 48.22% 48.26% 47.62% 47.89% 47.58% 47.34% 48.53% 48.18% | | | | | | | | | |
| N%>10% 404 383 818 1068 160 88 156 44  (0.00%) (0.00%) (0.01%) (0.01%) (0.00%) (0.00%) (0.00%) (0.00%)  Low quality 8722 8494 13646 12409 21411 25490 21529 19096  (0.06%) (0.06%) (0.13%) (0.10%) (0.17%) (0.22%) (0.15%) (0.18%)  5 adapter 359 290 260 414 572 302 341 268  contamine (0.00%) (0.00%) (0.00%) (0.00%) (0.00%) (0.00%) (0.00%) (0.00%)  3 adapter 193701 177510 108501 163491 241944 179920 337941 207937  contamine (1.32%) (1.24%) (1.06%) (1.28%) (1.88%) (1.58%) (2.41%) (1.97%)  With poly 6122 5818 4514 6625 7536 5105 6828 5953  A/T/G/C (0.04%) (0.04%) (0.04%) (0.05%) (0.06%) (0.04%) (0.05%) (0.06%)  Clean 14482789 14113264 10146435 12625535 12574454 11192273 13647160 10316509  reads (98.58%) (98.65%) (97.38%) (98.56%) (97.89%) (98.15%) (97.38%) (97.79%) | | | | | | | | | |
