# Supplementary material for: Specific microRNA library of IFN-τ on bovine endometrial epithelial cells
Source: Oncotarget. 2017 Jun 14;8(37):61487–98. doi: 10.18632/oncotarget.18470 (PMC5617439; doi:10.18632/oncotarget.18470)
Supplement: Supplementary file 3 [file oncotarget-08-61487-s003.doc]

**Supplementary Table 3: Read counts of known mature miRNA in each sample**

| **miRNA** | **CSa** | **CSb** | **TSa** | **TSb** | **CTa** | **CTb** | **TTa** | **TTb** |
| --- | --- | --- | --- | --- | --- | --- | --- | --- |
| bta-let-7a-3p | 570 | 615 | 264 | 435 | 527 | 275 | 281 | 260 |
| bta-let-7a-5p | 172985 | 174501 | 49758 | 96114 | 92143 | 45339 | 30891 | 43256 |
| bta-let-7b | 119109 | 117537 | 22098 | 49515 | 44538 | 17290 | 6853 | 21572 |
| bta-let-7c | 3879 | 3451 | 841 | 1674 | 1387 | 687 | 485 | 820 |
| bta-let-7d | 9064 | 9537 | 1978 | 3943 | 2668 | 1129 | 831 | 1990 |
| bta-let-7e | 11732 | 10699 | 6365 | 8800 | 8143 | 6335 | 2727 | 6781 |
| bta-let-7f | 188664 | 188943 | 75061 | 96355 | 81140 | 57254 | 109692 | 71371 |
| bta-let-7g | 131959 | 130259 | 45990 | 72231 | 69438 | 39704 | 20894 | 41734 |
| bta-let-7i | 185665 | 191961 | 167821 | 253309 | 389400 | 240495 | 104997 | 134147 |
| bta-miR-1 | 15 | 22 | 625 | 1037 | 2625 | 1799 | 383 | 832 |
| bta-miR-100 | 15360 | 15552 | 55618 | 47093 | 64878 | 87415 | 51695 | 30800 |
| bta-miR-101 | 18978 | 18375 | 9504 | 11299 | 8941 | 9516 | 18904 | 6270 |
| bta-miR-103 | 22325 | 23353 | 14472 | 17247 | 13415 | 10993 | 3269 | 9924 |
| bta-miR-106a | 13 | 20 | 2 | 3 | 8 | 8 | 0 | 7 |
| bta-miR-106b | 4512 | 4777 | 912 | 1971 | 1300 | 516 | 215 | 795 |
| bta-miR-107 | 3622 | 3751 | 817 | 1695 | 1061 | 468 | 100 | 693 |
| bta-miR-10a | 219682 | 236219 | 47524 | 38448 | 6878 | 7333 | 137337 | 53720 |
| bta-miR-10b | 4316 | 4501 | 51734 | 58666 | 92424 | 86628 | 22267 | 29051 |
| bta-miR-1185 | 0 | 1 | 3 | 12 | 10 | 3 | 0 | 2 |
| bta-miR-1197 | 1 | 1 | 8 | 36 | 30 | 10 | 1 | 6 |
| bta-miR-122 | 62 | 59 | 58 | 43 | 15 | 26 | 42 | 56 |
| bta-miR-1224 | 1 | 0 | 2 | 1 | 2 | 1 | 0 | 0 |
| bta-miR-1246 | 756 | 709 | 38 | 31 | 9 | 14 | 47 | 66 |
| bta-miR-1247-3p | 0 | 0 | 11 | 4 | 3 | 9 | 9 | 4 |
| bta-miR-1247-5p | 0 | 0 | 7 | 15 | 10 | 5 | 2 | 20 |
| bta-miR-1248 | 40 | 39 | 7 | 21 | 20 | 1 | 1 | 5 |
| bta-miR-124a | 0 | 1 | 12 | 8 | 0 | 0 | 0 | 0 |
| bta-miR-125a | 26560 | 30624 | 15448 | 20424 | 15096 | 9685 | 1468 | 11732 |
| bta-miR-125b | 30603 | 33639 | 23496 | 34605 | 36801 | 24487 | 1992 | 14348 |
| bta-miR-1260b | 35 | 32 | 45 | 39 | 65 | 72 | 21 | 30 |
| bta-miR-126-3p | 243 | 215 | 153 | 108 | 90 | 119 | 271 | 159 |
| bta-miR-126-5p | 7 | 14 | 6 | 11 | 6 | 4 | 5 | 8 |
| bta-miR-127 | 4 | 0 | 3310 | 5541 | 6863 | 3561 | 446 | 1543 |
| bta-miR-1271 | 1233 | 1356 | 1092 | 1109 | 1128 | 1324 | 1866 | 808 |
| bta-miR-1277 | 9 | 10 | 7 | 10 | 3 | 5 | 3 | 10 |
| bta-miR-128 | 6153 | 6197 | 1158 | 2750 | 1351 | 834 | 1037 | 1692 |
| bta-miR-129 | 16 | 20 | 32 | 26 | 53 | 70 | 68 | 33 |
| bta-miR-1291 | 3 | 6 | 3 | 3 | 5 | 4 | 6 | 5 |
| bta-miR-129-3p | 9 | 12 | 25 | 23 | 23 | 19 | 5 | 27 |
| bta-miR-1296 | 17 | 23 | 12 | 9 | 26 | 15 | 1 | 7 |
| bta-miR-1298 | 3 | 1 | 160 | 62 | 56 | 80 | 354 | 207 |
| bta-miR-1301 | 4 | 4 | 0 | 1 | 1 | 1 | 0 | 2 |
| bta-miR-1306 | 107 | 89 | 98 | 79 | 77 | 100 | 20 | 69 |
| bta-miR-1307 | 4123 | 3914 | 2228 | 2035 | 1453 | 1946 | 2455 | 1699 |
| bta-miR-130a | 348 | 344 | 61 | 91 | 49 | 40 | 27 | 53 |
| bta-miR-130b | 20 | 16 | 15 | 29 | 29 | 13 | 4 | 11 |
| bta-miR-132 | 9 | 13 | 319 | 289 | 324 | 340 | 70 | 183 |
| bta-miR-133a | 2 | 0 | 20 | 47 | 140 | 45 | 0 | 24 |
| bta-miR-133b | 0 | 0 | 0 | 1 | 1 | 2 | 0 | 1 |
| bta-miR-133c | 0 | 0 | 2 | 5 | 13 | 3 | 0 | 2 |
| bta-miR-134 | 2 | 2 | 69 | 70 | 65 | 118 | 36 | 30 |
| bta-miR-1343-3p | 104 | 88 | 55 | 41 | 37 | 44 | 52 | 48 |
| bta-miR-1343-5p | 0 | 0 | 2 | 0 | 0 | 0 | 3 | 1 |
| bta-miR-135a | 413 | 426 | 36 | 34 | 2 | 1 | 38 | 42 |
| bta-miR-135b | 3 | 6 | 4 | 3 | 0 | 0 | 8 | 2 |
| bta-miR-136 | 0 | 0 | 177 | 152 | 122 | 125 | 15 | 52 |
| bta-miR-138 | 15 | 13 | 11 | 15 | 1 | 1 | 8 | 14 |
| bta-miR-1388-3p | 16 | 24 | 11 | 18 | 17 | 16 | 11 | 11 |
| bta-miR-1388-5p | 191 | 158 | 162 | 168 | 143 | 213 | 498 | 130 |
| bta-miR-139 | 15 | 14 | 15 | 13 | 14 | 12 | 7 | 9 |
| bta-miR-140 | 5349 | 5340 | 3818 | 4399 | 5101 | 5449 | 4778 | 3409 |
| bta-miR-141 | 6270 | 5665 | 982 | 1382 | 161 | 134 | 586 | 1320 |
| bta-miR-142-5p | 0 | 0 | 1 | 1 | 0 | 0 | 0 | 0 |
| bta-miR-143 | 1056 | 1062 | 54766 | 52233 | 91055 | 127510 | 40034 | 45352 |
| bta-miR-1434-3p | 4 | 8 | 4 | 9 | 3 | 0 | 4 | 1 |
| bta-miR-1434-5p | 3 | 6 | 5 | 3 | 9 | 0 | 0 | 3 |
| bta-miR-144 | 0 | 0 | 0 | 1 | 0 | 0 | 0 | 0 |
| bta-miR-145 | 19 | 31 | 1833 | 3296 | 9018 | 4168 | 167 | 1550 |
| bta-miR-1468 | 606 | 565 | 251 | 197 | 142 | 150 | 530 | 211 |
| bta-miR-146a | 17 | 8 | 166 | 328 | 148 | 50 | 34 | 392 |
| bta-miR-146b | 117 | 139 | 710 | 1159 | 1238 | 1192 | 162 | 267 |
| bta-miR-147 | 86 | 90 | 90 | 66 | 20 | 51 | 642 | 89 |
| bta-miR-148a | 43021 | 44579 | 29599 | 26254 | 28380 | 30839 | 73499 | 37361 |
| bta-miR-148b | 20470 | 19670 | 5107 | 10047 | 8297 | 3366 | 2675 | 6829 |
| bta-miR-149-3p | 0 | 0 | 0 | 0 | 0 | 0 | 1 | 1 |
| bta-miR-149-5p | 521 | 596 | 350 | 575 | 799 | 525 | 84 | 285 |
| bta-miR-150 | 2 | 2 | 0 | 1 | 0 | 1 | 0 | 0 |
| bta-miR-151-3p | 8340 | 7428 | 6346 | 5365 | 5736 | 9642 | 21709 | 5192 |
| bta-miR-151-5p | 1760 | 1877 | 571 | 749 | 671 | 463 | 472 | 560 |
| bta-miR-152 | 3483 | 2993 | 4307 | 7433 | 10657 | 5291 | 330 | 2913 |
| bta-miR-153 | 2 | 0 | 4 | 9 | 7 | 9 | 0 | 2 |
| bta-miR-154a | 0 | 0 | 1 | 2 | 0 | 1 | 0 | 0 |
| bta-miR-154b | 0 | 1 | 67 | 95 | 73 | 80 | 10 | 17 |
| bta-miR-154c | 10 | 14 | 415 | 843 | 945 | 534 | 108 | 271 |
| bta-miR-155 | 1743 | 1869 | 613 | 1067 | 1188 | 558 | 613 | 987 |
| bta-miR-15a | 791 | 692 | 117 | 202 | 169 | 88 | 38 | 120 |
| bta-miR-15b | 1149 | 1186 | 465 | 725 | 538 | 366 | 37 | 281 |
| bta-miR-16a | 11273 | 12054 | 2387 | 3751 | 3023 | 1634 | 280 | 1963 |
| bta-miR-16b | 3521 | 3560 | 1220 | 2215 | 2130 | 940 | 121 | 969 |
| bta-miR-17-3p | 59 | 65 | 14 | 36 | 18 | 35 | 25 | 15 |
| bta-miR-17-5p | 6594 | 6164 | 1419 | 3511 | 2966 | 1163 | 107 | 1127 |
| bta-miR-1814c | 42 | 36 | 9 | 23 | 47 | 23 | 14 | 18 |
| bta-miR-181a | 17076 | 19355 | 6862 | 9794 | 6755 | 3202 | 2214 | 7703 |
| bta-miR-181b | 2880 | 3218 | 739 | 1548 | 1051 | 457 | 258 | 1047 |
| bta-miR-181c | 231 | 213 | 36 | 56 | 20 | 10 | 10 | 38 |
| bta-miR-181d | 548 | 534 | 80 | 164 | 122 | 49 | 33 | 95 |
| bta-miR-182 | 29106 | 28605 | 6771 | 7431 | 869 | 586 | 9165 | 7967 |
| bta-miR-183 | 7167 | 7284 | 2383 | 2312 | 255 | 197 | 9288 | 2794 |
| bta-miR-1839 | 7817 | 7779 | 2656 | 2285 | 1200 | 1321 | 3818 | 2917 |
| bta-miR-184 | 806790 | 858084 | 265428 | 283653 | 15754 | 16605 | 1621400 | 364740 |
| bta-miR-1842 | 9 | 10 | 9 | 8 | 16 | 9 | 6 | 6 |
| bta-miR-185 | 7890 | 8008 | 2811 | 4579 | 4317 | 2696 | 652 | 2064 |
| bta-miR-186 | 12761 | 13337 | 3096 | 7501 | 4749 | 1713 | 1411 | 3830 |
| bta-miR-187 | 0 | 0 | 0 | 1 | 0 | 0 | 1 | 0 |
| bta-miR-188 | 13 | 13 | 43 | 62 | 65 | 66 | 9 | 17 |
| bta-miR-18a | 190 | 174 | 76 | 113 | 106 | 104 | 48 | 59 |
| bta-miR-18b | 4 | 1 | 0 | 1 | 0 | 1 | 1 | 0 |
| bta-miR-190a | 23 | 18 | 3 | 13 | 4 | 3 | 2 | 6 |
| bta-miR-190b | 0 | 0 | 1 | 0 | 3 | 1 | 2 | 0 |
| bta-miR-191 | 15623 | 15649 | 3721 | 6075 | 3134 | 1826 | 904 | 3467 |
| bta-miR-192 | 5735 | 5887 | 2674 | 2347 | 1391 | 1651 | 5506 | 2300 |
| bta-miR-193a | 29 | 23 | 11 | 12 | 4 | 5 | 4 | 6 |
| bta-miR-193a-3p | 36 | 34 | 26 | 18 | 12 | 13 | 20 | 17 |
| bta-miR-193a-5p | 704 | 744 | 258 | 238 | 211 | 246 | 124 | 210 |
| bta-miR-193b | 513 | 667 | 759 | 1210 | 1927 | 1047 | 463 | 902 |
| bta-miR-194 | 4772 | 5692 | 991 | 1624 | 917 | 356 | 46 | 788 |
| bta-miR-195 | 23 | 29 | 46 | 69 | 91 | 67 | 5 | 63 |
| bta-miR-196a | 147 | 146 | 34 | 46 | 18 | 23 | 5 | 26 |
| bta-miR-196b | 49 | 41 | 839 | 1436 | 2096 | 1100 | 25 | 318 |
| bta-miR-197 | 247 | 234 | 262 | 227 | 205 | 311 | 333 | 246 |
| bta-miR-199a-3p | 7 | 1 | 21130 | 45195 | 55890 | 23647 | 1795 | 15705 |
| bta-miR-199a-5p | 4 | 2 | 11173 | 26866 | 34442 | 21539 | 1025 | 5906 |
| bta-miR-199b | 0 | 1 | 6145 | 9992 | 5783 | 4019 | 683 | 4301 |
| bta-miR-199c | 0 | 0 | 327 | 1156 | 1185 | 257 | 65 | 396 |
| bta-miR-19a | 95 | 94 | 46 | 85 | 76 | 80 | 21 | 41 |
| bta-miR-19b | 1738 | 1498 | 380 | 782 | 660 | 346 | 183 | 318 |
| bta-miR-200a | 471039 | 473770 | 70158 | 112862 | 9371 | 6210 | 68101 | 107154 |
| bta-miR-200b | 199379 | 199862 | 41571 | 42246 | 3004 | 2933 | 22562 | 40142 |
| bta-miR-200c | 55261 | 56661 | 10991 | 10702 | 982 | 852 | 9573 | 10837 |
| bta-miR-202 | 3 | 2 | 2 | 0 | 1 | 0 | 0 | 2 |
| bta-miR-204 | 10 | 7 | 761 | 1499 | 2594 | 1962 | 228 | 543 |
| bta-miR-205 | 2478 | 2474 | 654 | 2214 | 19 | 12 | 101 | 319 |
| bta-miR-206 | 1 | 0 | 1 | 0 | 0 | 0 | 0 | 0 |
| bta-miR-20a | 19572 | 18108 | 5116 | 9770 | 8377 | 4180 | 529 | 3352 |
| bta-miR-20b | 16 | 11 | 2 | 6 | 5 | 5 | 0 | 2 |
| bta-miR-210 | 4668 | 4641 | 273 | 661 | 391 | 110 | 77 | 306 |
| bta-miR-211 | 3 | 2 | 0 | 6 | 0 | 0 | 14 | 17 |
| bta-miR-212 | 2 | 0 | 32 | 55 | 47 | 46 | 24 | 18 |
| bta-miR-21-3p | 185 | 193 | 228 | 489 | 424 | 250 | 124 | 199 |
| bta-miR-214 | 0 | 1 | 550 | 850 | 1443 | 874 | 81 | 352 |
| bta-miR-215 | 61 | 59 | 52 | 54 | 61 | 44 | 30 | 38 |
| bta-miR-21-5p | 3181410 | 3529090 | 3858697 | 3370288 | 3339547 | 4701230 | 4207822 | 2255390 |
| bta-miR-216b | 0 | 0 | 2 | 1 | 0 | 0 | 2 | 3 |
| bta-miR-218 | 418 | 443 | 79 | 70 | 88 | 89 | 102 | 76 |
| bta-miR-219 | 9 | 7 | 6 | 6 | 2 | 9 | 33 | 3 |
| bta-miR-219-3p | 1 | 3 | 5 | 3 | 3 | 1 | 0 | 1 |
| bta-miR-219-5p | 1 | 3 | 1 | 3 | 2 | 7 | 1 | 0 |
| bta-miR-221 | 35726 | 41514 | 27008 | 52851 | 72997 | 32607 | 1893 | 22024 |
| bta-miR-222 | 11513 | 13665 | 10334 | 19207 | 35017 | 21641 | 2169 | 7185 |
| bta-miR-223 | 0 | 1 | 1 | 0 | 1 | 0 | 0 | 1 |
| bta-miR-22-3p | 31190 | 31867 | 9834 | 12718 | 9268 | 12883 | 18175 | 15270 |
| bta-miR-224 | 36510 | 35938 | 2721 | 3871 | 786 | 757 | 3534 | 2613 |
| bta-miR-22-5p | 732 | 843 | 202 | 330 | 220 | 181 | 499 | 308 |
| bta-miR-2284a | 0 | 0 | 1 | 0 | 0 | 0 | 0 | 1 |
| bta-miR-2284aa | 4 | 6 | 5 | 2 | 2 | 0 | 0 | 3 |
| bta-miR-2284ab | 45 | 64 | 33 | 34 | 20 | 15 | 5 | 18 |
| bta-miR-2284ac | 0 | 1 | 0 | 2 | 0 | 1 | 0 | 0 |
| bta-miR-2284c | 0 | 0 | 0 | 1 | 0 | 0 | 0 | 0 |
| bta-miR-2284d | 2 | 2 | 0 | 0 | 1 | 0 | 0 | 0 |
| bta-miR-2284h-5p | 47 | 33 | 13 | 9 | 3 | 8 | 14 | 25 |
| bta-miR-2284j | 2 | 2 | 0 | 0 | 0 | 0 | 1 | 2 |
| bta-miR-2284k | 1 | 3 | 0 | 0 | 0 | 1 | 3 | 0 |
| bta-miR-2284l | 1 | 0 | 1 | 0 | 0 | 0 | 0 | 0 |
| bta-miR-2284m | 1 | 0 | 0 | 0 | 0 | 0 | 0 | 0 |
| bta-miR-2284n | 1 | 3 | 0 | 0 | 0 | 0 | 0 | 0 |
| bta-miR-2284p | 8 | 9 | 7 | 9 | 5 | 0 | 0 | 5 |
| bta-miR-2284r | 0 | 0 | 0 | 0 | 0 | 1 | 0 | 0 |
| bta-miR-2284t-3p | 0 | 0 | 0 | 1 | 3 | 1 | 0 | 0 |
| bta-miR-2284t-5p | 0 | 0 | 0 | 0 | 2 | 0 | 0 | 1 |
| bta-miR-2284u | 1 | 2 | 0 | 0 | 0 | 0 | 0 | 1 |
| bta-miR-2284v | 1 | 0 | 0 | 0 | 0 | 0 | 0 | 1 |
| bta-miR-2284w | 3 | 3 | 1 | 6 | 9 | 6 | 0 | 1 |
| bta-miR-2284x | 13366 | 12701 | 4099 | 5827 | 4829 | 2771 | 4024 | 5327 |
| bta-miR-2284y | 464 | 437 | 167 | 180 | 129 | 115 | 173 | 181 |
| bta-miR-2284z | 4 | 10 | 5 | 1 | 2 | 1 | 0 | 3 |
| bta-miR-2285a | 5 | 6 | 4 | 2 | 0 | 0 | 2 | 2 |
| bta-miR-2285aa | 127 | 121 | 150 | 110 | 114 | 94 | 190 | 88 |
| bta-miR-2285ab | 206 | 246 | 81 | 69 | 59 | 44 | 121 | 91 |
| bta-miR-2285ac | 8 | 5 | 6 | 3 | 2 | 10 | 3 | 3 |
| bta-miR-2285ad | 4 | 4 | 1 | 3 | 4 | 3 | 2 | 2 |
| bta-miR-2285af | 41 | 38 | 24 | 24 | 31 | 28 | 20 | 16 |
| bta-miR-2285b | 68 | 79 | 76 | 66 | 54 | 64 | 141 | 62 |
| bta-miR-2285c | 9 | 9 | 4 | 3 | 3 | 2 | 7 | 4 |
| bta-miR-2285e | 148 | 118 | 55 | 47 | 27 | 25 | 175 | 84 |
| bta-miR-2285f | 214 | 175 | 614 | 394 | 544 | 1265 | 540 | 438 |
| bta-miR-2285g | 41 | 38 | 24 | 24 | 31 | 28 | 20 | 16 |
| bta-miR-2285h | 8 | 15 | 10 | 13 | 6 | 3 | 5 | 4 |
| bta-miR-2285i | 5 | 1 | 2 | 3 | 6 | 2 | 5 | 7 |
| bta-miR-2285j | 28 | 42 | 15 | 25 | 16 | 9 | 5 | 18 |
| bta-miR-2285k | 68 | 72 | 114 | 95 | 116 | 156 | 98 | 75 |
| bta-miR-2285l | 21 | 17 | 15 | 10 | 4 | 4 | 7 | 13 |
| bta-miR-2285m | 1 | 2 | 0 | 1 | 0 | 0 | 0 | 1 |
| bta-miR-2285n | 5 | 4 | 1 | 2 | 0 | 0 | 1 | 1 |
| bta-miR-2285o | 416 | 390 | 133 | 149 | 91 | 55 | 142 | 163 |
| bta-miR-2285p | 143 | 145 | 58 | 57 | 5 | 3 | 132 | 47 |
| bta-miR-2285q | 91 | 88 | 111 | 86 | 97 | 119 | 318 | 77 |
| bta-miR-2285r | 0 | 0 | 3 | 4 | 6 | 8 | 2 | 2 |
| bta-miR-2285s | 0 | 0 | 2 | 2 | 0 | 4 | 1 | 2 |
| bta-miR-2285t | 1 | 2 | 1 | 2 | 0 | 0 | 16 | 5 |
| bta-miR-2285u | 462 | 433 | 169 | 182 | 128 | 116 | 174 | 183 |
| bta-miR-2285v | 5 | 8 | 5 | 7 | 10 | 9 | 10 | 6 |
| bta-miR-2285w | 0 | 2 | 1 | 2 | 3 | 3 | 6 | 3 |
| bta-miR-2285x | 2 | 2 | 4 | 1 | 3 | 4 | 16 | 4 |
| bta-miR-2285y | 4 | 9 | 5 | 1 | 1 | 1 | 0 | 3 |
| bta-miR-2285z | 91 | 88 | 111 | 86 | 97 | 119 | 318 | 77 |
| bta-miR-2289 | 1 | 1 | 2 | 4 | 1 | 0 | 0 | 0 |
| bta-miR-2290 | 1 | 2 | 0 | 0 | 0 | 4 | 0 | 1 |
| bta-miR-2299-3p | 17 | 19 | 20 | 20 | 34 | 19 | 2 | 6 |
| bta-miR-2299-5p | 25 | 30 | 21 | 33 | 33 | 39 | 47 | 29 |
| bta-miR-2300a-5p | 1 | 0 | 0 | 0 | 0 | 0 | 0 | 1 |
| bta-miR-2300b-3p | 0 | 0 | 0 | 0 | 0 | 0 | 0 | 1 |
| bta-miR-2307 | 15 | 18 | 18 | 11 | 5 | 13 | 51 | 11 |
| bta-miR-2308 | 5 | 3 | 2 | 0 | 2 | 0 | 7 | 1 |
| bta-miR-2309 | 0 | 0 | 1 | 0 | 0 | 0 | 0 | 0 |
| bta-miR-2310 | 42 | 36 | 9 | 24 | 48 | 24 | 14 | 18 |
| bta-miR-2311 | 17 | 12 | 12 | 9 | 12 | 4 | 18 | 6 |
| bta-miR-2312 | 2 | 2 | 0 | 0 | 0 | 0 | 1 | 2 |
| bta-miR-2314 | 0 | 3 | 3 | 2 | 1 | 1 | 3 | 4 |
| bta-miR-2318 | 35 | 29 | 66 | 38 | 26 | 43 | 228 | 43 |
| bta-miR-2320-3p | 10 | 11 | 9 | 11 | 13 | 11 | 16 | 3 |
| bta-miR-2320-5p | 2 | 6 | 4 | 8 | 8 | 6 | 0 | 2 |
| bta-miR-2323 | 3 | 3 | 3 | 5 | 2 | 0 | 3 | 1 |
| bta-miR-2328-3p | 0 | 0 | 0 | 1 | 0 | 0 | 0 | 0 |
| bta-miR-2329-3p | 3 | 0 | 0 | 0 | 0 | 0 | 0 | 1 |
| bta-miR-2329-5p | 9 | 5 | 2 | 1 | 1 | 2 | 6 | 2 |
| bta-miR-2330-3p | 0 | 2 | 0 | 2 | 1 | 0 | 0 | 0 |
| bta-miR-2330-5p | 0 | 0 | 0 | 0 | 2 | 3 | 0 | 0 |
| bta-miR-2331-3p | 6 | 6 | 0 | 1 | 3 | 1 | 0 | 1 |
| bta-miR-2331-5p | 3 | 4 | 2 | 0 | 1 | 0 | 0 | 0 |
| bta-miR-2332 | 13 | 14 | 15 | 10 | 3 | 3 | 5 | 7 |
| bta-miR-2335 | 4 | 2 | 6 | 7 | 2 | 13 | 32 | 8 |
| bta-miR-2336 | 163 | 171 | 84 | 132 | 104 | 80 | 260 | 107 |
| bta-miR-2338 | 1 | 0 | 0 | 0 | 1 | 0 | 0 | 0 |
| bta-miR-2339 | 13 | 10 | 6 | 10 | 1 | 3 | 34 | 6 |
| bta-miR-2342 | 0 | 0 | 0 | 0 | 0 | 2 | 2 | 0 |
| bta-miR-2343 | 1 | 1 | 0 | 0 | 0 | 0 | 0 | 0 |
| bta-miR-2344 | 33 | 37 | 51 | 63 | 64 | 43 | 20 | 48 |
| bta-miR-2346 | 4 | 1 | 3 | 1 | 2 | 1 | 0 | 4 |
| bta-miR-2348 | 0 | 1 | 0 | 1 | 0 | 0 | 0 | 1 |
| bta-miR-2349 | 5 | 13 | 10 | 8 | 3 | 4 | 11 | 15 |
| bta-miR-2350 | 0 | 1 | 1 | 0 | 1 | 2 | 0 | 0 |
| bta-miR-2353 | 0 | 0 | 1 | 0 | 0 | 1 | 0 | 0 |
| bta-miR-2355-3p | 40 | 42 | 36 | 60 | 58 | 38 | 23 | 35 |
| bta-miR-2357 | 2 | 0 | 3 | 2 | 0 | 0 | 34 | 1 |
| bta-miR-2366 | 0 | 0 | 2 | 0 | 2 | 1 | 0 | 0 |
| bta-miR-2367-3p | 2 | 1 | 0 | 0 | 0 | 0 | 0 | 0 |
| bta-miR-2367-5p | 0 | 1 | 0 | 0 | 0 | 1 | 0 | 0 |
| bta-miR-2368-3p | 1 | 1 | 0 | 0 | 0 | 0 | 1 | 0 |
| bta-miR-2370-3p | 2 | 6 | 2 | 6 | 3 | 4 | 1 | 0 |
| bta-miR-2370-5p | 5 | 2 | 2 | 1 | 2 | 0 | 0 | 1 |
| bta-miR-2373-3p | 1 | 1 | 0 | 0 | 0 | 0 | 0 | 0 |
| bta-miR-2373-5p | 0 | 0 | 0 | 0 | 0 | 0 | 0 | 1 |
| bta-miR-2376 | 5 | 6 | 2 | 2 | 1 | 0 | 11 | 5 |
| bta-miR-2377 | 0 | 0 | 0 | 0 | 0 | 0 | 0 | 1 |
| bta-miR-2378 | 1 | 0 | 0 | 0 | 0 | 0 | 0 | 0 |
| bta-miR-2379 | 0 | 0 | 0 | 0 | 1 | 1 | 0 | 2 |
| bta-miR-2381 | 1 | 2 | 0 | 0 | 0 | 0 | 0 | 0 |
| bta-miR-2382-3p | 1 | 0 | 2 | 0 | 0 | 0 | 0 | 0 |
| bta-miR-2382-5p | 1 | 0 | 0 | 0 | 3 | 0 | 0 | 0 |
| bta-miR-2387 | 329 | 275 | 230 | 287 | 289 | 298 | 233 | 220 |
| bta-miR-2388-3p | 0 | 1 | 3 | 0 | 0 | 0 | 0 | 1 |
| bta-miR-2388-5p | 0 | 1 | 1 | 3 | 2 | 1 | 5 | 1 |
| bta-miR-2394 | 7 | 6 | 1 | 0 | 3 | 0 | 0 | 3 |
| bta-miR-2395 | 5 | 2 | 1 | 3 | 2 | 0 | 2 | 1 |
| bta-miR-2396 | 2 | 1 | 3 | 2 | 0 | 0 | 1 | 2 |
| bta-miR-2397-3p | 2 | 3 | 3 | 4 | 1 | 1 | 0 | 1 |
| bta-miR-2397-5p | 3 | 2 | 3 | 0 | 0 | 1 | 2 | 2 |
| bta-miR-2398 | 1 | 1 | 0 | 2 | 3 | 1 | 3 | 0 |
| bta-miR-2399-3p | 1 | 2 | 2 | 2 | 2 | 2 | 0 | 0 |
| bta-miR-2399-5p | 19 | 21 | 13 | 15 | 27 | 16 | 53 | 20 |
| bta-miR-23a | 40544 | 42442 | 15872 | 21464 | 16085 | 11893 | 4921 | 18566 |
| bta-miR-23b-3p | 18650 | 19002 | 7368 | 9950 | 8229 | 7183 | 2485 | 7761 |
| bta-miR-23b-5p | 182 | 226 | 37 | 77 | 132 | 45 | 7 | 31 |
| bta-miR-24 | 487 | 526 | 297 | 329 | 323 | 223 | 168 | 225 |
| bta-miR-2400 | 6 | 5 | 1 | 3 | 2 | 2 | 0 | 1 |
| bta-miR-2403 | 18 | 5 | 7 | 6 | 10 | 4 | 2 | 7 |
| bta-miR-2404 | 19 | 21 | 10 | 13 | 12 | 6 | 20 | 10 |
| bta-miR-2407 | 1 | 2 | 0 | 0 | 0 | 0 | 0 | 0 |
| bta-miR-2408 | 6 | 2 | 6 | 4 | 1 | 3 | 7 | 3 |
| bta-miR-2409 | 2 | 1 | 0 | 0 | 0 | 0 | 0 | 0 |
| bta-miR-2410 | 3 | 1 | 0 | 1 | 0 | 1 | 2 | 1 |
| bta-miR-2411-3p | 22 | 20 | 9 | 11 | 7 | 7 | 27 | 14 |
| bta-miR-2411-5p | 100 | 108 | 80 | 55 | 14 | 23 | 118 | 69 |
| bta-miR-2415-3p | 1 | 4 | 1 | 1 | 1 | 2 | 1 | 0 |
| bta-miR-2415-5p | 0 | 0 | 0 | 1 | 0 | 0 | 1 | 0 |
| bta-miR-2416 | 8 | 13 | 17 | 14 | 2 | 10 | 63 | 12 |
| bta-miR-2417 | 1 | 0 | 0 | 0 | 0 | 0 | 0 | 0 |
| bta-miR-2419-5p | 70 | 90 | 15 | 18 | 11 | 8 | 25 | 20 |
| bta-miR-2422 | 0 | 2 | 1 | 0 | 4 | 1 | 0 | 2 |
| bta-miR-2424 | 24 | 19 | 10 | 11 | 10 | 5 | 0 | 7 |
| bta-miR-2425-5p | 1 | 1 | 0 | 1 | 1 | 0 | 0 | 0 |
| bta-miR-2427 | 1 | 0 | 0 | 0 | 0 | 0 | 0 | 0 |
| bta-miR-2431-3p | 8 | 17 | 16 | 8 | 14 | 11 | 2 | 8 |
| bta-miR-2431-5p | 46 | 52 | 22 | 36 | 22 | 13 | 6 | 18 |
| bta-miR-2432 | 2 | 1 | 1 | 1 | 0 | 0 | 1 | 0 |
| bta-miR-2435 | 1 | 0 | 0 | 0 | 1 | 0 | 5 | 3 |
| bta-miR-2436-3p | 1 | 0 | 0 | 0 | 0 | 0 | 0 | 0 |
| bta-miR-2438 | 1 | 0 | 0 | 1 | 0 | 0 | 0 | 0 |
| bta-miR-2439-3p | 0 | 0 | 0 | 0 | 0 | 0 | 1 | 0 |
| bta-miR-24-3p | 62809 | 63547 | 52342 | 42778 | 28497 | 48710 | 77415 | 36891 |
| bta-miR-2440 | 0 | 0 | 0 | 0 | 0 | 1 | 1 | 1 |
| bta-miR-2441 | 0 | 0 | 1 | 0 | 0 | 0 | 1 | 0 |
| bta-miR-2443 | 64 | 71 | 40 | 58 | 46 | 43 | 35 | 31 |
| bta-miR-2446 | 0 | 0 | 0 | 0 | 0 | 0 | 1 | 0 |
| bta-miR-2447 | 1 | 0 | 1 | 1 | 4 | 0 | 0 | 2 |
| bta-miR-2448-3p | 17 | 13 | 6 | 10 | 17 | 3 | 2 | 8 |
| bta-miR-2448-5p | 0 | 1 | 0 | 3 | 1 | 1 | 0 | 2 |
| bta-miR-2450a | 4 | 5 | 3 | 2 | 2 | 3 | 4 | 0 |
| bta-miR-2450b | 0 | 0 | 2 | 1 | 0 | 0 | 1 | 1 |
| bta-miR-2451 | 0 | 0 | 1 | 0 | 0 | 0 | 1 | 0 |
| bta-miR-2453 | 0 | 0 | 0 | 0 | 0 | 0 | 0 | 1 |
| bta-miR-2454-3p | 0 | 1 | 0 | 1 | 0 | 0 | 1 | 0 |
| bta-miR-2454-5p | 0 | 0 | 0 | 0 | 0 | 0 | 1 | 0 |
| bta-miR-2455 | 0 | 0 | 0 | 0 | 1 | 0 | 0 | 0 |
| bta-miR-2457 | 5 | 6 | 1 | 1 | 1 | 1 | 3 | 1 |
| bta-miR-2459 | 1 | 1 | 0 | 2 | 1 | 0 | 1 | 1 |
| bta-miR-2460 | 0 | 0 | 2 | 0 | 0 | 1 | 1 | 0 |
| bta-miR-2461-3p | 0 | 2 | 1 | 1 | 1 | 3 | 1 | 2 |
| bta-miR-2462 | 0 | 0 | 0 | 1 | 0 | 0 | 1 | 0 |
| bta-miR-2463 | 7 | 6 | 2 | 0 | 3 | 1 | 0 | 2 |
| bta-miR-2465 | 0 | 0 | 1 | 0 | 0 | 0 | 0 | 0 |
| bta-miR-2466-3p | 1 | 1 | 0 | 1 | 1 | 0 | 1 | 0 |
| bta-miR-2466-5p | 1 | 3 | 2 | 11 | 7 | 9 | 3 | 2 |
| bta-miR-2467-3p | 0 | 0 | 0 | 0 | 0 | 0 | 1 | 0 |
| bta-miR-2468 | 1 | 3 | 3 | 5 | 1 | 3 | 1 | 2 |
| bta-miR-2469 | 1 | 1 | 2 | 0 | 1 | 0 | 2 | 0 |
| bta-miR-2473 | 0 | 1 | 0 | 0 | 0 | 0 | 0 | 0 |
| bta-miR-2474 | 23 | 26 | 27 | 7 | 2 | 6 | 85 | 15 |
| bta-miR-2475 | 15 | 8 | 14 | 14 | 12 | 12 | 19 | 11 |
| bta-miR-2477 | 3 | 5 | 20 | 5 | 15 | 26 | 68 | 18 |
| bta-miR-2478 | 489 | 540 | 325 | 363 | 456 | 528 | 118 | 263 |
| bta-miR-2481 | 0 | 0 | 1 | 0 | 2 | 1 | 0 | 0 |
| bta-miR-2483-3p | 14 | 7 | 4 | 1 | 2 | 2 | 3 | 4 |
| bta-miR-2483-5p | 59 | 63 | 9 | 16 | 8 | 9 | 14 | 17 |
| bta-miR-2484 | 102 | 112 | 40 | 42 | 22 | 32 | 329 | 47 |
| bta-miR-2485 | 0 | 0 | 0 | 2 | 0 | 0 | 0 | 0 |
| bta-miR-2486-3p | 0 | 0 | 0 | 0 | 1 | 0 | 0 | 0 |
| bta-miR-25 | 16749 | 15786 | 5505 | 7090 | 5131 | 4142 | 1420 | 3732 |
| bta-miR-26a | 142295 | 152332 | 39332 | 72340 | 74010 | 39851 | 12741 | 42111 |
| bta-miR-26b | 14029 | 13698 | 3993 | 6772 | 6654 | 3449 | 888 | 4659 |
| bta-miR-26c | 142274 | 152320 | 39329 | 72334 | 73998 | 39848 | 12741 | 42106 |
| bta-miR-27a-3p | 76933 | 68894 | 40657 | 54025 | 28730 | 24936 | 18298 | 36197 |
| bta-miR-27a-5p | 3840 | 4264 | 2085 | 1757 | 2322 | 2384 | 2303 | 1108 |
| bta-miR-27b | 296306 | 270830 | 196376 | 239971 | 252670 | 229227 | 84552 | 141773 |
| bta-miR-28 | 1265 | 1218 | 978 | 1385 | 1916 | 1636 | 575 | 676 |
| bta-miR-2887 | 8 | 10 | 5 | 1 | 1 | 1 | 2 | 5 |
| bta-miR-2889 | 11 | 14 | 7 | 7 | 4 | 3 | 2 | 6 |
| bta-miR-2890 | 0 | 2 | 0 | 0 | 0 | 0 | 0 | 0 |
| bta-miR-2898 | 1000 | 962 | 488 | 538 | 789 | 834 | 112 | 379 |
| bta-miR-2904 | 52 | 37 | 14 | 25 | 5 | 9 | 11 | 31 |
| bta-miR-2957 | 3483 | 2992 | 4307 | 7433 | 10657 | 5291 | 330 | 2913 |
| bta-miR-296-3p | 130 | 129 | 103 | 126 | 446 | 387 | 94 | 100 |
| bta-miR-299 | 3 | 0 | 24 | 37 | 34 | 18 | 1 | 11 |
| bta-miR-29a | 96703 | 99598 | 22361 | 46887 | 31656 | 16047 | 7810 | 24998 |
| bta-miR-29b | 3849 | 3640 | 1034 | 1435 | 554 | 484 | 886 | 1344 |
| bta-miR-29c | 783 | 758 | 83 | 251 | 46 | 20 | 55 | 143 |
| bta-miR-29d-3p | 21 | 12 | 1 | 5 | 2 | 0 | 0 | 6 |
| bta-miR-29d-5p | 177 | 151 | 51 | 55 | 16 | 16 | 144 | 107 |
| bta-miR-29e | 1 | 2 | 3 | 1 | 2 | 2 | 3 | 5 |
| bta-miR-301a | 9 | 7 | 6 | 11 | 17 | 5 | 2 | 8 |
| bta-miR-301b | 1 | 0 | 2 | 4 | 12 | 5 | 0 | 2 |
| bta-miR-302a | 1 | 0 | 0 | 2 | 0 | 0 | 0 | 0 |
| bta-miR-302d | 1 | 0 | 0 | 2 | 0 | 0 | 0 | 0 |
| bta-miR-3064 | 5 | 1 | 0 | 0 | 0 | 0 | 1 | 1 |
| bta-miR-30a-5p | 125941 | 123127 | 30161 | 40695 | 5215 | 4359 | 103505 | 56191 |
| bta-miR-30b-3p | 17 | 30 | 2 | 7 | 7 | 5 | 0 | 1 |
| bta-miR-30b-5p | 3612 | 3779 | 515 | 1453 | 1152 | 400 | 67 | 623 |
| bta-miR-30c | 7543 | 7941 | 1736 | 2769 | 2349 | 1351 | 293 | 1508 |
| bta-miR-30d | 36832 | 37160 | 14003 | 18067 | 18143 | 14890 | 19728 | 14781 |
| bta-miR-30e-5p | 10151 | 10372 | 4487 | 7665 | 7324 | 5034 | 2335 | 5913 |
| bta-miR-30f | 5751 | 6399 | 841 | 1245 | 118 | 65 | 429 | 1242 |
| bta-miR-31 | 9778 | 10514 | 1588 | 2875 | 666 | 319 | 369 | 1920 |
| bta-miR-3120 | 1 | 3 | 97 | 102 | 141 | 165 | 23 | 47 |
| bta-miR-3154 | 0 | 0 | 0 | 2 | 0 | 0 | 0 | 0 |
| bta-miR-32 | 1278 | 1214 | 1612 | 1627 | 1560 | 1903 | 385 | 941 |
| bta-miR-320a | 3017 | 3154 | 1850 | 1941 | 1950 | 1941 | 867 | 1225 |
| bta-miR-323 | 3 | 3 | 27 | 51 | 45 | 21 | 5 | 14 |
| bta-miR-324 | 28 | 32 | 17 | 47 | 23 | 19 | 13 | 22 |
| bta-miR-326 | 4 | 3 | 5 | 13 | 11 | 7 | 7 | 2 |
| bta-miR-328 | 244 | 230 | 113 | 213 | 229 | 129 | 29 | 71 |
| bta-miR-329a | 0 | 0 | 0 | 7 | 1 | 0 | 0 | 0 |
| bta-miR-330 | 71 | 56 | 28 | 33 | 38 | 32 | 24 | 23 |
| bta-miR-331-3p | 308 | 277 | 117 | 203 | 188 | 123 | 16 | 99 |
| bta-miR-331-5p | 146 | 156 | 44 | 68 | 58 | 29 | 7 | 38 |
| bta-miR-335 | 2 | 2 | 7 | 6 | 1 | 0 | 0 | 1 |
| bta-miR-338 | 1 | 1 | 1 | 2 | 1 | 0 | 0 | 0 |
| bta-miR-339a | 2386 | 2466 | 573 | 1551 | 1869 | 848 | 33 | 469 |
| bta-miR-339b | 745 | 761 | 146 | 382 | 275 | 145 | 21 | 153 |
| bta-miR-33a | 7 | 3 | 10 | 10 | 2 | 3 | 9 | 9 |
| bta-miR-33b | 1 | 1 | 0 | 0 | 0 | 0 | 0 | 1 |
| bta-miR-340 | 228 | 253 | 214 | 130 | 93 | 156 | 304 | 146 |
| bta-miR-342 | 3226 | 3674 | 1320 | 2052 | 1952 | 1123 | 944 | 1624 |
| bta-miR-3431 | 50221 | 52259 | 4542 | 7235 | 1700 | 1024 | 3555 | 4398 |
| bta-miR-3432a | 526 | 498 | 1323 | 1729 | 1890 | 1475 | 397 | 759 |
| bta-miR-3432b | 0 | 0 | 3 | 6 | 3 | 1 | 0 | 1 |
| bta-miR-345-3p | 241 | 227 | 43 | 82 | 37 | 24 | 21 | 32 |
| bta-miR-345-5p | 176 | 167 | 21 | 69 | 53 | 8 | 9 | 27 |
| bta-miR-346 | 1 | 0 | 2 | 3 | 4 | 0 | 0 | 2 |
| bta-miR-34a | 11215 | 11709 | 3006 | 7897 | 7010 | 2389 | 474 | 3906 |
| bta-miR-34b | 1 | 0 | 0 | 2 | 2 | 4 | 0 | 1 |
| bta-miR-34c | 275 | 295 | 196 | 197 | 426 | 377 | 88 | 166 |
| bta-miR-3533 | 5 | 4 | 2 | 5 | 0 | 2 | 18 | 2 |
| bta-miR-3578 | 1 | 2 | 223 | 192 | 158 | 164 | 31 | 70 |
| bta-miR-3596 | 119104 | 117536 | 22094 | 49512 | 44533 | 17288 | 6853 | 21572 |
| bta-miR-3600 | 31190 | 31867 | 9834 | 12718 | 9268 | 12883 | 18175 | 15270 |
| bta-miR-3601 | 3 | 2 | 0 | 1 | 5 | 5 | 3 | 3 |
| bta-miR-3604 | 7 | 1 | 21126 | 45184 | 55868 | 23635 | 1794 | 15703 |
| bta-miR-361 | 815 | 831 | 218 | 338 | 364 | 242 | 55 | 196 |
| bta-miR-362-3p | 27 | 30 | 17 | 29 | 36 | 8 | 2 | 10 |
| bta-miR-362-5p | 440 | 506 | 284 | 478 | 699 | 349 | 60 | 234 |
| bta-miR-363 | 0 | 4 | 1 | 0 | 0 | 0 | 5 | 0 |
| bta-miR-365-3p | 2308 | 2510 | 1423 | 2583 | 3234 | 1492 | 406 | 1600 |
| bta-miR-365-5p | 94 | 93 | 323 | 355 | 736 | 688 | 218 | 212 |
| bta-miR-369-3p | 10 | 12 | 294 | 783 | 703 | 273 | 10 | 218 |
| bta-miR-369-5p | 0 | 1 | 78 | 63 | 58 | 77 | 19 | 29 |
| bta-miR-370 | 0 | 0 | 82 | 84 | 177 | 220 | 75 | 31 |
| bta-miR-371 | 0 | 0 | 0 | 0 | 0 | 0 | 0 | 1 |
| bta-miR-374a | 2368 | 1903 | 290 | 951 | 940 | 246 | 30 | 263 |
| bta-miR-374b | 7141 | 7233 | 2167 | 4034 | 4414 | 2027 | 310 | 1478 |
| bta-miR-375 | 5238 | 5344 | 1165 | 855 | 97 | 76 | 2580 | 1085 |
| bta-miR-376a | 0 | 2 | 4 | 6 | 9 | 5 | 0 | 1 |
| bta-miR-376b | 0 | 0 | 8 | 6 | 13 | 7 | 1 | 3 |
| bta-miR-376c | 2 | 0 | 3 | 21 | 17 | 8 | 0 | 4 |
| bta-miR-376d | 1 | 0 | 6 | 17 | 22 | 3 | 0 | 13 |
| bta-miR-376e | 2 | 3 | 47 | 97 | 103 | 35 | 3 | 25 |
| bta-miR-377 | 0 | 0 | 11 | 6 | 5 | 4 | 1 | 3 |
| bta-miR-378 | 57774 | 54364 | 20190 | 17622 | 7828 | 11469 | 48031 | 14171 |
| bta-miR-378b | 4751 | 4518 | 5045 | 4434 | 1314 | 2384 | 8183 | 4016 |
| bta-miR-378c | 13179 | 11465 | 2622 | 3222 | 879 | 979 | 5211 | 2330 |
| bta-miR-378d | 316 | 317 | 56 | 110 | 39 | 30 | 148 | 93 |
| bta-miR-379 | 84 | 81 | 3954 | 3611 | 3309 | 4688 | 1482 | 1377 |
| bta-miR-380-3p | 4 | 5 | 66 | 155 | 159 | 72 | 5 | 40 |
| bta-miR-380-5p | 1 | 0 | 0 | 5 | 6 | 5 | 1 | 2 |
| bta-miR-381 | 4 | 11 | 648 | 599 | 809 | 1447 | 564 | 511 |
| bta-miR-382 | 1 | 2 | 232 | 222 | 184 | 179 | 33 | 80 |
| bta-miR-3956 | 0 | 0 | 17 | 15 | 9 | 16 | 27 | 8 |
| bta-miR-3957 | 0 | 1 | 9 | 10 | 6 | 7 | 2 | 2 |
| bta-miR-409a | 5 | 11 | 436 | 610 | 519 | 447 | 20 | 200 |
| bta-miR-409b | 8 | 9 | 470 | 705 | 854 | 701 | 215 | 230 |
| bta-miR-410 | 2 | 0 | 35 | 102 | 119 | 31 | 1 | 20 |
| bta-miR-411a | 88 | 67 | 2893 | 2578 | 2454 | 3036 | 242 | 957 |
| bta-miR-411b | 9 | 5 | 115 | 229 | 153 | 83 | 18 | 62 |
| bta-miR-411c-3p | 0 | 1 | 3 | 20 | 20 | 5 | 0 | 5 |
| bta-miR-411c-5p | 5 | 4 | 82 | 159 | 137 | 80 | 3 | 45 |
| bta-miR-412 | 0 | 0 | 1 | 1 | 1 | 1 | 0 | 2 |
| bta-miR-421 | 163 | 204 | 155 | 190 | 113 | 84 | 65 | 111 |
| bta-miR-423-3p | 10714 | 11253 | 4630 | 6001 | 6133 | 4533 | 4789 | 4166 |
| bta-miR-423-5p | 7466 | 8258 | 3051 | 3285 | 2688 | 2105 | 2871 | 3395 |
| bta-miR-424-3p | 17 | 26 | 19 | 51 | 47 | 25 | 10 | 24 |
| bta-miR-424-5p | 123 | 157 | 176 | 279 | 309 | 187 | 24 | 127 |
| bta-miR-425-3p | 194 | 216 | 87 | 112 | 52 | 45 | 44 | 58 |
| bta-miR-425-5p | 1771 | 1990 | 1033 | 847 | 756 | 775 | 1167 | 976 |
| bta-miR-4286 | 29 | 37 | 19 | 47 | 53 | 27 | 1 | 15 |
| bta-miR-429 | 8512 | 8532 | 1736 | 2454 | 131 | 108 | 2779 | 1939 |
| bta-miR-431 | 0 | 0 | 3 | 9 | 6 | 9 | 2 | 2 |
| bta-miR-432 | 0 | 0 | 72 | 109 | 107 | 75 | 7 | 29 |
| bta-miR-433 | 0 | 0 | 65 | 121 | 107 | 67 | 7 | 39 |
| bta-miR-4449 | 4 | 3 | 0 | 2 | 1 | 0 | 0 | 0 |
| bta-miR-449a | 39 | 30 | 93 | 175 | 241 | 92 | 14 | 50 |
| bta-miR-449b | 2 | 1 | 0 | 2 | 4 | 0 | 0 | 1 |
| bta-miR-449c | 1 | 0 | 0 | 2 | 3 | 1 | 0 | 1 |
| bta-miR-449d | 0 | 1 | 1 | 2 | 1 | 0 | 1 | 2 |
| bta-miR-450a | 784 | 765 | 719 | 1241 | 1359 | 1015 | 440 | 724 |
| bta-miR-450b | 538 | 629 | 782 | 1473 | 2048 | 1102 | 342 | 713 |
| bta-miR-451 | 0 | 2 | 0 | 4 | 2 | 0 | 0 | 2 |
| bta-miR-452 | 12853 | 13667 | 2037 | 1851 | 478 | 494 | 1258 | 1203 |
| bta-miR-4523 | 1 | 2 | 2 | 0 | 2 | 0 | 0 | 1 |
| bta-miR-454 | 172 | 194 | 55 | 146 | 128 | 62 | 11 | 73 |
| bta-miR-455-3p | 1333 | 1435 | 691 | 936 | 993 | 582 | 114 | 634 |
| bta-miR-455-5p | 3194 | 2905 | 3611 | 3306 | 3845 | 5469 | 4149 | 3521 |
| bta-miR-4680 | 2 | 2 | 2 | 2 | 2 | 1 | 18 | 7 |
| bta-miR-484 | 1191 | 1280 | 282 | 687 | 649 | 311 | 110 | 260 |
| bta-miR-485 | 0 | 0 | 7 | 11 | 4 | 17 | 9 | 4 |
| bta-miR-486 | 168 | 158 | 68 | 83 | 62 | 68 | 15 | 24 |
| bta-miR-487a | 0 | 0 | 9 | 14 | 33 | 9 | 1 | 3 |
| bta-miR-487b | 4 | 2 | 43 | 96 | 70 | 37 | 4 | 26 |
| bta-miR-488 | 0 | 0 | 0 | 1 | 0 | 0 | 0 | 0 |
| bta-miR-490 | 1 | 1 | 11 | 5 | 12 | 7 | 3 | 4 |
| bta-miR-491 | 20 | 18 | 5 | 10 | 8 | 5 | 1 | 10 |
| bta-miR-493 | 0 | 0 | 641 | 602 | 564 | 872 | 325 | 189 |
| bta-miR-494 | 21 | 18 | 462 | 1223 | 1355 | 492 | 10 | 294 |
| bta-miR-495 | 8 | 9 | 179 | 292 | 284 | 249 | 138 | 218 |
| bta-miR-496 | 0 | 0 | 2 | 4 | 1 | 0 | 0 | 4 |
| bta-miR-497 | 3 | 5 | 7 | 20 | 15 | 9 | 2 | 3 |
| bta-miR-499 | 1122 | 1070 | 233 | 297 | 47 | 40 | 179 | 395 |
| bta-miR-500 | 268 | 320 | 168 | 278 | 407 | 218 | 49 | 178 |
| bta-miR-502a | 85 | 108 | 19 | 40 | 29 | 9 | 6 | 30 |
| bta-miR-502b | 26 | 29 | 26 | 36 | 39 | 47 | 16 | 19 |
| bta-miR-503-3p | 0 | 5 | 1 | 5 | 3 | 1 | 2 | 1 |
| bta-miR-503-5p | 193 | 177 | 346 | 561 | 243 | 142 | 53 | 323 |
| bta-miR-504 | 1116 | 1158 | 138 | 137 | 11 | 7 | 217 | 186 |
| bta-miR-505 | 104 | 142 | 64 | 91 | 102 | 49 | 12 | 46 |
| bta-miR-532 | 3010 | 2894 | 2848 | 4215 | 7244 | 5970 | 5506 | 2713 |
| bta-miR-539 | 0 | 0 | 0 | 1 | 1 | 0 | 0 | 1 |
| bta-miR-541 | 1 | 0 | 1 | 3 | 4 | 3 | 0 | 0 |
| bta-miR-542-5p | 3 | 2 | 0 | 1 | 5 | 5 | 3 | 3 |
| bta-miR-543 | 9 | 10 | 97 | 209 | 247 | 222 | 82 | 106 |
| bta-miR-545-3p | 1 | 0 | 5 | 4 | 11 | 2 | 1 | 0 |
| bta-miR-545-5p | 2 | 1 | 0 | 0 | 0 | 0 | 0 | 0 |
| bta-miR-574 | 5187 | 4624 | 968 | 1874 | 1343 | 1011 | 724 | 1273 |
| bta-miR-582 | 96 | 100 | 40 | 85 | 31 | 13 | 12 | 48 |
| bta-miR-592 | 1425 | 1371 | 82 | 159 | 60 | 22 | 60 | 107 |
| bta-miR-6119-3p | 124 | 111 | 57 | 130 | 93 | 53 | 11 | 59 |
| bta-miR-6119-5p | 1882 | 2095 | 1195 | 2037 | 1932 | 764 | 192 | 931 |
| bta-miR-6120-3p | 1583 | 1663 | 1162 | 876 | 577 | 683 | 2568 | 822 |
| bta-miR-6120-5p | 3 | 1 | 0 | 3 | 0 | 2 | 0 | 0 |
| bta-miR-6121-3p | 9 | 8 | 9 | 14 | 1 | 0 | 10 | 5 |
| bta-miR-6122-3p | 10 | 8 | 6 | 12 | 2 | 3 | 8 | 3 |
| bta-miR-6123 | 60 | 50 | 37 | 71 | 71 | 30 | 13 | 52 |
| bta-miR-615 | 1 | 1 | 0 | 2 | 0 | 0 | 0 | 0 |
| bta-miR-628 | 18 | 14 | 4 | 9 | 7 | 6 | 2 | 9 |
| bta-miR-6516 | 88 | 101 | 39 | 39 | 28 | 16 | 16 | 36 |
| bta-miR-6517 | 66 | 100 | 53 | 69 | 76 | 67 | 64 | 49 |
| bta-miR-6518 | 0 | 1 | 5 | 22 | 35 | 29 | 2 | 6 |
| bta-miR-652 | 773 | 759 | 230 | 326 | 79 | 45 | 181 | 248 |
| bta-miR-6520 | 25 | 23 | 6 | 13 | 8 | 8 | 15 | 3 |
| bta-miR-6521 | 1 | 1 | 0 | 0 | 0 | 0 | 0 | 0 |
| bta-miR-6522 | 35 | 29 | 1 | 4 | 2 | 1 | 4 | 4 |
| bta-miR-6523a | 18 | 15 | 17 | 4 | 12 | 8 | 16 | 8 |
| bta-miR-6524 | 80 | 74 | 19 | 44 | 33 | 13 | 2 | 21 |
| bta-miR-6525 | 11 | 8 | 4 | 4 | 4 | 9 | 1 | 0 |
| bta-miR-6526 | 1 | 0 | 10 | 9 | 14 | 10 | 7 | 6 |
| bta-miR-6527 | 3 | 1 | 2 | 0 | 0 | 1 | 2 | 0 |
| bta-miR-6529a | 755 | 746 | 352 | 477 | 299 | 225 | 756 | 447 |
| bta-miR-6529b | 754 | 746 | 351 | 477 | 298 | 225 | 752 | 445 |
| bta-miR-6531 | 3 | 0 | 0 | 1 | 1 | 0 | 4 | 1 |
| bta-miR-6532 | 0 | 0 | 1 | 1 | 1 | 0 | 1 | 1 |
| bta-miR-6533 | 3 | 4 | 2 | 2 | 0 | 1 | 0 | 1 |
| bta-miR-6534 | 2 | 1 | 0 | 0 | 1 | 0 | 6 | 1 |
| bta-miR-6535 | 5 | 9 | 1 | 2 | 1 | 1 | 0 | 2 |
| bta-miR-6536 | 7 | 3 | 3 | 6 | 7 | 2 | 6 | 6 |
| bta-miR-654 | 0 | 0 | 13 | 24 | 15 | 14 | 0 | 6 |
| bta-miR-655 | 1 | 1 | 22 | 46 | 62 | 18 | 0 | 19 |
| bta-miR-656 | 1 | 0 | 4 | 15 | 14 | 3 | 0 | 8 |
| bta-miR-660 | 5458 | 5284 | 3705 | 5484 | 5901 | 4573 | 1731 | 3473 |
| bta-miR-664a | 1 | 2 | 3 | 1 | 2 | 1 | 1 | 0 |
| bta-miR-664b | 252 | 250 | 45 | 62 | 55 | 46 | 17 | 49 |
| bta-miR-665 | 0 | 0 | 2 | 6 | 13 | 7 | 0 | 2 |
| bta-miR-669 | 2 | 2 | 0 | 0 | 0 | 0 | 0 | 0 |
| bta-miR-671 | 45 | 57 | 38 | 54 | 107 | 89 | 17 | 28 |
| bta-miR-677 | 50 | 62 | 35 | 36 | 26 | 36 | 436 | 44 |
| bta-miR-7 | 10516 | 11282 | 6939 | 11225 | 8524 | 5242 | 11122 | 8752 |
| bta-miR-708 | 1349 | 1242 | 501 | 643 | 417 | 284 | 88 | 331 |
| bta-miR-744 | 261 | 265 | 179 | 169 | 115 | 103 | 218 | 112 |
| bta-miR-758 | 1 | 0 | 15 | 37 | 37 | 35 | 3 | 5 |
| bta-miR-760-3p | 20 | 28 | 10 | 31 | 29 | 27 | 2 | 16 |
| bta-miR-760-5p | 1 | 1 | 1 | 1 | 2 | 2 | 0 | 1 |
| bta-miR-769 | 436 | 420 | 165 | 276 | 330 | 232 | 361 | 177 |
| bta-miR-7691 | 0 | 0 | 1 | 0 | 0 | 4 | 0 | 0 |
| bta-miR-7857 | 432 | 433 | 1105 | 949 | 582 | 622 | 839 | 981 |
| bta-miR-7858 | 12 | 9 | 2 | 3 | 1 | 0 | 2 | 3 |
| bta-miR-7859 | 188 | 217 | 75 | 102 | 59 | 43 | 24 | 73 |
| bta-miR-7860 | 7 | 9 | 9 | 5 | 10 | 7 | 13 | 5 |
| bta-miR-7861 | 5 | 9 | 1 | 2 | 0 | 2 | 3 | 2 |
| bta-miR-7862 | 35 | 37 | 15 | 9 | 2 | 3 | 28 | 6 |
| bta-miR-7863 | 2 | 4 | 6 | 8 | 2 | 3 | 17 | 9 |
| bta-miR-7864 | 0 | 0 | 0 | 0 | 1 | 0 | 0 | 0 |
| bta-miR-7865 | 0 | 0 | 1 | 0 | 0 | 0 | 0 | 0 |
| bta-miR-873 | 1 | 0 | 0 | 1 | 0 | 0 | 0 | 0 |
| bta-miR-874 | 13 | 22 | 43 | 59 | 40 | 48 | 24 | 42 |
| bta-miR-877 | 65 | 81 | 99 | 47 | 45 | 78 | 62 | 68 |
| bta-miR-885 | 39 | 28 | 4 | 8 | 0 | 1 | 2 | 7 |
| bta-miR-92a | 20003 | 22709 | 4062 | 7176 | 5668 | 2810 | 1610 | 3958 |
| bta-miR-92b | 1282 | 1310 | 281 | 393 | 99 | 74 | 264 | 438 |
| bta-miR-93 | 12317 | 12225 | 3523 | 6098 | 4929 | 2532 | 412 | 2399 |
| bta-miR-935 | 3 | 5 | 0 | 0 | 1 | 0 | 0 | 0 |
| bta-miR-9-3p | 271 | 273 | 177 | 466 | 501 | 230 | 39 | 182 |
| bta-miR-95 | 800 | 797 | 71 | 82 | 10 | 7 | 114 | 53 |
| bta-miR-9-5p | 13145 | 13642 | 8783 | 8678 | 7701 | 8292 | 6617 | 6538 |
| bta-miR-96 | 3602 | 3863 | 670 | 994 | 124 | 63 | 687 | 784 |
| bta-miR-98 | 3112 | 2937 | 1161 | 2714 | 2151 | 1347 | 284 | 1180 |
| bta-miR-99a-3p | 1 | 0 | 0 | 1 | 3 | 0 | 0 | 0 |
| bta-miR-99a-5p | 1514 | 1406 | 756 | 981 | 1760 | 1200 | 222 | 729 |
| bta-miR-99b | 24603 | 20691 | 22751 | 22499 | 16796 | 25145 | 48341 | 20269 |
