# Supplementary material for: Specific microRNA library of IFN-τ on bovine endometrial epithelial cells
Source: Oncotarget. 2017 Jun 14;8(37):61487–98. doi: 10.18632/oncotarget.18470 (PMC5617439; doi:10.18632/oncotarget.18470)
Supplement: Supplementary file 4 [file oncotarget-08-61487-s004.doc]

**Supplementary Table 4: Novel mature miRNA sequence and read counts**

| **Name** | **Sequence** | **CSa** | **CSb** | **TSa** | **TSb** | **CTa** | **CTb** | **TTa** | **TTb** |
| --- | --- | --- | --- | --- | --- | --- | --- | --- | --- |
| novel_1 | gugggacgcgugcguuuu | 933 | 801 | 191 | 551 | 1011 | 363 | 42 | 212 |
| novel_100 | uguuugggucccgagggcugau | 2 | 2 | 0 | 0 | 0 | 0 | 0 | 3 |
| novel_101 | uacugugcugcagcuggguaga | 0 | 1 | 1 | 2 | 2 | 1 | 0 | 1 |
| novel_103 | aacugagcgacucacucccaaa | 0 | 0 | 3 | 1 | 0 | 1 | 0 | 2 |
| novel_104 | ucagaucuggaauccugggacu | 0 | 0 | 0 | 0 | 0 | 1 | 5 | 1 |
| novel_105 | uauccaggacuaggucugcaga | 2 | 2 | 1 | 0 | 0 | 0 | 0 | 1 |
| novel_106 | cuggagcugucacacacug | 0 | 0 | 1 | 3 | 1 | 2 | 2 | 1 |
| novel_107 | cugacucugagcccggcagugu | 0 | 0 | 0 | 2 | 0 | 3 | 0 | 3 |
| novel_108 | aaucaaugaacuuuuuggcca | 0 | 0 | 3 | 1 | 1 | 0 | 0 | 0 |
| novel_109 | uccuguccgaucagcucuagac | 1 | 1 | 1 | 0 | 0 | 0 | 1 | 0 |
| novel_11 | aaauuuagcagagcucucugaa | 5 | 8 | 0 | 2 | 0 | 1 | 1 | 1 |
| novel_110 | ugacugagcccaccaggcu | 2 | 2 | 1 | 0 | 0 | 0 | 0 | 0 |
| novel_111 | cgggugggaagaggcggg | 0 | 1 | 2 | 1 | 0 | 0 | 0 | 0 |
| novel_112 | gccuggcagcuuccggga | 0 | 0 | 3 | 0 | 0 | 1 | 4 | 2 |
| novel_113 | ucaacgaacucuuuggccaaug | 0 | 1 | 0 | 0 | 0 | 0 | 5 | 0 |
| novel_114 | uaccccguguggcuugugggau | 3 | 0 | 0 | 1 | 0 | 0 | 0 | 0 |
| novel_115 | uuucugggaguuguaguaucuagg | 1 | 1 | 1 | 0 | 0 | 1 | 3 | 0 |
| novel_116 | gugugcucggccugugacg | 1 | 1 | 0 | 0 | 0 | 0 | 0 | 0 |
| novel_117 | augugugcaugugggccaggu | 2 | 2 | 0 | 0 | 0 | 0 | 0 | 0 |
| novel_12 | uaggacuacaacucccaucgug | 1 | 4 | 2 | 0 | 1 | 1 | 11 | 2 |
| novel_13 | aucccagccgggucgagggaca | 1 | 0 | 2 | 2 | 0 | 5 | 0 | 2 |
| novel_14 | ccacgugcccuaaagacugugc | 4 | 2 | 0 | 3 | 1 | 0 | 0 | 3 |
| novel_15 | guggcugccggaccgaccggac | 0 | 0 | 0 | 0 | 0 | 0 | 3 | 0 |
| novel_16 | cgcaaaucaguggaccugguu | 0 | 3 | 0 | 0 | 0 | 0 | 0 | 0 |
| novel_17 | aucgacacaaggguuugu | 1 | 1 | 0 | 2 | 0 | 1 | 2 | 4 |
| novel_18 | gcggcccgcgggcucggaugcu | 2 | 1 | 0 | 1 | 0 | 0 | 2 | 0 |
| novel_19 | aaauacccagacgaaccuuuug | 0 | 0 | 0 | 0 | 1 | 2 | 0 | 0 |
| novel_2 | ccugacggugcuuucaauucuga | 13 | 15 | 27 | 15 | 7 | 22 | 248 | 36 |
| novel_20 | uggccuauuaagaacuagagc | 3 | 3 | 0 | 3 | 0 | 0 | 2 | 3 |
| novel_21 | caaaaaggucaucuggguuuuu | 1 | 1 | 2 | 0 | 2 | 0 | 0 | 1 |
| novel_22 | aaaaaaguuuguuuggauuuu | 1 | 1 | 0 | 3 | 1 | 0 | 0 | 0 |
| novel_23 | uugguuagaaauguagcacauucc | 1 | 1 | 0 | 0 | 1 | 0 | 0 | 1 |
| novel_24 | uugucuagacucuggauuagau | 0 | 0 | 0 | 1 | 1 | 2 | 0 | 1 |
| novel_26 | agaaagccugaaugcaucugaga | 2 | 1 | 0 | 0 | 1 | 0 | 0 | 0 |
| novel_27 | uggcccacuccuuccucaacagg | 1 | 0 | 0 | 0 | 0 | 0 | 0 | 0 |
| novel_28 | uugacuuggccuagcucuuggga | 0 | 2 | 0 | 0 | 0 | 0 | 0 | 0 |
| novel_29 | ccgagcuguggucucucucu | 1 | 1 | 0 | 0 | 0 | 0 | 0 | 0 |
| novel_3 | aacuguuaggaggcuuggcugcu | 17 | 22 | 23 | 57 | 2 | 0 | 53 | 81 |
| novel_30 | ugaccuuagagcgagcugccca | 1 | 0 | 0 | 0 | 0 | 0 | 2 | 0 |
| novel_31 | ucaauaugcucuuggauuugu | 0 | 1 | 0 | 0 | 0 | 1 | 0 | 0 |
| novel_32 | gaaaagcccuuaacuucccugu | 0 | 0 | 0 | 1 | 0 | 0 | 0 | 0 |
| novel_33 | ccgaaugaaccuuuuggccaagc | 1 | 1 | 0 | 0 | 0 | 0 | 0 | 0 |
| novel_34 | ccucaagcugcucuaaaucuaau | 0 | 1 | 0 | 0 | 0 | 0 | 0 | 0 |
| novel_35 | aggucugcaguuugacuacaaca | 0 | 0 | 0 | 1 | 0 | 0 | 0 | 1 |
| novel_36 | ggcccgcggcggcgggcgcgucggu | 259 | 237 | 56 | 72 | 20 | 26 | 94 | 93 |
| novel_37 | uucugccccucggugugcgacg | 5 | 1 | 3 | 8 | 0 | 0 | 4 | 1 |
| novel_38 | uggcaccagcacuggcggugg | 1 | 1 | 2 | 2 | 2 | 2 | 0 | 2 |
| novel_39 | cggcggcggcgccggggcg | 1 | 1 | 1 | 2 | 2 | 1 | 1 | 0 |
| novel_4 | uuuguucuccaaccuggcucuuua | 5 | 0 | 11 | 15 | 20 | 17 | 5 | 17 |
| novel_40 | agacacgacucagcgacucaguu | 5 | 5 | 3 | 2 | 0 | 0 | 14 | 3 |
| novel_41 | agccuuaguucuucggggucuga | 2 | 5 | 3 | 2 | 1 | 0 | 4 | 2 |
| novel_42 | uaggcucuagagugugugggcu | 2 | 2 | 0 | 4 | 1 | 2 | 0 | 1 |
| novel_43 | acaccaggacuugucuccccaga | 3 | 4 | 2 | 0 | 0 | 0 | 0 | 3 |
| novel_44 | uuguccuacuucucagcugucu | 15 | 17 | 7 | 16 | 8 | 3 | 5 | 7 |
| novel_45 | uggauuauagccuuuuucaua | 7 | 18 | 2 | 7 | 6 | 0 | 2 | 1 |
| novel_46 | ccgauacuuauagagauagau | 4 | 6 | 0 | 0 | 0 | 0 | 0 | 0 |
| novel_47 | uacugugccucgaauggguaug | 0 | 0 | 3 | 5 | 4 | 7 | 0 | 0 |
| novel_48 | uuauuuuugacgucccagaac | 0 | 2 | 1 | 1 | 2 | 3 | 0 | 1 |
| novel_49 | uggagaucuaggcucuggacg | 1 | 1 | 0 | 2 | 0 | 0 | 7 | 0 |
| novel_5 | gaaaguuuguugggguuuuucu | 0 | 1 | 5 | 15 | 13 | 2 | 1 | 4 |
| novel_50 | uaucuacucuagauaagacuuu | 6 | 3 | 11 | 2 | 8 | 2 | 0 | 3 |
| novel_51 | aaacgugaaugaacuuuuugg | 0 | 0 | 0 | 0 | 2 | 6 | 0 | 4 |
| novel_52 | cggaaugcucuguacaaacaau | 3 | 2 | 1 | 8 | 2 | 2 | 3 | 3 |
| novel_53 | aggaacccagaugaacuuucu | 1 | 0 | 2 | 9 | 20 | 6 | 2 | 3 |
| novel_54 | ucugaacgaacuuuguggcaa | 2 | 1 | 1 | 0 | 0 | 1 | 2 | 0 |
| novel_55 | aggaaggggcuucugagcuucu | 6 | 0 | 1 | 7 | 4 | 4 | 3 | 5 |
| novel_56 | aaacccugaaggaacauuuugg | 2 | 3 | 1 | 0 | 1 | 0 | 0 | 1 |
| novel_58 | ucagaccccgaagaacuaaggcu | 2 | 5 | 3 | 2 | 1 | 0 | 4 | 2 |
| novel_6 | accuguuaaagacuuuaccacu | 7 | 10 | 0 | 1 | 2 | 0 | 0 | 1 |
| novel_60 | gucaguaggaucaaaagacu | 2 | 3 | 1 | 5 | 0 | 2 | 0 | 1 |
| novel_61 | uccccagaagugccccugca | 0 | 0 | 0 | 2 | 4 | 0 | 0 | 0 |
| novel_62 | cucaaaucaguggaccugguag | 7 | 10 | 4 | 3 | 1 | 2 | 23 | 3 |
| novel_64 | ucauuugagcucuucccgaagug | 1 | 3 | 0 | 1 | 0 | 0 | 1 | 3 |
| novel_65 | uugauauguccugagacgcgga | 9 | 9 | 3 | 3 | 0 | 2 | 0 | 3 |
| novel_66 | aaccgcgaguggcugaaccuug | 6 | 8 | 15 | 6 | 8 | 2 | 11 | 7 |
| novel_67 | cuuccacuguggaguuccuggg | 4 | 2 | 12 | 11 | 6 | 3 | 9 | 8 |
| novel_68 | cugucccucucugcccuu | 0 | 2 | 0 | 1 | 0 | 0 | 0 | 1 |
| novel_69 | uugugguuauguuuguuu | 208 | 191 | 225 | 688 | 670 | 184 | 10 | 188 |
| novel_7 | ugaaaggaccugccuggguaga | 1 | 0 | 8 | 10 | 9 | 9 | 1 | 3 |
| novel_70 | ucuggagcuucuguuuucu | 15 | 9 | 5 | 6 | 1 | 1 | 15 | 4 |
| novel_71 | augacacugaacaauuacugca | 5 | 4 | 4 | 3 | 4 | 1 | 4 | 4 |
| novel_72 | uuucucucgugcucuguguag | 2 | 3 | 0 | 1 | 1 | 1 | 2 | 3 |
| novel_74 | accuuuguucggguuuuucugu | 6 | 3 | 2 | 5 | 1 | 2 | 0 | 1 |
| novel_75 | uugcacgcuacugaggccucugu | 2 | 3 | 0 | 1 | 0 | 1 | 0 | 1 |
| novel_76 | uuggugcuucugugggagucauu | 0 | 2 | 1 | 5 | 1 | 2 | 4 | 3 |
| novel_77 | ucugccuccuuuccucccagu | 0 | 2 | 1 | 0 | 1 | 1 | 2 | 1 |
| novel_78 | gagcucaucagacugaugu | 0 | 0 | 1 | 1 | 0 | 1 | 1 | 5 |
| novel_79 | ucaccgugcccugguccuccaga | 3 | 2 | 1 | 0 | 1 | 1 | 0 | 0 |
| novel_8 | cggguagggcugugacccucg | 1 | 3 | 3 | 2 | 2 | 4 | 18 | 1 |
| novel_80 | aagaggugcucaggagugaaga | 1 | 0 | 0 | 1 | 1 | 4 | 0 | 1 |
| novel_81 | uauauuguccaaacuggggua | 4 | 3 | 0 | 2 | 2 | 0 | 0 | 0 |
| novel_82 | uccagggggucaggucugugugu | 0 | 1 | 0 | 0 | 0 | 0 | 0 | 2 |
| novel_83 | aggggacccugcggagcuccgcu | 1 | 3 | 0 | 1 | 0 | 0 | 2 | 0 |
| novel_84 | gaaugugugaccucgaag | 1 | 1 | 0 | 0 | 1 | 0 | 2 | 1 |
| novel_85 | aagcggggucucagccgcugggcc | 0 | 2 | 1 | 1 | 0 | 0 | 0 | 0 |
| novel_87 | aaaaaccuaagugaacucuuug | 0 | 0 | 2 | 1 | 2 | 1 | 1 | 2 |
| novel_88 | caauggauuuaaggaggcucca | 6 | 7 | 0 | 2 | 0 | 0 | 2 | 1 |
| novel_89 | cugccucuccgccaccuccacc | 0 | 1 | 0 | 3 | 1 | 1 | 1 | 1 |
| novel_9 | cuuuaacucuucauccuuuug | 4 | 7 | 1 | 1 | 1 | 0 | 1 | 3 |
| novel_90 | gggucuccucuccccucuuu | 0 | 0 | 1 | 1 | 6 | 2 | 0 | 0 |
| novel_91 | uacugugcuuugaauggguagu | 1 | 1 | 7 | 16 | 24 | 10 | 21 | 8 |
| novel_92 | agguuccaggcguuuggcugagu | 0 | 1 | 2 | 1 | 1 | 1 | 2 | 0 |
| novel_93 | cagcaguacaugcagagca | 6 | 1 | 0 | 8 | 15 | 1 | 0 | 3 |
| novel_94 | ccacugcgugacuaacacuuu | 6 | 7 | 0 | 2 | 0 | 0 | 1 | 0 |
| novel_95 | gaaaguucguucggguuuuu | 1 | 3 | 1 | 2 | 2 | 0 | 0 | 1 |
| novel_96 | accgccuuuguguugcccauucacu | 4 | 4 | 1 | 7 | 2 | 3 | 2 | 3 |
| novel_97 | caccccagguuggaaagagcug | 0 | 1 | 1 | 0 | 0 | 2 | 0 | 0 |
| novel_98 | uucggcgccaccacccugcgggu | 0 | 3 | 0 | 0 | 1 | 0 | 1 | 1 |
| novel_99 | caaguaugagacagagcugg | 1 | 3 | 0 | 1 | 0 | 0 | 2 | 1 |
