# Supplementary material for: Specific microRNA library of IFN-τ on bovine endometrial epithelial cells
Source: Oncotarget. 2017 Jun 14;8(37):61487–98. doi: 10.18632/oncotarget.18470 (PMC5617439; doi:10.18632/oncotarget.18470)
Supplement: Supplementary file 5 [file oncotarget-08-61487-s005.doc]

**Supplementary Table 5: Differentially expressed miRNA in each group**

**TS vs. CS group**

| **sRNA** | **TS_readcount** | **CS_readcount** | **log2FoldChange** | ***p*-Value** |
| --- | --- | --- | --- | --- |
| bta-let-7i | 203471.015 | 383907.4744 | -0.82256 | 0.028287 |
| bta-miR-1 | 796.7544296 | 2587.974115 | -1.5156 | 8.70E-05 |
| bta-miR-10a | 43759.37203 | 6780.985128 | 2.3292 | 1.78E-08 |
| bta-miR-122 | 51.77180115 | 14.78842351 | 1.257 | 0.023902 |
| bta-miR-1246 | 35.09689144 | 8.873054108 | 1.2572 | 0.03115 |
| bta-miR-124a | 10.33807392 | 0 | 1.252 | 0.037032 |
| bta-miR-133a | 31.24926401 | 138.0252861 | -1.6984 | 0.00070456 |
| bta-miR-135a | 35.17832299 | 1.971789802 | 2.1377 | 0.00036966 |
| bta-miR-138 | 12.66904537 | 0.985894901 | 1.2459 | 0.042426 |
| bta-miR-141 | 1148.868012 | 158.729079 | 2.5141 | 1.65E-10 |
| bta-miR-145 | 2442.775386 | 8890.800216 | -1.6628 | 1.65E-05 |
| bta-miR-147 | 80.03401514 | 19.71789802 | 1.4927 | 0.0048437 |
| bta-miR-152 | 5610.031209 | 10506.68196 | -0.80875 | 0.034426 |
| bta-miR-182 | 7047.926538 | 856.7426689 | 2.6909 | 3.48E-12 |
| bta-miR-183 | 2354.172502 | 251.4031997 | 2.8148 | 3.03E-12 |
| bta-miR-1839 | 2502.333968 | 1183.073881 | 0.94123 | 0.021978 |
| bta-miR-184 | 273099.2336 | 15531.78827 | 3.6567 | 2.49E-21 |
| bta-miR-193b | 947.035902 | 1899.819474 | -0.89557 | 0.020027 |
| bta-miR-196b | 1087.856543 | 2066.435712 | -0.82369 | 0.03358 |
| bta-miR-199a-5p | 17711.18249 | 33956.19218 | -0.82018 | 0.046682 |
| bta-miR-200a | 87962.0942 | 9238.821116 | 2.8976 | 1.64E-14 |
| bta-miR-200b | 41864.94448 | 2961.628282 | 3.3669 | 6.57E-18 |
| bta-miR-200c | 10874.05957 | 968.1487927 | 3.0626 | 1.04E-14 |
| bta-miR-204 | 1068.546226 | 2557.411373 | -1.1129 | 0.0050848 |
| bta-miR-205 | 1303.802227 | 18.73200312 | 4.7274 | 8.98E-23 |
| bta-miR-214 | 675.0932808 | 1422.646342 | -0.95769 | 0.013327 |
| bta-miR-221 | 37777.64671 | 71967.37008 | -0.82568 | 0.034699 |
| bta-miR-222 | 14032.00575 | 34523.08175 | -1.1574 | 0.0027683 |
| bta-miR-224 | 3200.713884 | 774.9133921 | 1.8241 | 1.57E-06 |
| bta-miR-2285p | 57.60154287 | 4.929474505 | 2.2791 | 5.77E-05 |
| bta-miR-23b-5p | 53.66785769 | 130.1381269 | -1.0349 | 0.032305 |
| bta-miR-2411-5p | 69.61450967 | 13.80252861 | 1.6515 | 0.0025157 |
| bta-miR-2416 | 15.75603171 | 1.971789802 | 1.2471 | 0.043301 |
| bta-miR-2474 | 18.68015497 | 1.971789802 | 1.2667 | 0.039968 |
| bta-miR-2957 | 5610.031209 | 10506.68196 | -0.80875 | 0.034426 |
| bta-miR-296-3p | 112.6093149 | 439.7091258 | -1.7085 | 4.88E-05 |
| bta-miR-29b | 1201.300518 | 546.1857751 | 1.0104 | 0.0088585 |
| bta-miR-29c | 152.982592 | 45.35116544 | 1.3878 | 0.005159 |
| bta-miR-29d-5p | 52.68142674 | 15.77431841 | 1.2513 | 0.02149 |
| bta-miR-30a-5p | 34556.8001 | 5141.441908 | 2.4564 | 5.74E-11 |
| bta-miR-30f | 1009.490008 | 116.3355983 | 2.7318 | 6.73E-12 |
| bta-miR-31 | 2124.411369 | 656.606004 | 1.4977 | 0.00013072 |
| bta-miR-3431 | 5664.798366 | 1676.021332 | 1.5671 | 3.68E-05 |
| bta-miR-34c | 196.4770789 | 419.9912278 | -0.95168 | 0.024258 |
| bta-miR-362-5p | 364.8714716 | 689.1405357 | -0.80825 | 0.043425 |
| bta-miR-365-5p | 336.4251035 | 725.6186471 | -0.97662 | 0.015557 |
| bta-miR-370 | 82.85820206 | 174.5033975 | -0.9039 | 0.04829 |
| bta-miR-375 | 1036.273469 | 95.63180539 | 2.8802 | 7.50E-11 |
| bta-miR-378 | 19126.9083 | 7717.585284 | 1.1454 | 0.0047265 |
| bta-miR-378b | 4792.135016 | 1295.4659 | 1.6465 | 5.23E-05 |
| bta-miR-378c | 2872.657673 | 866.6016179 | 1.5386 | 5.81E-05 |
| bta-miR-429 | 2035.51982 | 129.152232 | 3.5015 | 3.06E-19 |
| bta-miR-450b | 1069.981451 | 2019.112757 | -0.81112 | 0.039649 |
| bta-miR-452 | 1960.178239 | 471.2577626 | 1.7913 | 1.14E-05 |
| bta-miR-499 | 259.7223694 | 46.33706034 | 2.0957 | 1.91E-06 |
| bta-miR-504 | 137.6263056 | 10.84484391 | 2.7633 | 5.18E-08 |
| bta-miR-532 | 3418.114192 | 7141.822662 | -0.95362 | 0.011402 |
| bta-miR-652 | 270.0465647 | 77.88569717 | 1.5346 | 0.00034151 |
| bta-miR-671 | 44.67432427 | 105.4907544 | -1.0016 | 0.04067 |
| bta-miR-92b | 327.7249129 | 97.60359519 | 1.5077 | 0.00032807 |
| bta-miR-95 | 75.60248829 | 9.858949009 | 2.1164 | 7.57E-05 |
| bta-miR-96 | 805.1242678 | 122.2509677 | 2.3809 | 2.74E-09 |
| bta-miR-99a-5p | 849.9262856 | 1735.175026 | -0.91904 | 0.016784 |
| novel_1 | 340.9667653 | 996.7397448 | -1.3138 | 0.003091 |
| novel_3 | 37.16506347 | 1.971789802 | 2.1868 | 0.00026587 |
| novel_36 | 62.67989589 | 19.71789802 | 1.2498 | 0.017539 |

**TT vs. CT group**

| sRNA | TS_readcount | CS_readcount | log2FoldChange | *p*-value |
| --- | --- | --- | --- | --- |
| bta-miR-10a | 111965.3334 | 6506.64583 | 2.4454 | 0.0019578 |
| bta-miR-135a | 42.37728959 | 0.887310218 | 2.4674 | 0.0025612 |
| bta-miR-141 | 935.8811186 | 118.8995692 | 1.9838 | 0.0094133 |
| bta-miR-146b | 217.5997659 | 1057.67378 | -1.6286 | 0.032361 |
| bta-miR-147 | 455.3043912 | 45.25282113 | 1.7644 | 0.031004 |
| bta-miR-182 | 9312.36412 | 519.9637879 | 2.6863 | 0.00044819 |
| bta-miR-183 | 7219.726858 | 174.800113 | 2.9004 | 0.00027854 |
| bta-miR-184 | 1208678.993 | 14733.78617 | 3.0745 | 0.00013504 |
| bta-miR-196b | 149.8371559 | 976.0412401 | -1.7021 | 0.035762 |
| bta-miR-199a-5p | 3147.818959 | 19111.77479 | -1.7595 | 0.025513 |
| bta-miR-200a | 89337.4459 | 5510.196455 | 2.6637 | 0.00043307 |
| bta-miR-200b | 31547.21757 | 2602.48087 | 2.4057 | 0.001494 |
| bta-miR-200c | 10783.38635 | 755.9883059 | 2.5338 | 0.00085357 |
| bta-miR-204 | 376.4863665 | 1740.902648 | -1.5687 | 0.039986 |
| bta-miR-205 | 199.7309852 | 10.64772262 | 2.5399 | 0.0012153 |
| bta-miR-211 | 16.25375206 | 0 | 1.9261 | 0.020539 |
| bta-miR-224 | 3398.01625 | 671.6938352 | 1.5553 | 0.043436 |
| bta-miR-2285e | 149.2007398 | 22.18275546 | 1.6648 | 0.036588 |
| bta-miR-2285p | 105.6686388 | 2.661930655 | 2.579 | 0.0014986 |
| bta-miR-2474 | 61.63269768 | 5.323861309 | 1.7142 | 0.037824 |
| bta-miR-2484 | 233.9099887 | 28.39392698 | 1.6171 | 0.048386 |
| bta-miR-29d-5p | 138.6808367 | 14.19696349 | 2.0145 | 0.010624 |
| bta-miR-30a-5p | 90979.51408 | 3867.785241 | 2.7784 | 0.00036254 |
| bta-miR-30f | 800.9179168 | 57.67516419 | 2.4353 | 0.0015757 |
| bta-miR-3431 | 4161.402774 | 908.6056635 | 1.5028 | 0.047687 |
| bta-miR-375 | 2135.215807 | 67.43557659 | 2.8713 | 0.00027278 |
| bta-miR-411a | 559.4837052 | 2693.873823 | -1.5701 | 0.043196 |
| bta-miR-429 | 2623.446815 | 95.82950357 | 2.953 | 0.00013244 |
| bta-miR-499 | 282.4274348 | 35.49240873 | 1.9547 | 0.011265 |
| bta-miR-504 | 219.3824567 | 6.211171528 | 2.978 | 0.00014484 |
| bta-miR-652 | 221.9880494 | 39.92895982 | 1.638 | 0.033523 |
| bta-miR-677 | 302.3038928 | 31.94316786 | 1.6484 | 0.045259 |
| bta-miR-758 | 4.05295913 | 31.05585764 | -1.6344 | 0.048523 |
| bta-miR-92b | 355.8203854 | 65.66095615 | 1.6358 | 0.032624 |
| bta-miR-95 | 96.47120925 | 6.211171528 | 2.1782 | 0.0068856 |
| bta-miR-96 | 776.5043795 | 55.90054375 | 2.4781 | 0.0011864 |
| novel_2 | 176.5612125 | 19.5208248 | 1.6637 | 0.042529 |
| novel_3 | 68.52221172 | 0 | 3.1514 | 0.00010144 |
