# Supplementary material for: Specific microRNA library of IFN-τ on bovine endometrial epithelial cells
Source: Oncotarget. 2017 Jun 14;8(37):61487–98. doi: 10.18632/oncotarget.18470 (PMC5617439; doi:10.18632/oncotarget.18470)
Supplement: Supplementary file 6 [file oncotarget-08-61487-s006.doc]

**Supplementary Table 7: Target gene prediction of differentially expressed miRNAs**

| **TS** vs. CS group | **TT** vs. CT group |  |  |
| --- | --- | --- | --- |
| **Gene _ ID** | **Gene _ ID** |  |  |
| ENSBTAG00000000005 | ENSBTAG00000000005 |  |  |
| ENSBTAG00000000009 | ENSBTAG00000000009 |  |  |
| ENSBTAG00000000012 | ENSBTAG00000000011 |  |  |
| ENSBTAG00000000013 | ENSBTAG00000000012 |  |  |
| ENSBTAG00000000014 | ENSBTAG00000000013 |  |  |
| ENSBTAG00000000016 | ENSBTAG00000000016 |  |  |
| ENSBTAG00000000025 | ENSBTAG00000000025 |  |  |
| ENSBTAG00000000026 | ENSBTAG00000000026 |  |  |
| ENSBTAG00000000029 | ENSBTAG00000000031 |  |  |
| ENSBTAG00000000030 | ENSBTAG00000000032 |  |  |
| ENSBTAG00000000031 | ENSBTAG00000000033 |  |  |
| ENSBTAG00000000032 | ENSBTAG00000000040 |  |  |
| ENSBTAG00000000033 | ENSBTAG00000000042 |  |  |
| ENSBTAG00000000039 | ENSBTAG00000000044 |  |  |
| ENSBTAG00000000040 | ENSBTAG00000000046 |  |  |
| ENSBTAG00000000042 | ENSBTAG00000000049 |  |  |
| ENSBTAG00000000044 | ENSBTAG00000000050 |  |  |
| ENSBTAG00000000046 | ENSBTAG00000000053 |  |  |
| ENSBTAG00000000049 | ENSBTAG00000000056 |  |  |
| ENSBTAG00000000050 | ENSBTAG00000000064 |  |  |
| ENSBTAG00000000053 | ENSBTAG00000000065 |  |  |
| ENSBTAG00000000054 | ENSBTAG00000000070 |  |  |
| ENSBTAG00000000056 | ENSBTAG00000000071 |  |  |
| ENSBTAG00000000064 | ENSBTAG00000000072 |  |  |
| ENSBTAG00000000065 | ENSBTAG00000000073 |  |  |
| ENSBTAG00000000067 | ENSBTAG00000000074 |  |  |
| ENSBTAG00000000070 | ENSBTAG00000000079 |  |  |
| ENSBTAG00000000071 | ENSBTAG00000000087 |  |  |
| ENSBTAG00000000072 | ENSBTAG00000000090 |  |  |
| ENSBTAG00000000073 | ENSBTAG00000000095 |  |  |
| ENSBTAG00000000074 | ENSBTAG00000000097 |  |  |
| ENSBTAG00000000078 | ENSBTAG00000000099 |  |  |
| ENSBTAG00000000079 | ENSBTAG00000000105 |  |  |
| ENSBTAG00000000080 | ENSBTAG00000000108 |  |  |
| ENSBTAG00000000081 | ENSBTAG00000000111 |  |  |
| ENSBTAG00000000082 | ENSBTAG00000000113 |  |  |
| ENSBTAG00000000085 | ENSBTAG00000000115 |  |  |
| ENSBTAG00000000087 | ENSBTAG00000000130 |  |  |
| ENSBTAG00000000090 | ENSBTAG00000000132 |  |  |
| ENSBTAG00000000092 | ENSBTAG00000000133 |  |  |
| ENSBTAG00000000095 | ENSBTAG00000000134 |  |  |
| ENSBTAG00000000097 | ENSBTAG00000000139 |  |  |
| ENSBTAG00000000099 | ENSBTAG00000000146 |  |  |
| ENSBTAG00000000105 | ENSBTAG00000000152 |  |  |
| ENSBTAG00000000108 | ENSBTAG00000000157 |  |  |
| ENSBTAG00000000111 | ENSBTAG00000000160 |  |  |
| ENSBTAG00000000113 | ENSBTAG00000000161 |  |  |
| ENSBTAG00000000115 | ENSBTAG00000000163 |  |  |
| ENSBTAG00000000123 | ENSBTAG00000000169 |  |  |
| ENSBTAG00000000125 | ENSBTAG00000000179 |  |  |
| ENSBTAG00000000130 | ENSBTAG00000000181 |  |  |
| ENSBTAG00000000132 | ENSBTAG00000000183 |  |  |
| ENSBTAG00000000133 | ENSBTAG00000000184 |  |  |
| ENSBTAG00000000134 | ENSBTAG00000000185 |  |  |
| ENSBTAG00000000139 | ENSBTAG00000000188 |  |  |
| ENSBTAG00000000140 | ENSBTAG00000000191 |  |  |
| ENSBTAG00000000146 | ENSBTAG00000000197 |  |  |
| ENSBTAG00000000149 | ENSBTAG00000000201 |  |  |
| ENSBTAG00000000152 | ENSBTAG00000000202 |  |  |
| ENSBTAG00000000157 | ENSBTAG00000000205 |  |  |
| ENSBTAG00000000160 | ENSBTAG00000000207 |  |  |
| ENSBTAG00000000161 | ENSBTAG00000000215 |  |  |
| ENSBTAG00000000162 | ENSBTAG00000000225 |  |  |
| ENSBTAG00000000163 | ENSBTAG00000000226 |  |  |
| ENSBTAG00000000169 | ENSBTAG00000000231 |  |  |
| ENSBTAG00000000172 | ENSBTAG00000000240 |  |  |
| ENSBTAG00000000177 | ENSBTAG00000000244 |  |  |
| ENSBTAG00000000179 | ENSBTAG00000000250 |  |  |
| ENSBTAG00000000183 | ENSBTAG00000000252 |  |  |
| ENSBTAG00000000184 | ENSBTAG00000000257 |  |  |
| ENSBTAG00000000185 | ENSBTAG00000000260 |  |  |
| ENSBTAG00000000188 | ENSBTAG00000000264 |  |  |
| ENSBTAG00000000191 | ENSBTAG00000000265 |  |  |
| ENSBTAG00000000197 | ENSBTAG00000000266 |  |  |
| ENSBTAG00000000202 | ENSBTAG00000000267 |  |  |
| ENSBTAG00000000203 | ENSBTAG00000000271 |  |  |
| ENSBTAG00000000204 | ENSBTAG00000000274 |  |  |
| ENSBTAG00000000205 | ENSBTAG00000000275 |  |  |
| ENSBTAG00000000207 | ENSBTAG00000000279 |  |  |
| ENSBTAG00000000212 | ENSBTAG00000000286 |  |  |
| ENSBTAG00000000215 | ENSBTAG00000000287 |  |  |
| ENSBTAG00000000224 | ENSBTAG00000000289 |  |  |
| ENSBTAG00000000225 | ENSBTAG00000000290 |  |  |
| ENSBTAG00000000226 | ENSBTAG00000000291 |  |  |
| ENSBTAG00000000231 | ENSBTAG00000000292 |  |  |
| ENSBTAG00000000240 | ENSBTAG00000000295 |  |  |
| ENSBTAG00000000243 | ENSBTAG00000000297 |  |  |
| ENSBTAG00000000244 | ENSBTAG00000000301 |  |  |
| ENSBTAG00000000250 | ENSBTAG00000000306 |  |  |
| ENSBTAG00000000252 | ENSBTAG00000000308 |  |  |
| ENSBTAG00000000257 | ENSBTAG00000000312 |  |  |
| ENSBTAG00000000258 | ENSBTAG00000000313 |  |  |
| ENSBTAG00000000260 | ENSBTAG00000000317 |  |  |
| ENSBTAG00000000264 | ENSBTAG00000000320 |  |  |
| ENSBTAG00000000265 | ENSBTAG00000000321 |  |  |
| ENSBTAG00000000266 | ENSBTAG00000000329 |  |  |
| ENSBTAG00000000267 | ENSBTAG00000000347 |  |  |
| ENSBTAG00000000271 | ENSBTAG00000000354 |  |  |
| ENSBTAG00000000275 | ENSBTAG00000000355 |  |  |
| ENSBTAG00000000279 | ENSBTAG00000000356 |  |  |
| ENSBTAG00000000284 | ENSBTAG00000000362 |  |  |
| ENSBTAG00000000287 | ENSBTAG00000000365 |  |  |
| ENSBTAG00000000289 | ENSBTAG00000000369 |  |  |
| ENSBTAG00000000290 | ENSBTAG00000000371 |  |  |
| ENSBTAG00000000291 | ENSBTAG00000000372 |  |  |
| ENSBTAG00000000292 | ENSBTAG00000000375 |  |  |
| ENSBTAG00000000295 | ENSBTAG00000000380 |  |  |
| ENSBTAG00000000296 | ENSBTAG00000000382 |  |  |
| ENSBTAG00000000297 | ENSBTAG00000000387 |  |  |
| ENSBTAG00000000305 | ENSBTAG00000000392 |  |  |
| ENSBTAG00000000306 | ENSBTAG00000000393 |  |  |
| ENSBTAG00000000308 | ENSBTAG00000000394 |  |  |
| ENSBTAG00000000312 | ENSBTAG00000000399 |  |  |
| ENSBTAG00000000313 | ENSBTAG00000000400 |  |  |
| ENSBTAG00000000317 | ENSBTAG00000000404 |  |  |
| ENSBTAG00000000320 | ENSBTAG00000000406 |  |  |
| ENSBTAG00000000321 | ENSBTAG00000000409 |  |  |
| ENSBTAG00000000329 | ENSBTAG00000000411 |  |  |
| ENSBTAG00000000330 | ENSBTAG00000000414 |  |  |
| ENSBTAG00000000336 | ENSBTAG00000000415 |  |  |
| ENSBTAG00000000342 | ENSBTAG00000000431 |  |  |
| ENSBTAG00000000345 | ENSBTAG00000000435 |  |  |
| ENSBTAG00000000347 | ENSBTAG00000000442 |  |  |
| ENSBTAG00000000354 | ENSBTAG00000000445 |  |  |
| ENSBTAG00000000355 | ENSBTAG00000000447 |  |  |
| ENSBTAG00000000356 | ENSBTAG00000000454 |  |  |
| ENSBTAG00000000359 | ENSBTAG00000000455 |  |  |
| ENSBTAG00000000362 | ENSBTAG00000000457 |  |  |
| ENSBTAG00000000365 | ENSBTAG00000000460 |  |  |
| ENSBTAG00000000369 | ENSBTAG00000000469 |  |  |
| ENSBTAG00000000371 | ENSBTAG00000000472 |  |  |
| ENSBTAG00000000372 | ENSBTAG00000000475 |  |  |
| ENSBTAG00000000375 | ENSBTAG00000000490 |  |  |
| ENSBTAG00000000379 | ENSBTAG00000000495 |  |  |
| ENSBTAG00000000380 | ENSBTAG00000000501 |  |  |
| ENSBTAG00000000382 | ENSBTAG00000000502 |  |  |
| ENSBTAG00000000387 | ENSBTAG00000000505 |  |  |
| ENSBTAG00000000390 | ENSBTAG00000000507 |  |  |
| ENSBTAG00000000392 | ENSBTAG00000000512 |  |  |
| ENSBTAG00000000393 | ENSBTAG00000000520 |  |  |
| ENSBTAG00000000394 | ENSBTAG00000000522 |  |  |
| ENSBTAG00000000399 | ENSBTAG00000000527 |  |  |
| ENSBTAG00000000400 | ENSBTAG00000000532 |  |  |
| ENSBTAG00000000405 | ENSBTAG00000000534 |  |  |
| ENSBTAG00000000406 | ENSBTAG00000000535 |  |  |
| ENSBTAG00000000409 | ENSBTAG00000000541 |  |  |
| ENSBTAG00000000411 | ENSBTAG00000000545 |  |  |
| ENSBTAG00000000413 | ENSBTAG00000000550 |  |  |
| ENSBTAG00000000414 | ENSBTAG00000000555 |  |  |
| ENSBTAG00000000417 | ENSBTAG00000000558 |  |  |
| ENSBTAG00000000423 | ENSBTAG00000000559 |  |  |
| ENSBTAG00000000431 | ENSBTAG00000000561 |  |  |
| ENSBTAG00000000432 | ENSBTAG00000000563 |  |  |
| ENSBTAG00000000435 | ENSBTAG00000000564 |  |  |
| ENSBTAG00000000445 | ENSBTAG00000000565 |  |  |
| ENSBTAG00000000446 | ENSBTAG00000000566 |  |  |
| ENSBTAG00000000447 | ENSBTAG00000000571 |  |  |
| ENSBTAG00000000454 | ENSBTAG00000000578 |  |  |
| ENSBTAG00000000455 | ENSBTAG00000000588 |  |  |
| ENSBTAG00000000457 | ENSBTAG00000000597 |  |  |
| ENSBTAG00000000459 | ENSBTAG00000000599 |  |  |
| ENSBTAG00000000460 | ENSBTAG00000000603 |  |  |
| ENSBTAG00000000462 | ENSBTAG00000000605 |  |  |
| ENSBTAG00000000469 | ENSBTAG00000000607 |  |  |
| ENSBTAG00000000472 | ENSBTAG00000000613 |  |  |
| ENSBTAG00000000475 | ENSBTAG00000000619 |  |  |
| ENSBTAG00000000476 | ENSBTAG00000000629 |  |  |
| ENSBTAG00000000480 | ENSBTAG00000000630 |  |  |
| ENSBTAG00000000490 | ENSBTAG00000000634 |  |  |
| ENSBTAG00000000495 | ENSBTAG00000000638 |  |  |
| ENSBTAG00000000497 | ENSBTAG00000000639 |  |  |
| ENSBTAG00000000501 | ENSBTAG00000000640 |  |  |
| ENSBTAG00000000502 | ENSBTAG00000000653 |  |  |
| ENSBTAG00000000505 | ENSBTAG00000000666 |  |  |
| ENSBTAG00000000507 | ENSBTAG00000000668 |  |  |
| ENSBTAG00000000510 | ENSBTAG00000000670 |  |  |
| ENSBTAG00000000512 | ENSBTAG00000000672 |  |  |
| ENSBTAG00000000520 | ENSBTAG00000000675 |  |  |
| ENSBTAG00000000522 | ENSBTAG00000000676 |  |  |
| ENSBTAG00000000524 | ENSBTAG00000000678 |  |  |
| ENSBTAG00000000527 | ENSBTAG00000000679 |  |  |
| ENSBTAG00000000532 | ENSBTAG00000000686 |  |  |
| ENSBTAG00000000534 | ENSBTAG00000000687 |  |  |
| ENSBTAG00000000535 | ENSBTAG00000000693 |  |  |
| ENSBTAG00000000536 | ENSBTAG00000000696 |  |  |
| ENSBTAG00000000539 | ENSBTAG00000000703 |  |  |
| ENSBTAG00000000541 | ENSBTAG00000000704 |  |  |
| ENSBTAG00000000545 | ENSBTAG00000000705 |  |  |
| ENSBTAG00000000550 | ENSBTAG00000000710 |  |  |
| ENSBTAG00000000555 | ENSBTAG00000000711 |  |  |
| ENSBTAG00000000558 | ENSBTAG00000000712 |  |  |
| ENSBTAG00000000559 | ENSBTAG00000000717 |  |  |
| ENSBTAG00000000561 | ENSBTAG00000000720 |  |  |
| ENSBTAG00000000562 | ENSBTAG00000000730 |  |  |
| ENSBTAG00000000563 | ENSBTAG00000000731 |  |  |
| ENSBTAG00000000564 | ENSBTAG00000000744 |  |  |
| ENSBTAG00000000565 | ENSBTAG00000000745 |  |  |
| ENSBTAG00000000566 | ENSBTAG00000000748 |  |  |
| ENSBTAG00000000569 | ENSBTAG00000000752 |  |  |
| ENSBTAG00000000571 | ENSBTAG00000000758 |  |  |
| ENSBTAG00000000578 | ENSBTAG00000000764 |  |  |
| ENSBTAG00000000580 | ENSBTAG00000000771 |  |  |
| ENSBTAG00000000581 | ENSBTAG00000000773 |  |  |
| ENSBTAG00000000588 | ENSBTAG00000000782 |  |  |
| ENSBTAG00000000598 | ENSBTAG00000000785 |  |  |
| ENSBTAG00000000599 | ENSBTAG00000000789 |  |  |
| ENSBTAG00000000602 | ENSBTAG00000000795 |  |  |
| ENSBTAG00000000603 | ENSBTAG00000000799 |  |  |
| ENSBTAG00000000604 | ENSBTAG00000000801 |  |  |
| ENSBTAG00000000607 | ENSBTAG00000000803 |  |  |
| ENSBTAG00000000613 | ENSBTAG00000000804 |  |  |
| ENSBTAG00000000619 | ENSBTAG00000000806 |  |  |
| ENSBTAG00000000623 | ENSBTAG00000000809 |  |  |
| ENSBTAG00000000629 | ENSBTAG00000000811 |  |  |
| ENSBTAG00000000630 | ENSBTAG00000000816 |  |  |
| ENSBTAG00000000634 | ENSBTAG00000000824 |  |  |
| ENSBTAG00000000638 | ENSBTAG00000000825 |  |  |
| ENSBTAG00000000639 | ENSBTAG00000000829 |  |  |
| ENSBTAG00000000640 | ENSBTAG00000000831 |  |  |
| ENSBTAG00000000650 | ENSBTAG00000000835 |  |  |
| ENSBTAG00000000653 | ENSBTAG00000000838 |  |  |
| ENSBTAG00000000655 | ENSBTAG00000000854 |  |  |
| ENSBTAG00000000656 | ENSBTAG00000000855 |  |  |
| ENSBTAG00000000658 | ENSBTAG00000000869 |  |  |
| ENSBTAG00000000666 | ENSBTAG00000000873 |  |  |
| ENSBTAG00000000668 | ENSBTAG00000000874 |  |  |
| ENSBTAG00000000672 | ENSBTAG00000000875 |  |  |
| ENSBTAG00000000675 | ENSBTAG00000000878 |  |  |
| ENSBTAG00000000678 | ENSBTAG00000000879 |  |  |
| ENSBTAG00000000679 | ENSBTAG00000000880 |  |  |
| ENSBTAG00000000682 | ENSBTAG00000000888 |  |  |
| ENSBTAG00000000686 | ENSBTAG00000000894 |  |  |
| ENSBTAG00000000687 | ENSBTAG00000000895 |  |  |
| ENSBTAG00000000693 | ENSBTAG00000000898 |  |  |
| ENSBTAG00000000694 | ENSBTAG00000000899 |  |  |
| ENSBTAG00000000696 | ENSBTAG00000000900 |  |  |
| ENSBTAG00000000697 | ENSBTAG00000000902 |  |  |
| ENSBTAG00000000703 | ENSBTAG00000000905 |  |  |
| ENSBTAG00000000704 | ENSBTAG00000000910 |  |  |
| ENSBTAG00000000705 | ENSBTAG00000000919 |  |  |
| ENSBTAG00000000706 | ENSBTAG00000000933 |  |  |
| ENSBTAG00000000710 | ENSBTAG00000000937 |  |  |
| ENSBTAG00000000711 | ENSBTAG00000000940 |  |  |
| ENSBTAG00000000712 | ENSBTAG00000000941 |  |  |
| ENSBTAG00000000717 | ENSBTAG00000000943 |  |  |
| ENSBTAG00000000720 | ENSBTAG00000000946 |  |  |
| ENSBTAG00000000721 | ENSBTAG00000000948 |  |  |
| ENSBTAG00000000730 | ENSBTAG00000000957 |  |  |
| ENSBTAG00000000731 | ENSBTAG00000000959 |  |  |
| ENSBTAG00000000735 | ENSBTAG00000000961 |  |  |
| ENSBTAG00000000736 | ENSBTAG00000000962 |  |  |
| ENSBTAG00000000742 | ENSBTAG00000000964 |  |  |
| ENSBTAG00000000744 | ENSBTAG00000000967 |  |  |
| ENSBTAG00000000745 | ENSBTAG00000000973 |  |  |
| ENSBTAG00000000748 | ENSBTAG00000000974 |  |  |
| ENSBTAG00000000752 | ENSBTAG00000000979 |  |  |
| ENSBTAG00000000753 | ENSBTAG00000000990 |  |  |
| ENSBTAG00000000754 | ENSBTAG00000000993 |  |  |
| ENSBTAG00000000755 | ENSBTAG00000001000 |  |  |
| ENSBTAG00000000758 | ENSBTAG00000001001 |  |  |
| ENSBTAG00000000764 | ENSBTAG00000001002 |  |  |
| ENSBTAG00000000771 | ENSBTAG00000001004 |  |  |
| ENSBTAG00000000773 | ENSBTAG00000001009 |  |  |
| ENSBTAG00000000782 | ENSBTAG00000001013 |  |  |
| ENSBTAG00000000785 | ENSBTAG00000001015 |  |  |
| ENSBTAG00000000791 | ENSBTAG00000001016 |  |  |
| ENSBTAG00000000795 | ENSBTAG00000001019 |  |  |
| ENSBTAG00000000799 | ENSBTAG00000001027 |  |  |
| ENSBTAG00000000801 | ENSBTAG00000001028 |  |  |
| ENSBTAG00000000802 | ENSBTAG00000001030 |  |  |
| ENSBTAG00000000803 | ENSBTAG00000001032 |  |  |
| ENSBTAG00000000804 | ENSBTAG00000001035 |  |  |
| ENSBTAG00000000806 | ENSBTAG00000001036 |  |  |
| ENSBTAG00000000807 | ENSBTAG00000001042 |  |  |
| ENSBTAG00000000808 | ENSBTAG00000001043 |  |  |
| ENSBTAG00000000809 | ENSBTAG00000001044 |  |  |
| ENSBTAG00000000811 | ENSBTAG00000001057 |  |  |
| ENSBTAG00000000815 | ENSBTAG00000001060 |  |  |
| ENSBTAG00000000816 | ENSBTAG00000001063 |  |  |
| ENSBTAG00000000821 | ENSBTAG00000001066 |  |  |
| ENSBTAG00000000824 | ENSBTAG00000001069 |  |  |
| ENSBTAG00000000825 | ENSBTAG00000001074 |  |  |
| ENSBTAG00000000829 | ENSBTAG00000001075 |  |  |
| ENSBTAG00000000831 | ENSBTAG00000001076 |  |  |
| ENSBTAG00000000833 | ENSBTAG00000001077 |  |  |
| ENSBTAG00000000835 | ENSBTAG00000001085 |  |  |
| ENSBTAG00000000837 | ENSBTAG00000001094 |  |  |
| ENSBTAG00000000838 | ENSBTAG00000001098 |  |  |
| ENSBTAG00000000854 | ENSBTAG00000001100 |  |  |
| ENSBTAG00000000855 | ENSBTAG00000001102 |  |  |
| ENSBTAG00000000857 | ENSBTAG00000001104 |  |  |
| ENSBTAG00000000869 | ENSBTAG00000001105 |  |  |
| ENSBTAG00000000873 | ENSBTAG00000001109 |  |  |
| ENSBTAG00000000874 | ENSBTAG00000001110 |  |  |
| ENSBTAG00000000875 | ENSBTAG00000001113 |  |  |
| ENSBTAG00000000878 | ENSBTAG00000001114 |  |  |
| ENSBTAG00000000879 | ENSBTAG00000001116 |  |  |
| ENSBTAG00000000880 | ENSBTAG00000001126 |  |  |
| ENSBTAG00000000888 | ENSBTAG00000001132 |  |  |
| ENSBTAG00000000894 | ENSBTAG00000001133 |  |  |
| ENSBTAG00000000895 | ENSBTAG00000001136 |  |  |
| ENSBTAG00000000898 | ENSBTAG00000001137 |  |  |
| ENSBTAG00000000899 | ENSBTAG00000001140 |  |  |
| ENSBTAG00000000900 | ENSBTAG00000001154 |  |  |
| ENSBTAG00000000902 | ENSBTAG00000001156 |  |  |
| ENSBTAG00000000905 | ENSBTAG00000001161 |  |  |
| ENSBTAG00000000910 | ENSBTAG00000001163 |  |  |
| ENSBTAG00000000913 | ENSBTAG00000001164 |  |  |
| ENSBTAG00000000917 | ENSBTAG00000001165 |  |  |
| ENSBTAG00000000919 | ENSBTAG00000001171 |  |  |
| ENSBTAG00000000925 | ENSBTAG00000001173 |  |  |
| ENSBTAG00000000933 | ENSBTAG00000001174 |  |  |
| ENSBTAG00000000937 | ENSBTAG00000001179 |  |  |
| ENSBTAG00000000939 | ENSBTAG00000001181 |  |  |
| ENSBTAG00000000940 | ENSBTAG00000001182 |  |  |
| ENSBTAG00000000941 | ENSBTAG00000001183 |  |  |
| ENSBTAG00000000942 | ENSBTAG00000001186 |  |  |
| ENSBTAG00000000943 | ENSBTAG00000001187 |  |  |
| ENSBTAG00000000946 | ENSBTAG00000001188 |  |  |
| ENSBTAG00000000948 | ENSBTAG00000001189 |  |  |
| ENSBTAG00000000949 | ENSBTAG00000001192 |  |  |
| ENSBTAG00000000957 | ENSBTAG00000001193 |  |  |
| ENSBTAG00000000959 | ENSBTAG00000001197 |  |  |
| ENSBTAG00000000961 | ENSBTAG00000001199 |  |  |
| ENSBTAG00000000962 | ENSBTAG00000001204 |  |  |
| ENSBTAG00000000963 | ENSBTAG00000001207 |  |  |
| ENSBTAG00000000964 | ENSBTAG00000001209 |  |  |
| ENSBTAG00000000966 | ENSBTAG00000001224 |  |  |
| ENSBTAG00000000967 | ENSBTAG00000001225 |  |  |
| ENSBTAG00000000970 | ENSBTAG00000001228 |  |  |
| ENSBTAG00000000973 | ENSBTAG00000001229 |  |  |
| ENSBTAG00000000974 | ENSBTAG00000001233 |  |  |
| ENSBTAG00000000979 | ENSBTAG00000001243 |  |  |
| ENSBTAG00000000981 | ENSBTAG00000001244 |  |  |
| ENSBTAG00000000985 | ENSBTAG00000001250 |  |  |
| ENSBTAG00000000986 | ENSBTAG00000001254 |  |  |
| ENSBTAG00000000990 | ENSBTAG00000001258 |  |  |
| ENSBTAG00000000991 | ENSBTAG00000001282 |  |  |
| ENSBTAG00000000993 | ENSBTAG00000001283 |  |  |
| ENSBTAG00000001000 | ENSBTAG00000001288 |  |  |
| ENSBTAG00000001001 | ENSBTAG00000001289 |  |  |
| ENSBTAG00000001002 | ENSBTAG00000001290 |  |  |
| ENSBTAG00000001004 | ENSBTAG00000001292 |  |  |
| ENSBTAG00000001007 | ENSBTAG00000001303 |  |  |
| ENSBTAG00000001009 | ENSBTAG00000001306 |  |  |
| ENSBTAG00000001013 | ENSBTAG00000001308 |  |  |
| ENSBTAG00000001014 | ENSBTAG00000001311 |  |  |
| ENSBTAG00000001015 | ENSBTAG00000001321 |  |  |
| ENSBTAG00000001016 | ENSBTAG00000001323 |  |  |
| ENSBTAG00000001019 | ENSBTAG00000001329 |  |  |
| ENSBTAG00000001020 | ENSBTAG00000001332 |  |  |
| ENSBTAG00000001023 | ENSBTAG00000001335 |  |  |
| ENSBTAG00000001027 | ENSBTAG00000001342 |  |  |
| ENSBTAG00000001028 | ENSBTAG00000001344 |  |  |
| ENSBTAG00000001030 | ENSBTAG00000001348 |  |  |
| ENSBTAG00000001032 | ENSBTAG00000001352 |  |  |
| ENSBTAG00000001035 | ENSBTAG00000001353 |  |  |
| ENSBTAG00000001036 | ENSBTAG00000001356 |  |  |
| ENSBTAG00000001037 | ENSBTAG00000001361 |  |  |
| ENSBTAG00000001041 | ENSBTAG00000001364 |  |  |
| ENSBTAG00000001042 | ENSBTAG00000001368 |  |  |
| ENSBTAG00000001043 | ENSBTAG00000001385 |  |  |
| ENSBTAG00000001044 | ENSBTAG00000001390 |  |  |
| ENSBTAG00000001049 | ENSBTAG00000001391 |  |  |
| ENSBTAG00000001050 | ENSBTAG00000001394 |  |  |
| ENSBTAG00000001057 | ENSBTAG00000001395 |  |  |
| ENSBTAG00000001058 | ENSBTAG00000001398 |  |  |
| ENSBTAG00000001059 | ENSBTAG00000001404 |  |  |
| ENSBTAG00000001060 | ENSBTAG00000001405 |  |  |
| ENSBTAG00000001063 | ENSBTAG00000001407 |  |  |
| ENSBTAG00000001066 | ENSBTAG00000001408 |  |  |
| ENSBTAG00000001068 | ENSBTAG00000001410 |  |  |
| ENSBTAG00000001069 | ENSBTAG00000001412 |  |  |
| ENSBTAG00000001074 | ENSBTAG00000001415 |  |  |
| ENSBTAG00000001075 | ENSBTAG00000001420 |  |  |
| ENSBTAG00000001076 | ENSBTAG00000001422 |  |  |
| ENSBTAG00000001077 | ENSBTAG00000001435 |  |  |
| ENSBTAG00000001085 | ENSBTAG00000001440 |  |  |
| ENSBTAG00000001094 | ENSBTAG00000001444 |  |  |
| ENSBTAG00000001096 | ENSBTAG00000001446 |  |  |
| ENSBTAG00000001097 | ENSBTAG00000001450 |  |  |
| ENSBTAG00000001098 | ENSBTAG00000001457 |  |  |
| ENSBTAG00000001099 | ENSBTAG00000001460 |  |  |
| ENSBTAG00000001100 | ENSBTAG00000001463 |  |  |
| ENSBTAG00000001102 | ENSBTAG00000001468 |  |  |
| ENSBTAG00000001104 | ENSBTAG00000001470 |  |  |
| ENSBTAG00000001105 | ENSBTAG00000001474 |  |  |
| ENSBTAG00000001107 | ENSBTAG00000001481 |  |  |
| ENSBTAG00000001108 | ENSBTAG00000001485 |  |  |
| ENSBTAG00000001109 | ENSBTAG00000001488 |  |  |
| ENSBTAG00000001110 | ENSBTAG00000001489 |  |  |
| ENSBTAG00000001112 | ENSBTAG00000001492 |  |  |
| ENSBTAG00000001113 | ENSBTAG00000001495 |  |  |
| ENSBTAG00000001116 | ENSBTAG00000001497 |  |  |
| ENSBTAG00000001120 | ENSBTAG00000001504 |  |  |
| ENSBTAG00000001126 | ENSBTAG00000001505 |  |  |
| ENSBTAG00000001133 | ENSBTAG00000001509 |  |  |
| ENSBTAG00000001134 | ENSBTAG00000001512 |  |  |
| ENSBTAG00000001136 | ENSBTAG00000001513 |  |  |
| ENSBTAG00000001137 | ENSBTAG00000001516 |  |  |
| ENSBTAG00000001138 | ENSBTAG00000001517 |  |  |
| ENSBTAG00000001139 | ENSBTAG00000001518 |  |  |
| ENSBTAG00000001140 | ENSBTAG00000001523 |  |  |
| ENSBTAG00000001144 | ENSBTAG00000001527 |  |  |
| ENSBTAG00000001150 | ENSBTAG00000001529 |  |  |
| ENSBTAG00000001153 | ENSBTAG00000001533 |  |  |
| ENSBTAG00000001154 | ENSBTAG00000001537 |  |  |
| ENSBTAG00000001156 | ENSBTAG00000001539 |  |  |
| ENSBTAG00000001161 | ENSBTAG00000001546 |  |  |
| ENSBTAG00000001163 | ENSBTAG00000001551 |  |  |
| ENSBTAG00000001164 | ENSBTAG00000001558 |  |  |
| ENSBTAG00000001165 | ENSBTAG00000001562 |  |  |
| ENSBTAG00000001171 | ENSBTAG00000001564 |  |  |
| ENSBTAG00000001173 | ENSBTAG00000001565 |  |  |
| ENSBTAG00000001174 | ENSBTAG00000001567 |  |  |
| ENSBTAG00000001179 | ENSBTAG00000001568 |  |  |
| ENSBTAG00000001181 | ENSBTAG00000001570 |  |  |
| ENSBTAG00000001182 | ENSBTAG00000001573 |  |  |
| ENSBTAG00000001183 | ENSBTAG00000001574 |  |  |
| ENSBTAG00000001186 | ENSBTAG00000001576 |  |  |
| ENSBTAG00000001187 | ENSBTAG00000001578 |  |  |
| ENSBTAG00000001188 | ENSBTAG00000001579 |  |  |
| ENSBTAG00000001189 | ENSBTAG00000001580 |  |  |
| ENSBTAG00000001191 | ENSBTAG00000001585 |  |  |
| ENSBTAG00000001192 | ENSBTAG00000001592 |  |  |
| ENSBTAG00000001193 | ENSBTAG00000001594 |  |  |
| ENSBTAG00000001197 | ENSBTAG00000001598 |  |  |
| ENSBTAG00000001199 | ENSBTAG00000001599 |  |  |
| ENSBTAG00000001204 | ENSBTAG00000001600 |  |  |
| ENSBTAG00000001207 | ENSBTAG00000001602 |  |  |
| ENSBTAG00000001209 | ENSBTAG00000001603 |  |  |
| ENSBTAG00000001224 | ENSBTAG00000001607 |  |  |
| ENSBTAG00000001225 | ENSBTAG00000001610 |  |  |
| ENSBTAG00000001228 | ENSBTAG00000001614 |  |  |
| ENSBTAG00000001229 | ENSBTAG00000001616 |  |  |
| ENSBTAG00000001231 | ENSBTAG00000001617 |  |  |
| ENSBTAG00000001233 | ENSBTAG00000001619 |  |  |
| ENSBTAG00000001235 | ENSBTAG00000001626 |  |  |
| ENSBTAG00000001243 | ENSBTAG00000001627 |  |  |
| ENSBTAG00000001244 | ENSBTAG00000001634 |  |  |
| ENSBTAG00000001250 | ENSBTAG00000001635 |  |  |
| ENSBTAG00000001254 | ENSBTAG00000001647 |  |  |
| ENSBTAG00000001258 | ENSBTAG00000001648 |  |  |
| ENSBTAG00000001279 | ENSBTAG00000001651 |  |  |
| ENSBTAG00000001280 | ENSBTAG00000001656 |  |  |
| ENSBTAG00000001282 | ENSBTAG00000001659 |  |  |
| ENSBTAG00000001283 | ENSBTAG00000001662 |  |  |
| ENSBTAG00000001286 | ENSBTAG00000001671 |  |  |
| ENSBTAG00000001288 | ENSBTAG00000001673 |  |  |
| ENSBTAG00000001289 | ENSBTAG00000001675 |  |  |
| ENSBTAG00000001290 | ENSBTAG00000001687 |  |  |
| ENSBTAG00000001292 | ENSBTAG00000001692 |  |  |
| ENSBTAG00000001296 | ENSBTAG00000001693 |  |  |
| ENSBTAG00000001302 | ENSBTAG00000001696 |  |  |
| ENSBTAG00000001303 | ENSBTAG00000001697 |  |  |
| ENSBTAG00000001306 | ENSBTAG00000001698 |  |  |
| ENSBTAG00000001308 | ENSBTAG00000001702 |  |  |
| ENSBTAG00000001311 | ENSBTAG00000001703 |  |  |
| ENSBTAG00000001321 | ENSBTAG00000001707 |  |  |
| ENSBTAG00000001322 | ENSBTAG00000001708 |  |  |
| ENSBTAG00000001323 | ENSBTAG00000001710 |  |  |
| ENSBTAG00000001325 | ENSBTAG00000001717 |  |  |
| ENSBTAG00000001328 | ENSBTAG00000001725 |  |  |
| ENSBTAG00000001329 | ENSBTAG00000001727 |  |  |
| ENSBTAG00000001332 | ENSBTAG00000001729 |  |  |
| ENSBTAG00000001335 | ENSBTAG00000001731 |  |  |
| ENSBTAG00000001342 | ENSBTAG00000001737 |  |  |
| ENSBTAG00000001344 | ENSBTAG00000001740 |  |  |
| ENSBTAG00000001348 | ENSBTAG00000001741 |  |  |
| ENSBTAG00000001352 | ENSBTAG00000001744 |  |  |
| ENSBTAG00000001353 | ENSBTAG00000001745 |  |  |
| ENSBTAG00000001356 | ENSBTAG00000001749 |  |  |
| ENSBTAG00000001361 | ENSBTAG00000001752 |  |  |
| ENSBTAG00000001364 | ENSBTAG00000001754 |  |  |
| ENSBTAG00000001368 | ENSBTAG00000001755 |  |  |
| ENSBTAG00000001385 | ENSBTAG00000001762 |  |  |
| ENSBTAG00000001388 | ENSBTAG00000001763 |  |  |
| ENSBTAG00000001390 | ENSBTAG00000001764 |  |  |
| ENSBTAG00000001391 | ENSBTAG00000001767 |  |  |
| ENSBTAG00000001392 | ENSBTAG00000001773 |  |  |
| ENSBTAG00000001394 | ENSBTAG00000001774 |  |  |
| ENSBTAG00000001395 | ENSBTAG00000001785 |  |  |
| ENSBTAG00000001398 | ENSBTAG00000001786 |  |  |
| ENSBTAG00000001404 | ENSBTAG00000001788 |  |  |
| ENSBTAG00000001405 | ENSBTAG00000001793 |  |  |
| ENSBTAG00000001407 | ENSBTAG00000001801 |  |  |
| ENSBTAG00000001408 | ENSBTAG00000001805 |  |  |
| ENSBTAG00000001410 | ENSBTAG00000001807 |  |  |
| ENSBTAG00000001412 | ENSBTAG00000001810 |  |  |
| ENSBTAG00000001415 | ENSBTAG00000001815 |  |  |
| ENSBTAG00000001417 | ENSBTAG00000001817 |  |  |
| ENSBTAG00000001419 | ENSBTAG00000001822 |  |  |
| ENSBTAG00000001420 | ENSBTAG00000001827 |  |  |
| ENSBTAG00000001422 | ENSBTAG00000001829 |  |  |
| ENSBTAG00000001435 | ENSBTAG00000001835 |  |  |
| ENSBTAG00000001440 | ENSBTAG00000001839 |  |  |
| ENSBTAG00000001441 | ENSBTAG00000001842 |  |  |
| ENSBTAG00000001444 | ENSBTAG00000001843 |  |  |
| ENSBTAG00000001446 | ENSBTAG00000001847 |  |  |
| ENSBTAG00000001449 | ENSBTAG00000001851 |  |  |
| ENSBTAG00000001450 | ENSBTAG00000001854 |  |  |
| ENSBTAG00000001457 | ENSBTAG00000001855 |  |  |
| ENSBTAG00000001460 | ENSBTAG00000001856 |  |  |
| ENSBTAG00000001462 | ENSBTAG00000001857 |  |  |
| ENSBTAG00000001463 | ENSBTAG00000001862 |  |  |
| ENSBTAG00000001468 | ENSBTAG00000001865 |  |  |
| ENSBTAG00000001470 | ENSBTAG00000001868 |  |  |
| ENSBTAG00000001473 | ENSBTAG00000001870 |  |  |
| ENSBTAG00000001474 | ENSBTAG00000001872 |  |  |
| ENSBTAG00000001481 | ENSBTAG00000001881 |  |  |
| ENSBTAG00000001485 | ENSBTAG00000001888 |  |  |
| ENSBTAG00000001488 | ENSBTAG00000001889 |  |  |
| ENSBTAG00000001492 | ENSBTAG00000001894 |  |  |
| ENSBTAG00000001495 | ENSBTAG00000001898 |  |  |
| ENSBTAG00000001497 | ENSBTAG00000001904 |  |  |
| ENSBTAG00000001505 | ENSBTAG00000001906 |  |  |
| ENSBTAG00000001506 | ENSBTAG00000001908 |  |  |
| ENSBTAG00000001508 | ENSBTAG00000001922 |  |  |
| ENSBTAG00000001509 | ENSBTAG00000001926 |  |  |
| ENSBTAG00000001512 | ENSBTAG00000001931 |  |  |
| ENSBTAG00000001513 | ENSBTAG00000001932 |  |  |
| ENSBTAG00000001514 | ENSBTAG00000001933 |  |  |
| ENSBTAG00000001516 | ENSBTAG00000001937 |  |  |
| ENSBTAG00000001517 | ENSBTAG00000001938 |  |  |
| ENSBTAG00000001518 | ENSBTAG00000001939 |  |  |
| ENSBTAG00000001523 | ENSBTAG00000001942 |  |  |
| ENSBTAG00000001527 | ENSBTAG00000001945 |  |  |
| ENSBTAG00000001529 | ENSBTAG00000001949 |  |  |
| ENSBTAG00000001533 | ENSBTAG00000001966 |  |  |
| ENSBTAG00000001537 | ENSBTAG00000001968 |  |  |
| ENSBTAG00000001539 | ENSBTAG00000001977 |  |  |
| ENSBTAG00000001543 | ENSBTAG00000001983 |  |  |
| ENSBTAG00000001546 | ENSBTAG00000001987 |  |  |
| ENSBTAG00000001551 | ENSBTAG00000001992 |  |  |
| ENSBTAG00000001564 | ENSBTAG00000001999 |  |  |
| ENSBTAG00000001565 | ENSBTAG00000002006 |  |  |
| ENSBTAG00000001567 | ENSBTAG00000002010 |  |  |
| ENSBTAG00000001568 | ENSBTAG00000002011 |  |  |
| ENSBTAG00000001570 | ENSBTAG00000002014 |  |  |
| ENSBTAG00000001573 | ENSBTAG00000002020 |  |  |
| ENSBTAG00000001574 | ENSBTAG00000002021 |  |  |
| ENSBTAG00000001576 | ENSBTAG00000002028 |  |  |
| ENSBTAG00000001578 | ENSBTAG00000002033 |  |  |
| ENSBTAG00000001579 | ENSBTAG00000002037 |  |  |
| ENSBTAG00000001580 | ENSBTAG00000002039 |  |  |
| ENSBTAG00000001582 | ENSBTAG00000002041 |  |  |
| ENSBTAG00000001585 | ENSBTAG00000002042 |  |  |
| ENSBTAG00000001592 | ENSBTAG00000002046 |  |  |
| ENSBTAG00000001593 | ENSBTAG00000002048 |  |  |
| ENSBTAG00000001594 | ENSBTAG00000002052 |  |  |
| ENSBTAG00000001595 | ENSBTAG00000002056 |  |  |
| ENSBTAG00000001598 | ENSBTAG00000002062 |  |  |
| ENSBTAG00000001599 | ENSBTAG00000002065 |  |  |
| ENSBTAG00000001600 | ENSBTAG00000002069 |  |  |
| ENSBTAG00000001601 | ENSBTAG00000002072 |  |  |
| ENSBTAG00000001602 | ENSBTAG00000002080 |  |  |
| ENSBTAG00000001603 | ENSBTAG00000002081 |  |  |
| ENSBTAG00000001607 | ENSBTAG00000002082 |  |  |
| ENSBTAG00000001610 | ENSBTAG00000002086 |  |  |
| ENSBTAG00000001614 | ENSBTAG00000002090 |  |  |
| ENSBTAG00000001615 | ENSBTAG00000002092 |  |  |
| ENSBTAG00000001616 | ENSBTAG00000002096 |  |  |
| ENSBTAG00000001617 | ENSBTAG00000002098 |  |  |
| ENSBTAG00000001618 | ENSBTAG00000002101 |  |  |
| ENSBTAG00000001619 | ENSBTAG00000002103 |  |  |
| ENSBTAG00000001626 | ENSBTAG00000002105 |  |  |
| ENSBTAG00000001627 | ENSBTAG00000002107 |  |  |
| ENSBTAG00000001632 | ENSBTAG00000002108 |  |  |
| ENSBTAG00000001634 | ENSBTAG00000002112 |  |  |
| ENSBTAG00000001635 | ENSBTAG00000002115 |  |  |
| ENSBTAG00000001645 | ENSBTAG00000002116 |  |  |
| ENSBTAG00000001647 | ENSBTAG00000002121 |  |  |
| ENSBTAG00000001648 | ENSBTAG00000002125 |  |  |
| ENSBTAG00000001656 | ENSBTAG00000002126 |  |  |
| ENSBTAG00000001659 | ENSBTAG00000002127 |  |  |
| ENSBTAG00000001662 | ENSBTAG00000002129 |  |  |
| ENSBTAG00000001663 | ENSBTAG00000002130 |  |  |
| ENSBTAG00000001671 | ENSBTAG00000002135 |  |  |
| ENSBTAG00000001673 | ENSBTAG00000002138 |  |  |
| ENSBTAG00000001675 | ENSBTAG00000002140 |  |  |
| ENSBTAG00000001687 | ENSBTAG00000002148 |  |  |
| ENSBTAG00000001692 | ENSBTAG00000002151 |  |  |
| ENSBTAG00000001693 | ENSBTAG00000002163 |  |  |
| ENSBTAG00000001694 | ENSBTAG00000002174 |  |  |
| ENSBTAG00000001696 | ENSBTAG00000002179 |  |  |
| ENSBTAG00000001697 | ENSBTAG00000002181 |  |  |
| ENSBTAG00000001698 | ENSBTAG00000002184 |  |  |
| ENSBTAG00000001700 | ENSBTAG00000002185 |  |  |
| ENSBTAG00000001702 | ENSBTAG00000002186 |  |  |
| ENSBTAG00000001703 | ENSBTAG00000002187 |  |  |
| ENSBTAG00000001707 | ENSBTAG00000002190 |  |  |
| ENSBTAG00000001708 | ENSBTAG00000002191 |  |  |
| ENSBTAG00000001710 | ENSBTAG00000002192 |  |  |
| ENSBTAG00000001711 | ENSBTAG00000002196 |  |  |
| ENSBTAG00000001712 | ENSBTAG00000002205 |  |  |
| ENSBTAG00000001717 | ENSBTAG00000002209 |  |  |
| ENSBTAG00000001725 | ENSBTAG00000002211 |  |  |
| ENSBTAG00000001727 | ENSBTAG00000002214 |  |  |
| ENSBTAG00000001729 | ENSBTAG00000002215 |  |  |
| ENSBTAG00000001730 | ENSBTAG00000002216 |  |  |
| ENSBTAG00000001731 | ENSBTAG00000002219 |  |  |
| ENSBTAG00000001736 | ENSBTAG00000002220 |  |  |
| ENSBTAG00000001737 | ENSBTAG00000002224 |  |  |
| ENSBTAG00000001740 | ENSBTAG00000002227 |  |  |
| ENSBTAG00000001741 | ENSBTAG00000002243 |  |  |
| ENSBTAG00000001744 | ENSBTAG00000002248 |  |  |
| ENSBTAG00000001745 | ENSBTAG00000002253 |  |  |
| ENSBTAG00000001748 | ENSBTAG00000002255 |  |  |
| ENSBTAG00000001749 | ENSBTAG00000002260 |  |  |
| ENSBTAG00000001752 | ENSBTAG00000002261 |  |  |
| ENSBTAG00000001754 | ENSBTAG00000002267 |  |  |
| ENSBTAG00000001755 | ENSBTAG00000002272 |  |  |
| ENSBTAG00000001762 | ENSBTAG00000002275 |  |  |
| ENSBTAG00000001763 | ENSBTAG00000002279 |  |  |
| ENSBTAG00000001764 | ENSBTAG00000002280 |  |  |
| ENSBTAG00000001767 | ENSBTAG00000002282 |  |  |
| ENSBTAG00000001772 | ENSBTAG00000002283 |  |  |
| ENSBTAG00000001773 | ENSBTAG00000002286 |  |  |
| ENSBTAG00000001774 | ENSBTAG00000002293 |  |  |
| ENSBTAG00000001776 | ENSBTAG00000002296 |  |  |
| ENSBTAG00000001780 | ENSBTAG00000002298 |  |  |
| ENSBTAG00000001781 | ENSBTAG00000002303 |  |  |
| ENSBTAG00000001782 | ENSBTAG00000002306 |  |  |
| ENSBTAG00000001785 | ENSBTAG00000002311 |  |  |
| ENSBTAG00000001786 | ENSBTAG00000002313 |  |  |
| ENSBTAG00000001788 | ENSBTAG00000002315 |  |  |
| ENSBTAG00000001793 | ENSBTAG00000002317 |  |  |
| ENSBTAG00000001794 | ENSBTAG00000002321 |  |  |
| ENSBTAG00000001801 | ENSBTAG00000002323 |  |  |
| ENSBTAG00000001805 | ENSBTAG00000002329 |  |  |
| ENSBTAG00000001807 | ENSBTAG00000002331 |  |  |
| ENSBTAG00000001810 | ENSBTAG00000002332 |  |  |
| ENSBTAG00000001815 | ENSBTAG00000002335 |  |  |
| ENSBTAG00000001816 | ENSBTAG00000002341 |  |  |
| ENSBTAG00000001817 | ENSBTAG00000002344 |  |  |
| ENSBTAG00000001822 | ENSBTAG00000002349 |  |  |
| ENSBTAG00000001824 | ENSBTAG00000002350 |  |  |
| ENSBTAG00000001827 | ENSBTAG00000002352 |  |  |
| ENSBTAG00000001829 | ENSBTAG00000002357 |  |  |
| ENSBTAG00000001832 | ENSBTAG00000002361 |  |  |
| ENSBTAG00000001835 | ENSBTAG00000002362 |  |  |
| ENSBTAG00000001839 | ENSBTAG00000002367 |  |  |
| ENSBTAG00000001840 | ENSBTAG00000002369 |  |  |
| ENSBTAG00000001842 | ENSBTAG00000002376 |  |  |
| ENSBTAG00000001843 | ENSBTAG00000002378 |  |  |
| ENSBTAG00000001847 | ENSBTAG00000002381 |  |  |
| ENSBTAG00000001848 | ENSBTAG00000002382 |  |  |
| ENSBTAG00000001851 | ENSBTAG00000002389 |  |  |
| ENSBTAG00000001852 | ENSBTAG00000002390 |  |  |
| ENSBTAG00000001854 | ENSBTAG00000002392 |  |  |
| ENSBTAG00000001855 | ENSBTAG00000002393 |  |  |
| ENSBTAG00000001856 | ENSBTAG00000002394 |  |  |
| ENSBTAG00000001857 | ENSBTAG00000002395 |  |  |
| ENSBTAG00000001861 | ENSBTAG00000002402 |  |  |
| ENSBTAG00000001862 | ENSBTAG00000002404 |  |  |
| ENSBTAG00000001864 | ENSBTAG00000002411 |  |  |
| ENSBTAG00000001865 | ENSBTAG00000002413 |  |  |
| ENSBTAG00000001866 | ENSBTAG00000002414 |  |  |
| ENSBTAG00000001868 | ENSBTAG00000002418 |  |  |
| ENSBTAG00000001870 | ENSBTAG00000002419 |  |  |
| ENSBTAG00000001872 | ENSBTAG00000002425 |  |  |
| ENSBTAG00000001881 | ENSBTAG00000002430 |  |  |
| ENSBTAG00000001882 | ENSBTAG00000002448 |  |  |
| ENSBTAG00000001885 | ENSBTAG00000002454 |  |  |
| ENSBTAG00000001887 | ENSBTAG00000002455 |  |  |
| ENSBTAG00000001888 | ENSBTAG00000002457 |  |  |
| ENSBTAG00000001889 | ENSBTAG00000002460 |  |  |
| ENSBTAG00000001892 | ENSBTAG00000002463 |  |  |
| ENSBTAG00000001893 | ENSBTAG00000002469 |  |  |
| ENSBTAG00000001894 | ENSBTAG00000002472 |  |  |
| ENSBTAG00000001898 | ENSBTAG00000002474 |  |  |
| ENSBTAG00000001904 | ENSBTAG00000002476 |  |  |
| ENSBTAG00000001906 | ENSBTAG00000002477 |  |  |
| ENSBTAG00000001908 | ENSBTAG00000002479 |  |  |
| ENSBTAG00000001917 | ENSBTAG00000002483 |  |  |
| ENSBTAG00000001922 | ENSBTAG00000002484 |  |  |
| ENSBTAG00000001926 | ENSBTAG00000002487 |  |  |
| ENSBTAG00000001928 | ENSBTAG00000002489 |  |  |
| ENSBTAG00000001931 | ENSBTAG00000002490 |  |  |
| ENSBTAG00000001932 | ENSBTAG00000002492 |  |  |
| ENSBTAG00000001933 | ENSBTAG00000002508 |  |  |
| ENSBTAG00000001936 | ENSBTAG00000002522 |  |  |
| ENSBTAG00000001937 | ENSBTAG00000002524 |  |  |
| ENSBTAG00000001939 | ENSBTAG00000002525 |  |  |
| ENSBTAG00000001941 | ENSBTAG00000002526 |  |  |
| ENSBTAG00000001942 | ENSBTAG00000002531 |  |  |
| ENSBTAG00000001945 | ENSBTAG00000002534 |  |  |
| ENSBTAG00000001949 | ENSBTAG00000002542 |  |  |
| ENSBTAG00000001962 | ENSBTAG00000002554 |  |  |
| ENSBTAG00000001966 | ENSBTAG00000002557 |  |  |
| ENSBTAG00000001968 | ENSBTAG00000002561 |  |  |
| ENSBTAG00000001977 | ENSBTAG00000002562 |  |  |
| ENSBTAG00000001983 | ENSBTAG00000002566 |  |  |
| ENSBTAG00000001987 | ENSBTAG00000002570 |  |  |
| ENSBTAG00000001992 | ENSBTAG00000002573 |  |  |
| ENSBTAG00000001996 | ENSBTAG00000002575 |  |  |
| ENSBTAG00000001998 | ENSBTAG00000002578 |  |  |
| ENSBTAG00000001999 | ENSBTAG00000002581 |  |  |
| ENSBTAG00000002000 | ENSBTAG00000002582 |  |  |
| ENSBTAG00000002006 | ENSBTAG00000002583 |  |  |
| ENSBTAG00000002010 | ENSBTAG00000002586 |  |  |
| ENSBTAG00000002011 | ENSBTAG00000002590 |  |  |
| ENSBTAG00000002014 | ENSBTAG00000002591 |  |  |
| ENSBTAG00000002015 | ENSBTAG00000002593 |  |  |
| ENSBTAG00000002018 | ENSBTAG00000002596 |  |  |
| ENSBTAG00000002019 | ENSBTAG00000002610 |  |  |
| ENSBTAG00000002020 | ENSBTAG00000002612 |  |  |
| ENSBTAG00000002021 | ENSBTAG00000002615 |  |  |
| ENSBTAG00000002027 | ENSBTAG00000002623 |  |  |
| ENSBTAG00000002028 | ENSBTAG00000002624 |  |  |
| ENSBTAG00000002030 | ENSBTAG00000002625 |  |  |
| ENSBTAG00000002033 | ENSBTAG00000002628 |  |  |
| ENSBTAG00000002036 | ENSBTAG00000002632 |  |  |
| ENSBTAG00000002037 | ENSBTAG00000002634 |  |  |
| ENSBTAG00000002039 | ENSBTAG00000002641 |  |  |
| ENSBTAG00000002041 | ENSBTAG00000002642 |  |  |
| ENSBTAG00000002042 | ENSBTAG00000002643 |  |  |
| ENSBTAG00000002045 | ENSBTAG00000002645 |  |  |
| ENSBTAG00000002046 | ENSBTAG00000002651 |  |  |
| ENSBTAG00000002048 | ENSBTAG00000002654 |  |  |
| ENSBTAG00000002049 | ENSBTAG00000002655 |  |  |
| ENSBTAG00000002052 | ENSBTAG00000002676 |  |  |
| ENSBTAG00000002056 | ENSBTAG00000002691 |  |  |
| ENSBTAG00000002058 | ENSBTAG00000002693 |  |  |
| ENSBTAG00000002062 | ENSBTAG00000002697 |  |  |
| ENSBTAG00000002065 | ENSBTAG00000002699 |  |  |
| ENSBTAG00000002068 | ENSBTAG00000002700 |  |  |
| ENSBTAG00000002069 | ENSBTAG00000002701 |  |  |
| ENSBTAG00000002072 | ENSBTAG00000002703 |  |  |
| ENSBTAG00000002073 | ENSBTAG00000002707 |  |  |
| ENSBTAG00000002075 | ENSBTAG00000002714 |  |  |
| ENSBTAG00000002076 | ENSBTAG00000002716 |  |  |
| ENSBTAG00000002078 | ENSBTAG00000002717 |  |  |
| ENSBTAG00000002080 | ENSBTAG00000002720 |  |  |
| ENSBTAG00000002081 | ENSBTAG00000002724 |  |  |
| ENSBTAG00000002082 | ENSBTAG00000002725 |  |  |
| ENSBTAG00000002084 | ENSBTAG00000002726 |  |  |
| ENSBTAG00000002085 | ENSBTAG00000002727 |  |  |
| ENSBTAG00000002086 | ENSBTAG00000002730 |  |  |
| ENSBTAG00000002090 | ENSBTAG00000002733 |  |  |
| ENSBTAG00000002092 | ENSBTAG00000002734 |  |  |
| ENSBTAG00000002096 | ENSBTAG00000002736 |  |  |
| ENSBTAG00000002098 | ENSBTAG00000002738 |  |  |
| ENSBTAG00000002101 | ENSBTAG00000002745 |  |  |
| ENSBTAG00000002103 | ENSBTAG00000002746 |  |  |
| ENSBTAG00000002105 | ENSBTAG00000002748 |  |  |
| ENSBTAG00000002107 | ENSBTAG00000002758 |  |  |
| ENSBTAG00000002108 | ENSBTAG00000002768 |  |  |
| ENSBTAG00000002112 | ENSBTAG00000002769 |  |  |
| ENSBTAG00000002115 | ENSBTAG00000002770 |  |  |
| ENSBTAG00000002116 | ENSBTAG00000002772 |  |  |
| ENSBTAG00000002117 | ENSBTAG00000002778 |  |  |
| ENSBTAG00000002121 | ENSBTAG00000002779 |  |  |
| ENSBTAG00000002125 | ENSBTAG00000002786 |  |  |
| ENSBTAG00000002126 | ENSBTAG00000002791 |  |  |
| ENSBTAG00000002127 | ENSBTAG00000002792 |  |  |
| ENSBTAG00000002129 | ENSBTAG00000002804 |  |  |
| ENSBTAG00000002130 | ENSBTAG00000002810 |  |  |
| ENSBTAG00000002135 | ENSBTAG00000002813 |  |  |
| ENSBTAG00000002136 | ENSBTAG00000002816 |  |  |
| ENSBTAG00000002138 | ENSBTAG00000002817 |  |  |
| ENSBTAG00000002148 | ENSBTAG00000002823 |  |  |
| ENSBTAG00000002151 | ENSBTAG00000002828 |  |  |
| ENSBTAG00000002163 | ENSBTAG00000002830 |  |  |
| ENSBTAG00000002166 | ENSBTAG00000002833 |  |  |
| ENSBTAG00000002174 | ENSBTAG00000002834 |  |  |
| ENSBTAG00000002179 | ENSBTAG00000002835 |  |  |
| ENSBTAG00000002181 | ENSBTAG00000002845 |  |  |
| ENSBTAG00000002184 | ENSBTAG00000002846 |  |  |
| ENSBTAG00000002185 | ENSBTAG00000002849 |  |  |
| ENSBTAG00000002186 | ENSBTAG00000002856 |  |  |
| ENSBTAG00000002187 | ENSBTAG00000002858 |  |  |
| ENSBTAG00000002190 | ENSBTAG00000002859 |  |  |
| ENSBTAG00000002191 | ENSBTAG00000002863 |  |  |
| ENSBTAG00000002192 | ENSBTAG00000002868 |  |  |
| ENSBTAG00000002194 | ENSBTAG00000002871 |  |  |
| ENSBTAG00000002196 | ENSBTAG00000002874 |  |  |
| ENSBTAG00000002203 | ENSBTAG00000002878 |  |  |
| ENSBTAG00000002205 | ENSBTAG00000002879 |  |  |
| ENSBTAG00000002209 | ENSBTAG00000002880 |  |  |
| ENSBTAG00000002211 | ENSBTAG00000002883 |  |  |
| ENSBTAG00000002214 | ENSBTAG00000002885 |  |  |
| ENSBTAG00000002215 | ENSBTAG00000002887 |  |  |
| ENSBTAG00000002216 | ENSBTAG00000002891 |  |  |
| ENSBTAG00000002219 | ENSBTAG00000002895 |  |  |
| ENSBTAG00000002220 | ENSBTAG00000002896 |  |  |
| ENSBTAG00000002223 | ENSBTAG00000002907 |  |  |
| ENSBTAG00000002224 | ENSBTAG00000002908 |  |  |
| ENSBTAG00000002227 | ENSBTAG00000002917 |  |  |
| ENSBTAG00000002233 | ENSBTAG00000002918 |  |  |
| ENSBTAG00000002238 | ENSBTAG00000002921 |  |  |
| ENSBTAG00000002240 | ENSBTAG00000002923 |  |  |
| ENSBTAG00000002242 | ENSBTAG00000002940 |  |  |
| ENSBTAG00000002243 | ENSBTAG00000002942 |  |  |
| ENSBTAG00000002247 | ENSBTAG00000002947 |  |  |
| ENSBTAG00000002248 | ENSBTAG00000002948 |  |  |
| ENSBTAG00000002249 | ENSBTAG00000002949 |  |  |
| ENSBTAG00000002253 | ENSBTAG00000002952 |  |  |
| ENSBTAG00000002255 | ENSBTAG00000002956 |  |  |
| ENSBTAG00000002261 | ENSBTAG00000002960 |  |  |
| ENSBTAG00000002267 | ENSBTAG00000002966 |  |  |
| ENSBTAG00000002272 | ENSBTAG00000002970 |  |  |
| ENSBTAG00000002275 | ENSBTAG00000002971 |  |  |
| ENSBTAG00000002278 | ENSBTAG00000002974 |  |  |
| ENSBTAG00000002279 | ENSBTAG00000002977 |  |  |
| ENSBTAG00000002280 | ENSBTAG00000002980 |  |  |
| ENSBTAG00000002282 | ENSBTAG00000002981 |  |  |
| ENSBTAG00000002283 | ENSBTAG00000002985 |  |  |
| ENSBTAG00000002286 | ENSBTAG00000002988 |  |  |
| ENSBTAG00000002292 | ENSBTAG00000002993 |  |  |
| ENSBTAG00000002293 | ENSBTAG00000002997 |  |  |
| ENSBTAG00000002296 | ENSBTAG00000003001 |  |  |
| ENSBTAG00000002298 | ENSBTAG00000003002 |  |  |
| ENSBTAG00000002300 | ENSBTAG00000003012 |  |  |
| ENSBTAG00000002303 | ENSBTAG00000003015 |  |  |
| ENSBTAG00000002306 | ENSBTAG00000003016 |  |  |
| ENSBTAG00000002311 | ENSBTAG00000003018 |  |  |
| ENSBTAG00000002313 | ENSBTAG00000003021 |  |  |
| ENSBTAG00000002315 | ENSBTAG00000003022 |  |  |
| ENSBTAG00000002319 | ENSBTAG00000003025 |  |  |
| ENSBTAG00000002321 | ENSBTAG00000003027 |  |  |
| ENSBTAG00000002323 | ENSBTAG00000003033 |  |  |
| ENSBTAG00000002327 | ENSBTAG00000003034 |  |  |
| ENSBTAG00000002329 | ENSBTAG00000003035 |  |  |
| ENSBTAG00000002331 | ENSBTAG00000003036 |  |  |
| ENSBTAG00000002332 | ENSBTAG00000003040 |  |  |
| ENSBTAG00000002333 | ENSBTAG00000003044 |  |  |
| ENSBTAG00000002335 | ENSBTAG00000003058 |  |  |
| ENSBTAG00000002341 | ENSBTAG00000003059 |  |  |
| ENSBTAG00000002344 | ENSBTAG00000003061 |  |  |
| ENSBTAG00000002349 | ENSBTAG00000003062 |  |  |
| ENSBTAG00000002350 | ENSBTAG00000003063 |  |  |
| ENSBTAG00000002352 | ENSBTAG00000003064 |  |  |
| ENSBTAG00000002357 | ENSBTAG00000003068 |  |  |
| ENSBTAG00000002361 | ENSBTAG00000003074 |  |  |
| ENSBTAG00000002362 | ENSBTAG00000003077 |  |  |
| ENSBTAG00000002367 | ENSBTAG00000003084 |  |  |
| ENSBTAG00000002376 | ENSBTAG00000003087 |  |  |
| ENSBTAG00000002378 | ENSBTAG00000003089 |  |  |
| ENSBTAG00000002381 | ENSBTAG00000003094 |  |  |
| ENSBTAG00000002382 | ENSBTAG00000003097 |  |  |
| ENSBTAG00000002389 | ENSBTAG00000003098 |  |  |
| ENSBTAG00000002390 | ENSBTAG00000003115 |  |  |
| ENSBTAG00000002391 | ENSBTAG00000003116 |  |  |
| ENSBTAG00000002392 | ENSBTAG00000003124 |  |  |
| ENSBTAG00000002393 | ENSBTAG00000003137 |  |  |
| ENSBTAG00000002394 | ENSBTAG00000003143 |  |  |
| ENSBTAG00000002395 | ENSBTAG00000003152 |  |  |
| ENSBTAG00000002402 | ENSBTAG00000003160 |  |  |
| ENSBTAG00000002404 | ENSBTAG00000003161 |  |  |
| ENSBTAG00000002411 | ENSBTAG00000003162 |  |  |
| ENSBTAG00000002413 | ENSBTAG00000003165 |  |  |
| ENSBTAG00000002414 | ENSBTAG00000003166 |  |  |
| ENSBTAG00000002415 | ENSBTAG00000003172 |  |  |
| ENSBTAG00000002417 | ENSBTAG00000003176 |  |  |
| ENSBTAG00000002418 | ENSBTAG00000003177 |  |  |
| ENSBTAG00000002419 | ENSBTAG00000003183 |  |  |
| ENSBTAG00000002425 | ENSBTAG00000003185 |  |  |
| ENSBTAG00000002430 | ENSBTAG00000003186 |  |  |
| ENSBTAG00000002434 | ENSBTAG00000003191 |  |  |
| ENSBTAG00000002435 | ENSBTAG00000003192 |  |  |
| ENSBTAG00000002440 | ENSBTAG00000003199 |  |  |
| ENSBTAG00000002448 | ENSBTAG00000003201 |  |  |
| ENSBTAG00000002452 | ENSBTAG00000003208 |  |  |
| ENSBTAG00000002454 | ENSBTAG00000003209 |  |  |
| ENSBTAG00000002455 | ENSBTAG00000003212 |  |  |
| ENSBTAG00000002457 | ENSBTAG00000003215 |  |  |
| ENSBTAG00000002458 | ENSBTAG00000003218 |  |  |
| ENSBTAG00000002460 | ENSBTAG00000003225 |  |  |
| ENSBTAG00000002469 | ENSBTAG00000003232 |  |  |
| ENSBTAG00000002472 | ENSBTAG00000003238 |  |  |
| ENSBTAG00000002473 | ENSBTAG00000003240 |  |  |
| ENSBTAG00000002474 | ENSBTAG00000003263 |  |  |
| ENSBTAG00000002475 | ENSBTAG00000003265 |  |  |
| ENSBTAG00000002476 | ENSBTAG00000003276 |  |  |
| ENSBTAG00000002477 | ENSBTAG00000003282 |  |  |
| ENSBTAG00000002479 | ENSBTAG00000003291 |  |  |
| ENSBTAG00000002480 | ENSBTAG00000003296 |  |  |
| ENSBTAG00000002483 | ENSBTAG00000003297 |  |  |
| ENSBTAG00000002484 | ENSBTAG00000003298 |  |  |
| ENSBTAG00000002485 | ENSBTAG00000003299 |  |  |
| ENSBTAG00000002487 | ENSBTAG00000003300 |  |  |
| ENSBTAG00000002489 | ENSBTAG00000003304 |  |  |
| ENSBTAG00000002490 | ENSBTAG00000003307 |  |  |
| ENSBTAG00000002492 | ENSBTAG00000003312 |  |  |
| ENSBTAG00000002493 | ENSBTAG00000003314 |  |  |
| ENSBTAG00000002500 | ENSBTAG00000003315 |  |  |
| ENSBTAG00000002504 | ENSBTAG00000003316 |  |  |
| ENSBTAG00000002508 | ENSBTAG00000003319 |  |  |
| ENSBTAG00000002512 | ENSBTAG00000003323 |  |  |
| ENSBTAG00000002516 | ENSBTAG00000003326 |  |  |
| ENSBTAG00000002522 | ENSBTAG00000003327 |  |  |
| ENSBTAG00000002525 | ENSBTAG00000003334 |  |  |
| ENSBTAG00000002526 | ENSBTAG00000003335 |  |  |
| ENSBTAG00000002531 | ENSBTAG00000003342 |  |  |
| ENSBTAG00000002534 | ENSBTAG00000003349 |  |  |
| ENSBTAG00000002542 | ENSBTAG00000003353 |  |  |
| ENSBTAG00000002551 | ENSBTAG00000003358 |  |  |
| ENSBTAG00000002554 | ENSBTAG00000003359 |  |  |
| ENSBTAG00000002557 | ENSBTAG00000003365 |  |  |
| ENSBTAG00000002561 | ENSBTAG00000003371 |  |  |
| ENSBTAG00000002562 | ENSBTAG00000003375 |  |  |
| ENSBTAG00000002563 | ENSBTAG00000003378 |  |  |
| ENSBTAG00000002566 | ENSBTAG00000003381 |  |  |
| ENSBTAG00000002568 | ENSBTAG00000003386 |  |  |
| ENSBTAG00000002570 | ENSBTAG00000003392 |  |  |
| ENSBTAG00000002573 | ENSBTAG00000003397 |  |  |
| ENSBTAG00000002575 | ENSBTAG00000003399 |  |  |
| ENSBTAG00000002578 | ENSBTAG00000003415 |  |  |
| ENSBTAG00000002581 | ENSBTAG00000003418 |  |  |
| ENSBTAG00000002582 | ENSBTAG00000003419 |  |  |
| ENSBTAG00000002583 | ENSBTAG00000003423 |  |  |
| ENSBTAG00000002585 | ENSBTAG00000003425 |  |  |
| ENSBTAG00000002586 | ENSBTAG00000003430 |  |  |
| ENSBTAG00000002590 | ENSBTAG00000003434 |  |  |
| ENSBTAG00000002591 | ENSBTAG00000003438 |  |  |
| ENSBTAG00000002593 | ENSBTAG00000003439 |  |  |
| ENSBTAG00000002596 | ENSBTAG00000003440 |  |  |
| ENSBTAG00000002599 | ENSBTAG00000003443 |  |  |
| ENSBTAG00000002605 | ENSBTAG00000003444 |  |  |
| ENSBTAG00000002609 | ENSBTAG00000003445 |  |  |
| ENSBTAG00000002610 | ENSBTAG00000003454 |  |  |
| ENSBTAG00000002615 | ENSBTAG00000003455 |  |  |
| ENSBTAG00000002623 | ENSBTAG00000003458 |  |  |
| ENSBTAG00000002624 | ENSBTAG00000003462 |  |  |
| ENSBTAG00000002625 | ENSBTAG00000003467 |  |  |
| ENSBTAG00000002628 | ENSBTAG00000003469 |  |  |
| ENSBTAG00000002632 | ENSBTAG00000003470 |  |  |
| ENSBTAG00000002634 | ENSBTAG00000003472 |  |  |
| ENSBTAG00000002641 | ENSBTAG00000003474 |  |  |
| ENSBTAG00000002642 | ENSBTAG00000003476 |  |  |
| ENSBTAG00000002643 | ENSBTAG00000003484 |  |  |
| ENSBTAG00000002651 | ENSBTAG00000003490 |  |  |
| ENSBTAG00000002654 | ENSBTAG00000003499 |  |  |
| ENSBTAG00000002655 | ENSBTAG00000003501 |  |  |
| ENSBTAG00000002657 | ENSBTAG00000003503 |  |  |
| ENSBTAG00000002658 | ENSBTAG00000003504 |  |  |
| ENSBTAG00000002676 | ENSBTAG00000003505 |  |  |
| ENSBTAG00000002678 | ENSBTAG00000003506 |  |  |
| ENSBTAG00000002683 | ENSBTAG00000003511 |  |  |
| ENSBTAG00000002685 | ENSBTAG00000003515 |  |  |
| ENSBTAG00000002689 | ENSBTAG00000003516 |  |  |
| ENSBTAG00000002691 | ENSBTAG00000003525 |  |  |
| ENSBTAG00000002693 | ENSBTAG00000003527 |  |  |
| ENSBTAG00000002697 | ENSBTAG00000003528 |  |  |
| ENSBTAG00000002699 | ENSBTAG00000003531 |  |  |
| ENSBTAG00000002700 | ENSBTAG00000003532 |  |  |
| ENSBTAG00000002701 | ENSBTAG00000003536 |  |  |
| ENSBTAG00000002703 | ENSBTAG00000003547 |  |  |
| ENSBTAG00000002704 | ENSBTAG00000003550 |  |  |
| ENSBTAG00000002707 | ENSBTAG00000003557 |  |  |
| ENSBTAG00000002714 | ENSBTAG00000003565 |  |  |
| ENSBTAG00000002716 | ENSBTAG00000003569 |  |  |
| ENSBTAG00000002717 | ENSBTAG00000003570 |  |  |
| ENSBTAG00000002720 | ENSBTAG00000003576 |  |  |
| ENSBTAG00000002721 | ENSBTAG00000003578 |  |  |
| ENSBTAG00000002724 | ENSBTAG00000003579 |  |  |
| ENSBTAG00000002725 | ENSBTAG00000003585 |  |  |
| ENSBTAG00000002726 | ENSBTAG00000003586 |  |  |
| ENSBTAG00000002727 | ENSBTAG00000003588 |  |  |
| ENSBTAG00000002730 | ENSBTAG00000003589 |  |  |
| ENSBTAG00000002733 | ENSBTAG00000003600 |  |  |
| ENSBTAG00000002735 | ENSBTAG00000003604 |  |  |
| ENSBTAG00000002736 | ENSBTAG00000003610 |  |  |
| ENSBTAG00000002738 | ENSBTAG00000003619 |  |  |
| ENSBTAG00000002746 | ENSBTAG00000003625 |  |  |
| ENSBTAG00000002748 | ENSBTAG00000003632 |  |  |
| ENSBTAG00000002750 | ENSBTAG00000003636 |  |  |
| ENSBTAG00000002758 | ENSBTAG00000003638 |  |  |
| ENSBTAG00000002763 | ENSBTAG00000003639 |  |  |
| ENSBTAG00000002768 | ENSBTAG00000003649 |  |  |
| ENSBTAG00000002769 | ENSBTAG00000003650 |  |  |
| ENSBTAG00000002770 | ENSBTAG00000003652 |  |  |
| ENSBTAG00000002772 | ENSBTAG00000003653 |  |  |
| ENSBTAG00000002774 | ENSBTAG00000003665 |  |  |
| ENSBTAG00000002779 | ENSBTAG00000003675 |  |  |
| ENSBTAG00000002786 | ENSBTAG00000003679 |  |  |
| ENSBTAG00000002791 | ENSBTAG00000003687 |  |  |
| ENSBTAG00000002792 | ENSBTAG00000003690 |  |  |
| ENSBTAG00000002798 | ENSBTAG00000003695 |  |  |
| ENSBTAG00000002799 | ENSBTAG00000003697 |  |  |
| ENSBTAG00000002804 | ENSBTAG00000003699 |  |  |
| ENSBTAG00000002810 | ENSBTAG00000003701 |  |  |
| ENSBTAG00000002813 | ENSBTAG00000003702 |  |  |
| ENSBTAG00000002816 | ENSBTAG00000003707 |  |  |
| ENSBTAG00000002817 | ENSBTAG00000003708 |  |  |
| ENSBTAG00000002823 | ENSBTAG00000003709 |  |  |
| ENSBTAG00000002827 | ENSBTAG00000003710 |  |  |
| ENSBTAG00000002828 | ENSBTAG00000003711 |  |  |
| ENSBTAG00000002830 | ENSBTAG00000003721 |  |  |
| ENSBTAG00000002833 | ENSBTAG00000003726 |  |  |
| ENSBTAG00000002834 | ENSBTAG00000003728 |  |  |
| ENSBTAG00000002835 | ENSBTAG00000003733 |  |  |
| ENSBTAG00000002844 | ENSBTAG00000003737 |  |  |
| ENSBTAG00000002845 | ENSBTAG00000003745 |  |  |
| ENSBTAG00000002846 | ENSBTAG00000003746 |  |  |
| ENSBTAG00000002847 | ENSBTAG00000003748 |  |  |
| ENSBTAG00000002848 | ENSBTAG00000003749 |  |  |
| ENSBTAG00000002849 | ENSBTAG00000003752 |  |  |
| ENSBTAG00000002854 | ENSBTAG00000003762 |  |  |
| ENSBTAG00000002856 | ENSBTAG00000003764 |  |  |
| ENSBTAG00000002858 | ENSBTAG00000003766 |  |  |
| ENSBTAG00000002859 | ENSBTAG00000003773 |  |  |
| ENSBTAG00000002863 | ENSBTAG00000003775 |  |  |
| ENSBTAG00000002868 | ENSBTAG00000003779 |  |  |
| ENSBTAG00000002869 | ENSBTAG00000003791 |  |  |
| ENSBTAG00000002871 | ENSBTAG00000003794 |  |  |
| ENSBTAG00000002874 | ENSBTAG00000003807 |  |  |
| ENSBTAG00000002878 | ENSBTAG00000003809 |  |  |
| ENSBTAG00000002879 | ENSBTAG00000003810 |  |  |
| ENSBTAG00000002880 | ENSBTAG00000003819 |  |  |
| ENSBTAG00000002883 | ENSBTAG00000003826 |  |  |
| ENSBTAG00000002885 | ENSBTAG00000003827 |  |  |
| ENSBTAG00000002887 | ENSBTAG00000003836 |  |  |
| ENSBTAG00000002891 | ENSBTAG00000003837 |  |  |
| ENSBTAG00000002896 | ENSBTAG00000003840 |  |  |
| ENSBTAG00000002898 | ENSBTAG00000003842 |  |  |
| ENSBTAG00000002907 | ENSBTAG00000003851 |  |  |
| ENSBTAG00000002908 | ENSBTAG00000003855 |  |  |
| ENSBTAG00000002914 | ENSBTAG00000003864 |  |  |
| ENSBTAG00000002917 | ENSBTAG00000003865 |  |  |
| ENSBTAG00000002918 | ENSBTAG00000003866 |  |  |
| ENSBTAG00000002921 | ENSBTAG00000003871 |  |  |
| ENSBTAG00000002923 | ENSBTAG00000003872 |  |  |
| ENSBTAG00000002935 | ENSBTAG00000003877 |  |  |
| ENSBTAG00000002940 | ENSBTAG00000003878 |  |  |
| ENSBTAG00000002941 | ENSBTAG00000003882 |  |  |
| ENSBTAG00000002942 | ENSBTAG00000003884 |  |  |
| ENSBTAG00000002947 | ENSBTAG00000003887 |  |  |
| ENSBTAG00000002952 | ENSBTAG00000003894 |  |  |
| ENSBTAG00000002956 | ENSBTAG00000003897 |  |  |
| ENSBTAG00000002960 | ENSBTAG00000003898 |  |  |
| ENSBTAG00000002962 | ENSBTAG00000003904 |  |  |
| ENSBTAG00000002966 | ENSBTAG00000003906 |  |  |
| ENSBTAG00000002970 | ENSBTAG00000003907 |  |  |
| ENSBTAG00000002971 | ENSBTAG00000003916 |  |  |
| ENSBTAG00000002973 | ENSBTAG00000003919 |  |  |
| ENSBTAG00000002974 | ENSBTAG00000003922 |  |  |
| ENSBTAG00000002977 | ENSBTAG00000003925 |  |  |
| ENSBTAG00000002980 | ENSBTAG00000003929 |  |  |
| ENSBTAG00000002981 | ENSBTAG00000003934 |  |  |
| ENSBTAG00000002985 | ENSBTAG00000003935 |  |  |
| ENSBTAG00000002988 | ENSBTAG00000003936 |  |  |
| ENSBTAG00000002993 | ENSBTAG00000003938 |  |  |
| ENSBTAG00000002997 | ENSBTAG00000003941 |  |  |
| ENSBTAG00000003001 | ENSBTAG00000003943 |  |  |
| ENSBTAG00000003002 | ENSBTAG00000003946 |  |  |
| ENSBTAG00000003012 | ENSBTAG00000003952 |  |  |
| ENSBTAG00000003014 | ENSBTAG00000003954 |  |  |
| ENSBTAG00000003015 | ENSBTAG00000003955 |  |  |
| ENSBTAG00000003016 | ENSBTAG00000003956 |  |  |
| ENSBTAG00000003018 | ENSBTAG00000003958 |  |  |
| ENSBTAG00000003021 | ENSBTAG00000003960 |  |  |
| ENSBTAG00000003025 | ENSBTAG00000003961 |  |  |
| ENSBTAG00000003027 | ENSBTAG00000003965 |  |  |
| ENSBTAG00000003033 | ENSBTAG00000003966 |  |  |
| ENSBTAG00000003034 | ENSBTAG00000003977 |  |  |
| ENSBTAG00000003035 | ENSBTAG00000003983 |  |  |
| ENSBTAG00000003036 | ENSBTAG00000003986 |  |  |
| ENSBTAG00000003038 | ENSBTAG00000003989 |  |  |
| ENSBTAG00000003039 | ENSBTAG00000003990 |  |  |
| ENSBTAG00000003040 | ENSBTAG00000003994 |  |  |
| ENSBTAG00000003043 | ENSBTAG00000003997 |  |  |
| ENSBTAG00000003044 | ENSBTAG00000004003 |  |  |
| ENSBTAG00000003045 | ENSBTAG00000004004 |  |  |
| ENSBTAG00000003052 | ENSBTAG00000004008 |  |  |
| ENSBTAG00000003058 | ENSBTAG00000004009 |  |  |
| ENSBTAG00000003059 | ENSBTAG00000004018 |  |  |
| ENSBTAG00000003061 | ENSBTAG00000004021 |  |  |
| ENSBTAG00000003062 | ENSBTAG00000004023 |  |  |
| ENSBTAG00000003063 | ENSBTAG00000004024 |  |  |
| ENSBTAG00000003064 | ENSBTAG00000004028 |  |  |
| ENSBTAG00000003067 | ENSBTAG00000004036 |  |  |
| ENSBTAG00000003068 | ENSBTAG00000004037 |  |  |
| ENSBTAG00000003069 | ENSBTAG00000004038 |  |  |
| ENSBTAG00000003072 | ENSBTAG00000004040 |  |  |
| ENSBTAG00000003074 | ENSBTAG00000004041 |  |  |
| ENSBTAG00000003077 | ENSBTAG00000004043 |  |  |
| ENSBTAG00000003083 | ENSBTAG00000004048 |  |  |
| ENSBTAG00000003084 | ENSBTAG00000004051 |  |  |
| ENSBTAG00000003087 | ENSBTAG00000004059 |  |  |
| ENSBTAG00000003089 | ENSBTAG00000004063 |  |  |
| ENSBTAG00000003094 | ENSBTAG00000004072 |  |  |
| ENSBTAG00000003097 | ENSBTAG00000004073 |  |  |
| ENSBTAG00000003098 | ENSBTAG00000004077 |  |  |
| ENSBTAG00000003107 | ENSBTAG00000004088 |  |  |
| ENSBTAG00000003109 | ENSBTAG00000004094 |  |  |
| ENSBTAG00000003115 | ENSBTAG00000004098 |  |  |
| ENSBTAG00000003116 | ENSBTAG00000004099 |  |  |
| ENSBTAG00000003124 | ENSBTAG00000004114 |  |  |
| ENSBTAG00000003126 | ENSBTAG00000004115 |  |  |
| ENSBTAG00000003134 | ENSBTAG00000004117 |  |  |
| ENSBTAG00000003143 | ENSBTAG00000004124 |  |  |
| ENSBTAG00000003147 | ENSBTAG00000004129 |  |  |
| ENSBTAG00000003151 | ENSBTAG00000004131 |  |  |
| ENSBTAG00000003152 | ENSBTAG00000004135 |  |  |
| ENSBTAG00000003160 | ENSBTAG00000004136 |  |  |
| ENSBTAG00000003161 | ENSBTAG00000004145 |  |  |
| ENSBTAG00000003162 | ENSBTAG00000004147 |  |  |
| ENSBTAG00000003165 | ENSBTAG00000004154 |  |  |
| ENSBTAG00000003166 | ENSBTAG00000004161 |  |  |
| ENSBTAG00000003168 | ENSBTAG00000004165 |  |  |
| ENSBTAG00000003172 | ENSBTAG00000004168 |  |  |
| ENSBTAG00000003174 | ENSBTAG00000004171 |  |  |
| ENSBTAG00000003176 | ENSBTAG00000004173 |  |  |
| ENSBTAG00000003177 | ENSBTAG00000004178 |  |  |
| ENSBTAG00000003183 | ENSBTAG00000004179 |  |  |
| ENSBTAG00000003185 | ENSBTAG00000004188 |  |  |
| ENSBTAG00000003186 | ENSBTAG00000004190 |  |  |
| ENSBTAG00000003189 | ENSBTAG00000004193 |  |  |
| ENSBTAG00000003191 | ENSBTAG00000004199 |  |  |
| ENSBTAG00000003192 | ENSBTAG00000004200 |  |  |
| ENSBTAG00000003199 | ENSBTAG00000004207 |  |  |
| ENSBTAG00000003201 | ENSBTAG00000004211 |  |  |
| ENSBTAG00000003205 | ENSBTAG00000004218 |  |  |
| ENSBTAG00000003208 | ENSBTAG00000004221 |  |  |
| ENSBTAG00000003209 | ENSBTAG00000004223 |  |  |
| ENSBTAG00000003212 | ENSBTAG00000004230 |  |  |
| ENSBTAG00000003215 | ENSBTAG00000004237 |  |  |
| ENSBTAG00000003218 | ENSBTAG00000004240 |  |  |
| ENSBTAG00000003225 | ENSBTAG00000004243 |  |  |
| ENSBTAG00000003232 | ENSBTAG00000004257 |  |  |
| ENSBTAG00000003238 | ENSBTAG00000004258 |  |  |
| ENSBTAG00000003240 | ENSBTAG00000004259 |  |  |
| ENSBTAG00000003242 | ENSBTAG00000004261 |  |  |
| ENSBTAG00000003253 | ENSBTAG00000004263 |  |  |
| ENSBTAG00000003256 | ENSBTAG00000004269 |  |  |
| ENSBTAG00000003257 | ENSBTAG00000004273 |  |  |
| ENSBTAG00000003263 | ENSBTAG00000004275 |  |  |
| ENSBTAG00000003265 | ENSBTAG00000004277 |  |  |
| ENSBTAG00000003268 | ENSBTAG00000004278 |  |  |
| ENSBTAG00000003276 | ENSBTAG00000004279 |  |  |
| ENSBTAG00000003280 | ENSBTAG00000004283 |  |  |
| ENSBTAG00000003282 | ENSBTAG00000004284 |  |  |
| ENSBTAG00000003288 | ENSBTAG00000004287 |  |  |
| ENSBTAG00000003291 | ENSBTAG00000004288 |  |  |
| ENSBTAG00000003295 | ENSBTAG00000004290 |  |  |
| ENSBTAG00000003296 | ENSBTAG00000004291 |  |  |
| ENSBTAG00000003297 | ENSBTAG00000004292 |  |  |
| ENSBTAG00000003298 | ENSBTAG00000004293 |  |  |
| ENSBTAG00000003299 | ENSBTAG00000004294 |  |  |
| ENSBTAG00000003300 | ENSBTAG00000004295 |  |  |
| ENSBTAG00000003304 | ENSBTAG00000004305 |  |  |
| ENSBTAG00000003307 | ENSBTAG00000004307 |  |  |
| ENSBTAG00000003312 | ENSBTAG00000004310 |  |  |
| ENSBTAG00000003313 | ENSBTAG00000004316 |  |  |
| ENSBTAG00000003314 | ENSBTAG00000004321 |  |  |
| ENSBTAG00000003315 | ENSBTAG00000004322 |  |  |
| ENSBTAG00000003316 | ENSBTAG00000004328 |  |  |
| ENSBTAG00000003319 | ENSBTAG00000004331 |  |  |
| ENSBTAG00000003323 | ENSBTAG00000004333 |  |  |
| ENSBTAG00000003326 | ENSBTAG00000004339 |  |  |
| ENSBTAG00000003327 | ENSBTAG00000004344 |  |  |
| ENSBTAG00000003332 | ENSBTAG00000004347 |  |  |
| ENSBTAG00000003334 | ENSBTAG00000004349 |  |  |
| ENSBTAG00000003335 | ENSBTAG00000004353 |  |  |
| ENSBTAG00000003336 | ENSBTAG00000004356 |  |  |
| ENSBTAG00000003342 | ENSBTAG00000004358 |  |  |
| ENSBTAG00000003349 | ENSBTAG00000004364 |  |  |
| ENSBTAG00000003353 | ENSBTAG00000004371 |  |  |
| ENSBTAG00000003358 | ENSBTAG00000004376 |  |  |
| ENSBTAG00000003359 | ENSBTAG00000004377 |  |  |
| ENSBTAG00000003362 | ENSBTAG00000004379 |  |  |
| ENSBTAG00000003364 | ENSBTAG00000004383 |  |  |
| ENSBTAG00000003365 | ENSBTAG00000004384 |  |  |
| ENSBTAG00000003371 | ENSBTAG00000004387 |  |  |
| ENSBTAG00000003375 | ENSBTAG00000004392 |  |  |
| ENSBTAG00000003378 | ENSBTAG00000004394 |  |  |
| ENSBTAG00000003381 | ENSBTAG00000004402 |  |  |
| ENSBTAG00000003386 | ENSBTAG00000004403 |  |  |
| ENSBTAG00000003388 | ENSBTAG00000004407 |  |  |
| ENSBTAG00000003390 | ENSBTAG00000004409 |  |  |
| ENSBTAG00000003397 | ENSBTAG00000004411 |  |  |
| ENSBTAG00000003399 | ENSBTAG00000004421 |  |  |
| ENSBTAG00000003405 | ENSBTAG00000004422 |  |  |
| ENSBTAG00000003409 | ENSBTAG00000004423 |  |  |
| ENSBTAG00000003410 | ENSBTAG00000004428 |  |  |
| ENSBTAG00000003415 | ENSBTAG00000004430 |  |  |
| ENSBTAG00000003417 | ENSBTAG00000004432 |  |  |
| ENSBTAG00000003418 | ENSBTAG00000004436 |  |  |
| ENSBTAG00000003419 | ENSBTAG00000004442 |  |  |
| ENSBTAG00000003422 | ENSBTAG00000004449 |  |  |
| ENSBTAG00000003423 | ENSBTAG00000004457 |  |  |
| ENSBTAG00000003424 | ENSBTAG00000004459 |  |  |
| ENSBTAG00000003425 | ENSBTAG00000004461 |  |  |
| ENSBTAG00000003430 | ENSBTAG00000004471 |  |  |
| ENSBTAG00000003434 | ENSBTAG00000004489 |  |  |
| ENSBTAG00000003438 | ENSBTAG00000004492 |  |  |
| ENSBTAG00000003439 | ENSBTAG00000004494 |  |  |
| ENSBTAG00000003440 | ENSBTAG00000004495 |  |  |
| ENSBTAG00000003443 | ENSBTAG00000004496 |  |  |
| ENSBTAG00000003444 | ENSBTAG00000004499 |  |  |
| ENSBTAG00000003445 | ENSBTAG00000004502 |  |  |
| ENSBTAG00000003450 | ENSBTAG00000004505 |  |  |
| ENSBTAG00000003454 | ENSBTAG00000004508 |  |  |
| ENSBTAG00000003455 | ENSBTAG00000004511 |  |  |
| ENSBTAG00000003458 | ENSBTAG00000004541 |  |  |
| ENSBTAG00000003462 | ENSBTAG00000004542 |  |  |
| ENSBTAG00000003467 | ENSBTAG00000004549 |  |  |
| ENSBTAG00000003469 | ENSBTAG00000004553 |  |  |
| ENSBTAG00000003470 | ENSBTAG00000004554 |  |  |
| ENSBTAG00000003474 | ENSBTAG00000004556 |  |  |
| ENSBTAG00000003476 | ENSBTAG00000004557 |  |  |
| ENSBTAG00000003484 | ENSBTAG00000004571 |  |  |
| ENSBTAG00000003490 | ENSBTAG00000004572 |  |  |
| ENSBTAG00000003499 | ENSBTAG00000004574 |  |  |
| ENSBTAG00000003500 | ENSBTAG00000004581 |  |  |
| ENSBTAG00000003501 | ENSBTAG00000004587 |  |  |
| ENSBTAG00000003503 | ENSBTAG00000004588 |  |  |
| ENSBTAG00000003504 | ENSBTAG00000004590 |  |  |
| ENSBTAG00000003505 | ENSBTAG00000004593 |  |  |
| ENSBTAG00000003506 | ENSBTAG00000004594 |  |  |
| ENSBTAG00000003511 | ENSBTAG00000004595 |  |  |
| ENSBTAG00000003512 | ENSBTAG00000004597 |  |  |
| ENSBTAG00000003514 | ENSBTAG00000004598 |  |  |
| ENSBTAG00000003516 | ENSBTAG00000004599 |  |  |
| ENSBTAG00000003525 | ENSBTAG00000004603 |  |  |
| ENSBTAG00000003527 | ENSBTAG00000004604 |  |  |
| ENSBTAG00000003528 | ENSBTAG00000004608 |  |  |
| ENSBTAG00000003530 | ENSBTAG00000004613 |  |  |
| ENSBTAG00000003531 | ENSBTAG00000004622 |  |  |
| ENSBTAG00000003532 | ENSBTAG00000004636 |  |  |
| ENSBTAG00000003535 | ENSBTAG00000004639 |  |  |
| ENSBTAG00000003536 | ENSBTAG00000004641 |  |  |
| ENSBTAG00000003539 | ENSBTAG00000004643 |  |  |
| ENSBTAG00000003544 | ENSBTAG00000004647 |  |  |
| ENSBTAG00000003547 | ENSBTAG00000004651 |  |  |
| ENSBTAG00000003548 | ENSBTAG00000004652 |  |  |
| ENSBTAG00000003550 | ENSBTAG00000004659 |  |  |
| ENSBTAG00000003556 | ENSBTAG00000004660 |  |  |
| ENSBTAG00000003557 | ENSBTAG00000004663 |  |  |
| ENSBTAG00000003565 | ENSBTAG00000004664 |  |  |
| ENSBTAG00000003566 | ENSBTAG00000004672 |  |  |
| ENSBTAG00000003569 | ENSBTAG00000004676 |  |  |
| ENSBTAG00000003570 | ENSBTAG00000004679 |  |  |
| ENSBTAG00000003572 | ENSBTAG00000004688 |  |  |
| ENSBTAG00000003576 | ENSBTAG00000004694 |  |  |
| ENSBTAG00000003577 | ENSBTAG00000004695 |  |  |
| ENSBTAG00000003578 | ENSBTAG00000004706 |  |  |
| ENSBTAG00000003579 | ENSBTAG00000004709 |  |  |
| ENSBTAG00000003580 | ENSBTAG00000004710 |  |  |
| ENSBTAG00000003585 | ENSBTAG00000004715 |  |  |
| ENSBTAG00000003586 | ENSBTAG00000004718 |  |  |
| ENSBTAG00000003588 | ENSBTAG00000004722 |  |  |
| ENSBTAG00000003589 | ENSBTAG00000004725 |  |  |
| ENSBTAG00000003595 | ENSBTAG00000004727 |  |  |
| ENSBTAG00000003598 | ENSBTAG00000004732 |  |  |
| ENSBTAG00000003600 | ENSBTAG00000004738 |  |  |
| ENSBTAG00000003607 | ENSBTAG00000004739 |  |  |
| ENSBTAG00000003610 | ENSBTAG00000004745 |  |  |
| ENSBTAG00000003614 | ENSBTAG00000004747 |  |  |
| ENSBTAG00000003619 | ENSBTAG00000004750 |  |  |
| ENSBTAG00000003625 | ENSBTAG00000004751 |  |  |
| ENSBTAG00000003629 | ENSBTAG00000004754 |  |  |
| ENSBTAG00000003632 | ENSBTAG00000004755 |  |  |
| ENSBTAG00000003634 | ENSBTAG00000004767 |  |  |
| ENSBTAG00000003635 | ENSBTAG00000004769 |  |  |
| ENSBTAG00000003636 | ENSBTAG00000004775 |  |  |
| ENSBTAG00000003638 | ENSBTAG00000004777 |  |  |
| ENSBTAG00000003639 | ENSBTAG00000004781 |  |  |
| ENSBTAG00000003642 | ENSBTAG00000004782 |  |  |
| ENSBTAG00000003644 | ENSBTAG00000004783 |  |  |
| ENSBTAG00000003649 | ENSBTAG00000004791 |  |  |
| ENSBTAG00000003650 | ENSBTAG00000004792 |  |  |
| ENSBTAG00000003652 | ENSBTAG00000004799 |  |  |
| ENSBTAG00000003653 | ENSBTAG00000004801 |  |  |
| ENSBTAG00000003658 | ENSBTAG00000004804 |  |  |
| ENSBTAG00000003665 | ENSBTAG00000004805 |  |  |
| ENSBTAG00000003675 | ENSBTAG00000004822 |  |  |
| ENSBTAG00000003679 | ENSBTAG00000004824 |  |  |
| ENSBTAG00000003684 | ENSBTAG00000004833 |  |  |
| ENSBTAG00000003687 | ENSBTAG00000004840 |  |  |
| ENSBTAG00000003690 | ENSBTAG00000004842 |  |  |
| ENSBTAG00000003691 | ENSBTAG00000004855 |  |  |
| ENSBTAG00000003692 | ENSBTAG00000004863 |  |  |
| ENSBTAG00000003695 | ENSBTAG00000004869 |  |  |
| ENSBTAG00000003697 | ENSBTAG00000004870 |  |  |
| ENSBTAG00000003700 | ENSBTAG00000004873 |  |  |
| ENSBTAG00000003701 | ENSBTAG00000004875 |  |  |
| ENSBTAG00000003702 | ENSBTAG00000004876 |  |  |
| ENSBTAG00000003707 | ENSBTAG00000004877 |  |  |
| ENSBTAG00000003708 | ENSBTAG00000004878 |  |  |
| ENSBTAG00000003709 | ENSBTAG00000004879 |  |  |
| ENSBTAG00000003710 | ENSBTAG00000004886 |  |  |
| ENSBTAG00000003711 | ENSBTAG00000004888 |  |  |
| ENSBTAG00000003712 | ENSBTAG00000004894 |  |  |
| ENSBTAG00000003721 | ENSBTAG00000004895 |  |  |
| ENSBTAG00000003726 | ENSBTAG00000004896 |  |  |
| ENSBTAG00000003728 | ENSBTAG00000004906 |  |  |
| ENSBTAG00000003733 | ENSBTAG00000004912 |  |  |
| ENSBTAG00000003737 | ENSBTAG00000004913 |  |  |
| ENSBTAG00000003740 | ENSBTAG00000004922 |  |  |
| ENSBTAG00000003741 | ENSBTAG00000004924 |  |  |
| ENSBTAG00000003745 | ENSBTAG00000004930 |  |  |
| ENSBTAG00000003746 | ENSBTAG00000004940 |  |  |
| ENSBTAG00000003748 | ENSBTAG00000004948 |  |  |
| ENSBTAG00000003749 | ENSBTAG00000004952 |  |  |
| ENSBTAG00000003752 | ENSBTAG00000004954 |  |  |
| ENSBTAG00000003754 | ENSBTAG00000004956 |  |  |
| ENSBTAG00000003758 | ENSBTAG00000004964 |  |  |
| ENSBTAG00000003759 | ENSBTAG00000004965 |  |  |
| ENSBTAG00000003762 | ENSBTAG00000004971 |  |  |
| ENSBTAG00000003764 | ENSBTAG00000004976 |  |  |
| ENSBTAG00000003766 | ENSBTAG00000004979 |  |  |
| ENSBTAG00000003773 | ENSBTAG00000004989 |  |  |
| ENSBTAG00000003775 | ENSBTAG00000004995 |  |  |
| ENSBTAG00000003779 | ENSBTAG00000004996 |  |  |
| ENSBTAG00000003786 | ENSBTAG00000004999 |  |  |
| ENSBTAG00000003791 | ENSBTAG00000005002 |  |  |
| ENSBTAG00000003794 | ENSBTAG00000005009 |  |  |
| ENSBTAG00000003798 | ENSBTAG00000005010 |  |  |
| ENSBTAG00000003807 | ENSBTAG00000005012 |  |  |
| ENSBTAG00000003809 | ENSBTAG00000005015 |  |  |
| ENSBTAG00000003810 | ENSBTAG00000005025 |  |  |
| ENSBTAG00000003815 | ENSBTAG00000005028 |  |  |
| ENSBTAG00000003819 | ENSBTAG00000005029 |  |  |
| ENSBTAG00000003820 | ENSBTAG00000005031 |  |  |
| ENSBTAG00000003825 | ENSBTAG00000005034 |  |  |
| ENSBTAG00000003826 | ENSBTAG00000005043 |  |  |
| ENSBTAG00000003827 | ENSBTAG00000005062 |  |  |
| ENSBTAG00000003829 | ENSBTAG00000005063 |  |  |
| ENSBTAG00000003832 | ENSBTAG00000005064 |  |  |
| ENSBTAG00000003833 | ENSBTAG00000005068 |  |  |
| ENSBTAG00000003836 | ENSBTAG00000005069 |  |  |
| ENSBTAG00000003837 | ENSBTAG00000005071 |  |  |
| ENSBTAG00000003840 | ENSBTAG00000005072 |  |  |
| ENSBTAG00000003842 | ENSBTAG00000005075 |  |  |
| ENSBTAG00000003845 | ENSBTAG00000005077 |  |  |
| ENSBTAG00000003851 | ENSBTAG00000005078 |  |  |
| ENSBTAG00000003855 | ENSBTAG00000005082 |  |  |
| ENSBTAG00000003863 | ENSBTAG00000005085 |  |  |
| ENSBTAG00000003864 | ENSBTAG00000005086 |  |  |
| ENSBTAG00000003865 | ENSBTAG00000005089 |  |  |
| ENSBTAG00000003866 | ENSBTAG00000005090 |  |  |
| ENSBTAG00000003871 | ENSBTAG00000005092 |  |  |
| ENSBTAG00000003872 | ENSBTAG00000005102 |  |  |
| ENSBTAG00000003877 | ENSBTAG00000005105 |  |  |
| ENSBTAG00000003878 | ENSBTAG00000005107 |  |  |
| ENSBTAG00000003880 | ENSBTAG00000005110 |  |  |
| ENSBTAG00000003882 | ENSBTAG00000005111 |  |  |
| ENSBTAG00000003884 | ENSBTAG00000005115 |  |  |
| ENSBTAG00000003885 | ENSBTAG00000005120 |  |  |
| ENSBTAG00000003886 | ENSBTAG00000005122 |  |  |
| ENSBTAG00000003887 | ENSBTAG00000005133 |  |  |
| ENSBTAG00000003891 | ENSBTAG00000005136 |  |  |
| ENSBTAG00000003894 | ENSBTAG00000005144 |  |  |
| ENSBTAG00000003897 | ENSBTAG00000005146 |  |  |
| ENSBTAG00000003898 | ENSBTAG00000005158 |  |  |
| ENSBTAG00000003902 | ENSBTAG00000005160 |  |  |
| ENSBTAG00000003904 | ENSBTAG00000005161 |  |  |
| ENSBTAG00000003906 | ENSBTAG00000005166 |  |  |
| ENSBTAG00000003907 | ENSBTAG00000005174 |  |  |
| ENSBTAG00000003914 | ENSBTAG00000005181 |  |  |
| ENSBTAG00000003916 | ENSBTAG00000005183 |  |  |
| ENSBTAG00000003919 | ENSBTAG00000005186 |  |  |
| ENSBTAG00000003920 | ENSBTAG00000005191 |  |  |
| ENSBTAG00000003922 | ENSBTAG00000005193 |  |  |
| ENSBTAG00000003925 | ENSBTAG00000005195 |  |  |
| ENSBTAG00000003929 | ENSBTAG00000005197 |  |  |
| ENSBTAG00000003934 | ENSBTAG00000005205 |  |  |
| ENSBTAG00000003935 | ENSBTAG00000005206 |  |  |
| ENSBTAG00000003936 | ENSBTAG00000005207 |  |  |
| ENSBTAG00000003938 | ENSBTAG00000005210 |  |  |
| ENSBTAG00000003941 | ENSBTAG00000005211 |  |  |
| ENSBTAG00000003943 | ENSBTAG00000005212 |  |  |
| ENSBTAG00000003946 | ENSBTAG00000005214 |  |  |
| ENSBTAG00000003952 | ENSBTAG00000005225 |  |  |
| ENSBTAG00000003954 | ENSBTAG00000005227 |  |  |
| ENSBTAG00000003955 | ENSBTAG00000005230 |  |  |
| ENSBTAG00000003956 | ENSBTAG00000005231 |  |  |
| ENSBTAG00000003958 | ENSBTAG00000005234 |  |  |
| ENSBTAG00000003959 | ENSBTAG00000005235 |  |  |
| ENSBTAG00000003960 | ENSBTAG00000005236 |  |  |
| ENSBTAG00000003961 | ENSBTAG00000005246 |  |  |
| ENSBTAG00000003965 | ENSBTAG00000005249 |  |  |
| ENSBTAG00000003966 | ENSBTAG00000005252 |  |  |
| ENSBTAG00000003967 | ENSBTAG00000005257 |  |  |
| ENSBTAG00000003975 | ENSBTAG00000005259 |  |  |
| ENSBTAG00000003977 | ENSBTAG00000005263 |  |  |
| ENSBTAG00000003983 | ENSBTAG00000005268 |  |  |
| ENSBTAG00000003986 | ENSBTAG00000005269 |  |  |
| ENSBTAG00000003990 | ENSBTAG00000005272 |  |  |
| ENSBTAG00000003994 | ENSBTAG00000005275 |  |  |
| ENSBTAG00000003997 | ENSBTAG00000005278 |  |  |
| ENSBTAG00000004003 | ENSBTAG00000005280 |  |  |
| ENSBTAG00000004004 | ENSBTAG00000005288 |  |  |
| ENSBTAG00000004008 | ENSBTAG00000005289 |  |  |
| ENSBTAG00000004009 | ENSBTAG00000005290 |  |  |
| ENSBTAG00000004013 | ENSBTAG00000005293 |  |  |
| ENSBTAG00000004018 | ENSBTAG00000005294 |  |  |
| ENSBTAG00000004021 | ENSBTAG00000005299 |  |  |
| ENSBTAG00000004023 | ENSBTAG00000005300 |  |  |
| ENSBTAG00000004024 | ENSBTAG00000005305 |  |  |
| ENSBTAG00000004028 | ENSBTAG00000005308 |  |  |
| ENSBTAG00000004036 | ENSBTAG00000005309 |  |  |
| ENSBTAG00000004037 | ENSBTAG00000005311 |  |  |
| ENSBTAG00000004038 | ENSBTAG00000005316 |  |  |
| ENSBTAG00000004039 | ENSBTAG00000005318 |  |  |
| ENSBTAG00000004040 | ENSBTAG00000005321 |  |  |
| ENSBTAG00000004041 | ENSBTAG00000005328 |  |  |
| ENSBTAG00000004043 | ENSBTAG00000005337 |  |  |
| ENSBTAG00000004048 | ENSBTAG00000005339 |  |  |
| ENSBTAG00000004051 | ENSBTAG00000005345 |  |  |
| ENSBTAG00000004054 | ENSBTAG00000005353 |  |  |
| ENSBTAG00000004059 | ENSBTAG00000005354 |  |  |
| ENSBTAG00000004063 | ENSBTAG00000005355 |  |  |
| ENSBTAG00000004064 | ENSBTAG00000005356 |  |  |
| ENSBTAG00000004072 | ENSBTAG00000005359 |  |  |
| ENSBTAG00000004073 | ENSBTAG00000005367 |  |  |
| ENSBTAG00000004077 | ENSBTAG00000005371 |  |  |
| ENSBTAG00000004078 | ENSBTAG00000005376 |  |  |
| ENSBTAG00000004085 | ENSBTAG00000005377 |  |  |
| ENSBTAG00000004086 | ENSBTAG00000005380 |  |  |
| ENSBTAG00000004088 | ENSBTAG00000005384 |  |  |
| ENSBTAG00000004094 | ENSBTAG00000005385 |  |  |
| ENSBTAG00000004098 | ENSBTAG00000005386 |  |  |
| ENSBTAG00000004099 | ENSBTAG00000005397 |  |  |
| ENSBTAG00000004108 | ENSBTAG00000005403 |  |  |
| ENSBTAG00000004112 | ENSBTAG00000005414 |  |  |
| ENSBTAG00000004114 | ENSBTAG00000005416 |  |  |
| ENSBTAG00000004115 | ENSBTAG00000005419 |  |  |
| ENSBTAG00000004117 | ENSBTAG00000005424 |  |  |
| ENSBTAG00000004118 | ENSBTAG00000005425 |  |  |
| ENSBTAG00000004120 | ENSBTAG00000005427 |  |  |
| ENSBTAG00000004124 | ENSBTAG00000005431 |  |  |
| ENSBTAG00000004129 | ENSBTAG00000005434 |  |  |
| ENSBTAG00000004131 | ENSBTAG00000005436 |  |  |
| ENSBTAG00000004135 | ENSBTAG00000005437 |  |  |
| ENSBTAG00000004136 | ENSBTAG00000005443 |  |  |
| ENSBTAG00000004145 | ENSBTAG00000005445 |  |  |
| ENSBTAG00000004147 | ENSBTAG00000005448 |  |  |
| ENSBTAG00000004148 | ENSBTAG00000005453 |  |  |
| ENSBTAG00000004154 | ENSBTAG00000005454 |  |  |
| ENSBTAG00000004155 | ENSBTAG00000005464 |  |  |
| ENSBTAG00000004161 | ENSBTAG00000005469 |  |  |
| ENSBTAG00000004165 | ENSBTAG00000005470 |  |  |
| ENSBTAG00000004168 | ENSBTAG00000005471 |  |  |
| ENSBTAG00000004171 | ENSBTAG00000005474 |  |  |
| ENSBTAG00000004173 | ENSBTAG00000005475 |  |  |
| ENSBTAG00000004178 | ENSBTAG00000005476 |  |  |
| ENSBTAG00000004179 | ENSBTAG00000005477 |  |  |
| ENSBTAG00000004188 | ENSBTAG00000005481 |  |  |
| ENSBTAG00000004190 | ENSBTAG00000005483 |  |  |
| ENSBTAG00000004193 | ENSBTAG00000005488 |  |  |
| ENSBTAG00000004196 | ENSBTAG00000005492 |  |  |
| ENSBTAG00000004199 | ENSBTAG00000005493 |  |  |
| ENSBTAG00000004200 | ENSBTAG00000005496 |  |  |
| ENSBTAG00000004203 | ENSBTAG00000005497 |  |  |
| ENSBTAG00000004207 | ENSBTAG00000005498 |  |  |
| ENSBTAG00000004208 | ENSBTAG00000005503 |  |  |
| ENSBTAG00000004211 | ENSBTAG00000005514 |  |  |
| ENSBTAG00000004215 | ENSBTAG00000005519 |  |  |
| ENSBTAG00000004218 | ENSBTAG00000005524 |  |  |
| ENSBTAG00000004221 | ENSBTAG00000005526 |  |  |
| ENSBTAG00000004222 | ENSBTAG00000005532 |  |  |
| ENSBTAG00000004223 | ENSBTAG00000005533 |  |  |
| ENSBTAG00000004237 | ENSBTAG00000005534 |  |  |
| ENSBTAG00000004238 | ENSBTAG00000005542 |  |  |
| ENSBTAG00000004240 | ENSBTAG00000005556 |  |  |
| ENSBTAG00000004243 | ENSBTAG00000005571 |  |  |
| ENSBTAG00000004247 | ENSBTAG00000005573 |  |  |
| ENSBTAG00000004257 | ENSBTAG00000005581 |  |  |
| ENSBTAG00000004258 | ENSBTAG00000005586 |  |  |
| ENSBTAG00000004259 | ENSBTAG00000005589 |  |  |
| ENSBTAG00000004261 | ENSBTAG00000005615 |  |  |
| ENSBTAG00000004263 | ENSBTAG00000005622 |  |  |
| ENSBTAG00000004269 | ENSBTAG00000005623 |  |  |
| ENSBTAG00000004271 | ENSBTAG00000005627 |  |  |
| ENSBTAG00000004272 | ENSBTAG00000005635 |  |  |
| ENSBTAG00000004273 | ENSBTAG00000005644 |  |  |
| ENSBTAG00000004275 | ENSBTAG00000005650 |  |  |
| ENSBTAG00000004277 | ENSBTAG00000005653 |  |  |
| ENSBTAG00000004278 | ENSBTAG00000005657 |  |  |
| ENSBTAG00000004279 | ENSBTAG00000005658 |  |  |
| ENSBTAG00000004280 | ENSBTAG00000005661 |  |  |
| ENSBTAG00000004281 | ENSBTAG00000005663 |  |  |
| ENSBTAG00000004283 | ENSBTAG00000005664 |  |  |
| ENSBTAG00000004284 | ENSBTAG00000005665 |  |  |
| ENSBTAG00000004287 | ENSBTAG00000005666 |  |  |
| ENSBTAG00000004288 | ENSBTAG00000005668 |  |  |
| ENSBTAG00000004290 | ENSBTAG00000005674 |  |  |
| ENSBTAG00000004291 | ENSBTAG00000005678 |  |  |
| ENSBTAG00000004292 | ENSBTAG00000005679 |  |  |
| ENSBTAG00000004293 | ENSBTAG00000005681 |  |  |
| ENSBTAG00000004294 | ENSBTAG00000005682 |  |  |
| ENSBTAG00000004295 | ENSBTAG00000005688 |  |  |
| ENSBTAG00000004296 | ENSBTAG00000005691 |  |  |
| ENSBTAG00000004303 | ENSBTAG00000005693 |  |  |
| ENSBTAG00000004305 | ENSBTAG00000005695 |  |  |
| ENSBTAG00000004307 | ENSBTAG00000005699 |  |  |
| ENSBTAG00000004310 | ENSBTAG00000005710 |  |  |
| ENSBTAG00000004316 | ENSBTAG00000005712 |  |  |
| ENSBTAG00000004321 | ENSBTAG00000005714 |  |  |
| ENSBTAG00000004322 | ENSBTAG00000005726 |  |  |
| ENSBTAG00000004328 | ENSBTAG00000005730 |  |  |
| ENSBTAG00000004331 | ENSBTAG00000005744 |  |  |
| ENSBTAG00000004333 | ENSBTAG00000005754 |  |  |
| ENSBTAG00000004337 | ENSBTAG00000005757 |  |  |
| ENSBTAG00000004339 | ENSBTAG00000005759 |  |  |
| ENSBTAG00000004343 | ENSBTAG00000005760 |  |  |
| ENSBTAG00000004344 | ENSBTAG00000005763 |  |  |
| ENSBTAG00000004347 | ENSBTAG00000005773 |  |  |
| ENSBTAG00000004349 | ENSBTAG00000005780 |  |  |
| ENSBTAG00000004354 | ENSBTAG00000005785 |  |  |
| ENSBTAG00000004356 | ENSBTAG00000005792 |  |  |
| ENSBTAG00000004358 | ENSBTAG00000005793 |  |  |
| ENSBTAG00000004364 | ENSBTAG00000005795 |  |  |
| ENSBTAG00000004367 | ENSBTAG00000005805 |  |  |
| ENSBTAG00000004368 | ENSBTAG00000005807 |  |  |
| ENSBTAG00000004371 | ENSBTAG00000005808 |  |  |
| ENSBTAG00000004375 | ENSBTAG00000005824 |  |  |
| ENSBTAG00000004376 | ENSBTAG00000005828 |  |  |
| ENSBTAG00000004377 | ENSBTAG00000005830 |  |  |
| ENSBTAG00000004379 | ENSBTAG00000005832 |  |  |
| ENSBTAG00000004380 | ENSBTAG00000005835 |  |  |
| ENSBTAG00000004383 | ENSBTAG00000005838 |  |  |
| ENSBTAG00000004392 | ENSBTAG00000005844 |  |  |
| ENSBTAG00000004394 | ENSBTAG00000005846 |  |  |
| ENSBTAG00000004400 | ENSBTAG00000005851 |  |  |
| ENSBTAG00000004401 | ENSBTAG00000005852 |  |  |
| ENSBTAG00000004402 | ENSBTAG00000005854 |  |  |
| ENSBTAG00000004403 | ENSBTAG00000005857 |  |  |
| ENSBTAG00000004407 | ENSBTAG00000005862 |  |  |
| ENSBTAG00000004409 | ENSBTAG00000005863 |  |  |
| ENSBTAG00000004411 | ENSBTAG00000005869 |  |  |
| ENSBTAG00000004416 | ENSBTAG00000005891 |  |  |
| ENSBTAG00000004420 | ENSBTAG00000005892 |  |  |
| ENSBTAG00000004421 | ENSBTAG00000005893 |  |  |
| ENSBTAG00000004422 | ENSBTAG00000005897 |  |  |
| ENSBTAG00000004423 | ENSBTAG00000005903 |  |  |
| ENSBTAG00000004427 | ENSBTAG00000005904 |  |  |
| ENSBTAG00000004428 | ENSBTAG00000005905 |  |  |
| ENSBTAG00000004430 | ENSBTAG00000005909 |  |  |
| ENSBTAG00000004432 | ENSBTAG00000005913 |  |  |
| ENSBTAG00000004436 | ENSBTAG00000005917 |  |  |
| ENSBTAG00000004440 | ENSBTAG00000005922 |  |  |
| ENSBTAG00000004442 | ENSBTAG00000005929 |  |  |
| ENSBTAG00000004449 | ENSBTAG00000005932 |  |  |
| ENSBTAG00000004452 | ENSBTAG00000005933 |  |  |
| ENSBTAG00000004457 | ENSBTAG00000005934 |  |  |
| ENSBTAG00000004458 | ENSBTAG00000005945 |  |  |
| ENSBTAG00000004459 | ENSBTAG00000005955 |  |  |
| ENSBTAG00000004460 | ENSBTAG00000005957 |  |  |
| ENSBTAG00000004464 | ENSBTAG00000005960 |  |  |
| ENSBTAG00000004471 | ENSBTAG00000005961 |  |  |
| ENSBTAG00000004472 | ENSBTAG00000005963 |  |  |
| ENSBTAG00000004489 | ENSBTAG00000005970 |  |  |
| ENSBTAG00000004490 | ENSBTAG00000005975 |  |  |
| ENSBTAG00000004492 | ENSBTAG00000005976 |  |  |
| ENSBTAG00000004494 | ENSBTAG00000005979 |  |  |
| ENSBTAG00000004495 | ENSBTAG00000005984 |  |  |
| ENSBTAG00000004496 | ENSBTAG00000005989 |  |  |
| ENSBTAG00000004499 | ENSBTAG00000005990 |  |  |
| ENSBTAG00000004502 | ENSBTAG00000005997 |  |  |
| ENSBTAG00000004505 | ENSBTAG00000005998 |  |  |
| ENSBTAG00000004508 | ENSBTAG00000006003 |  |  |
| ENSBTAG00000004511 | ENSBTAG00000006007 |  |  |
| ENSBTAG00000004517 | ENSBTAG00000006008 |  |  |
| ENSBTAG00000004521 | ENSBTAG00000006010 |  |  |
| ENSBTAG00000004541 | ENSBTAG00000006016 |  |  |
| ENSBTAG00000004542 | ENSBTAG00000006021 |  |  |
| ENSBTAG00000004547 | ENSBTAG00000006022 |  |  |
| ENSBTAG00000004549 | ENSBTAG00000006025 |  |  |
| ENSBTAG00000004551 | ENSBTAG00000006029 |  |  |
| ENSBTAG00000004553 | ENSBTAG00000006035 |  |  |
| ENSBTAG00000004554 | ENSBTAG00000006036 |  |  |
| ENSBTAG00000004556 | ENSBTAG00000006037 |  |  |
| ENSBTAG00000004557 | ENSBTAG00000006039 |  |  |
| ENSBTAG00000004571 | ENSBTAG00000006043 |  |  |
| ENSBTAG00000004572 | ENSBTAG00000006045 |  |  |
| ENSBTAG00000004574 | ENSBTAG00000006050 |  |  |
| ENSBTAG00000004581 | ENSBTAG00000006052 |  |  |
| ENSBTAG00000004587 | ENSBTAG00000006063 |  |  |
| ENSBTAG00000004588 | ENSBTAG00000006065 |  |  |
| ENSBTAG00000004590 | ENSBTAG00000006066 |  |  |
| ENSBTAG00000004594 | ENSBTAG00000006067 |  |  |
| ENSBTAG00000004597 | ENSBTAG00000006068 |  |  |
| ENSBTAG00000004598 | ENSBTAG00000006069 |  |  |
| ENSBTAG00000004599 | ENSBTAG00000006070 |  |  |
| ENSBTAG00000004600 | ENSBTAG00000006071 |  |  |
| ENSBTAG00000004603 | ENSBTAG00000006072 |  |  |
| ENSBTAG00000004604 | ENSBTAG00000006078 |  |  |
| ENSBTAG00000004607 | ENSBTAG00000006084 |  |  |
| ENSBTAG00000004608 | ENSBTAG00000006088 |  |  |
| ENSBTAG00000004613 | ENSBTAG00000006096 |  |  |
| ENSBTAG00000004622 | ENSBTAG00000006099 |  |  |
| ENSBTAG00000004635 | ENSBTAG00000006108 |  |  |
| ENSBTAG00000004639 | ENSBTAG00000006118 |  |  |
| ENSBTAG00000004641 | ENSBTAG00000006129 |  |  |
| ENSBTAG00000004643 | ENSBTAG00000006130 |  |  |
| ENSBTAG00000004645 | ENSBTAG00000006136 |  |  |
| ENSBTAG00000004647 | ENSBTAG00000006139 |  |  |
| ENSBTAG00000004651 | ENSBTAG00000006140 |  |  |
| ENSBTAG00000004652 | ENSBTAG00000006142 |  |  |
| ENSBTAG00000004654 | ENSBTAG00000006143 |  |  |
| ENSBTAG00000004659 | ENSBTAG00000006160 |  |  |
| ENSBTAG00000004660 | ENSBTAG00000006161 |  |  |
| ENSBTAG00000004662 | ENSBTAG00000006162 |  |  |
| ENSBTAG00000004663 | ENSBTAG00000006165 |  |  |
| ENSBTAG00000004664 | ENSBTAG00000006166 |  |  |
| ENSBTAG00000004672 | ENSBTAG00000006173 |  |  |
| ENSBTAG00000004674 | ENSBTAG00000006177 |  |  |
| ENSBTAG00000004676 | ENSBTAG00000006188 |  |  |
| ENSBTAG00000004679 | ENSBTAG00000006189 |  |  |
| ENSBTAG00000004688 | ENSBTAG00000006193 |  |  |
| ENSBTAG00000004695 | ENSBTAG00000006197 |  |  |
| ENSBTAG00000004705 | ENSBTAG00000006202 |  |  |
| ENSBTAG00000004706 | ENSBTAG00000006208 |  |  |
| ENSBTAG00000004710 | ENSBTAG00000006213 |  |  |
| ENSBTAG00000004715 | ENSBTAG00000006225 |  |  |
| ENSBTAG00000004718 | ENSBTAG00000006234 |  |  |
| ENSBTAG00000004722 | ENSBTAG00000006235 |  |  |
| ENSBTAG00000004725 | ENSBTAG00000006240 |  |  |
| ENSBTAG00000004727 | ENSBTAG00000006241 |  |  |
| ENSBTAG00000004732 | ENSBTAG00000006244 |  |  |
| ENSBTAG00000004736 | ENSBTAG00000006247 |  |  |
| ENSBTAG00000004738 | ENSBTAG00000006250 |  |  |
| ENSBTAG00000004739 | ENSBTAG00000006255 |  |  |
| ENSBTAG00000004745 | ENSBTAG00000006259 |  |  |
| ENSBTAG00000004747 | ENSBTAG00000006261 |  |  |
| ENSBTAG00000004750 | ENSBTAG00000006262 |  |  |
| ENSBTAG00000004751 | ENSBTAG00000006263 |  |  |
| ENSBTAG00000004754 | ENSBTAG00000006270 |  |  |
| ENSBTAG00000004755 | ENSBTAG00000006273 |  |  |
| ENSBTAG00000004767 | ENSBTAG00000006276 |  |  |
| ENSBTAG00000004769 | ENSBTAG00000006280 |  |  |
| ENSBTAG00000004775 | ENSBTAG00000006282 |  |  |
| ENSBTAG00000004776 | ENSBTAG00000006287 |  |  |
| ENSBTAG00000004777 | ENSBTAG00000006296 |  |  |
| ENSBTAG00000004781 | ENSBTAG00000006320 |  |  |
| ENSBTAG00000004782 | ENSBTAG00000006322 |  |  |
| ENSBTAG00000004783 | ENSBTAG00000006323 |  |  |
| ENSBTAG00000004785 | ENSBTAG00000006324 |  |  |
| ENSBTAG00000004786 | ENSBTAG00000006325 |  |  |
| ENSBTAG00000004788 | ENSBTAG00000006326 |  |  |
| ENSBTAG00000004791 | ENSBTAG00000006330 |  |  |
| ENSBTAG00000004792 | ENSBTAG00000006345 |  |  |
| ENSBTAG00000004793 | ENSBTAG00000006346 |  |  |
| ENSBTAG00000004799 | ENSBTAG00000006349 |  |  |
| ENSBTAG00000004801 | ENSBTAG00000006350 |  |  |
| ENSBTAG00000004803 | ENSBTAG00000006355 |  |  |
| ENSBTAG00000004804 | ENSBTAG00000006359 |  |  |
| ENSBTAG00000004805 | ENSBTAG00000006364 |  |  |
| ENSBTAG00000004813 | ENSBTAG00000006367 |  |  |
| ENSBTAG00000004822 | ENSBTAG00000006370 |  |  |
| ENSBTAG00000004824 | ENSBTAG00000006374 |  |  |
| ENSBTAG00000004829 | ENSBTAG00000006377 |  |  |
| ENSBTAG00000004832 | ENSBTAG00000006378 |  |  |
| ENSBTAG00000004833 | ENSBTAG00000006379 |  |  |
| ENSBTAG00000004838 | ENSBTAG00000006396 |  |  |
| ENSBTAG00000004840 | ENSBTAG00000006405 |  |  |
| ENSBTAG00000004842 | ENSBTAG00000006410 |  |  |
| ENSBTAG00000004844 | ENSBTAG00000006429 |  |  |
| ENSBTAG00000004851 | ENSBTAG00000006432 |  |  |
| ENSBTAG00000004855 | ENSBTAG00000006434 |  |  |
| ENSBTAG00000004863 | ENSBTAG00000006439 |  |  |
| ENSBTAG00000004869 | ENSBTAG00000006451 |  |  |
| ENSBTAG00000004870 | ENSBTAG00000006452 |  |  |
| ENSBTAG00000004871 | ENSBTAG00000006464 |  |  |
| ENSBTAG00000004872 | ENSBTAG00000006466 |  |  |
| ENSBTAG00000004873 | ENSBTAG00000006471 |  |  |
| ENSBTAG00000004875 | ENSBTAG00000006481 |  |  |
| ENSBTAG00000004876 | ENSBTAG00000006482 |  |  |
| ENSBTAG00000004877 | ENSBTAG00000006489 |  |  |
| ENSBTAG00000004878 | ENSBTAG00000006491 |  |  |
| ENSBTAG00000004879 | ENSBTAG00000006492 |  |  |
| ENSBTAG00000004885 | ENSBTAG00000006494 |  |  |
| ENSBTAG00000004886 | ENSBTAG00000006504 |  |  |
| ENSBTAG00000004887 | ENSBTAG00000006506 |  |  |
| ENSBTAG00000004888 | ENSBTAG00000006510 |  |  |
| ENSBTAG00000004891 | ENSBTAG00000006511 |  |  |
| ENSBTAG00000004894 | ENSBTAG00000006517 |  |  |
| ENSBTAG00000004895 | ENSBTAG00000006525 |  |  |
| ENSBTAG00000004896 | ENSBTAG00000006526 |  |  |
| ENSBTAG00000004906 | ENSBTAG00000006529 |  |  |
| ENSBTAG00000004907 | ENSBTAG00000006534 |  |  |
| ENSBTAG00000004908 | ENSBTAG00000006538 |  |  |
| ENSBTAG00000004910 | ENSBTAG00000006542 |  |  |
| ENSBTAG00000004912 | ENSBTAG00000006546 |  |  |
| ENSBTAG00000004913 | ENSBTAG00000006550 |  |  |
| ENSBTAG00000004916 | ENSBTAG00000006552 |  |  |
| ENSBTAG00000004922 | ENSBTAG00000006556 |  |  |
| ENSBTAG00000004924 | ENSBTAG00000006561 |  |  |
| ENSBTAG00000004930 | ENSBTAG00000006563 |  |  |
| ENSBTAG00000004936 | ENSBTAG00000006564 |  |  |
| ENSBTAG00000004940 | ENSBTAG00000006567 |  |  |
| ENSBTAG00000004948 | ENSBTAG00000006573 |  |  |
| ENSBTAG00000004950 | ENSBTAG00000006578 |  |  |
| ENSBTAG00000004952 | ENSBTAG00000006579 |  |  |
| ENSBTAG00000004953 | ENSBTAG00000006587 |  |  |
| ENSBTAG00000004954 | ENSBTAG00000006589 |  |  |
| ENSBTAG00000004956 | ENSBTAG00000006590 |  |  |
| ENSBTAG00000004962 | ENSBTAG00000006592 |  |  |
| ENSBTAG00000004964 | ENSBTAG00000006607 |  |  |
| ENSBTAG00000004965 | ENSBTAG00000006609 |  |  |
| ENSBTAG00000004970 | ENSBTAG00000006610 |  |  |
| ENSBTAG00000004971 | ENSBTAG00000006612 |  |  |
| ENSBTAG00000004976 | ENSBTAG00000006614 |  |  |
| ENSBTAG00000004977 | ENSBTAG00000006615 |  |  |
| ENSBTAG00000004979 | ENSBTAG00000006624 |  |  |
| ENSBTAG00000004982 | ENSBTAG00000006630 |  |  |
| ENSBTAG00000004988 | ENSBTAG00000006635 |  |  |
| ENSBTAG00000004989 | ENSBTAG00000006639 |  |  |
| ENSBTAG00000004990 | ENSBTAG00000006640 |  |  |
| ENSBTAG00000004992 | ENSBTAG00000006643 |  |  |
| ENSBTAG00000004995 | ENSBTAG00000006645 |  |  |
| ENSBTAG00000004996 | ENSBTAG00000006647 |  |  |
| ENSBTAG00000004999 | ENSBTAG00000006650 |  |  |
| ENSBTAG00000005002 | ENSBTAG00000006654 |  |  |
| ENSBTAG00000005005 | ENSBTAG00000006656 |  |  |
| ENSBTAG00000005009 | ENSBTAG00000006657 |  |  |
| ENSBTAG00000005010 | ENSBTAG00000006662 |  |  |
| ENSBTAG00000005012 | ENSBTAG00000006666 |  |  |
| ENSBTAG00000005015 | ENSBTAG00000006674 |  |  |
| ENSBTAG00000005016 | ENSBTAG00000006675 |  |  |
| ENSBTAG00000005018 | ENSBTAG00000006676 |  |  |
| ENSBTAG00000005025 | ENSBTAG00000006680 |  |  |
| ENSBTAG00000005027 | ENSBTAG00000006685 |  |  |
| ENSBTAG00000005028 | ENSBTAG00000006686 |  |  |
| ENSBTAG00000005029 | ENSBTAG00000006688 |  |  |
| ENSBTAG00000005031 | ENSBTAG00000006689 |  |  |
| ENSBTAG00000005034 | ENSBTAG00000006690 |  |  |
| ENSBTAG00000005039 | ENSBTAG00000006691 |  |  |
| ENSBTAG00000005041 | ENSBTAG00000006694 |  |  |
| ENSBTAG00000005043 | ENSBTAG00000006697 |  |  |
| ENSBTAG00000005045 | ENSBTAG00000006702 |  |  |
| ENSBTAG00000005049 | ENSBTAG00000006703 |  |  |
| ENSBTAG00000005062 | ENSBTAG00000006704 |  |  |
| ENSBTAG00000005063 | ENSBTAG00000006707 |  |  |
| ENSBTAG00000005066 | ENSBTAG00000006708 |  |  |
| ENSBTAG00000005068 | ENSBTAG00000006712 |  |  |
| ENSBTAG00000005069 | ENSBTAG00000006713 |  |  |
| ENSBTAG00000005071 | ENSBTAG00000006714 |  |  |
| ENSBTAG00000005075 | ENSBTAG00000006715 |  |  |
| ENSBTAG00000005077 | ENSBTAG00000006716 |  |  |
| ENSBTAG00000005078 | ENSBTAG00000006719 |  |  |
| ENSBTAG00000005082 | ENSBTAG00000006720 |  |  |
| ENSBTAG00000005083 | ENSBTAG00000006721 |  |  |
| ENSBTAG00000005085 | ENSBTAG00000006722 |  |  |
| ENSBTAG00000005086 | ENSBTAG00000006726 |  |  |
| ENSBTAG00000005089 | ENSBTAG00000006730 |  |  |
| ENSBTAG00000005090 | ENSBTAG00000006731 |  |  |
| ENSBTAG00000005092 | ENSBTAG00000006735 |  |  |
| ENSBTAG00000005094 | ENSBTAG00000006739 |  |  |
| ENSBTAG00000005100 | ENSBTAG00000006740 |  |  |
| ENSBTAG00000005102 | ENSBTAG00000006745 |  |  |
| ENSBTAG00000005104 | ENSBTAG00000006747 |  |  |
| ENSBTAG00000005105 | ENSBTAG00000006753 |  |  |
| ENSBTAG00000005107 | ENSBTAG00000006755 |  |  |
| ENSBTAG00000005110 | ENSBTAG00000006756 |  |  |
| ENSBTAG00000005111 | ENSBTAG00000006759 |  |  |
| ENSBTAG00000005115 | ENSBTAG00000006761 |  |  |
| ENSBTAG00000005120 | ENSBTAG00000006765 |  |  |
| ENSBTAG00000005122 | ENSBTAG00000006775 |  |  |
| ENSBTAG00000005133 | ENSBTAG00000006779 |  |  |
| ENSBTAG00000005136 | ENSBTAG00000006786 |  |  |
| ENSBTAG00000005137 | ENSBTAG00000006789 |  |  |
| ENSBTAG00000005144 | ENSBTAG00000006790 |  |  |
| ENSBTAG00000005146 | ENSBTAG00000006792 |  |  |
| ENSBTAG00000005152 | ENSBTAG00000006801 |  |  |
| ENSBTAG00000005158 | ENSBTAG00000006804 |  |  |
| ENSBTAG00000005160 | ENSBTAG00000006807 |  |  |
| ENSBTAG00000005161 | ENSBTAG00000006810 |  |  |
| ENSBTAG00000005163 | ENSBTAG00000006812 |  |  |
| ENSBTAG00000005166 | ENSBTAG00000006816 |  |  |
| ENSBTAG00000005174 | ENSBTAG00000006828 |  |  |
| ENSBTAG00000005181 | ENSBTAG00000006832 |  |  |
| ENSBTAG00000005183 | ENSBTAG00000006835 |  |  |
| ENSBTAG00000005186 | ENSBTAG00000006837 |  |  |
| ENSBTAG00000005189 | ENSBTAG00000006843 |  |  |
| ENSBTAG00000005191 | ENSBTAG00000006844 |  |  |
| ENSBTAG00000005193 | ENSBTAG00000006846 |  |  |
| ENSBTAG00000005195 | ENSBTAG00000006852 |  |  |
| ENSBTAG00000005197 | ENSBTAG00000006862 |  |  |
| ENSBTAG00000005202 | ENSBTAG00000006869 |  |  |
| ENSBTAG00000005205 | ENSBTAG00000006877 |  |  |
| ENSBTAG00000005206 | ENSBTAG00000006881 |  |  |
| ENSBTAG00000005207 | ENSBTAG00000006884 |  |  |
| ENSBTAG00000005210 | ENSBTAG00000006886 |  |  |
| ENSBTAG00000005211 | ENSBTAG00000006896 |  |  |
| ENSBTAG00000005212 | ENSBTAG00000006899 |  |  |
| ENSBTAG00000005218 | ENSBTAG00000006901 |  |  |
| ENSBTAG00000005221 | ENSBTAG00000006904 |  |  |
| ENSBTAG00000005225 | ENSBTAG00000006914 |  |  |
| ENSBTAG00000005227 | ENSBTAG00000006918 |  |  |
| ENSBTAG00000005230 | ENSBTAG00000006919 |  |  |
| ENSBTAG00000005231 | ENSBTAG00000006921 |  |  |
| ENSBTAG00000005234 | ENSBTAG00000006925 |  |  |
| ENSBTAG00000005235 | ENSBTAG00000006928 |  |  |
| ENSBTAG00000005236 | ENSBTAG00000006934 |  |  |
| ENSBTAG00000005243 | ENSBTAG00000006936 |  |  |
| ENSBTAG00000005246 | ENSBTAG00000006940 |  |  |
| ENSBTAG00000005249 | ENSBTAG00000006941 |  |  |
| ENSBTAG00000005250 | ENSBTAG00000006948 |  |  |
| ENSBTAG00000005252 | ENSBTAG00000006951 |  |  |
| ENSBTAG00000005257 | ENSBTAG00000006957 |  |  |
| ENSBTAG00000005259 | ENSBTAG00000006962 |  |  |
| ENSBTAG00000005260 | ENSBTAG00000006971 |  |  |
| ENSBTAG00000005263 | ENSBTAG00000006972 |  |  |
| ENSBTAG00000005269 | ENSBTAG00000006977 |  |  |
| ENSBTAG00000005272 | ENSBTAG00000006982 |  |  |
| ENSBTAG00000005275 | ENSBTAG00000006984 |  |  |
| ENSBTAG00000005278 | ENSBTAG00000006985 |  |  |
| ENSBTAG00000005280 | ENSBTAG00000006987 |  |  |
| ENSBTAG00000005287 | ENSBTAG00000006989 |  |  |
| ENSBTAG00000005288 | ENSBTAG00000006995 |  |  |
| ENSBTAG00000005289 | ENSBTAG00000007012 |  |  |
| ENSBTAG00000005290 | ENSBTAG00000007013 |  |  |
| ENSBTAG00000005294 | ENSBTAG00000007016 |  |  |
| ENSBTAG00000005295 | ENSBTAG00000007020 |  |  |
| ENSBTAG00000005299 | ENSBTAG00000007035 |  |  |
| ENSBTAG00000005300 | ENSBTAG00000007036 |  |  |
| ENSBTAG00000005304 | ENSBTAG00000007039 |  |  |
| ENSBTAG00000005305 | ENSBTAG00000007066 |  |  |
| ENSBTAG00000005308 | ENSBTAG00000007071 |  |  |
| ENSBTAG00000005309 | ENSBTAG00000007073 |  |  |
| ENSBTAG00000005311 | ENSBTAG00000007074 |  |  |
| ENSBTAG00000005312 | ENSBTAG00000007080 |  |  |
| ENSBTAG00000005314 | ENSBTAG00000007084 |  |  |
| ENSBTAG00000005316 | ENSBTAG00000007094 |  |  |
| ENSBTAG00000005318 | ENSBTAG00000007096 |  |  |
| ENSBTAG00000005321 | ENSBTAG00000007097 |  |  |
| ENSBTAG00000005328 | ENSBTAG00000007101 |  |  |
| ENSBTAG00000005337 | ENSBTAG00000007103 |  |  |
| ENSBTAG00000005339 | ENSBTAG00000007104 |  |  |
| ENSBTAG00000005345 | ENSBTAG00000007106 |  |  |
| ENSBTAG00000005353 | ENSBTAG00000007107 |  |  |
| ENSBTAG00000005354 | ENSBTAG00000007109 |  |  |
| ENSBTAG00000005355 | ENSBTAG00000007112 |  |  |
| ENSBTAG00000005356 | ENSBTAG00000007113 |  |  |
| ENSBTAG00000005357 | ENSBTAG00000007116 |  |  |
| ENSBTAG00000005359 | ENSBTAG00000007122 |  |  |
| ENSBTAG00000005364 | ENSBTAG00000007123 |  |  |
| ENSBTAG00000005367 | ENSBTAG00000007125 |  |  |
| ENSBTAG00000005370 | ENSBTAG00000007128 |  |  |
| ENSBTAG00000005371 | ENSBTAG00000007129 |  |  |
| ENSBTAG00000005376 | ENSBTAG00000007130 |  |  |
| ENSBTAG00000005377 | ENSBTAG00000007131 |  |  |
| ENSBTAG00000005380 | ENSBTAG00000007133 |  |  |
| ENSBTAG00000005384 | ENSBTAG00000007135 |  |  |
| ENSBTAG00000005385 | ENSBTAG00000007137 |  |  |
| ENSBTAG00000005386 | ENSBTAG00000007139 |  |  |
| ENSBTAG00000005397 | ENSBTAG00000007141 |  |  |
| ENSBTAG00000005403 | ENSBTAG00000007142 |  |  |
| ENSBTAG00000005404 | ENSBTAG00000007145 |  |  |
| ENSBTAG00000005413 | ENSBTAG00000007147 |  |  |
| ENSBTAG00000005414 | ENSBTAG00000007152 |  |  |
| ENSBTAG00000005416 | ENSBTAG00000007164 |  |  |
| ENSBTAG00000005419 | ENSBTAG00000007167 |  |  |
| ENSBTAG00000005424 | ENSBTAG00000007170 |  |  |
| ENSBTAG00000005425 | ENSBTAG00000007172 |  |  |
| ENSBTAG00000005426 | ENSBTAG00000007173 |  |  |
| ENSBTAG00000005427 | ENSBTAG00000007176 |  |  |
| ENSBTAG00000005431 | ENSBTAG00000007184 |  |  |
| ENSBTAG00000005432 | ENSBTAG00000007189 |  |  |
| ENSBTAG00000005434 | ENSBTAG00000007190 |  |  |
| ENSBTAG00000005436 | ENSBTAG00000007192 |  |  |
| ENSBTAG00000005437 | ENSBTAG00000007201 |  |  |
| ENSBTAG00000005439 | ENSBTAG00000007202 |  |  |
| ENSBTAG00000005441 | ENSBTAG00000007204 |  |  |
| ENSBTAG00000005443 | ENSBTAG00000007213 |  |  |
| ENSBTAG00000005445 | ENSBTAG00000007214 |  |  |
| ENSBTAG00000005446 | ENSBTAG00000007216 |  |  |
| ENSBTAG00000005448 | ENSBTAG00000007220 |  |  |
| ENSBTAG00000005453 | ENSBTAG00000007230 |  |  |
| ENSBTAG00000005454 | ENSBTAG00000007234 |  |  |
| ENSBTAG00000005464 | ENSBTAG00000007235 |  |  |
| ENSBTAG00000005469 | ENSBTAG00000007237 |  |  |
| ENSBTAG00000005470 | ENSBTAG00000007243 |  |  |
| ENSBTAG00000005471 | ENSBTAG00000007247 |  |  |
| ENSBTAG00000005474 | ENSBTAG00000007253 |  |  |
| ENSBTAG00000005475 | ENSBTAG00000007258 |  |  |
| ENSBTAG00000005476 | ENSBTAG00000007268 |  |  |
| ENSBTAG00000005477 | ENSBTAG00000007269 |  |  |
| ENSBTAG00000005478 | ENSBTAG00000007270 |  |  |
| ENSBTAG00000005481 | ENSBTAG00000007272 |  |  |
| ENSBTAG00000005483 | ENSBTAG00000007299 |  |  |
| ENSBTAG00000005488 | ENSBTAG00000007303 |  |  |
| ENSBTAG00000005492 | ENSBTAG00000007304 |  |  |
| ENSBTAG00000005493 | ENSBTAG00000007305 |  |  |
| ENSBTAG00000005496 | ENSBTAG00000007306 |  |  |
| ENSBTAG00000005497 | ENSBTAG00000007307 |  |  |
| ENSBTAG00000005498 | ENSBTAG00000007308 |  |  |
| ENSBTAG00000005503 | ENSBTAG00000007309 |  |  |
| ENSBTAG00000005517 | ENSBTAG00000007315 |  |  |
| ENSBTAG00000005519 | ENSBTAG00000007316 |  |  |
| ENSBTAG00000005524 | ENSBTAG00000007320 |  |  |
| ENSBTAG00000005526 | ENSBTAG00000007321 |  |  |
| ENSBTAG00000005530 | ENSBTAG00000007331 |  |  |
| ENSBTAG00000005532 | ENSBTAG00000007335 |  |  |
| ENSBTAG00000005533 | ENSBTAG00000007343 |  |  |
| ENSBTAG00000005534 | ENSBTAG00000007347 |  |  |
| ENSBTAG00000005542 | ENSBTAG00000007348 |  |  |
| ENSBTAG00000005547 | ENSBTAG00000007352 |  |  |
| ENSBTAG00000005556 | ENSBTAG00000007356 |  |  |
| ENSBTAG00000005564 | ENSBTAG00000007357 |  |  |
| ENSBTAG00000005565 | ENSBTAG00000007360 |  |  |
| ENSBTAG00000005571 | ENSBTAG00000007365 |  |  |
| ENSBTAG00000005573 | ENSBTAG00000007367 |  |  |
| ENSBTAG00000005574 | ENSBTAG00000007369 |  |  |
| ENSBTAG00000005576 | ENSBTAG00000007374 |  |  |
| ENSBTAG00000005578 | ENSBTAG00000007383 |  |  |
| ENSBTAG00000005586 | ENSBTAG00000007385 |  |  |
| ENSBTAG00000005587 | ENSBTAG00000007386 |  |  |
| ENSBTAG00000005589 | ENSBTAG00000007390 |  |  |
| ENSBTAG00000005592 | ENSBTAG00000007393 |  |  |
| ENSBTAG00000005595 | ENSBTAG00000007394 |  |  |
| ENSBTAG00000005596 | ENSBTAG00000007395 |  |  |
| ENSBTAG00000005604 | ENSBTAG00000007399 |  |  |
| ENSBTAG00000005606 | ENSBTAG00000007402 |  |  |
| ENSBTAG00000005607 | ENSBTAG00000007414 |  |  |
| ENSBTAG00000005615 | ENSBTAG00000007415 |  |  |
| ENSBTAG00000005622 | ENSBTAG00000007423 |  |  |
| ENSBTAG00000005623 | ENSBTAG00000007424 |  |  |
| ENSBTAG00000005627 | ENSBTAG00000007434 |  |  |
| ENSBTAG00000005631 | ENSBTAG00000007436 |  |  |
| ENSBTAG00000005635 | ENSBTAG00000007444 |  |  |
| ENSBTAG00000005638 | ENSBTAG00000007447 |  |  |
| ENSBTAG00000005644 | ENSBTAG00000007449 |  |  |
| ENSBTAG00000005650 | ENSBTAG00000007460 |  |  |
| ENSBTAG00000005653 | ENSBTAG00000007474 |  |  |
| ENSBTAG00000005654 | ENSBTAG00000007479 |  |  |
| ENSBTAG00000005657 | ENSBTAG00000007480 |  |  |
| ENSBTAG00000005658 | ENSBTAG00000007484 |  |  |
| ENSBTAG00000005660 | ENSBTAG00000007485 |  |  |
| ENSBTAG00000005661 | ENSBTAG00000007492 |  |  |
| ENSBTAG00000005663 | ENSBTAG00000007494 |  |  |
| ENSBTAG00000005664 | ENSBTAG00000007496 |  |  |
| ENSBTAG00000005665 | ENSBTAG00000007497 |  |  |
| ENSBTAG00000005666 | ENSBTAG00000007498 |  |  |
| ENSBTAG00000005668 | ENSBTAG00000007499 |  |  |
| ENSBTAG00000005670 | ENSBTAG00000007502 |  |  |
| ENSBTAG00000005674 | ENSBTAG00000007503 |  |  |
| ENSBTAG00000005676 | ENSBTAG00000007507 |  |  |
| ENSBTAG00000005678 | ENSBTAG00000007512 |  |  |
| ENSBTAG00000005679 | ENSBTAG00000007513 |  |  |
| ENSBTAG00000005681 | ENSBTAG00000007515 |  |  |
| ENSBTAG00000005682 | ENSBTAG00000007519 |  |  |
| ENSBTAG00000005685 | ENSBTAG00000007523 |  |  |
| ENSBTAG00000005688 | ENSBTAG00000007534 |  |  |
| ENSBTAG00000005691 | ENSBTAG00000007537 |  |  |
| ENSBTAG00000005693 | ENSBTAG00000007544 |  |  |
| ENSBTAG00000005694 | ENSBTAG00000007547 |  |  |
| ENSBTAG00000005695 | ENSBTAG00000007559 |  |  |
| ENSBTAG00000005699 | ENSBTAG00000007567 |  |  |
| ENSBTAG00000005710 | ENSBTAG00000007569 |  |  |
| ENSBTAG00000005712 | ENSBTAG00000007570 |  |  |
| ENSBTAG00000005714 | ENSBTAG00000007578 |  |  |
| ENSBTAG00000005715 | ENSBTAG00000007581 |  |  |
| ENSBTAG00000005716 | ENSBTAG00000007586 |  |  |
| ENSBTAG00000005719 | ENSBTAG00000007588 |  |  |
| ENSBTAG00000005726 | ENSBTAG00000007589 |  |  |
| ENSBTAG00000005730 | ENSBTAG00000007591 |  |  |
| ENSBTAG00000005732 | ENSBTAG00000007592 |  |  |
| ENSBTAG00000005738 | ENSBTAG00000007594 |  |  |
| ENSBTAG00000005744 | ENSBTAG00000007596 |  |  |
| ENSBTAG00000005751 | ENSBTAG00000007606 |  |  |
| ENSBTAG00000005754 | ENSBTAG00000007609 |  |  |
| ENSBTAG00000005757 | ENSBTAG00000007614 |  |  |
| ENSBTAG00000005759 | ENSBTAG00000007617 |  |  |
| ENSBTAG00000005760 | ENSBTAG00000007618 |  |  |
| ENSBTAG00000005761 | ENSBTAG00000007619 |  |  |
| ENSBTAG00000005763 | ENSBTAG00000007622 |  |  |
| ENSBTAG00000005765 | ENSBTAG00000007623 |  |  |
| ENSBTAG00000005773 | ENSBTAG00000007624 |  |  |
| ENSBTAG00000005780 | ENSBTAG00000007626 |  |  |
| ENSBTAG00000005785 | ENSBTAG00000007632 |  |  |
| ENSBTAG00000005791 | ENSBTAG00000007634 |  |  |
| ENSBTAG00000005792 | ENSBTAG00000007639 |  |  |
| ENSBTAG00000005793 | ENSBTAG00000007644 |  |  |
| ENSBTAG00000005795 | ENSBTAG00000007648 |  |  |
| ENSBTAG00000005805 | ENSBTAG00000007651 |  |  |
| ENSBTAG00000005807 | ENSBTAG00000007657 |  |  |
| ENSBTAG00000005808 | ENSBTAG00000007658 |  |  |
| ENSBTAG00000005816 | ENSBTAG00000007659 |  |  |
| ENSBTAG00000005824 | ENSBTAG00000007675 |  |  |
| ENSBTAG00000005827 | ENSBTAG00000007678 |  |  |
| ENSBTAG00000005828 | ENSBTAG00000007680 |  |  |
| ENSBTAG00000005830 | ENSBTAG00000007681 |  |  |
| ENSBTAG00000005832 | ENSBTAG00000007683 |  |  |
| ENSBTAG00000005835 | ENSBTAG00000007684 |  |  |
| ENSBTAG00000005838 | ENSBTAG00000007690 |  |  |
| ENSBTAG00000005841 | ENSBTAG00000007695 |  |  |
| ENSBTAG00000005842 | ENSBTAG00000007703 |  |  |
| ENSBTAG00000005844 | ENSBTAG00000007705 |  |  |
| ENSBTAG00000005846 | ENSBTAG00000007708 |  |  |
| ENSBTAG00000005851 | ENSBTAG00000007709 |  |  |
| ENSBTAG00000005852 | ENSBTAG00000007712 |  |  |
| ENSBTAG00000005854 | ENSBTAG00000007718 |  |  |
| ENSBTAG00000005857 | ENSBTAG00000007719 |  |  |
| ENSBTAG00000005859 | ENSBTAG00000007721 |  |  |
| ENSBTAG00000005861 | ENSBTAG00000007722 |  |  |
| ENSBTAG00000005862 | ENSBTAG00000007725 |  |  |
| ENSBTAG00000005863 | ENSBTAG00000007730 |  |  |
| ENSBTAG00000005868 | ENSBTAG00000007731 |  |  |
| ENSBTAG00000005869 | ENSBTAG00000007732 |  |  |
| ENSBTAG00000005871 | ENSBTAG00000007743 |  |  |
| ENSBTAG00000005891 | ENSBTAG00000007759 |  |  |
| ENSBTAG00000005892 | ENSBTAG00000007763 |  |  |
| ENSBTAG00000005893 | ENSBTAG00000007777 |  |  |
| ENSBTAG00000005897 | ENSBTAG00000007780 |  |  |
| ENSBTAG00000005903 | ENSBTAG00000007783 |  |  |
| ENSBTAG00000005904 | ENSBTAG00000007784 |  |  |
| ENSBTAG00000005905 | ENSBTAG00000007786 |  |  |
| ENSBTAG00000005909 | ENSBTAG00000007787 |  |  |
| ENSBTAG00000005913 | ENSBTAG00000007788 |  |  |
| ENSBTAG00000005917 | ENSBTAG00000007799 |  |  |
| ENSBTAG00000005922 | ENSBTAG00000007806 |  |  |
| ENSBTAG00000005929 | ENSBTAG00000007813 |  |  |
| ENSBTAG00000005932 | ENSBTAG00000007814 |  |  |
| ENSBTAG00000005933 | ENSBTAG00000007818 |  |  |
| ENSBTAG00000005934 | ENSBTAG00000007828 |  |  |
| ENSBTAG00000005940 | ENSBTAG00000007835 |  |  |
| ENSBTAG00000005945 | ENSBTAG00000007837 |  |  |
| ENSBTAG00000005947 | ENSBTAG00000007840 |  |  |
| ENSBTAG00000005955 | ENSBTAG00000007842 |  |  |
| ENSBTAG00000005957 | ENSBTAG00000007847 |  |  |
| ENSBTAG00000005960 | ENSBTAG00000007859 |  |  |
| ENSBTAG00000005961 | ENSBTAG00000007863 |  |  |
| ENSBTAG00000005963 | ENSBTAG00000007865 |  |  |
| ENSBTAG00000005967 | ENSBTAG00000007867 |  |  |
| ENSBTAG00000005969 | ENSBTAG00000007870 |  |  |
| ENSBTAG00000005970 | ENSBTAG00000007871 |  |  |
| ENSBTAG00000005971 | ENSBTAG00000007876 |  |  |
| ENSBTAG00000005973 | ENSBTAG00000007880 |  |  |
| ENSBTAG00000005974 | ENSBTAG00000007884 |  |  |
| ENSBTAG00000005975 | ENSBTAG00000007893 |  |  |
| ENSBTAG00000005976 | ENSBTAG00000007895 |  |  |
| ENSBTAG00000005978 | ENSBTAG00000007896 |  |  |
| ENSBTAG00000005979 | ENSBTAG00000007900 |  |  |
| ENSBTAG00000005984 | ENSBTAG00000007904 |  |  |
| ENSBTAG00000005985 | ENSBTAG00000007920 |  |  |
| ENSBTAG00000005989 | ENSBTAG00000007921 |  |  |
| ENSBTAG00000005990 | ENSBTAG00000007923 |  |  |
| ENSBTAG00000005994 | ENSBTAG00000007934 |  |  |
| ENSBTAG00000005996 | ENSBTAG00000007937 |  |  |
| ENSBTAG00000005997 | ENSBTAG00000007939 |  |  |
| ENSBTAG00000005998 | ENSBTAG00000007942 |  |  |
| ENSBTAG00000006003 | ENSBTAG00000007943 |  |  |
| ENSBTAG00000006005 | ENSBTAG00000007946 |  |  |
| ENSBTAG00000006007 | ENSBTAG00000007954 |  |  |
| ENSBTAG00000006008 | ENSBTAG00000007958 |  |  |
| ENSBTAG00000006010 | ENSBTAG00000007969 |  |  |
| ENSBTAG00000006016 | ENSBTAG00000007974 |  |  |
| ENSBTAG00000006021 | ENSBTAG00000007975 |  |  |
| ENSBTAG00000006022 | ENSBTAG00000007976 |  |  |
| ENSBTAG00000006024 | ENSBTAG00000007993 |  |  |
| ENSBTAG00000006025 | ENSBTAG00000007994 |  |  |
| ENSBTAG00000006027 | ENSBTAG00000008001 |  |  |
| ENSBTAG00000006029 | ENSBTAG00000008003 |  |  |
| ENSBTAG00000006030 | ENSBTAG00000008004 |  |  |
| ENSBTAG00000006035 | ENSBTAG00000008005 |  |  |
| ENSBTAG00000006036 | ENSBTAG00000008006 |  |  |
| ENSBTAG00000006037 | ENSBTAG00000008008 |  |  |
| ENSBTAG00000006039 | ENSBTAG00000008016 |  |  |
| ENSBTAG00000006040 | ENSBTAG00000008022 |  |  |
| ENSBTAG00000006043 | ENSBTAG00000008023 |  |  |
| ENSBTAG00000006044 | ENSBTAG00000008025 |  |  |
| ENSBTAG00000006045 | ENSBTAG00000008028 |  |  |
| ENSBTAG00000006049 | ENSBTAG00000008032 |  |  |
| ENSBTAG00000006050 | ENSBTAG00000008034 |  |  |
| ENSBTAG00000006051 | ENSBTAG00000008040 |  |  |
| ENSBTAG00000006052 | ENSBTAG00000008048 |  |  |
| ENSBTAG00000006054 | ENSBTAG00000008053 |  |  |
| ENSBTAG00000006056 | ENSBTAG00000008054 |  |  |
| ENSBTAG00000006063 | ENSBTAG00000008056 |  |  |
| ENSBTAG00000006065 | ENSBTAG00000008065 |  |  |
| ENSBTAG00000006066 | ENSBTAG00000008072 |  |  |
| ENSBTAG00000006067 | ENSBTAG00000008074 |  |  |
| ENSBTAG00000006068 | ENSBTAG00000008076 |  |  |
| ENSBTAG00000006070 | ENSBTAG00000008077 |  |  |
| ENSBTAG00000006071 | ENSBTAG00000008079 |  |  |
| ENSBTAG00000006072 | ENSBTAG00000008084 |  |  |
| ENSBTAG00000006078 | ENSBTAG00000008088 |  |  |
| ENSBTAG00000006082 | ENSBTAG00000008089 |  |  |
| ENSBTAG00000006084 | ENSBTAG00000008096 |  |  |
| ENSBTAG00000006088 | ENSBTAG00000008097 |  |  |
| ENSBTAG00000006096 | ENSBTAG00000008099 |  |  |
| ENSBTAG00000006099 | ENSBTAG00000008100 |  |  |
| ENSBTAG00000006103 | ENSBTAG00000008109 |  |  |
| ENSBTAG00000006104 | ENSBTAG00000008112 |  |  |
| ENSBTAG00000006108 | ENSBTAG00000008114 |  |  |
| ENSBTAG00000006116 | ENSBTAG00000008116 |  |  |
| ENSBTAG00000006118 | ENSBTAG00000008118 |  |  |
| ENSBTAG00000006126 | ENSBTAG00000008120 |  |  |
| ENSBTAG00000006129 | ENSBTAG00000008121 |  |  |
| ENSBTAG00000006130 | ENSBTAG00000008122 |  |  |
| ENSBTAG00000006132 | ENSBTAG00000008126 |  |  |
| ENSBTAG00000006136 | ENSBTAG00000008127 |  |  |
| ENSBTAG00000006139 | ENSBTAG00000008130 |  |  |
| ENSBTAG00000006140 | ENSBTAG00000008132 |  |  |
| ENSBTAG00000006142 | ENSBTAG00000008133 |  |  |
| ENSBTAG00000006143 | ENSBTAG00000008134 |  |  |
| ENSBTAG00000006160 | ENSBTAG00000008139 |  |  |
| ENSBTAG00000006161 | ENSBTAG00000008140 |  |  |
| ENSBTAG00000006162 | ENSBTAG00000008143 |  |  |
| ENSBTAG00000006166 | ENSBTAG00000008147 |  |  |
| ENSBTAG00000006167 | ENSBTAG00000008150 |  |  |
| ENSBTAG00000006170 | ENSBTAG00000008153 |  |  |
| ENSBTAG00000006173 | ENSBTAG00000008154 |  |  |
| ENSBTAG00000006177 | ENSBTAG00000008159 |  |  |
| ENSBTAG00000006185 | ENSBTAG00000008167 |  |  |
| ENSBTAG00000006187 | ENSBTAG00000008169 |  |  |
| ENSBTAG00000006188 | ENSBTAG00000008172 |  |  |
| ENSBTAG00000006189 | ENSBTAG00000008176 |  |  |
| ENSBTAG00000006193 | ENSBTAG00000008180 |  |  |
| ENSBTAG00000006195 | ENSBTAG00000008181 |  |  |
| ENSBTAG00000006197 | ENSBTAG00000008182 |  |  |
| ENSBTAG00000006200 | ENSBTAG00000008190 |  |  |
| ENSBTAG00000006202 | ENSBTAG00000008191 |  |  |
| ENSBTAG00000006208 | ENSBTAG00000008192 |  |  |
| ENSBTAG00000006213 | ENSBTAG00000008195 |  |  |
| ENSBTAG00000006214 | ENSBTAG00000008202 |  |  |
| ENSBTAG00000006222 | ENSBTAG00000008216 |  |  |
| ENSBTAG00000006225 | ENSBTAG00000008219 |  |  |
| ENSBTAG00000006227 | ENSBTAG00000008224 |  |  |
| ENSBTAG00000006234 | ENSBTAG00000008237 |  |  |
| ENSBTAG00000006235 | ENSBTAG00000008248 |  |  |
| ENSBTAG00000006240 | ENSBTAG00000008250 |  |  |
| ENSBTAG00000006241 | ENSBTAG00000008260 |  |  |
| ENSBTAG00000006244 | ENSBTAG00000008271 |  |  |
| ENSBTAG00000006245 | ENSBTAG00000008278 |  |  |
| ENSBTAG00000006247 | ENSBTAG00000008279 |  |  |
| ENSBTAG00000006250 | ENSBTAG00000008280 |  |  |
| ENSBTAG00000006255 | ENSBTAG00000008285 |  |  |
| ENSBTAG00000006259 | ENSBTAG00000008287 |  |  |
| ENSBTAG00000006261 | ENSBTAG00000008288 |  |  |
| ENSBTAG00000006262 | ENSBTAG00000008291 |  |  |
| ENSBTAG00000006263 | ENSBTAG00000008292 |  |  |
| ENSBTAG00000006264 | ENSBTAG00000008294 |  |  |
| ENSBTAG00000006268 | ENSBTAG00000008296 |  |  |
| ENSBTAG00000006270 | ENSBTAG00000008299 |  |  |
| ENSBTAG00000006272 | ENSBTAG00000008300 |  |  |
| ENSBTAG00000006273 | ENSBTAG00000008303 |  |  |
| ENSBTAG00000006276 | ENSBTAG00000008306 |  |  |
| ENSBTAG00000006280 | ENSBTAG00000008309 |  |  |
| ENSBTAG00000006282 | ENSBTAG00000008310 |  |  |
| ENSBTAG00000006287 | ENSBTAG00000008313 |  |  |
| ENSBTAG00000006288 | ENSBTAG00000008314 |  |  |
| ENSBTAG00000006293 | ENSBTAG00000008320 |  |  |
| ENSBTAG00000006296 | ENSBTAG00000008321 |  |  |
| ENSBTAG00000006304 | ENSBTAG00000008323 |  |  |
| ENSBTAG00000006307 | ENSBTAG00000008329 |  |  |
| ENSBTAG00000006320 | ENSBTAG00000008331 |  |  |
| ENSBTAG00000006322 | ENSBTAG00000008332 |  |  |
| ENSBTAG00000006323 | ENSBTAG00000008336 |  |  |
| ENSBTAG00000006324 | ENSBTAG00000008339 |  |  |
| ENSBTAG00000006325 | ENSBTAG00000008341 |  |  |
| ENSBTAG00000006326 | ENSBTAG00000008342 |  |  |
| ENSBTAG00000006330 | ENSBTAG00000008343 |  |  |
| ENSBTAG00000006345 | ENSBTAG00000008350 |  |  |
| ENSBTAG00000006346 | ENSBTAG00000008353 |  |  |
| ENSBTAG00000006349 | ENSBTAG00000008361 |  |  |
| ENSBTAG00000006350 | ENSBTAG00000008362 |  |  |
| ENSBTAG00000006353 | ENSBTAG00000008367 |  |  |
| ENSBTAG00000006355 | ENSBTAG00000008371 |  |  |
| ENSBTAG00000006359 | ENSBTAG00000008380 |  |  |
| ENSBTAG00000006364 | ENSBTAG00000008382 |  |  |
| ENSBTAG00000006366 | ENSBTAG00000008395 |  |  |
| ENSBTAG00000006367 | ENSBTAG00000008396 |  |  |
| ENSBTAG00000006370 | ENSBTAG00000008401 |  |  |
| ENSBTAG00000006371 | ENSBTAG00000008410 |  |  |
| ENSBTAG00000006373 | ENSBTAG00000008411 |  |  |
| ENSBTAG00000006374 | ENSBTAG00000008414 |  |  |
| ENSBTAG00000006377 | ENSBTAG00000008416 |  |  |
| ENSBTAG00000006378 | ENSBTAG00000008417 |  |  |
| ENSBTAG00000006379 | ENSBTAG00000008423 |  |  |
| ENSBTAG00000006385 | ENSBTAG00000008424 |  |  |
| ENSBTAG00000006392 | ENSBTAG00000008433 |  |  |
| ENSBTAG00000006396 | ENSBTAG00000008435 |  |  |
| ENSBTAG00000006405 | ENSBTAG00000008436 |  |  |
| ENSBTAG00000006410 | ENSBTAG00000008438 |  |  |
| ENSBTAG00000006411 | ENSBTAG00000008439 |  |  |
| ENSBTAG00000006414 | ENSBTAG00000008442 |  |  |
| ENSBTAG00000006416 | ENSBTAG00000008448 |  |  |
| ENSBTAG00000006417 | ENSBTAG00000008452 |  |  |
| ENSBTAG00000006422 | ENSBTAG00000008457 |  |  |
| ENSBTAG00000006423 | ENSBTAG00000008461 |  |  |
| ENSBTAG00000006429 | ENSBTAG00000008466 |  |  |
| ENSBTAG00000006432 | ENSBTAG00000008471 |  |  |
| ENSBTAG00000006434 | ENSBTAG00000008479 |  |  |
| ENSBTAG00000006439 | ENSBTAG00000008482 |  |  |
| ENSBTAG00000006441 | ENSBTAG00000008483 |  |  |
| ENSBTAG00000006451 | ENSBTAG00000008484 |  |  |
| ENSBTAG00000006452 | ENSBTAG00000008485 |  |  |
| ENSBTAG00000006464 | ENSBTAG00000008490 |  |  |
| ENSBTAG00000006466 | ENSBTAG00000008492 |  |  |
| ENSBTAG00000006471 | ENSBTAG00000008493 |  |  |
| ENSBTAG00000006479 | ENSBTAG00000008497 |  |  |
| ENSBTAG00000006481 | ENSBTAG00000008504 |  |  |
| ENSBTAG00000006482 | ENSBTAG00000008509 |  |  |
| ENSBTAG00000006487 | ENSBTAG00000008517 |  |  |
| ENSBTAG00000006489 | ENSBTAG00000008523 |  |  |
| ENSBTAG00000006490 | ENSBTAG00000008525 |  |  |
| ENSBTAG00000006491 | ENSBTAG00000008527 |  |  |
| ENSBTAG00000006492 | ENSBTAG00000008528 |  |  |
| ENSBTAG00000006493 | ENSBTAG00000008538 |  |  |
| ENSBTAG00000006495 | ENSBTAG00000008539 |  |  |
| ENSBTAG00000006499 | ENSBTAG00000008540 |  |  |
| ENSBTAG00000006504 | ENSBTAG00000008541 |  |  |
| ENSBTAG00000006505 | ENSBTAG00000008543 |  |  |
| ENSBTAG00000006506 | ENSBTAG00000008545 |  |  |
| ENSBTAG00000006510 | ENSBTAG00000008550 |  |  |
| ENSBTAG00000006511 | ENSBTAG00000008553 |  |  |
| ENSBTAG00000006517 | ENSBTAG00000008554 |  |  |
| ENSBTAG00000006525 | ENSBTAG00000008555 |  |  |
| ENSBTAG00000006526 | ENSBTAG00000008562 |  |  |
| ENSBTAG00000006529 | ENSBTAG00000008571 |  |  |
| ENSBTAG00000006534 | ENSBTAG00000008573 |  |  |
| ENSBTAG00000006536 | ENSBTAG00000008577 |  |  |
| ENSBTAG00000006538 | ENSBTAG00000008578 |  |  |
| ENSBTAG00000006542 | ENSBTAG00000008579 |  |  |
| ENSBTAG00000006546 | ENSBTAG00000008591 |  |  |
| ENSBTAG00000006550 | ENSBTAG00000008595 |  |  |
| ENSBTAG00000006551 | ENSBTAG00000008596 |  |  |
| ENSBTAG00000006552 | ENSBTAG00000008600 |  |  |
| ENSBTAG00000006556 | ENSBTAG00000008603 |  |  |
| ENSBTAG00000006561 | ENSBTAG00000008606 |  |  |
| ENSBTAG00000006563 | ENSBTAG00000008611 |  |  |
| ENSBTAG00000006564 | ENSBTAG00000008619 |  |  |
| ENSBTAG00000006567 | ENSBTAG00000008621 |  |  |
| ENSBTAG00000006573 | ENSBTAG00000008624 |  |  |
| ENSBTAG00000006578 | ENSBTAG00000008625 |  |  |
| ENSBTAG00000006579 | ENSBTAG00000008629 |  |  |
| ENSBTAG00000006586 | ENSBTAG00000008636 |  |  |
| ENSBTAG00000006587 | ENSBTAG00000008642 |  |  |
| ENSBTAG00000006588 | ENSBTAG00000008645 |  |  |
| ENSBTAG00000006589 | ENSBTAG00000008646 |  |  |
| ENSBTAG00000006590 | ENSBTAG00000008652 |  |  |
| ENSBTAG00000006592 | ENSBTAG00000008664 |  |  |
| ENSBTAG00000006593 | ENSBTAG00000008674 |  |  |
| ENSBTAG00000006595 | ENSBTAG00000008683 |  |  |
| ENSBTAG00000006607 | ENSBTAG00000008686 |  |  |
| ENSBTAG00000006609 | ENSBTAG00000008688 |  |  |
| ENSBTAG00000006610 | ENSBTAG00000008690 |  |  |
| ENSBTAG00000006614 | ENSBTAG00000008693 |  |  |
| ENSBTAG00000006615 | ENSBTAG00000008699 |  |  |
| ENSBTAG00000006616 | ENSBTAG00000008703 |  |  |
| ENSBTAG00000006620 | ENSBTAG00000008705 |  |  |
| ENSBTAG00000006630 | ENSBTAG00000008711 |  |  |
| ENSBTAG00000006635 | ENSBTAG00000008716 |  |  |
| ENSBTAG00000006639 | ENSBTAG00000008717 |  |  |
| ENSBTAG00000006640 | ENSBTAG00000008718 |  |  |
| ENSBTAG00000006643 | ENSBTAG00000008720 |  |  |
| ENSBTAG00000006644 | ENSBTAG00000008726 |  |  |
| ENSBTAG00000006645 | ENSBTAG00000008728 |  |  |
| ENSBTAG00000006647 | ENSBTAG00000008729 |  |  |
| ENSBTAG00000006650 | ENSBTAG00000008730 |  |  |
| ENSBTAG00000006654 | ENSBTAG00000008731 |  |  |
| ENSBTAG00000006656 | ENSBTAG00000008733 |  |  |
| ENSBTAG00000006657 | ENSBTAG00000008734 |  |  |
| ENSBTAG00000006662 | ENSBTAG00000008737 |  |  |
| ENSBTAG00000006665 | ENSBTAG00000008743 |  |  |
| ENSBTAG00000006666 | ENSBTAG00000008744 |  |  |
| ENSBTAG00000006674 | ENSBTAG00000008755 |  |  |
| ENSBTAG00000006675 | ENSBTAG00000008756 |  |  |
| ENSBTAG00000006676 | ENSBTAG00000008759 |  |  |
| ENSBTAG00000006680 | ENSBTAG00000008764 |  |  |
| ENSBTAG00000006686 | ENSBTAG00000008765 |  |  |
| ENSBTAG00000006688 | ENSBTAG00000008773 |  |  |
| ENSBTAG00000006689 | ENSBTAG00000008777 |  |  |
| ENSBTAG00000006690 | ENSBTAG00000008788 |  |  |
| ENSBTAG00000006694 | ENSBTAG00000008794 |  |  |
| ENSBTAG00000006697 | ENSBTAG00000008800 |  |  |
| ENSBTAG00000006702 | ENSBTAG00000008802 |  |  |
| ENSBTAG00000006703 | ENSBTAG00000008805 |  |  |
| ENSBTAG00000006704 | ENSBTAG00000008807 |  |  |
| ENSBTAG00000006707 | ENSBTAG00000008808 |  |  |
| ENSBTAG00000006708 | ENSBTAG00000008812 |  |  |
| ENSBTAG00000006712 | ENSBTAG00000008815 |  |  |
| ENSBTAG00000006713 | ENSBTAG00000008816 |  |  |
| ENSBTAG00000006714 | ENSBTAG00000008819 |  |  |
| ENSBTAG00000006715 | ENSBTAG00000008825 |  |  |
| ENSBTAG00000006716 | ENSBTAG00000008826 |  |  |
| ENSBTAG00000006718 | ENSBTAG00000008827 |  |  |
| ENSBTAG00000006719 | ENSBTAG00000008840 |  |  |
| ENSBTAG00000006720 | ENSBTAG00000008841 |  |  |
| ENSBTAG00000006721 | ENSBTAG00000008842 |  |  |
| ENSBTAG00000006726 | ENSBTAG00000008845 |  |  |
| ENSBTAG00000006730 | ENSBTAG00000008849 |  |  |
| ENSBTAG00000006731 | ENSBTAG00000008853 |  |  |
| ENSBTAG00000006735 | ENSBTAG00000008854 |  |  |
| ENSBTAG00000006738 | ENSBTAG00000008862 |  |  |
| ENSBTAG00000006739 | ENSBTAG00000008863 |  |  |
| ENSBTAG00000006740 | ENSBTAG00000008868 |  |  |
| ENSBTAG00000006745 | ENSBTAG00000008880 |  |  |
| ENSBTAG00000006747 | ENSBTAG00000008884 |  |  |
| ENSBTAG00000006752 | ENSBTAG00000008887 |  |  |
| ENSBTAG00000006753 | ENSBTAG00000008895 |  |  |
| ENSBTAG00000006755 | ENSBTAG00000008897 |  |  |
| ENSBTAG00000006756 | ENSBTAG00000008902 |  |  |
| ENSBTAG00000006759 | ENSBTAG00000008915 |  |  |
| ENSBTAG00000006761 | ENSBTAG00000008916 |  |  |
| ENSBTAG00000006765 | ENSBTAG00000008920 |  |  |
| ENSBTAG00000006767 | ENSBTAG00000008925 |  |  |
| ENSBTAG00000006769 | ENSBTAG00000008926 |  |  |
| ENSBTAG00000006775 | ENSBTAG00000008931 |  |  |
| ENSBTAG00000006776 | ENSBTAG00000008935 |  |  |
| ENSBTAG00000006777 | ENSBTAG00000008937 |  |  |
| ENSBTAG00000006779 | ENSBTAG00000008939 |  |  |
| ENSBTAG00000006784 | ENSBTAG00000008940 |  |  |
| ENSBTAG00000006786 | ENSBTAG00000008943 |  |  |
| ENSBTAG00000006789 | ENSBTAG00000008946 |  |  |
| ENSBTAG00000006790 | ENSBTAG00000008947 |  |  |
| ENSBTAG00000006792 | ENSBTAG00000008951 |  |  |
| ENSBTAG00000006795 | ENSBTAG00000008952 |  |  |
| ENSBTAG00000006797 | ENSBTAG00000008956 |  |  |
| ENSBTAG00000006801 | ENSBTAG00000008958 |  |  |
| ENSBTAG00000006804 | ENSBTAG00000008964 |  |  |
| ENSBTAG00000006805 | ENSBTAG00000008966 |  |  |
| ENSBTAG00000006806 | ENSBTAG00000008967 |  |  |
| ENSBTAG00000006807 | ENSBTAG00000008973 |  |  |
| ENSBTAG00000006810 | ENSBTAG00000008978 |  |  |
| ENSBTAG00000006812 | ENSBTAG00000008985 |  |  |
| ENSBTAG00000006816 | ENSBTAG00000008987 |  |  |
| ENSBTAG00000006818 | ENSBTAG00000008993 |  |  |
| ENSBTAG00000006820 | ENSBTAG00000008998 |  |  |
| ENSBTAG00000006828 | ENSBTAG00000009012 |  |  |
| ENSBTAG00000006832 | ENSBTAG00000009014 |  |  |
| ENSBTAG00000006835 | ENSBTAG00000009021 |  |  |
| ENSBTAG00000006837 | ENSBTAG00000009022 |  |  |
| ENSBTAG00000006843 | ENSBTAG00000009024 |  |  |
| ENSBTAG00000006844 | ENSBTAG00000009034 |  |  |
| ENSBTAG00000006846 | ENSBTAG00000009035 |  |  |
| ENSBTAG00000006852 | ENSBTAG00000009036 |  |  |
| ENSBTAG00000006860 | ENSBTAG00000009037 |  |  |
| ENSBTAG00000006862 | ENSBTAG00000009048 |  |  |
| ENSBTAG00000006877 | ENSBTAG00000009049 |  |  |
| ENSBTAG00000006879 | ENSBTAG00000009050 |  |  |
| ENSBTAG00000006881 | ENSBTAG00000009051 |  |  |
| ENSBTAG00000006884 | ENSBTAG00000009055 |  |  |
| ENSBTAG00000006886 | ENSBTAG00000009059 |  |  |
| ENSBTAG00000006893 | ENSBTAG00000009061 |  |  |
| ENSBTAG00000006896 | ENSBTAG00000009065 |  |  |
| ENSBTAG00000006899 | ENSBTAG00000009067 |  |  |
| ENSBTAG00000006901 | ENSBTAG00000009075 |  |  |
| ENSBTAG00000006904 | ENSBTAG00000009076 |  |  |
| ENSBTAG00000006914 | ENSBTAG00000009079 |  |  |
| ENSBTAG00000006918 | ENSBTAG00000009085 |  |  |
| ENSBTAG00000006919 | ENSBTAG00000009087 |  |  |
| ENSBTAG00000006921 | ENSBTAG00000009091 |  |  |
| ENSBTAG00000006925 | ENSBTAG00000009097 |  |  |
| ENSBTAG00000006928 | ENSBTAG00000009098 |  |  |
| ENSBTAG00000006933 | ENSBTAG00000009101 |  |  |
| ENSBTAG00000006934 | ENSBTAG00000009103 |  |  |
| ENSBTAG00000006936 | ENSBTAG00000009106 |  |  |
| ENSBTAG00000006939 | ENSBTAG00000009115 |  |  |
| ENSBTAG00000006940 | ENSBTAG00000009121 |  |  |
| ENSBTAG00000006941 | ENSBTAG00000009127 |  |  |
| ENSBTAG00000006945 | ENSBTAG00000009129 |  |  |
| ENSBTAG00000006948 | ENSBTAG00000009132 |  |  |
| ENSBTAG00000006951 | ENSBTAG00000009137 |  |  |
| ENSBTAG00000006955 | ENSBTAG00000009141 |  |  |
| ENSBTAG00000006957 | ENSBTAG00000009152 |  |  |
| ENSBTAG00000006960 | ENSBTAG00000009153 |  |  |
| ENSBTAG00000006962 | ENSBTAG00000009156 |  |  |
| ENSBTAG00000006966 | ENSBTAG00000009163 |  |  |
| ENSBTAG00000006969 | ENSBTAG00000009167 |  |  |
| ENSBTAG00000006970 | ENSBTAG00000009172 |  |  |
| ENSBTAG00000006971 | ENSBTAG00000009174 |  |  |
| ENSBTAG00000006972 | ENSBTAG00000009176 |  |  |
| ENSBTAG00000006977 | ENSBTAG00000009180 |  |  |
| ENSBTAG00000006982 | ENSBTAG00000009182 |  |  |
| ENSBTAG00000006984 | ENSBTAG00000009183 |  |  |
| ENSBTAG00000006985 | ENSBTAG00000009188 |  |  |
| ENSBTAG00000006987 | ENSBTAG00000009191 |  |  |
| ENSBTAG00000006989 | ENSBTAG00000009194 |  |  |
| ENSBTAG00000006995 | ENSBTAG00000009198 |  |  |
| ENSBTAG00000006998 | ENSBTAG00000009207 |  |  |
| ENSBTAG00000006999 | ENSBTAG00000009208 |  |  |
| ENSBTAG00000007003 | ENSBTAG00000009210 |  |  |
| ENSBTAG00000007007 | ENSBTAG00000009213 |  |  |
| ENSBTAG00000007008 | ENSBTAG00000009214 |  |  |
| ENSBTAG00000007012 | ENSBTAG00000009216 |  |  |
| ENSBTAG00000007013 | ENSBTAG00000009218 |  |  |
| ENSBTAG00000007014 | ENSBTAG00000009231 |  |  |
| ENSBTAG00000007016 | ENSBTAG00000009233 |  |  |
| ENSBTAG00000007020 | ENSBTAG00000009236 |  |  |
| ENSBTAG00000007035 | ENSBTAG00000009237 |  |  |
| ENSBTAG00000007036 | ENSBTAG00000009245 |  |  |
| ENSBTAG00000007039 | ENSBTAG00000009251 |  |  |
| ENSBTAG00000007047 | ENSBTAG00000009252 |  |  |
| ENSBTAG00000007065 | ENSBTAG00000009256 |  |  |
| ENSBTAG00000007066 | ENSBTAG00000009260 |  |  |
| ENSBTAG00000007068 | ENSBTAG00000009263 |  |  |
| ENSBTAG00000007071 | ENSBTAG00000009265 |  |  |
| ENSBTAG00000007073 | ENSBTAG00000009271 |  |  |
| ENSBTAG00000007074 | ENSBTAG00000009275 |  |  |
| ENSBTAG00000007080 | ENSBTAG00000009279 |  |  |
| ENSBTAG00000007084 | ENSBTAG00000009281 |  |  |
| ENSBTAG00000007093 | ENSBTAG00000009284 |  |  |
| ENSBTAG00000007094 | ENSBTAG00000009287 |  |  |
| ENSBTAG00000007096 | ENSBTAG00000009290 |  |  |
| ENSBTAG00000007097 | ENSBTAG00000009291 |  |  |
| ENSBTAG00000007099 | ENSBTAG00000009292 |  |  |
| ENSBTAG00000007100 | ENSBTAG00000009293 |  |  |
| ENSBTAG00000007101 | ENSBTAG00000009297 |  |  |
| ENSBTAG00000007103 | ENSBTAG00000009299 |  |  |
| ENSBTAG00000007104 | ENSBTAG00000009304 |  |  |
| ENSBTAG00000007106 | ENSBTAG00000009305 |  |  |
| ENSBTAG00000007107 | ENSBTAG00000009307 |  |  |
| ENSBTAG00000007108 | ENSBTAG00000009309 |  |  |
| ENSBTAG00000007109 | ENSBTAG00000009328 |  |  |
| ENSBTAG00000007110 | ENSBTAG00000009330 |  |  |
| ENSBTAG00000007112 | ENSBTAG00000009337 |  |  |
| ENSBTAG00000007113 | ENSBTAG00000009338 |  |  |
| ENSBTAG00000007115 | ENSBTAG00000009345 |  |  |
| ENSBTAG00000007116 | ENSBTAG00000009348 |  |  |
| ENSBTAG00000007117 | ENSBTAG00000009353 |  |  |
| ENSBTAG00000007118 | ENSBTAG00000009354 |  |  |
| ENSBTAG00000007121 | ENSBTAG00000009355 |  |  |
| ENSBTAG00000007122 | ENSBTAG00000009362 |  |  |
| ENSBTAG00000007123 | ENSBTAG00000009363 |  |  |
| ENSBTAG00000007125 | ENSBTAG00000009368 |  |  |
| ENSBTAG00000007128 | ENSBTAG00000009372 |  |  |
| ENSBTAG00000007129 | ENSBTAG00000009381 |  |  |
| ENSBTAG00000007130 | ENSBTAG00000009383 |  |  |
| ENSBTAG00000007131 | ENSBTAG00000009384 |  |  |
| ENSBTAG00000007133 | ENSBTAG00000009387 |  |  |
| ENSBTAG00000007137 | ENSBTAG00000009389 |  |  |
| ENSBTAG00000007139 | ENSBTAG00000009392 |  |  |
| ENSBTAG00000007141 | ENSBTAG00000009393 |  |  |
| ENSBTAG00000007142 | ENSBTAG00000009414 |  |  |
| ENSBTAG00000007145 | ENSBTAG00000009417 |  |  |
| ENSBTAG00000007147 | ENSBTAG00000009421 |  |  |
| ENSBTAG00000007148 | ENSBTAG00000009428 |  |  |
| ENSBTAG00000007152 | ENSBTAG00000009431 |  |  |
| ENSBTAG00000007153 | ENSBTAG00000009435 |  |  |
| ENSBTAG00000007158 | ENSBTAG00000009436 |  |  |
| ENSBTAG00000007164 | ENSBTAG00000009439 |  |  |
| ENSBTAG00000007167 | ENSBTAG00000009443 |  |  |
| ENSBTAG00000007169 | ENSBTAG00000009446 |  |  |
| ENSBTAG00000007170 | ENSBTAG00000009451 |  |  |
| ENSBTAG00000007172 | ENSBTAG00000009453 |  |  |
| ENSBTAG00000007173 | ENSBTAG00000009455 |  |  |
| ENSBTAG00000007176 | ENSBTAG00000009458 |  |  |
| ENSBTAG00000007177 | ENSBTAG00000009459 |  |  |
| ENSBTAG00000007181 | ENSBTAG00000009470 |  |  |
| ENSBTAG00000007184 | ENSBTAG00000009471 |  |  |
| ENSBTAG00000007189 | ENSBTAG00000009474 |  |  |
| ENSBTAG00000007190 | ENSBTAG00000009475 |  |  |
| ENSBTAG00000007191 | ENSBTAG00000009476 |  |  |
| ENSBTAG00000007192 | ENSBTAG00000009477 |  |  |
| ENSBTAG00000007193 | ENSBTAG00000009478 |  |  |
| ENSBTAG00000007194 | ENSBTAG00000009481 |  |  |
| ENSBTAG00000007196 | ENSBTAG00000009486 |  |  |
| ENSBTAG00000007201 | ENSBTAG00000009487 |  |  |
| ENSBTAG00000007202 | ENSBTAG00000009490 |  |  |
| ENSBTAG00000007203 | ENSBTAG00000009495 |  |  |
| ENSBTAG00000007204 | ENSBTAG00000009504 |  |  |
| ENSBTAG00000007213 | ENSBTAG00000009506 |  |  |
| ENSBTAG00000007214 | ENSBTAG00000009507 |  |  |
| ENSBTAG00000007215 | ENSBTAG00000009513 |  |  |
| ENSBTAG00000007216 | ENSBTAG00000009514 |  |  |
| ENSBTAG00000007217 | ENSBTAG00000009516 |  |  |
| ENSBTAG00000007220 | ENSBTAG00000009517 |  |  |
| ENSBTAG00000007223 | ENSBTAG00000009518 |  |  |
| ENSBTAG00000007230 | ENSBTAG00000009522 |  |  |
| ENSBTAG00000007234 | ENSBTAG00000009523 |  |  |
| ENSBTAG00000007235 | ENSBTAG00000009527 |  |  |
| ENSBTAG00000007237 | ENSBTAG00000009535 |  |  |
| ENSBTAG00000007246 | ENSBTAG00000009541 |  |  |
| ENSBTAG00000007247 | ENSBTAG00000009542 |  |  |
| ENSBTAG00000007253 | ENSBTAG00000009543 |  |  |
| ENSBTAG00000007258 | ENSBTAG00000009549 |  |  |
| ENSBTAG00000007268 | ENSBTAG00000009552 |  |  |
| ENSBTAG00000007269 | ENSBTAG00000009554 |  |  |
| ENSBTAG00000007270 | ENSBTAG00000009560 |  |  |
| ENSBTAG00000007272 | ENSBTAG00000009565 |  |  |
| ENSBTAG00000007275 | ENSBTAG00000009574 |  |  |
| ENSBTAG00000007299 | ENSBTAG00000009578 |  |  |
| ENSBTAG00000007300 | ENSBTAG00000009580 |  |  |
| ENSBTAG00000007303 | ENSBTAG00000009584 |  |  |
| ENSBTAG00000007304 | ENSBTAG00000009586 |  |  |
| ENSBTAG00000007305 | ENSBTAG00000009596 |  |  |
| ENSBTAG00000007306 | ENSBTAG00000009600 |  |  |
| ENSBTAG00000007307 | ENSBTAG00000009602 |  |  |
| ENSBTAG00000007308 | ENSBTAG00000009603 |  |  |
| ENSBTAG00000007309 | ENSBTAG00000009607 |  |  |
| ENSBTAG00000007315 | ENSBTAG00000009611 |  |  |
| ENSBTAG00000007316 | ENSBTAG00000009615 |  |  |
| ENSBTAG00000007320 | ENSBTAG00000009617 |  |  |
| ENSBTAG00000007321 | ENSBTAG00000009624 |  |  |
| ENSBTAG00000007330 | ENSBTAG00000009635 |  |  |
| ENSBTAG00000007331 | ENSBTAG00000009636 |  |  |
| ENSBTAG00000007334 | ENSBTAG00000009639 |  |  |
| ENSBTAG00000007335 | ENSBTAG00000009646 |  |  |
| ENSBTAG00000007343 | ENSBTAG00000009647 |  |  |
| ENSBTAG00000007346 | ENSBTAG00000009649 |  |  |
| ENSBTAG00000007347 | ENSBTAG00000009653 |  |  |
| ENSBTAG00000007348 | ENSBTAG00000009654 |  |  |
| ENSBTAG00000007352 | ENSBTAG00000009657 |  |  |
| ENSBTAG00000007356 | ENSBTAG00000009661 |  |  |
| ENSBTAG00000007357 | ENSBTAG00000009663 |  |  |
| ENSBTAG00000007360 | ENSBTAG00000009668 |  |  |
| ENSBTAG00000007363 | ENSBTAG00000009676 |  |  |
| ENSBTAG00000007365 | ENSBTAG00000009680 |  |  |
| ENSBTAG00000007367 | ENSBTAG00000009685 |  |  |
| ENSBTAG00000007369 | ENSBTAG00000009687 |  |  |
| ENSBTAG00000007370 | ENSBTAG00000009698 |  |  |
| ENSBTAG00000007371 | ENSBTAG00000009707 |  |  |
| ENSBTAG00000007374 | ENSBTAG00000009711 |  |  |
| ENSBTAG00000007383 | ENSBTAG00000009719 |  |  |
| ENSBTAG00000007384 | ENSBTAG00000009726 |  |  |
| ENSBTAG00000007385 | ENSBTAG00000009732 |  |  |
| ENSBTAG00000007386 | ENSBTAG00000009735 |  |  |
| ENSBTAG00000007390 | ENSBTAG00000009736 |  |  |
| ENSBTAG00000007393 | ENSBTAG00000009738 |  |  |
| ENSBTAG00000007394 | ENSBTAG00000009743 |  |  |
| ENSBTAG00000007395 | ENSBTAG00000009746 |  |  |
| ENSBTAG00000007397 | ENSBTAG00000009747 |  |  |
| ENSBTAG00000007399 | ENSBTAG00000009748 |  |  |
| ENSBTAG00000007402 | ENSBTAG00000009750 |  |  |
| ENSBTAG00000007403 | ENSBTAG00000009755 |  |  |
| ENSBTAG00000007414 | ENSBTAG00000009761 |  |  |
| ENSBTAG00000007415 | ENSBTAG00000009765 |  |  |
| ENSBTAG00000007422 | ENSBTAG00000009768 |  |  |
| ENSBTAG00000007423 | ENSBTAG00000009770 |  |  |
| ENSBTAG00000007424 | ENSBTAG00000009772 |  |  |
| ENSBTAG00000007434 | ENSBTAG00000009777 |  |  |
| ENSBTAG00000007435 | ENSBTAG00000009778 |  |  |
| ENSBTAG00000007436 | ENSBTAG00000009780 |  |  |
| ENSBTAG00000007442 | ENSBTAG00000009784 |  |  |
| ENSBTAG00000007444 | ENSBTAG00000009796 |  |  |
| ENSBTAG00000007446 | ENSBTAG00000009798 |  |  |
| ENSBTAG00000007447 | ENSBTAG00000009800 |  |  |
| ENSBTAG00000007449 | ENSBTAG00000009803 |  |  |
| ENSBTAG00000007450 | ENSBTAG00000009806 |  |  |
| ENSBTAG00000007460 | ENSBTAG00000009813 |  |  |
| ENSBTAG00000007474 | ENSBTAG00000009819 |  |  |
| ENSBTAG00000007479 | ENSBTAG00000009830 |  |  |
| ENSBTAG00000007480 | ENSBTAG00000009831 |  |  |
| ENSBTAG00000007484 | ENSBTAG00000009832 |  |  |
| ENSBTAG00000007485 | ENSBTAG00000009834 |  |  |
| ENSBTAG00000007490 | ENSBTAG00000009835 |  |  |
| ENSBTAG00000007492 | ENSBTAG00000009837 |  |  |
| ENSBTAG00000007494 | ENSBTAG00000009841 |  |  |
| ENSBTAG00000007496 | ENSBTAG00000009842 |  |  |
| ENSBTAG00000007497 | ENSBTAG00000009844 |  |  |
| ENSBTAG00000007498 | ENSBTAG00000009850 |  |  |
| ENSBTAG00000007499 | ENSBTAG00000009851 |  |  |
| ENSBTAG00000007502 | ENSBTAG00000009855 |  |  |
| ENSBTAG00000007503 | ENSBTAG00000009863 |  |  |
| ENSBTAG00000007507 | ENSBTAG00000009867 |  |  |
| ENSBTAG00000007510 | ENSBTAG00000009870 |  |  |
| ENSBTAG00000007512 | ENSBTAG00000009873 |  |  |
| ENSBTAG00000007513 | ENSBTAG00000009874 |  |  |
| ENSBTAG00000007515 | ENSBTAG00000009881 |  |  |
| ENSBTAG00000007519 | ENSBTAG00000009886 |  |  |
| ENSBTAG00000007523 | ENSBTAG00000009888 |  |  |
| ENSBTAG00000007531 | ENSBTAG00000009889 |  |  |
| ENSBTAG00000007534 | ENSBTAG00000009902 |  |  |
| ENSBTAG00000007537 | ENSBTAG00000009903 |  |  |
| ENSBTAG00000007540 | ENSBTAG00000009904 |  |  |
| ENSBTAG00000007544 | ENSBTAG00000009905 |  |  |
| ENSBTAG00000007547 | ENSBTAG00000009906 |  |  |
| ENSBTAG00000007553 | ENSBTAG00000009914 |  |  |
| ENSBTAG00000007554 | ENSBTAG00000009915 |  |  |
| ENSBTAG00000007559 | ENSBTAG00000009916 |  |  |
| ENSBTAG00000007567 | ENSBTAG00000009918 |  |  |
| ENSBTAG00000007569 | ENSBTAG00000009923 |  |  |
| ENSBTAG00000007570 | ENSBTAG00000009928 |  |  |
| ENSBTAG00000007577 | ENSBTAG00000009942 |  |  |
| ENSBTAG00000007578 | ENSBTAG00000009948 |  |  |
| ENSBTAG00000007581 | ENSBTAG00000009956 |  |  |
| ENSBTAG00000007583 | ENSBTAG00000009960 |  |  |
| ENSBTAG00000007586 | ENSBTAG00000009964 |  |  |
| ENSBTAG00000007588 | ENSBTAG00000009966 |  |  |
| ENSBTAG00000007589 | ENSBTAG00000009978 |  |  |
| ENSBTAG00000007591 | ENSBTAG00000009982 |  |  |
| ENSBTAG00000007592 | ENSBTAG00000009983 |  |  |
| ENSBTAG00000007594 | ENSBTAG00000009984 |  |  |
| ENSBTAG00000007596 | ENSBTAG00000009995 |  |  |
| ENSBTAG00000007599 | ENSBTAG00000009997 |  |  |
| ENSBTAG00000007602 | ENSBTAG00000010001 |  |  |
| ENSBTAG00000007606 | ENSBTAG00000010006 |  |  |
| ENSBTAG00000007609 | ENSBTAG00000010009 |  |  |
| ENSBTAG00000007614 | ENSBTAG00000010013 |  |  |
| ENSBTAG00000007618 | ENSBTAG00000010023 |  |  |
| ENSBTAG00000007619 | ENSBTAG00000010030 |  |  |
| ENSBTAG00000007622 | ENSBTAG00000010036 |  |  |
| ENSBTAG00000007623 | ENSBTAG00000010042 |  |  |
| ENSBTAG00000007624 | ENSBTAG00000010046 |  |  |
| ENSBTAG00000007626 | ENSBTAG00000010048 |  |  |
| ENSBTAG00000007632 | ENSBTAG00000010050 |  |  |
| ENSBTAG00000007634 | ENSBTAG00000010059 |  |  |
| ENSBTAG00000007639 | ENSBTAG00000010060 |  |  |
| ENSBTAG00000007642 | ENSBTAG00000010063 |  |  |
| ENSBTAG00000007644 | ENSBTAG00000010068 |  |  |
| ENSBTAG00000007648 | ENSBTAG00000010069 |  |  |
| ENSBTAG00000007651 | ENSBTAG00000010077 |  |  |
| ENSBTAG00000007657 | ENSBTAG00000010082 |  |  |
| ENSBTAG00000007658 | ENSBTAG00000010091 |  |  |
| ENSBTAG00000007659 | ENSBTAG00000010100 |  |  |
| ENSBTAG00000007661 | ENSBTAG00000010101 |  |  |
| ENSBTAG00000007666 | ENSBTAG00000010103 |  |  |
| ENSBTAG00000007668 | ENSBTAG00000010116 |  |  |
| ENSBTAG00000007674 | ENSBTAG00000010120 |  |  |
| ENSBTAG00000007675 | ENSBTAG00000010124 |  |  |
| ENSBTAG00000007678 | ENSBTAG00000010128 |  |  |
| ENSBTAG00000007680 | ENSBTAG00000010130 |  |  |
| ENSBTAG00000007681 | ENSBTAG00000010131 |  |  |
| ENSBTAG00000007683 | ENSBTAG00000010132 |  |  |
| ENSBTAG00000007684 | ENSBTAG00000010134 |  |  |
| ENSBTAG00000007690 | ENSBTAG00000010135 |  |  |
| ENSBTAG00000007695 | ENSBTAG00000010152 |  |  |
| ENSBTAG00000007698 | ENSBTAG00000010155 |  |  |
| ENSBTAG00000007700 | ENSBTAG00000010161 |  |  |
| ENSBTAG00000007703 | ENSBTAG00000010165 |  |  |
| ENSBTAG00000007705 | ENSBTAG00000010170 |  |  |
| ENSBTAG00000007708 | ENSBTAG00000010171 |  |  |
| ENSBTAG00000007709 | ENSBTAG00000010174 |  |  |
| ENSBTAG00000007712 | ENSBTAG00000010177 |  |  |
| ENSBTAG00000007718 | ENSBTAG00000010180 |  |  |
| ENSBTAG00000007719 | ENSBTAG00000010181 |  |  |
| ENSBTAG00000007721 | ENSBTAG00000010184 |  |  |
| ENSBTAG00000007722 | ENSBTAG00000010188 |  |  |
| ENSBTAG00000007725 | ENSBTAG00000010191 |  |  |
| ENSBTAG00000007730 | ENSBTAG00000010193 |  |  |
| ENSBTAG00000007731 | ENSBTAG00000010196 |  |  |
| ENSBTAG00000007732 | ENSBTAG00000010207 |  |  |
| ENSBTAG00000007740 | ENSBTAG00000010210 |  |  |
| ENSBTAG00000007743 | ENSBTAG00000010221 |  |  |
| ENSBTAG00000007755 | ENSBTAG00000010223 |  |  |
| ENSBTAG00000007756 | ENSBTAG00000010227 |  |  |
| ENSBTAG00000007758 | ENSBTAG00000010228 |  |  |
| ENSBTAG00000007759 | ENSBTAG00000010230 |  |  |
| ENSBTAG00000007762 | ENSBTAG00000010234 |  |  |
| ENSBTAG00000007763 | ENSBTAG00000010238 |  |  |
| ENSBTAG00000007766 | ENSBTAG00000010242 |  |  |
| ENSBTAG00000007773 | ENSBTAG00000010243 |  |  |
| ENSBTAG00000007776 | ENSBTAG00000010246 |  |  |
| ENSBTAG00000007777 | ENSBTAG00000010247 |  |  |
| ENSBTAG00000007780 | ENSBTAG00000010248 |  |  |
| ENSBTAG00000007783 | ENSBTAG00000010249 |  |  |
| ENSBTAG00000007784 | ENSBTAG00000010254 |  |  |
| ENSBTAG00000007786 | ENSBTAG00000010265 |  |  |
| ENSBTAG00000007787 | ENSBTAG00000010271 |  |  |
| ENSBTAG00000007794 | ENSBTAG00000010273 |  |  |
| ENSBTAG00000007798 | ENSBTAG00000010277 |  |  |
| ENSBTAG00000007799 | ENSBTAG00000010278 |  |  |
| ENSBTAG00000007802 | ENSBTAG00000010279 |  |  |
| ENSBTAG00000007804 | ENSBTAG00000010284 |  |  |
| ENSBTAG00000007806 | ENSBTAG00000010285 |  |  |
| ENSBTAG00000007808 | ENSBTAG00000010291 |  |  |
| ENSBTAG00000007813 | ENSBTAG00000010292 |  |  |
| ENSBTAG00000007814 | ENSBTAG00000010299 |  |  |
| ENSBTAG00000007817 | ENSBTAG00000010304 |  |  |
| ENSBTAG00000007818 | ENSBTAG00000010313 |  |  |
| ENSBTAG00000007825 | ENSBTAG00000010321 |  |  |
| ENSBTAG00000007828 | ENSBTAG00000010324 |  |  |
| ENSBTAG00000007834 | ENSBTAG00000010326 |  |  |
| ENSBTAG00000007835 | ENSBTAG00000010328 |  |  |
| ENSBTAG00000007837 | ENSBTAG00000010334 |  |  |
| ENSBTAG00000007840 | ENSBTAG00000010336 |  |  |
| ENSBTAG00000007841 | ENSBTAG00000010337 |  |  |
| ENSBTAG00000007843 | ENSBTAG00000010338 |  |  |
| ENSBTAG00000007846 | ENSBTAG00000010347 |  |  |
| ENSBTAG00000007847 | ENSBTAG00000010348 |  |  |
| ENSBTAG00000007855 | ENSBTAG00000010349 |  |  |
| ENSBTAG00000007859 | ENSBTAG00000010350 |  |  |
| ENSBTAG00000007863 | ENSBTAG00000010351 |  |  |
| ENSBTAG00000007865 | ENSBTAG00000010355 |  |  |
| ENSBTAG00000007867 | ENSBTAG00000010356 |  |  |
| ENSBTAG00000007870 | ENSBTAG00000010359 |  |  |
| ENSBTAG00000007871 | ENSBTAG00000010361 |  |  |
| ENSBTAG00000007876 | ENSBTAG00000010362 |  |  |
| ENSBTAG00000007880 | ENSBTAG00000010365 |  |  |
| ENSBTAG00000007884 | ENSBTAG00000010367 |  |  |
| ENSBTAG00000007893 | ENSBTAG00000010370 |  |  |
| ENSBTAG00000007895 | ENSBTAG00000010372 |  |  |
| ENSBTAG00000007896 | ENSBTAG00000010375 |  |  |
| ENSBTAG00000007900 | ENSBTAG00000010376 |  |  |
| ENSBTAG00000007904 | ENSBTAG00000010378 |  |  |
| ENSBTAG00000007906 | ENSBTAG00000010381 |  |  |
| ENSBTAG00000007913 | ENSBTAG00000010383 |  |  |
| ENSBTAG00000007920 | ENSBTAG00000010386 |  |  |
| ENSBTAG00000007921 | ENSBTAG00000010387 |  |  |
| ENSBTAG00000007922 | ENSBTAG00000010390 |  |  |
| ENSBTAG00000007923 | ENSBTAG00000010395 |  |  |
| ENSBTAG00000007927 | ENSBTAG00000010397 |  |  |
| ENSBTAG00000007933 | ENSBTAG00000010401 |  |  |
| ENSBTAG00000007934 | ENSBTAG00000010402 |  |  |
| ENSBTAG00000007935 | ENSBTAG00000010403 |  |  |
| ENSBTAG00000007937 | ENSBTAG00000010419 |  |  |
| ENSBTAG00000007939 | ENSBTAG00000010422 |  |  |
| ENSBTAG00000007942 | ENSBTAG00000010427 |  |  |
| ENSBTAG00000007943 | ENSBTAG00000010431 |  |  |
| ENSBTAG00000007946 | ENSBTAG00000010433 |  |  |
| ENSBTAG00000007952 | ENSBTAG00000010437 |  |  |
| ENSBTAG00000007954 | ENSBTAG00000010442 |  |  |
| ENSBTAG00000007955 | ENSBTAG00000010444 |  |  |
| ENSBTAG00000007958 | ENSBTAG00000010445 |  |  |
| ENSBTAG00000007961 | ENSBTAG00000010447 |  |  |
| ENSBTAG00000007966 | ENSBTAG00000010449 |  |  |
| ENSBTAG00000007968 | ENSBTAG00000010452 |  |  |
| ENSBTAG00000007969 | ENSBTAG00000010455 |  |  |
| ENSBTAG00000007974 | ENSBTAG00000010456 |  |  |
| ENSBTAG00000007975 | ENSBTAG00000010460 |  |  |
| ENSBTAG00000007976 | ENSBTAG00000010462 |  |  |
| ENSBTAG00000007977 | ENSBTAG00000010470 |  |  |
| ENSBTAG00000007979 | ENSBTAG00000010484 |  |  |
| ENSBTAG00000007993 | ENSBTAG00000010485 |  |  |
| ENSBTAG00000007994 | ENSBTAG00000010487 |  |  |
| ENSBTAG00000008001 | ENSBTAG00000010490 |  |  |
| ENSBTAG00000008003 | ENSBTAG00000010492 |  |  |
| ENSBTAG00000008004 | ENSBTAG00000010496 |  |  |
| ENSBTAG00000008005 | ENSBTAG00000010498 |  |  |
| ENSBTAG00000008006 | ENSBTAG00000010500 |  |  |
| ENSBTAG00000008008 | ENSBTAG00000010501 |  |  |
| ENSBTAG00000008009 | ENSBTAG00000010502 |  |  |
| ENSBTAG00000008010 | ENSBTAG00000010505 |  |  |
| ENSBTAG00000008013 | ENSBTAG00000010506 |  |  |
| ENSBTAG00000008014 | ENSBTAG00000010508 |  |  |
| ENSBTAG00000008016 | ENSBTAG00000010509 |  |  |
| ENSBTAG00000008021 | ENSBTAG00000010515 |  |  |
| ENSBTAG00000008022 | ENSBTAG00000010519 |  |  |
| ENSBTAG00000008023 | ENSBTAG00000010529 |  |  |
| ENSBTAG00000008025 | ENSBTAG00000010530 |  |  |
| ENSBTAG00000008026 | ENSBTAG00000010532 |  |  |
| ENSBTAG00000008028 | ENSBTAG00000010533 |  |  |
| ENSBTAG00000008029 | ENSBTAG00000010534 |  |  |
| ENSBTAG00000008032 | ENSBTAG00000010535 |  |  |
| ENSBTAG00000008033 | ENSBTAG00000010541 |  |  |
| ENSBTAG00000008034 | ENSBTAG00000010543 |  |  |
| ENSBTAG00000008040 | ENSBTAG00000010545 |  |  |
| ENSBTAG00000008048 | ENSBTAG00000010548 |  |  |
| ENSBTAG00000008053 | ENSBTAG00000010551 |  |  |
| ENSBTAG00000008054 | ENSBTAG00000010552 |  |  |
| ENSBTAG00000008060 | ENSBTAG00000010563 |  |  |
| ENSBTAG00000008061 | ENSBTAG00000010564 |  |  |
| ENSBTAG00000008065 | ENSBTAG00000010576 |  |  |
| ENSBTAG00000008072 | ENSBTAG00000010579 |  |  |
| ENSBTAG00000008074 | ENSBTAG00000010582 |  |  |
| ENSBTAG00000008075 | ENSBTAG00000010584 |  |  |
| ENSBTAG00000008076 | ENSBTAG00000010587 |  |  |
| ENSBTAG00000008077 | ENSBTAG00000010590 |  |  |
| ENSBTAG00000008079 | ENSBTAG00000010593 |  |  |
| ENSBTAG00000008084 | ENSBTAG00000010595 |  |  |
| ENSBTAG00000008088 | ENSBTAG00000010597 |  |  |
| ENSBTAG00000008089 | ENSBTAG00000010610 |  |  |
| ENSBTAG00000008090 | ENSBTAG00000010611 |  |  |
| ENSBTAG00000008092 | ENSBTAG00000010612 |  |  |
| ENSBTAG00000008095 | ENSBTAG00000010616 |  |  |
| ENSBTAG00000008096 | ENSBTAG00000010619 |  |  |
| ENSBTAG00000008097 | ENSBTAG00000010620 |  |  |
| ENSBTAG00000008099 | ENSBTAG00000010622 |  |  |
| ENSBTAG00000008100 | ENSBTAG00000010624 |  |  |
| ENSBTAG00000008109 | ENSBTAG00000010626 |  |  |
| ENSBTAG00000008110 | ENSBTAG00000010642 |  |  |
| ENSBTAG00000008112 | ENSBTAG00000010647 |  |  |
| ENSBTAG00000008113 | ENSBTAG00000010649 |  |  |
| ENSBTAG00000008114 | ENSBTAG00000010653 |  |  |
| ENSBTAG00000008115 | ENSBTAG00000010661 |  |  |
| ENSBTAG00000008116 | ENSBTAG00000010662 |  |  |
| ENSBTAG00000008118 | ENSBTAG00000010664 |  |  |
| ENSBTAG00000008120 | ENSBTAG00000010666 |  |  |
| ENSBTAG00000008121 | ENSBTAG00000010667 |  |  |
| ENSBTAG00000008122 | ENSBTAG00000010673 |  |  |
| ENSBTAG00000008126 | ENSBTAG00000010677 |  |  |
| ENSBTAG00000008127 | ENSBTAG00000010681 |  |  |
| ENSBTAG00000008129 | ENSBTAG00000010682 |  |  |
| ENSBTAG00000008130 | ENSBTAG00000010688 |  |  |
| ENSBTAG00000008131 | ENSBTAG00000010692 |  |  |
| ENSBTAG00000008132 | ENSBTAG00000010693 |  |  |
| ENSBTAG00000008133 | ENSBTAG00000010694 |  |  |
| ENSBTAG00000008134 | ENSBTAG00000010698 |  |  |
| ENSBTAG00000008135 | ENSBTAG00000010717 |  |  |
| ENSBTAG00000008139 | ENSBTAG00000010719 |  |  |
| ENSBTAG00000008140 | ENSBTAG00000010721 |  |  |
| ENSBTAG00000008142 | ENSBTAG00000010726 |  |  |
| ENSBTAG00000008143 | ENSBTAG00000010727 |  |  |
| ENSBTAG00000008147 | ENSBTAG00000010731 |  |  |
| ENSBTAG00000008150 | ENSBTAG00000010735 |  |  |
| ENSBTAG00000008151 | ENSBTAG00000010738 |  |  |
| ENSBTAG00000008153 | ENSBTAG00000010739 |  |  |
| ENSBTAG00000008154 | ENSBTAG00000010745 |  |  |
| ENSBTAG00000008159 | ENSBTAG00000010756 |  |  |
| ENSBTAG00000008165 | ENSBTAG00000010758 |  |  |
| ENSBTAG00000008167 | ENSBTAG00000010760 |  |  |
| ENSBTAG00000008169 | ENSBTAG00000010773 |  |  |
| ENSBTAG00000008172 | ENSBTAG00000010774 |  |  |
| ENSBTAG00000008176 | ENSBTAG00000010775 |  |  |
| ENSBTAG00000008180 | ENSBTAG00000010777 |  |  |
| ENSBTAG00000008181 | ENSBTAG00000010778 |  |  |
| ENSBTAG00000008182 | ENSBTAG00000010784 |  |  |
| ENSBTAG00000008185 | ENSBTAG00000010785 |  |  |
| ENSBTAG00000008188 | ENSBTAG00000010786 |  |  |
| ENSBTAG00000008190 | ENSBTAG00000010789 |  |  |
| ENSBTAG00000008191 | ENSBTAG00000010792 |  |  |
| ENSBTAG00000008192 | ENSBTAG00000010795 |  |  |
| ENSBTAG00000008195 | ENSBTAG00000010799 |  |  |
| ENSBTAG00000008196 | ENSBTAG00000010809 |  |  |
| ENSBTAG00000008201 | ENSBTAG00000010810 |  |  |
| ENSBTAG00000008202 | ENSBTAG00000010812 |  |  |
| ENSBTAG00000008203 | ENSBTAG00000010818 |  |  |
| ENSBTAG00000008216 | ENSBTAG00000010819 |  |  |
| ENSBTAG00000008219 | ENSBTAG00000010820 |  |  |
| ENSBTAG00000008224 | ENSBTAG00000010822 |  |  |
| ENSBTAG00000008237 | ENSBTAG00000010832 |  |  |
| ENSBTAG00000008248 | ENSBTAG00000010837 |  |  |
| ENSBTAG00000008250 | ENSBTAG00000010841 |  |  |
| ENSBTAG00000008260 | ENSBTAG00000010843 |  |  |
| ENSBTAG00000008267 | ENSBTAG00000010850 |  |  |
| ENSBTAG00000008269 | ENSBTAG00000010860 |  |  |
| ENSBTAG00000008271 | ENSBTAG00000010863 |  |  |
| ENSBTAG00000008278 | ENSBTAG00000010865 |  |  |
| ENSBTAG00000008279 | ENSBTAG00000010866 |  |  |
| ENSBTAG00000008280 | ENSBTAG00000010867 |  |  |
| ENSBTAG00000008285 | ENSBTAG00000010868 |  |  |
| ENSBTAG00000008287 | ENSBTAG00000010871 |  |  |
| ENSBTAG00000008288 | ENSBTAG00000010875 |  |  |
| ENSBTAG00000008291 | ENSBTAG00000010878 |  |  |
| ENSBTAG00000008292 | ENSBTAG00000010885 |  |  |
| ENSBTAG00000008294 | ENSBTAG00000010889 |  |  |
| ENSBTAG00000008296 | ENSBTAG00000010890 |  |  |
| ENSBTAG00000008299 | ENSBTAG00000010897 |  |  |
| ENSBTAG00000008300 | ENSBTAG00000010898 |  |  |
| ENSBTAG00000008302 | ENSBTAG00000010906 |  |  |
| ENSBTAG00000008303 | ENSBTAG00000010907 |  |  |
| ENSBTAG00000008306 | ENSBTAG00000010910 |  |  |
| ENSBTAG00000008309 | ENSBTAG00000010913 |  |  |
| ENSBTAG00000008310 | ENSBTAG00000010916 |  |  |
| ENSBTAG00000008313 | ENSBTAG00000010919 |  |  |
| ENSBTAG00000008314 | ENSBTAG00000010924 |  |  |
| ENSBTAG00000008320 | ENSBTAG00000010937 |  |  |
| ENSBTAG00000008321 | ENSBTAG00000010943 |  |  |
| ENSBTAG00000008323 | ENSBTAG00000010944 |  |  |
| ENSBTAG00000008327 | ENSBTAG00000010945 |  |  |
| ENSBTAG00000008329 | ENSBTAG00000010947 |  |  |
| ENSBTAG00000008331 | ENSBTAG00000010948 |  |  |
| ENSBTAG00000008332 | ENSBTAG00000010955 |  |  |
| ENSBTAG00000008333 | ENSBTAG00000010956 |  |  |
| ENSBTAG00000008336 | ENSBTAG00000010959 |  |  |
| ENSBTAG00000008340 | ENSBTAG00000010963 |  |  |
| ENSBTAG00000008341 | ENSBTAG00000010968 |  |  |
| ENSBTAG00000008342 | ENSBTAG00000010971 |  |  |
| ENSBTAG00000008343 | ENSBTAG00000010977 |  |  |
| ENSBTAG00000008347 | ENSBTAG00000010980 |  |  |
| ENSBTAG00000008348 | ENSBTAG00000010987 |  |  |
| ENSBTAG00000008350 | ENSBTAG00000010990 |  |  |
| ENSBTAG00000008353 | ENSBTAG00000010991 |  |  |
| ENSBTAG00000008361 | ENSBTAG00000010993 |  |  |
| ENSBTAG00000008362 | ENSBTAG00000011000 |  |  |
| ENSBTAG00000008367 | ENSBTAG00000011001 |  |  |
| ENSBTAG00000008371 | ENSBTAG00000011007 |  |  |
| ENSBTAG00000008372 | ENSBTAG00000011010 |  |  |
| ENSBTAG00000008374 | ENSBTAG00000011017 |  |  |
| ENSBTAG00000008378 | ENSBTAG00000011021 |  |  |
| ENSBTAG00000008380 | ENSBTAG00000011022 |  |  |
| ENSBTAG00000008388 | ENSBTAG00000011023 |  |  |
| ENSBTAG00000008394 | ENSBTAG00000011024 |  |  |
| ENSBTAG00000008395 | ENSBTAG00000011027 |  |  |
| ENSBTAG00000008396 | ENSBTAG00000011034 |  |  |
| ENSBTAG00000008401 | ENSBTAG00000011037 |  |  |
| ENSBTAG00000008403 | ENSBTAG00000011038 |  |  |
| ENSBTAG00000008409 | ENSBTAG00000011042 |  |  |
| ENSBTAG00000008410 | ENSBTAG00000011043 |  |  |
| ENSBTAG00000008411 | ENSBTAG00000011050 |  |  |
| ENSBTAG00000008414 | ENSBTAG00000011068 |  |  |
| ENSBTAG00000008416 | ENSBTAG00000011070 |  |  |
| ENSBTAG00000008417 | ENSBTAG00000011071 |  |  |
| ENSBTAG00000008419 | ENSBTAG00000011072 |  |  |
| ENSBTAG00000008423 | ENSBTAG00000011075 |  |  |
| ENSBTAG00000008424 | ENSBTAG00000011076 |  |  |
| ENSBTAG00000008433 | ENSBTAG00000011082 |  |  |
| ENSBTAG00000008435 | ENSBTAG00000011087 |  |  |
| ENSBTAG00000008436 | ENSBTAG00000011095 |  |  |
| ENSBTAG00000008437 | ENSBTAG00000011096 |  |  |
| ENSBTAG00000008438 | ENSBTAG00000011102 |  |  |
| ENSBTAG00000008439 | ENSBTAG00000011103 |  |  |
| ENSBTAG00000008442 | ENSBTAG00000011104 |  |  |
| ENSBTAG00000008448 | ENSBTAG00000011105 |  |  |
| ENSBTAG00000008452 | ENSBTAG00000011108 |  |  |
| ENSBTAG00000008457 | ENSBTAG00000011124 |  |  |
| ENSBTAG00000008461 | ENSBTAG00000011125 |  |  |
| ENSBTAG00000008465 | ENSBTAG00000011126 |  |  |
| ENSBTAG00000008466 | ENSBTAG00000011131 |  |  |
| ENSBTAG00000008479 | ENSBTAG00000011133 |  |  |
| ENSBTAG00000008482 | ENSBTAG00000011143 |  |  |
| ENSBTAG00000008483 | ENSBTAG00000011145 |  |  |
| ENSBTAG00000008484 | ENSBTAG00000011146 |  |  |
| ENSBTAG00000008485 | ENSBTAG00000011147 |  |  |
| ENSBTAG00000008490 | ENSBTAG00000011150 |  |  |
| ENSBTAG00000008492 | ENSBTAG00000011154 |  |  |
| ENSBTAG00000008493 | ENSBTAG00000011163 |  |  |
| ENSBTAG00000008497 | ENSBTAG00000011176 |  |  |
| ENSBTAG00000008504 | ENSBTAG00000011179 |  |  |
| ENSBTAG00000008509 | ENSBTAG00000011184 |  |  |
| ENSBTAG00000008523 | ENSBTAG00000011186 |  |  |
| ENSBTAG00000008525 | ENSBTAG00000011187 |  |  |
| ENSBTAG00000008527 | ENSBTAG00000011189 |  |  |
| ENSBTAG00000008528 | ENSBTAG00000011190 |  |  |
| ENSBTAG00000008538 | ENSBTAG00000011193 |  |  |
| ENSBTAG00000008539 | ENSBTAG00000011197 |  |  |
| ENSBTAG00000008541 | ENSBTAG00000011198 |  |  |
| ENSBTAG00000008542 | ENSBTAG00000011200 |  |  |
| ENSBTAG00000008543 | ENSBTAG00000011204 |  |  |
| ENSBTAG00000008545 | ENSBTAG00000011206 |  |  |
| ENSBTAG00000008548 | ENSBTAG00000011207 |  |  |
| ENSBTAG00000008550 | ENSBTAG00000011217 |  |  |
| ENSBTAG00000008553 | ENSBTAG00000011224 |  |  |
| ENSBTAG00000008554 | ENSBTAG00000011225 |  |  |
| ENSBTAG00000008555 | ENSBTAG00000011227 |  |  |
| ENSBTAG00000008562 | ENSBTAG00000011238 |  |  |
| ENSBTAG00000008571 | ENSBTAG00000011239 |  |  |
| ENSBTAG00000008573 | ENSBTAG00000011241 |  |  |
| ENSBTAG00000008577 | ENSBTAG00000011243 |  |  |
| ENSBTAG00000008578 | ENSBTAG00000011245 |  |  |
| ENSBTAG00000008579 | ENSBTAG00000011246 |  |  |
| ENSBTAG00000008587 | ENSBTAG00000011247 |  |  |
| ENSBTAG00000008591 | ENSBTAG00000011250 |  |  |
| ENSBTAG00000008592 | ENSBTAG00000011256 |  |  |
| ENSBTAG00000008595 | ENSBTAG00000011257 |  |  |
| ENSBTAG00000008596 | ENSBTAG00000011258 |  |  |
| ENSBTAG00000008597 | ENSBTAG00000011263 |  |  |
| ENSBTAG00000008600 | ENSBTAG00000011268 |  |  |
| ENSBTAG00000008603 | ENSBTAG00000011274 |  |  |
| ENSBTAG00000008606 | ENSBTAG00000011275 |  |  |
| ENSBTAG00000008611 | ENSBTAG00000011278 |  |  |
| ENSBTAG00000008612 | ENSBTAG00000011284 |  |  |
| ENSBTAG00000008621 | ENSBTAG00000011285 |  |  |
| ENSBTAG00000008624 | ENSBTAG00000011291 |  |  |
| ENSBTAG00000008625 | ENSBTAG00000011304 |  |  |
| ENSBTAG00000008629 | ENSBTAG00000011307 |  |  |
| ENSBTAG00000008633 | ENSBTAG00000011311 |  |  |
| ENSBTAG00000008636 | ENSBTAG00000011313 |  |  |
| ENSBTAG00000008638 | ENSBTAG00000011316 |  |  |
| ENSBTAG00000008642 | ENSBTAG00000011319 |  |  |
| ENSBTAG00000008645 | ENSBTAG00000011322 |  |  |
| ENSBTAG00000008646 | ENSBTAG00000011328 |  |  |
| ENSBTAG00000008648 | ENSBTAG00000011339 |  |  |
| ENSBTAG00000008650 | ENSBTAG00000011340 |  |  |
| ENSBTAG00000008652 | ENSBTAG00000011344 |  |  |
| ENSBTAG00000008654 | ENSBTAG00000011350 |  |  |
| ENSBTAG00000008664 | ENSBTAG00000011354 |  |  |
| ENSBTAG00000008665 | ENSBTAG00000011358 |  |  |
| ENSBTAG00000008672 | ENSBTAG00000011366 |  |  |
| ENSBTAG00000008674 | ENSBTAG00000011367 |  |  |
| ENSBTAG00000008683 | ENSBTAG00000011374 |  |  |
| ENSBTAG00000008686 | ENSBTAG00000011375 |  |  |
| ENSBTAG00000008687 | ENSBTAG00000011382 |  |  |
| ENSBTAG00000008688 | ENSBTAG00000011383 |  |  |
| ENSBTAG00000008690 | ENSBTAG00000011387 |  |  |
| ENSBTAG00000008692 | ENSBTAG00000011389 |  |  |
| ENSBTAG00000008693 | ENSBTAG00000011390 |  |  |
| ENSBTAG00000008695 | ENSBTAG00000011392 |  |  |
| ENSBTAG00000008699 | ENSBTAG00000011395 |  |  |
| ENSBTAG00000008700 | ENSBTAG00000011396 |  |  |
| ENSBTAG00000008703 | ENSBTAG00000011397 |  |  |
| ENSBTAG00000008705 | ENSBTAG00000011399 |  |  |
| ENSBTAG00000008709 | ENSBTAG00000011401 |  |  |
| ENSBTAG00000008711 | ENSBTAG00000011403 |  |  |
| ENSBTAG00000008716 | ENSBTAG00000011411 |  |  |
| ENSBTAG00000008717 | ENSBTAG00000011414 |  |  |
| ENSBTAG00000008718 | ENSBTAG00000011417 |  |  |
| ENSBTAG00000008720 | ENSBTAG00000011419 |  |  |
| ENSBTAG00000008721 | ENSBTAG00000011424 |  |  |
| ENSBTAG00000008726 | ENSBTAG00000011425 |  |  |
| ENSBTAG00000008728 | ENSBTAG00000011427 |  |  |
| ENSBTAG00000008729 | ENSBTAG00000011429 |  |  |
| ENSBTAG00000008730 | ENSBTAG00000011433 |  |  |
| ENSBTAG00000008731 | ENSBTAG00000011435 |  |  |
| ENSBTAG00000008733 | ENSBTAG00000011445 |  |  |
| ENSBTAG00000008734 | ENSBTAG00000011455 |  |  |
| ENSBTAG00000008735 | ENSBTAG00000011458 |  |  |
| ENSBTAG00000008736 | ENSBTAG00000011461 |  |  |
| ENSBTAG00000008737 | ENSBTAG00000011463 |  |  |
| ENSBTAG00000008743 | ENSBTAG00000011467 |  |  |
| ENSBTAG00000008744 | ENSBTAG00000011473 |  |  |
| ENSBTAG00000008747 | ENSBTAG00000011476 |  |  |
| ENSBTAG00000008755 | ENSBTAG00000011479 |  |  |
| ENSBTAG00000008756 | ENSBTAG00000011480 |  |  |
| ENSBTAG00000008759 | ENSBTAG00000011481 |  |  |
| ENSBTAG00000008762 | ENSBTAG00000011482 |  |  |
| ENSBTAG00000008763 | ENSBTAG00000011484 |  |  |
| ENSBTAG00000008764 | ENSBTAG00000011487 |  |  |
| ENSBTAG00000008765 | ENSBTAG00000011488 |  |  |
| ENSBTAG00000008766 | ENSBTAG00000011490 |  |  |
| ENSBTAG00000008773 | ENSBTAG00000011491 |  |  |
| ENSBTAG00000008777 | ENSBTAG00000011494 |  |  |
| ENSBTAG00000008778 | ENSBTAG00000011495 |  |  |
| ENSBTAG00000008789 | ENSBTAG00000011498 |  |  |
| ENSBTAG00000008793 | ENSBTAG00000011500 |  |  |
| ENSBTAG00000008794 | ENSBTAG00000011505 |  |  |
| ENSBTAG00000008800 | ENSBTAG00000011514 |  |  |
| ENSBTAG00000008801 | ENSBTAG00000011516 |  |  |
| ENSBTAG00000008802 | ENSBTAG00000011517 |  |  |
| ENSBTAG00000008805 | ENSBTAG00000011525 |  |  |
| ENSBTAG00000008807 | ENSBTAG00000011528 |  |  |
| ENSBTAG00000008808 | ENSBTAG00000011530 |  |  |
| ENSBTAG00000008812 | ENSBTAG00000011531 |  |  |
| ENSBTAG00000008815 | ENSBTAG00000011540 |  |  |
| ENSBTAG00000008816 | ENSBTAG00000011541 |  |  |
| ENSBTAG00000008819 | ENSBTAG00000011543 |  |  |
| ENSBTAG00000008821 | ENSBTAG00000011544 |  |  |
| ENSBTAG00000008825 | ENSBTAG00000011545 |  |  |
| ENSBTAG00000008826 | ENSBTAG00000011551 |  |  |
| ENSBTAG00000008827 | ENSBTAG00000011553 |  |  |
| ENSBTAG00000008828 | ENSBTAG00000011556 |  |  |
| ENSBTAG00000008833 | ENSBTAG00000011563 |  |  |
| ENSBTAG00000008835 | ENSBTAG00000011571 |  |  |
| ENSBTAG00000008837 | ENSBTAG00000011578 |  |  |
| ENSBTAG00000008840 | ENSBTAG00000011583 |  |  |
| ENSBTAG00000008841 | ENSBTAG00000011584 |  |  |
| ENSBTAG00000008842 | ENSBTAG00000011586 |  |  |
| ENSBTAG00000008845 | ENSBTAG00000011591 |  |  |
| ENSBTAG00000008849 | ENSBTAG00000011593 |  |  |
| ENSBTAG00000008852 | ENSBTAG00000011600 |  |  |
| ENSBTAG00000008853 | ENSBTAG00000011602 |  |  |
| ENSBTAG00000008854 | ENSBTAG00000011608 |  |  |
| ENSBTAG00000008860 | ENSBTAG00000011611 |  |  |
| ENSBTAG00000008862 | ENSBTAG00000011613 |  |  |
| ENSBTAG00000008863 | ENSBTAG00000011617 |  |  |
| ENSBTAG00000008868 | ENSBTAG00000011623 |  |  |
| ENSBTAG00000008873 | ENSBTAG00000011628 |  |  |
| ENSBTAG00000008880 | ENSBTAG00000011632 |  |  |
| ENSBTAG00000008884 | ENSBTAG00000011634 |  |  |
| ENSBTAG00000008887 | ENSBTAG00000011639 |  |  |
| ENSBTAG00000008893 | ENSBTAG00000011642 |  |  |
| ENSBTAG00000008895 | ENSBTAG00000011644 |  |  |
| ENSBTAG00000008896 | ENSBTAG00000011649 |  |  |
| ENSBTAG00000008900 | ENSBTAG00000011658 |  |  |
| ENSBTAG00000008902 | ENSBTAG00000011660 |  |  |
| ENSBTAG00000008908 | ENSBTAG00000011689 |  |  |
| ENSBTAG00000008913 | ENSBTAG00000011692 |  |  |
| ENSBTAG00000008915 | ENSBTAG00000011698 |  |  |
| ENSBTAG00000008916 | ENSBTAG00000011700 |  |  |
| ENSBTAG00000008920 | ENSBTAG00000011709 |  |  |
| ENSBTAG00000008925 | ENSBTAG00000011713 |  |  |
| ENSBTAG00000008926 | ENSBTAG00000011726 |  |  |
| ENSBTAG00000008931 | ENSBTAG00000011727 |  |  |
| ENSBTAG00000008933 | ENSBTAG00000011729 |  |  |
| ENSBTAG00000008934 | ENSBTAG00000011732 |  |  |
| ENSBTAG00000008935 | ENSBTAG00000011736 |  |  |
| ENSBTAG00000008937 | ENSBTAG00000011740 |  |  |
| ENSBTAG00000008939 | ENSBTAG00000011741 |  |  |
| ENSBTAG00000008940 | ENSBTAG00000011752 |  |  |
| ENSBTAG00000008943 | ENSBTAG00000011757 |  |  |
| ENSBTAG00000008945 | ENSBTAG00000011765 |  |  |
| ENSBTAG00000008946 | ENSBTAG00000011766 |  |  |
| ENSBTAG00000008947 | ENSBTAG00000011767 |  |  |
| ENSBTAG00000008951 | ENSBTAG00000011770 |  |  |
| ENSBTAG00000008952 | ENSBTAG00000011779 |  |  |
| ENSBTAG00000008953 | ENSBTAG00000011785 |  |  |
| ENSBTAG00000008956 | ENSBTAG00000011787 |  |  |
| ENSBTAG00000008958 | ENSBTAG00000011788 |  |  |
| ENSBTAG00000008959 | ENSBTAG00000011789 |  |  |
| ENSBTAG00000008964 | ENSBTAG00000011793 |  |  |
| ENSBTAG00000008966 | ENSBTAG00000011796 |  |  |
| ENSBTAG00000008967 | ENSBTAG00000011798 |  |  |
| ENSBTAG00000008973 | ENSBTAG00000011800 |  |  |
| ENSBTAG00000008978 | ENSBTAG00000011802 |  |  |
| ENSBTAG00000008987 | ENSBTAG00000011805 |  |  |
| ENSBTAG00000008989 | ENSBTAG00000011808 |  |  |
| ENSBTAG00000008993 | ENSBTAG00000011810 |  |  |
| ENSBTAG00000008998 | ENSBTAG00000011812 |  |  |
| ENSBTAG00000009002 | ENSBTAG00000011815 |  |  |
| ENSBTAG00000009012 | ENSBTAG00000011818 |  |  |
| ENSBTAG00000009014 | ENSBTAG00000011823 |  |  |
| ENSBTAG00000009017 | ENSBTAG00000011824 |  |  |
| ENSBTAG00000009021 | ENSBTAG00000011825 |  |  |
| ENSBTAG00000009022 | ENSBTAG00000011826 |  |  |
| ENSBTAG00000009024 | ENSBTAG00000011831 |  |  |
| ENSBTAG00000009026 | ENSBTAG00000011832 |  |  |
| ENSBTAG00000009034 | ENSBTAG00000011847 |  |  |
| ENSBTAG00000009035 | ENSBTAG00000011851 |  |  |
| ENSBTAG00000009036 | ENSBTAG00000011854 |  |  |
| ENSBTAG00000009046 | ENSBTAG00000011860 |  |  |
| ENSBTAG00000009047 | ENSBTAG00000011861 |  |  |
| ENSBTAG00000009048 | ENSBTAG00000011865 |  |  |
| ENSBTAG00000009049 | ENSBTAG00000011869 |  |  |
| ENSBTAG00000009050 | ENSBTAG00000011876 |  |  |
| ENSBTAG00000009051 | ENSBTAG00000011879 |  |  |
| ENSBTAG00000009055 | ENSBTAG00000011880 |  |  |
| ENSBTAG00000009059 | ENSBTAG00000011885 |  |  |
| ENSBTAG00000009061 | ENSBTAG00000011893 |  |  |
| ENSBTAG00000009065 | ENSBTAG00000011894 |  |  |
| ENSBTAG00000009067 | ENSBTAG00000011895 |  |  |
| ENSBTAG00000009075 | ENSBTAG00000011898 |  |  |
| ENSBTAG00000009076 | ENSBTAG00000011902 |  |  |
| ENSBTAG00000009077 | ENSBTAG00000011905 |  |  |
| ENSBTAG00000009079 | ENSBTAG00000011909 |  |  |
| ENSBTAG00000009085 | ENSBTAG00000011910 |  |  |
| ENSBTAG00000009087 | ENSBTAG00000011911 |  |  |
| ENSBTAG00000009091 | ENSBTAG00000011912 |  |  |
| ENSBTAG00000009097 | ENSBTAG00000011916 |  |  |
| ENSBTAG00000009098 | ENSBTAG00000011917 |  |  |
| ENSBTAG00000009101 | ENSBTAG00000011932 |  |  |
| ENSBTAG00000009103 | ENSBTAG00000011937 |  |  |
| ENSBTAG00000009104 | ENSBTAG00000011944 |  |  |
| ENSBTAG00000009106 | ENSBTAG00000011951 |  |  |
| ENSBTAG00000009112 | ENSBTAG00000011952 |  |  |
| ENSBTAG00000009115 | ENSBTAG00000011953 |  |  |
| ENSBTAG00000009121 | ENSBTAG00000011957 |  |  |
| ENSBTAG00000009126 | ENSBTAG00000011960 |  |  |
| ENSBTAG00000009127 | ENSBTAG00000011964 |  |  |
| ENSBTAG00000009129 | ENSBTAG00000011966 |  |  |
| ENSBTAG00000009132 | ENSBTAG00000011986 |  |  |
| ENSBTAG00000009137 | ENSBTAG00000011990 |  |  |
| ENSBTAG00000009141 | ENSBTAG00000011994 |  |  |
| ENSBTAG00000009144 | ENSBTAG00000012004 |  |  |
| ENSBTAG00000009145 | ENSBTAG00000012007 |  |  |
| ENSBTAG00000009148 | ENSBTAG00000012014 |  |  |
| ENSBTAG00000009150 | ENSBTAG00000012020 |  |  |
| ENSBTAG00000009152 | ENSBTAG00000012024 |  |  |
| ENSBTAG00000009153 | ENSBTAG00000012034 |  |  |
| ENSBTAG00000009156 | ENSBTAG00000012039 |  |  |
| ENSBTAG00000009163 | ENSBTAG00000012046 |  |  |
| ENSBTAG00000009165 | ENSBTAG00000012049 |  |  |
| ENSBTAG00000009167 | ENSBTAG00000012057 |  |  |
| ENSBTAG00000009172 | ENSBTAG00000012059 |  |  |
| ENSBTAG00000009174 | ENSBTAG00000012063 |  |  |
| ENSBTAG00000009175 | ENSBTAG00000012066 |  |  |
| ENSBTAG00000009176 | ENSBTAG00000012069 |  |  |
| ENSBTAG00000009178 | ENSBTAG00000012073 |  |  |
| ENSBTAG00000009180 | ENSBTAG00000012074 |  |  |
| ENSBTAG00000009182 | ENSBTAG00000012077 |  |  |
| ENSBTAG00000009183 | ENSBTAG00000012081 |  |  |
| ENSBTAG00000009188 | ENSBTAG00000012086 |  |  |
| ENSBTAG00000009191 | ENSBTAG00000012094 |  |  |
| ENSBTAG00000009194 | ENSBTAG00000012100 |  |  |
| ENSBTAG00000009198 | ENSBTAG00000012103 |  |  |
| ENSBTAG00000009201 | ENSBTAG00000012104 |  |  |
| ENSBTAG00000009207 | ENSBTAG00000012106 |  |  |
| ENSBTAG00000009208 | ENSBTAG00000012112 |  |  |
| ENSBTAG00000009209 | ENSBTAG00000012117 |  |  |
| ENSBTAG00000009210 | ENSBTAG00000012119 |  |  |
| ENSBTAG00000009213 | ENSBTAG00000012121 |  |  |
| ENSBTAG00000009214 | ENSBTAG00000012125 |  |  |
| ENSBTAG00000009215 | ENSBTAG00000012139 |  |  |
| ENSBTAG00000009216 | ENSBTAG00000012140 |  |  |
| ENSBTAG00000009218 | ENSBTAG00000012141 |  |  |
| ENSBTAG00000009223 | ENSBTAG00000012142 |  |  |
| ENSBTAG00000009228 | ENSBTAG00000012152 |  |  |
| ENSBTAG00000009231 | ENSBTAG00000012156 |  |  |
| ENSBTAG00000009233 | ENSBTAG00000012163 |  |  |
| ENSBTAG00000009236 | ENSBTAG00000012170 |  |  |
| ENSBTAG00000009237 | ENSBTAG00000012176 |  |  |
| ENSBTAG00000009245 | ENSBTAG00000012197 |  |  |
| ENSBTAG00000009251 | ENSBTAG00000012199 |  |  |
| ENSBTAG00000009252 | ENSBTAG00000012211 |  |  |
| ENSBTAG00000009256 | ENSBTAG00000012213 |  |  |
| ENSBTAG00000009260 | ENSBTAG00000012217 |  |  |
| ENSBTAG00000009263 | ENSBTAG00000012219 |  |  |
| ENSBTAG00000009265 | ENSBTAG00000012223 |  |  |
| ENSBTAG00000009271 | ENSBTAG00000012225 |  |  |
| ENSBTAG00000009272 | ENSBTAG00000012228 |  |  |
| ENSBTAG00000009275 | ENSBTAG00000012232 |  |  |
| ENSBTAG00000009278 | ENSBTAG00000012235 |  |  |
| ENSBTAG00000009279 | ENSBTAG00000012237 |  |  |
| ENSBTAG00000009281 | ENSBTAG00000012239 |  |  |
| ENSBTAG00000009284 | ENSBTAG00000012242 |  |  |
| ENSBTAG00000009289 | ENSBTAG00000012247 |  |  |
| ENSBTAG00000009290 | ENSBTAG00000012249 |  |  |
| ENSBTAG00000009291 | ENSBTAG00000012250 |  |  |
| ENSBTAG00000009292 | ENSBTAG00000012259 |  |  |
| ENSBTAG00000009293 | ENSBTAG00000012261 |  |  |
| ENSBTAG00000009294 | ENSBTAG00000012262 |  |  |
| ENSBTAG00000009297 | ENSBTAG00000012267 |  |  |
| ENSBTAG00000009299 | ENSBTAG00000012272 |  |  |
| ENSBTAG00000009304 | ENSBTAG00000012273 |  |  |
| ENSBTAG00000009305 | ENSBTAG00000012275 |  |  |
| ENSBTAG00000009307 | ENSBTAG00000012284 |  |  |
| ENSBTAG00000009309 | ENSBTAG00000012291 |  |  |
| ENSBTAG00000009314 | ENSBTAG00000012293 |  |  |
| ENSBTAG00000009315 | ENSBTAG00000012295 |  |  |
| ENSBTAG00000009328 | ENSBTAG00000012305 |  |  |
| ENSBTAG00000009330 | ENSBTAG00000012307 |  |  |
| ENSBTAG00000009331 | ENSBTAG00000012314 |  |  |
| ENSBTAG00000009336 | ENSBTAG00000012319 |  |  |
| ENSBTAG00000009337 | ENSBTAG00000012332 |  |  |
| ENSBTAG00000009338 | ENSBTAG00000012334 |  |  |
| ENSBTAG00000009341 | ENSBTAG00000012342 |  |  |
| ENSBTAG00000009345 | ENSBTAG00000012343 |  |  |
| ENSBTAG00000009348 | ENSBTAG00000012348 |  |  |
| ENSBTAG00000009351 | ENSBTAG00000012349 |  |  |
| ENSBTAG00000009353 | ENSBTAG00000012352 |  |  |
| ENSBTAG00000009354 | ENSBTAG00000012353 |  |  |
| ENSBTAG00000009355 | ENSBTAG00000012355 |  |  |
| ENSBTAG00000009357 | ENSBTAG00000012365 |  |  |
| ENSBTAG00000009362 | ENSBTAG00000012366 |  |  |
| ENSBTAG00000009363 | ENSBTAG00000012374 |  |  |
| ENSBTAG00000009364 | ENSBTAG00000012375 |  |  |
| ENSBTAG00000009365 | ENSBTAG00000012380 |  |  |
| ENSBTAG00000009366 | ENSBTAG00000012382 |  |  |
| ENSBTAG00000009368 | ENSBTAG00000012383 |  |  |
| ENSBTAG00000009372 | ENSBTAG00000012387 |  |  |
| ENSBTAG00000009376 | ENSBTAG00000012390 |  |  |
| ENSBTAG00000009378 | ENSBTAG00000012397 |  |  |
| ENSBTAG00000009381 | ENSBTAG00000012403 |  |  |
| ENSBTAG00000009383 | ENSBTAG00000012405 |  |  |
| ENSBTAG00000009384 | ENSBTAG00000012406 |  |  |
| ENSBTAG00000009387 | ENSBTAG00000012409 |  |  |
| ENSBTAG00000009389 | ENSBTAG00000012412 |  |  |
| ENSBTAG00000009390 | ENSBTAG00000012416 |  |  |
| ENSBTAG00000009392 | ENSBTAG00000012417 |  |  |
| ENSBTAG00000009394 | ENSBTAG00000012432 |  |  |
| ENSBTAG00000009400 | ENSBTAG00000012434 |  |  |
| ENSBTAG00000009408 | ENSBTAG00000012442 |  |  |
| ENSBTAG00000009415 | ENSBTAG00000012443 |  |  |
| ENSBTAG00000009417 | ENSBTAG00000012446 |  |  |
| ENSBTAG00000009419 | ENSBTAG00000012447 |  |  |
| ENSBTAG00000009421 | ENSBTAG00000012448 |  |  |
| ENSBTAG00000009426 | ENSBTAG00000012450 |  |  |
| ENSBTAG00000009428 | ENSBTAG00000012451 |  |  |
| ENSBTAG00000009431 | ENSBTAG00000012454 |  |  |
| ENSBTAG00000009432 | ENSBTAG00000012456 |  |  |
| ENSBTAG00000009435 | ENSBTAG00000012458 |  |  |
| ENSBTAG00000009436 | ENSBTAG00000012460 |  |  |
| ENSBTAG00000009439 | ENSBTAG00000012463 |  |  |
| ENSBTAG00000009443 | ENSBTAG00000012467 |  |  |
| ENSBTAG00000009446 | ENSBTAG00000012470 |  |  |
| ENSBTAG00000009451 | ENSBTAG00000012471 |  |  |
| ENSBTAG00000009453 | ENSBTAG00000012476 |  |  |
| ENSBTAG00000009455 | ENSBTAG00000012481 |  |  |
| ENSBTAG00000009458 | ENSBTAG00000012489 |  |  |
| ENSBTAG00000009462 | ENSBTAG00000012491 |  |  |
| ENSBTAG00000009470 | ENSBTAG00000012496 |  |  |
| ENSBTAG00000009471 | ENSBTAG00000012498 |  |  |
| ENSBTAG00000009474 | ENSBTAG00000012499 |  |  |
| ENSBTAG00000009475 | ENSBTAG00000012500 |  |  |
| ENSBTAG00000009476 | ENSBTAG00000012501 |  |  |
| ENSBTAG00000009477 | ENSBTAG00000012507 |  |  |
| ENSBTAG00000009478 | ENSBTAG00000012509 |  |  |
| ENSBTAG00000009481 | ENSBTAG00000012510 |  |  |
| ENSBTAG00000009486 | ENSBTAG00000012511 |  |  |
| ENSBTAG00000009487 | ENSBTAG00000012512 |  |  |
| ENSBTAG00000009490 | ENSBTAG00000012518 |  |  |
| ENSBTAG00000009495 | ENSBTAG00000012519 |  |  |
| ENSBTAG00000009501 | ENSBTAG00000012526 |  |  |
| ENSBTAG00000009504 | ENSBTAG00000012535 |  |  |
| ENSBTAG00000009506 | ENSBTAG00000012541 |  |  |
| ENSBTAG00000009507 | ENSBTAG00000012544 |  |  |
| ENSBTAG00000009513 | ENSBTAG00000012545 |  |  |
| ENSBTAG00000009514 | ENSBTAG00000012552 |  |  |
| ENSBTAG00000009516 | ENSBTAG00000012561 |  |  |
| ENSBTAG00000009517 | ENSBTAG00000012582 |  |  |
| ENSBTAG00000009518 | ENSBTAG00000012584 |  |  |
| ENSBTAG00000009522 | ENSBTAG00000012585 |  |  |
| ENSBTAG00000009523 | ENSBTAG00000012586 |  |  |
| ENSBTAG00000009527 | ENSBTAG00000012587 |  |  |
| ENSBTAG00000009535 | ENSBTAG00000012594 |  |  |
| ENSBTAG00000009541 | ENSBTAG00000012595 |  |  |
| ENSBTAG00000009542 | ENSBTAG00000012596 |  |  |
| ENSBTAG00000009543 | ENSBTAG00000012604 |  |  |
| ENSBTAG00000009545 | ENSBTAG00000012606 |  |  |
| ENSBTAG00000009549 | ENSBTAG00000012608 |  |  |
| ENSBTAG00000009552 | ENSBTAG00000012619 |  |  |
| ENSBTAG00000009554 | ENSBTAG00000012623 |  |  |
| ENSBTAG00000009560 | ENSBTAG00000012626 |  |  |
| ENSBTAG00000009565 | ENSBTAG00000012629 |  |  |
| ENSBTAG00000009569 | ENSBTAG00000012632 |  |  |
| ENSBTAG00000009570 | ENSBTAG00000012637 |  |  |
| ENSBTAG00000009573 | ENSBTAG00000012642 |  |  |
| ENSBTAG00000009574 | ENSBTAG00000012652 |  |  |
| ENSBTAG00000009578 | ENSBTAG00000012656 |  |  |
| ENSBTAG00000009579 | ENSBTAG00000012659 |  |  |
| ENSBTAG00000009580 | ENSBTAG00000012671 |  |  |
| ENSBTAG00000009584 | ENSBTAG00000012672 |  |  |
| ENSBTAG00000009585 | ENSBTAG00000012673 |  |  |
| ENSBTAG00000009586 | ENSBTAG00000012674 |  |  |
| ENSBTAG00000009596 | ENSBTAG00000012675 |  |  |
| ENSBTAG00000009600 | ENSBTAG00000012677 |  |  |
| ENSBTAG00000009602 | ENSBTAG00000012678 |  |  |
| ENSBTAG00000009603 | ENSBTAG00000012688 |  |  |
| ENSBTAG00000009607 | ENSBTAG00000012697 |  |  |
| ENSBTAG00000009611 | ENSBTAG00000012698 |  |  |
| ENSBTAG00000009615 | ENSBTAG00000012700 |  |  |
| ENSBTAG00000009617 | ENSBTAG00000012703 |  |  |
| ENSBTAG00000009624 | ENSBTAG00000012705 |  |  |
| ENSBTAG00000009635 | ENSBTAG00000012719 |  |  |
| ENSBTAG00000009636 | ENSBTAG00000012723 |  |  |
| ENSBTAG00000009639 | ENSBTAG00000012737 |  |  |
| ENSBTAG00000009641 | ENSBTAG00000012739 |  |  |
| ENSBTAG00000009646 | ENSBTAG00000012749 |  |  |
| ENSBTAG00000009647 | ENSBTAG00000012755 |  |  |
| ENSBTAG00000009649 | ENSBTAG00000012757 |  |  |
| ENSBTAG00000009654 | ENSBTAG00000012758 |  |  |
| ENSBTAG00000009655 | ENSBTAG00000012761 |  |  |
| ENSBTAG00000009657 | ENSBTAG00000012762 |  |  |
| ENSBTAG00000009658 | ENSBTAG00000012768 |  |  |
| ENSBTAG00000009661 | ENSBTAG00000012771 |  |  |
| ENSBTAG00000009663 | ENSBTAG00000012774 |  |  |
| ENSBTAG00000009668 | ENSBTAG00000012777 |  |  |
| ENSBTAG00000009676 | ENSBTAG00000012778 |  |  |
| ENSBTAG00000009677 | ENSBTAG00000012780 |  |  |
| ENSBTAG00000009680 | ENSBTAG00000012781 |  |  |
| ENSBTAG00000009685 | ENSBTAG00000012790 |  |  |
| ENSBTAG00000009687 | ENSBTAG00000012792 |  |  |
| ENSBTAG00000009698 | ENSBTAG00000012801 |  |  |
| ENSBTAG00000009702 | ENSBTAG00000012804 |  |  |
| ENSBTAG00000009707 | ENSBTAG00000012805 |  |  |
| ENSBTAG00000009711 | ENSBTAG00000012808 |  |  |
| ENSBTAG00000009719 | ENSBTAG00000012817 |  |  |
| ENSBTAG00000009726 | ENSBTAG00000012818 |  |  |
| ENSBTAG00000009732 | ENSBTAG00000012820 |  |  |
| ENSBTAG00000009733 | ENSBTAG00000012826 |  |  |
| ENSBTAG00000009736 | ENSBTAG00000012827 |  |  |
| ENSBTAG00000009738 | ENSBTAG00000012829 |  |  |
| ENSBTAG00000009739 | ENSBTAG00000012833 |  |  |
| ENSBTAG00000009743 | ENSBTAG00000012838 |  |  |
| ENSBTAG00000009746 | ENSBTAG00000012844 |  |  |
| ENSBTAG00000009747 | ENSBTAG00000012845 |  |  |
| ENSBTAG00000009748 | ENSBTAG00000012848 |  |  |
| ENSBTAG00000009750 | ENSBTAG00000012849 |  |  |
| ENSBTAG00000009755 | ENSBTAG00000012854 |  |  |
| ENSBTAG00000009761 | ENSBTAG00000012855 |  |  |
| ENSBTAG00000009765 | ENSBTAG00000012857 |  |  |
| ENSBTAG00000009768 | ENSBTAG00000012858 |  |  |
| ENSBTAG00000009770 | ENSBTAG00000012860 |  |  |
| ENSBTAG00000009772 | ENSBTAG00000012863 |  |  |
| ENSBTAG00000009774 | ENSBTAG00000012865 |  |  |
| ENSBTAG00000009777 | ENSBTAG00000012885 |  |  |
| ENSBTAG00000009778 | ENSBTAG00000012890 |  |  |
| ENSBTAG00000009780 | ENSBTAG00000012898 |  |  |
| ENSBTAG00000009784 | ENSBTAG00000012900 |  |  |
| ENSBTAG00000009786 | ENSBTAG00000012904 |  |  |
| ENSBTAG00000009796 | ENSBTAG00000012908 |  |  |
| ENSBTAG00000009797 | ENSBTAG00000012909 |  |  |
| ENSBTAG00000009798 | ENSBTAG00000012912 |  |  |
| ENSBTAG00000009800 | ENSBTAG00000012918 |  |  |
| ENSBTAG00000009803 | ENSBTAG00000012919 |  |  |
| ENSBTAG00000009806 | ENSBTAG00000012922 |  |  |
| ENSBTAG00000009813 | ENSBTAG00000012931 |  |  |
| ENSBTAG00000009819 | ENSBTAG00000012937 |  |  |
| ENSBTAG00000009822 | ENSBTAG00000012938 |  |  |
| ENSBTAG00000009828 | ENSBTAG00000012939 |  |  |
| ENSBTAG00000009830 | ENSBTAG00000012940 |  |  |
| ENSBTAG00000009832 | ENSBTAG00000012941 |  |  |
| ENSBTAG00000009834 | ENSBTAG00000012943 |  |  |
| ENSBTAG00000009835 | ENSBTAG00000012957 |  |  |
| ENSBTAG00000009836 | ENSBTAG00000012962 |  |  |
| ENSBTAG00000009837 | ENSBTAG00000012968 |  |  |
| ENSBTAG00000009841 | ENSBTAG00000012969 |  |  |
| ENSBTAG00000009842 | ENSBTAG00000012972 |  |  |
| ENSBTAG00000009844 | ENSBTAG00000012975 |  |  |
| ENSBTAG00000009848 | ENSBTAG00000012979 |  |  |
| ENSBTAG00000009850 | ENSBTAG00000012980 |  |  |
| ENSBTAG00000009851 | ENSBTAG00000012982 |  |  |
| ENSBTAG00000009855 | ENSBTAG00000012985 |  |  |
| ENSBTAG00000009856 | ENSBTAG00000012990 |  |  |
| ENSBTAG00000009859 | ENSBTAG00000012991 |  |  |
| ENSBTAG00000009863 | ENSBTAG00000012994 |  |  |
| ENSBTAG00000009864 | ENSBTAG00000012995 |  |  |
| ENSBTAG00000009867 | ENSBTAG00000012996 |  |  |
| ENSBTAG00000009870 | ENSBTAG00000013004 |  |  |
| ENSBTAG00000009871 | ENSBTAG00000013007 |  |  |
| ENSBTAG00000009873 | ENSBTAG00000013009 |  |  |
| ENSBTAG00000009874 | ENSBTAG00000013010 |  |  |
| ENSBTAG00000009881 | ENSBTAG00000013011 |  |  |
| ENSBTAG00000009886 | ENSBTAG00000013014 |  |  |
| ENSBTAG00000009888 | ENSBTAG00000013016 |  |  |
| ENSBTAG00000009889 | ENSBTAG00000013017 |  |  |
| ENSBTAG00000009899 | ENSBTAG00000013018 |  |  |
| ENSBTAG00000009902 | ENSBTAG00000013030 |  |  |
| ENSBTAG00000009903 | ENSBTAG00000013031 |  |  |
| ENSBTAG00000009904 | ENSBTAG00000013032 |  |  |
| ENSBTAG00000009905 | ENSBTAG00000013033 |  |  |
| ENSBTAG00000009906 | ENSBTAG00000013035 |  |  |
| ENSBTAG00000009911 | ENSBTAG00000013038 |  |  |
| ENSBTAG00000009914 | ENSBTAG00000013042 |  |  |
| ENSBTAG00000009915 | ENSBTAG00000013043 |  |  |
| ENSBTAG00000009918 | ENSBTAG00000013044 |  |  |
| ENSBTAG00000009923 | ENSBTAG00000013050 |  |  |
| ENSBTAG00000009928 | ENSBTAG00000013060 |  |  |
| ENSBTAG00000009939 | ENSBTAG00000013070 |  |  |
| ENSBTAG00000009942 | ENSBTAG00000013072 |  |  |
| ENSBTAG00000009948 | ENSBTAG00000013074 |  |  |
| ENSBTAG00000009956 | ENSBTAG00000013076 |  |  |
| ENSBTAG00000009959 | ENSBTAG00000013078 |  |  |
| ENSBTAG00000009960 | ENSBTAG00000013079 |  |  |
| ENSBTAG00000009962 | ENSBTAG00000013081 |  |  |
| ENSBTAG00000009963 | ENSBTAG00000013096 |  |  |
| ENSBTAG00000009964 | ENSBTAG00000013098 |  |  |
| ENSBTAG00000009965 | ENSBTAG00000013099 |  |  |
| ENSBTAG00000009966 | ENSBTAG00000013105 |  |  |
| ENSBTAG00000009969 | ENSBTAG00000013109 |  |  |
| ENSBTAG00000009975 | ENSBTAG00000013111 |  |  |
| ENSBTAG00000009978 | ENSBTAG00000013112 |  |  |
| ENSBTAG00000009979 | ENSBTAG00000013113 |  |  |
| ENSBTAG00000009982 | ENSBTAG00000013114 |  |  |
| ENSBTAG00000009983 | ENSBTAG00000013118 |  |  |
| ENSBTAG00000009984 | ENSBTAG00000013125 |  |  |
| ENSBTAG00000009985 | ENSBTAG00000013128 |  |  |
| ENSBTAG00000009988 | ENSBTAG00000013131 |  |  |
| ENSBTAG00000009994 | ENSBTAG00000013133 |  |  |
| ENSBTAG00000009995 | ENSBTAG00000013142 |  |  |
| ENSBTAG00000009997 | ENSBTAG00000013143 |  |  |
| ENSBTAG00000009998 | ENSBTAG00000013145 |  |  |
| ENSBTAG00000010001 | ENSBTAG00000013152 |  |  |
| ENSBTAG00000010006 | ENSBTAG00000013159 |  |  |
| ENSBTAG00000010007 | ENSBTAG00000013166 |  |  |
| ENSBTAG00000010009 | ENSBTAG00000013167 |  |  |
| ENSBTAG00000010013 | ENSBTAG00000013168 |  |  |
| ENSBTAG00000010015 | ENSBTAG00000013169 |  |  |
| ENSBTAG00000010016 | ENSBTAG00000013174 |  |  |
| ENSBTAG00000010023 | ENSBTAG00000013184 |  |  |
| ENSBTAG00000010030 | ENSBTAG00000013185 |  |  |
| ENSBTAG00000010036 | ENSBTAG00000013187 |  |  |
| ENSBTAG00000010042 | ENSBTAG00000013191 |  |  |
| ENSBTAG00000010046 | ENSBTAG00000013192 |  |  |
| ENSBTAG00000010048 | ENSBTAG00000013197 |  |  |
| ENSBTAG00000010050 | ENSBTAG00000013201 |  |  |
| ENSBTAG00000010051 | ENSBTAG00000013204 |  |  |
| ENSBTAG00000010059 | ENSBTAG00000013205 |  |  |
| ENSBTAG00000010060 | ENSBTAG00000013211 |  |  |
| ENSBTAG00000010063 | ENSBTAG00000013213 |  |  |
| ENSBTAG00000010068 | ENSBTAG00000013218 |  |  |
| ENSBTAG00000010069 | ENSBTAG00000013224 |  |  |
| ENSBTAG00000010070 | ENSBTAG00000013226 |  |  |
| ENSBTAG00000010071 | ENSBTAG00000013227 |  |  |
| ENSBTAG00000010077 | ENSBTAG00000013231 |  |  |
| ENSBTAG00000010082 | ENSBTAG00000013242 |  |  |
| ENSBTAG00000010091 | ENSBTAG00000013244 |  |  |
| ENSBTAG00000010094 | ENSBTAG00000013247 |  |  |
| ENSBTAG00000010100 | ENSBTAG00000013249 |  |  |
| ENSBTAG00000010101 | ENSBTAG00000013259 |  |  |
| ENSBTAG00000010103 | ENSBTAG00000013270 |  |  |
| ENSBTAG00000010106 | ENSBTAG00000013271 |  |  |
| ENSBTAG00000010120 | ENSBTAG00000013282 |  |  |
| ENSBTAG00000010124 | ENSBTAG00000013284 |  |  |
| ENSBTAG00000010126 | ENSBTAG00000013287 |  |  |
| ENSBTAG00000010128 | ENSBTAG00000013291 |  |  |
| ENSBTAG00000010130 | ENSBTAG00000013298 |  |  |
| ENSBTAG00000010134 | ENSBTAG00000013301 |  |  |
| ENSBTAG00000010135 | ENSBTAG00000013303 |  |  |
| ENSBTAG00000010145 | ENSBTAG00000013309 |  |  |
| ENSBTAG00000010146 | ENSBTAG00000013314 |  |  |
| ENSBTAG00000010152 | ENSBTAG00000013316 |  |  |
| ENSBTAG00000010153 | ENSBTAG00000013317 |  |  |
| ENSBTAG00000010155 | ENSBTAG00000013319 |  |  |
| ENSBTAG00000010161 | ENSBTAG00000013320 |  |  |
| ENSBTAG00000010165 | ENSBTAG00000013322 |  |  |
| ENSBTAG00000010170 | ENSBTAG00000013329 |  |  |
| ENSBTAG00000010171 | ENSBTAG00000013330 |  |  |
| ENSBTAG00000010174 | ENSBTAG00000013333 |  |  |
| ENSBTAG00000010177 | ENSBTAG00000013336 |  |  |
| ENSBTAG00000010179 | ENSBTAG00000013337 |  |  |
| ENSBTAG00000010180 | ENSBTAG00000013341 |  |  |
| ENSBTAG00000010181 | ENSBTAG00000013362 |  |  |
| ENSBTAG00000010184 | ENSBTAG00000013363 |  |  |
| ENSBTAG00000010188 | ENSBTAG00000013366 |  |  |
| ENSBTAG00000010191 | ENSBTAG00000013367 |  |  |
| ENSBTAG00000010193 | ENSBTAG00000013368 |  |  |
| ENSBTAG00000010195 | ENSBTAG00000013371 |  |  |
| ENSBTAG00000010196 | ENSBTAG00000013387 |  |  |
| ENSBTAG00000010206 | ENSBTAG00000013391 |  |  |
| ENSBTAG00000010207 | ENSBTAG00000013400 |  |  |
| ENSBTAG00000010210 | ENSBTAG00000013402 |  |  |
| ENSBTAG00000010221 | ENSBTAG00000013406 |  |  |
| ENSBTAG00000010223 | ENSBTAG00000013411 |  |  |
| ENSBTAG00000010225 | ENSBTAG00000013414 |  |  |
| ENSBTAG00000010227 | ENSBTAG00000013419 |  |  |
| ENSBTAG00000010228 | ENSBTAG00000013420 |  |  |
| ENSBTAG00000010230 | ENSBTAG00000013421 |  |  |
| ENSBTAG00000010234 | ENSBTAG00000013422 |  |  |
| ENSBTAG00000010235 | ENSBTAG00000013423 |  |  |
| ENSBTAG00000010236 | ENSBTAG00000013426 |  |  |
| ENSBTAG00000010238 | ENSBTAG00000013436 |  |  |
| ENSBTAG00000010242 | ENSBTAG00000013442 |  |  |
| ENSBTAG00000010243 | ENSBTAG00000013443 |  |  |
| ENSBTAG00000010246 | ENSBTAG00000013449 |  |  |
| ENSBTAG00000010247 | ENSBTAG00000013454 |  |  |
| ENSBTAG00000010248 | ENSBTAG00000013456 |  |  |
| ENSBTAG00000010249 | ENSBTAG00000013462 |  |  |
| ENSBTAG00000010254 | ENSBTAG00000013464 |  |  |
| ENSBTAG00000010265 | ENSBTAG00000013472 |  |  |
| ENSBTAG00000010271 | ENSBTAG00000013477 |  |  |
| ENSBTAG00000010273 | ENSBTAG00000013478 |  |  |
| ENSBTAG00000010276 | ENSBTAG00000013479 |  |  |
| ENSBTAG00000010277 | ENSBTAG00000013483 |  |  |
| ENSBTAG00000010278 | ENSBTAG00000013492 |  |  |
| ENSBTAG00000010279 | ENSBTAG00000013495 |  |  |
| ENSBTAG00000010284 | ENSBTAG00000013496 |  |  |
| ENSBTAG00000010285 | ENSBTAG00000013498 |  |  |
| ENSBTAG00000010289 | ENSBTAG00000013499 |  |  |
| ENSBTAG00000010291 | ENSBTAG00000013502 |  |  |
| ENSBTAG00000010292 | ENSBTAG00000013507 |  |  |
| ENSBTAG00000010297 | ENSBTAG00000013510 |  |  |
| ENSBTAG00000010299 | ENSBTAG00000013513 |  |  |
| ENSBTAG00000010304 | ENSBTAG00000013515 |  |  |
| ENSBTAG00000010313 | ENSBTAG00000013525 |  |  |
| ENSBTAG00000010316 | ENSBTAG00000013526 |  |  |
| ENSBTAG00000010321 | ENSBTAG00000013528 |  |  |
| ENSBTAG00000010324 | ENSBTAG00000013531 |  |  |
| ENSBTAG00000010326 | ENSBTAG00000013534 |  |  |
| ENSBTAG00000010328 | ENSBTAG00000013535 |  |  |
| ENSBTAG00000010330 | ENSBTAG00000013538 |  |  |
| ENSBTAG00000010336 | ENSBTAG00000013541 |  |  |
| ENSBTAG00000010337 | ENSBTAG00000013544 |  |  |
| ENSBTAG00000010338 | ENSBTAG00000013545 |  |  |
| ENSBTAG00000010339 | ENSBTAG00000013550 |  |  |
| ENSBTAG00000010347 | ENSBTAG00000013556 |  |  |
| ENSBTAG00000010348 | ENSBTAG00000013569 |  |  |
| ENSBTAG00000010349 | ENSBTAG00000013573 |  |  |
| ENSBTAG00000010350 | ENSBTAG00000013579 |  |  |
| ENSBTAG00000010351 | ENSBTAG00000013580 |  |  |
| ENSBTAG00000010353 | ENSBTAG00000013588 |  |  |
| ENSBTAG00000010355 | ENSBTAG00000013589 |  |  |
| ENSBTAG00000010356 | ENSBTAG00000013593 |  |  |
| ENSBTAG00000010359 | ENSBTAG00000013596 |  |  |
| ENSBTAG00000010360 | ENSBTAG00000013602 |  |  |
| ENSBTAG00000010361 | ENSBTAG00000013607 |  |  |
| ENSBTAG00000010362 | ENSBTAG00000013611 |  |  |
| ENSBTAG00000010365 | ENSBTAG00000013612 |  |  |
| ENSBTAG00000010367 | ENSBTAG00000013614 |  |  |
| ENSBTAG00000010368 | ENSBTAG00000013615 |  |  |
| ENSBTAG00000010370 | ENSBTAG00000013620 |  |  |
| ENSBTAG00000010371 | ENSBTAG00000013621 |  |  |
| ENSBTAG00000010372 | ENSBTAG00000013623 |  |  |
| ENSBTAG00000010376 | ENSBTAG00000013629 |  |  |
| ENSBTAG00000010378 | ENSBTAG00000013631 |  |  |
| ENSBTAG00000010381 | ENSBTAG00000013641 |  |  |
| ENSBTAG00000010383 | ENSBTAG00000013642 |  |  |
| ENSBTAG00000010384 | ENSBTAG00000013645 |  |  |
| ENSBTAG00000010386 | ENSBTAG00000013648 |  |  |
| ENSBTAG00000010387 | ENSBTAG00000013652 |  |  |
| ENSBTAG00000010390 | ENSBTAG00000013653 |  |  |
| ENSBTAG00000010392 | ENSBTAG00000013662 |  |  |
| ENSBTAG00000010395 | ENSBTAG00000013666 |  |  |
| ENSBTAG00000010397 | ENSBTAG00000013670 |  |  |
| ENSBTAG00000010401 | ENSBTAG00000013674 |  |  |
| ENSBTAG00000010402 | ENSBTAG00000013675 |  |  |
| ENSBTAG00000010403 | ENSBTAG00000013685 |  |  |
| ENSBTAG00000010408 | ENSBTAG00000013720 |  |  |
| ENSBTAG00000010413 | ENSBTAG00000013721 |  |  |
| ENSBTAG00000010419 | ENSBTAG00000013724 |  |  |
| ENSBTAG00000010422 | ENSBTAG00000013730 |  |  |
| ENSBTAG00000010427 | ENSBTAG00000013740 |  |  |
| ENSBTAG00000010431 | ENSBTAG00000013741 |  |  |
| ENSBTAG00000010437 | ENSBTAG00000013745 |  |  |
| ENSBTAG00000010442 | ENSBTAG00000013750 |  |  |
| ENSBTAG00000010444 | ENSBTAG00000013753 |  |  |
| ENSBTAG00000010447 | ENSBTAG00000013757 |  |  |
| ENSBTAG00000010449 | ENSBTAG00000013774 |  |  |
| ENSBTAG00000010451 | ENSBTAG00000013776 |  |  |
| ENSBTAG00000010452 | ENSBTAG00000013778 |  |  |
| ENSBTAG00000010455 | ENSBTAG00000013782 |  |  |
| ENSBTAG00000010456 | ENSBTAG00000013812 |  |  |
| ENSBTAG00000010458 | ENSBTAG00000013813 |  |  |
| ENSBTAG00000010460 | ENSBTAG00000013819 |  |  |
| ENSBTAG00000010462 | ENSBTAG00000013825 |  |  |
| ENSBTAG00000010465 | ENSBTAG00000013830 |  |  |
| ENSBTAG00000010470 | ENSBTAG00000013831 |  |  |
| ENSBTAG00000010484 | ENSBTAG00000013832 |  |  |
| ENSBTAG00000010485 | ENSBTAG00000013842 |  |  |
| ENSBTAG00000010487 | ENSBTAG00000013843 |  |  |
| ENSBTAG00000010490 | ENSBTAG00000013848 |  |  |
| ENSBTAG00000010492 | ENSBTAG00000013849 |  |  |
| ENSBTAG00000010493 | ENSBTAG00000013851 |  |  |
| ENSBTAG00000010496 | ENSBTAG00000013854 |  |  |
| ENSBTAG00000010498 | ENSBTAG00000013855 |  |  |
| ENSBTAG00000010500 | ENSBTAG00000013860 |  |  |
| ENSBTAG00000010501 | ENSBTAG00000013863 |  |  |
| ENSBTAG00000010502 | ENSBTAG00000013869 |  |  |
| ENSBTAG00000010503 | ENSBTAG00000013880 |  |  |
| ENSBTAG00000010505 | ENSBTAG00000013885 |  |  |
| ENSBTAG00000010506 | ENSBTAG00000013889 |  |  |
| ENSBTAG00000010508 | ENSBTAG00000013895 |  |  |
| ENSBTAG00000010509 | ENSBTAG00000013901 |  |  |
| ENSBTAG00000010513 | ENSBTAG00000013907 |  |  |
| ENSBTAG00000010515 | ENSBTAG00000013910 |  |  |
| ENSBTAG00000010519 | ENSBTAG00000013912 |  |  |
| ENSBTAG00000010524 | ENSBTAG00000013914 |  |  |
| ENSBTAG00000010529 | ENSBTAG00000013915 |  |  |
| ENSBTAG00000010530 | ENSBTAG00000013916 |  |  |
| ENSBTAG00000010532 | ENSBTAG00000013919 |  |  |
| ENSBTAG00000010533 | ENSBTAG00000013923 |  |  |
| ENSBTAG00000010534 | ENSBTAG00000013926 |  |  |
| ENSBTAG00000010535 | ENSBTAG00000013928 |  |  |
| ENSBTAG00000010541 | ENSBTAG00000013929 |  |  |
| ENSBTAG00000010543 | ENSBTAG00000013932 |  |  |
| ENSBTAG00000010545 | ENSBTAG00000013935 |  |  |
| ENSBTAG00000010548 | ENSBTAG00000013949 |  |  |
| ENSBTAG00000010549 | ENSBTAG00000013952 |  |  |
| ENSBTAG00000010551 | ENSBTAG00000013955 |  |  |
| ENSBTAG00000010552 | ENSBTAG00000013956 |  |  |
| ENSBTAG00000010555 | ENSBTAG00000013957 |  |  |
| ENSBTAG00000010559 | ENSBTAG00000013960 |  |  |
| ENSBTAG00000010563 | ENSBTAG00000013974 |  |  |
| ENSBTAG00000010564 | ENSBTAG00000013981 |  |  |
| ENSBTAG00000010566 | ENSBTAG00000013985 |  |  |
| ENSBTAG00000010576 | ENSBTAG00000013999 |  |  |
| ENSBTAG00000010579 | ENSBTAG00000014005 |  |  |
| ENSBTAG00000010582 | ENSBTAG00000014006 |  |  |
| ENSBTAG00000010584 | ENSBTAG00000014007 |  |  |
| ENSBTAG00000010587 | ENSBTAG00000014011 |  |  |
| ENSBTAG00000010590 | ENSBTAG00000014012 |  |  |
| ENSBTAG00000010593 | ENSBTAG00000014017 |  |  |
| ENSBTAG00000010595 | ENSBTAG00000014021 |  |  |
| ENSBTAG00000010597 | ENSBTAG00000014023 |  |  |
| ENSBTAG00000010601 | ENSBTAG00000014024 |  |  |
| ENSBTAG00000010602 | ENSBTAG00000014029 |  |  |
| ENSBTAG00000010604 | ENSBTAG00000014030 |  |  |
| ENSBTAG00000010610 | ENSBTAG00000014032 |  |  |
| ENSBTAG00000010611 | ENSBTAG00000014041 |  |  |
| ENSBTAG00000010612 | ENSBTAG00000014042 |  |  |
| ENSBTAG00000010613 | ENSBTAG00000014043 |  |  |
| ENSBTAG00000010616 | ENSBTAG00000014044 |  |  |
| ENSBTAG00000010619 | ENSBTAG00000014053 |  |  |
| ENSBTAG00000010620 | ENSBTAG00000014054 |  |  |
| ENSBTAG00000010622 | ENSBTAG00000014057 |  |  |
| ENSBTAG00000010624 | ENSBTAG00000014060 |  |  |
| ENSBTAG00000010626 | ENSBTAG00000014064 |  |  |
| ENSBTAG00000010627 | ENSBTAG00000014068 |  |  |
| ENSBTAG00000010635 | ENSBTAG00000014069 |  |  |
| ENSBTAG00000010641 | ENSBTAG00000014078 |  |  |
| ENSBTAG00000010647 | ENSBTAG00000014079 |  |  |
| ENSBTAG00000010649 | ENSBTAG00000014083 |  |  |
| ENSBTAG00000010653 | ENSBTAG00000014089 |  |  |
| ENSBTAG00000010657 | ENSBTAG00000014090 |  |  |
| ENSBTAG00000010658 | ENSBTAG00000014091 |  |  |
| ENSBTAG00000010661 | ENSBTAG00000014092 |  |  |
| ENSBTAG00000010662 | ENSBTAG00000014093 |  |  |
| ENSBTAG00000010664 | ENSBTAG00000014102 |  |  |
| ENSBTAG00000010665 | ENSBTAG00000014103 |  |  |
| ENSBTAG00000010666 | ENSBTAG00000014106 |  |  |
| ENSBTAG00000010667 | ENSBTAG00000014113 |  |  |
| ENSBTAG00000010673 | ENSBTAG00000014118 |  |  |
| ENSBTAG00000010677 | ENSBTAG00000014122 |  |  |
| ENSBTAG00000010681 | ENSBTAG00000014124 |  |  |
| ENSBTAG00000010682 | ENSBTAG00000014127 |  |  |
| ENSBTAG00000010688 | ENSBTAG00000014136 |  |  |
| ENSBTAG00000010692 | ENSBTAG00000014140 |  |  |
| ENSBTAG00000010693 | ENSBTAG00000014143 |  |  |
| ENSBTAG00000010694 | ENSBTAG00000014147 |  |  |
| ENSBTAG00000010704 | ENSBTAG00000014151 |  |  |
| ENSBTAG00000010717 | ENSBTAG00000014156 |  |  |
| ENSBTAG00000010719 | ENSBTAG00000014158 |  |  |
| ENSBTAG00000010721 | ENSBTAG00000014159 |  |  |
| ENSBTAG00000010726 | ENSBTAG00000014166 |  |  |
| ENSBTAG00000010727 | ENSBTAG00000014175 |  |  |
| ENSBTAG00000010729 | ENSBTAG00000014176 |  |  |
| ENSBTAG00000010731 | ENSBTAG00000014179 |  |  |
| ENSBTAG00000010734 | ENSBTAG00000014181 |  |  |
| ENSBTAG00000010735 | ENSBTAG00000014187 |  |  |
| ENSBTAG00000010736 | ENSBTAG00000014191 |  |  |
| ENSBTAG00000010737 | ENSBTAG00000014192 |  |  |
| ENSBTAG00000010738 | ENSBTAG00000014197 |  |  |
| ENSBTAG00000010739 | ENSBTAG00000014199 |  |  |
| ENSBTAG00000010742 | ENSBTAG00000014204 |  |  |
| ENSBTAG00000010745 | ENSBTAG00000014215 |  |  |
| ENSBTAG00000010756 | ENSBTAG00000014217 |  |  |
| ENSBTAG00000010758 | ENSBTAG00000014218 |  |  |
| ENSBTAG00000010760 | ENSBTAG00000014220 |  |  |
| ENSBTAG00000010773 | ENSBTAG00000014227 |  |  |
| ENSBTAG00000010774 | ENSBTAG00000014229 |  |  |
| ENSBTAG00000010775 | ENSBTAG00000014232 |  |  |
| ENSBTAG00000010777 | ENSBTAG00000014234 |  |  |
| ENSBTAG00000010778 | ENSBTAG00000014235 |  |  |
| ENSBTAG00000010784 | ENSBTAG00000014237 |  |  |
| ENSBTAG00000010785 | ENSBTAG00000014238 |  |  |
| ENSBTAG00000010786 | ENSBTAG00000014239 |  |  |
| ENSBTAG00000010789 | ENSBTAG00000014246 |  |  |
| ENSBTAG00000010790 | ENSBTAG00000014249 |  |  |
| ENSBTAG00000010792 | ENSBTAG00000014251 |  |  |
| ENSBTAG00000010795 | ENSBTAG00000014253 |  |  |
| ENSBTAG00000010799 | ENSBTAG00000014255 |  |  |
| ENSBTAG00000010802 | ENSBTAG00000014259 |  |  |
| ENSBTAG00000010805 | ENSBTAG00000014261 |  |  |
| ENSBTAG00000010809 | ENSBTAG00000014262 |  |  |
| ENSBTAG00000010810 | ENSBTAG00000014272 |  |  |
| ENSBTAG00000010812 | ENSBTAG00000014273 |  |  |
| ENSBTAG00000010818 | ENSBTAG00000014287 |  |  |
| ENSBTAG00000010819 | ENSBTAG00000014295 |  |  |
| ENSBTAG00000010820 | ENSBTAG00000014302 |  |  |
| ENSBTAG00000010822 | ENSBTAG00000014306 |  |  |
| ENSBTAG00000010830 | ENSBTAG00000014312 |  |  |
| ENSBTAG00000010832 | ENSBTAG00000014314 |  |  |
| ENSBTAG00000010835 | ENSBTAG00000014316 |  |  |
| ENSBTAG00000010837 | ENSBTAG00000014318 |  |  |
| ENSBTAG00000010841 | ENSBTAG00000014324 |  |  |
| ENSBTAG00000010843 | ENSBTAG00000014325 |  |  |
| ENSBTAG00000010846 | ENSBTAG00000014326 |  |  |
| ENSBTAG00000010850 | ENSBTAG00000014328 |  |  |
| ENSBTAG00000010860 | ENSBTAG00000014329 |  |  |
| ENSBTAG00000010863 | ENSBTAG00000014333 |  |  |
| ENSBTAG00000010865 | ENSBTAG00000014335 |  |  |
| ENSBTAG00000010866 | ENSBTAG00000014340 |  |  |
| ENSBTAG00000010867 | ENSBTAG00000014345 |  |  |
| ENSBTAG00000010868 | ENSBTAG00000014349 |  |  |
| ENSBTAG00000010871 | ENSBTAG00000014353 |  |  |
| ENSBTAG00000010875 | ENSBTAG00000014359 |  |  |
| ENSBTAG00000010878 | ENSBTAG00000014366 |  |  |
| ENSBTAG00000010885 | ENSBTAG00000014367 |  |  |
| ENSBTAG00000010888 | ENSBTAG00000014374 |  |  |
| ENSBTAG00000010889 | ENSBTAG00000014375 |  |  |
| ENSBTAG00000010890 | ENSBTAG00000014377 |  |  |
| ENSBTAG00000010897 | ENSBTAG00000014380 |  |  |
| ENSBTAG00000010898 | ENSBTAG00000014382 |  |  |
| ENSBTAG00000010906 | ENSBTAG00000014388 |  |  |
| ENSBTAG00000010907 | ENSBTAG00000014393 |  |  |
| ENSBTAG00000010910 | ENSBTAG00000014395 |  |  |
| ENSBTAG00000010913 | ENSBTAG00000014396 |  |  |
| ENSBTAG00000010916 | ENSBTAG00000014411 |  |  |
| ENSBTAG00000010919 | ENSBTAG00000014417 |  |  |
| ENSBTAG00000010923 | ENSBTAG00000014420 |  |  |
| ENSBTAG00000010924 | ENSBTAG00000014422 |  |  |
| ENSBTAG00000010932 | ENSBTAG00000014423 |  |  |
| ENSBTAG00000010934 | ENSBTAG00000014424 |  |  |
| ENSBTAG00000010937 | ENSBTAG00000014432 |  |  |
| ENSBTAG00000010938 | ENSBTAG00000014433 |  |  |
| ENSBTAG00000010943 | ENSBTAG00000014435 |  |  |
| ENSBTAG00000010944 | ENSBTAG00000014438 |  |  |
| ENSBTAG00000010945 | ENSBTAG00000014441 |  |  |
| ENSBTAG00000010947 | ENSBTAG00000014442 |  |  |
| ENSBTAG00000010948 | ENSBTAG00000014451 |  |  |
| ENSBTAG00000010951 | ENSBTAG00000014458 |  |  |
| ENSBTAG00000010955 | ENSBTAG00000014459 |  |  |
| ENSBTAG00000010956 | ENSBTAG00000014460 |  |  |
| ENSBTAG00000010959 | ENSBTAG00000014461 |  |  |
| ENSBTAG00000010963 | ENSBTAG00000014463 |  |  |
| ENSBTAG00000010964 | ENSBTAG00000014465 |  |  |
| ENSBTAG00000010967 | ENSBTAG00000014466 |  |  |
| ENSBTAG00000010968 | ENSBTAG00000014468 |  |  |
| ENSBTAG00000010971 | ENSBTAG00000014469 |  |  |
| ENSBTAG00000010976 | ENSBTAG00000014471 |  |  |
| ENSBTAG00000010977 | ENSBTAG00000014476 |  |  |
| ENSBTAG00000010980 | ENSBTAG00000014479 |  |  |
| ENSBTAG00000010987 | ENSBTAG00000014482 |  |  |
| ENSBTAG00000010990 | ENSBTAG00000014485 |  |  |
| ENSBTAG00000010991 | ENSBTAG00000014486 |  |  |
| ENSBTAG00000010992 | ENSBTAG00000014490 |  |  |
| ENSBTAG00000010993 | ENSBTAG00000014491 |  |  |
| ENSBTAG00000010994 | ENSBTAG00000014494 |  |  |
| ENSBTAG00000010995 | ENSBTAG00000014495 |  |  |
| ENSBTAG00000011001 | ENSBTAG00000014501 |  |  |
| ENSBTAG00000011007 | ENSBTAG00000014503 |  |  |
| ENSBTAG00000011010 | ENSBTAG00000014505 |  |  |
| ENSBTAG00000011012 | ENSBTAG00000014511 |  |  |
| ENSBTAG00000011017 | ENSBTAG00000014514 |  |  |
| ENSBTAG00000011019 | ENSBTAG00000014518 |  |  |
| ENSBTAG00000011021 | ENSBTAG00000014520 |  |  |
| ENSBTAG00000011022 | ENSBTAG00000014521 |  |  |
| ENSBTAG00000011023 | ENSBTAG00000014522 |  |  |
| ENSBTAG00000011024 | ENSBTAG00000014523 |  |  |
| ENSBTAG00000011027 | ENSBTAG00000014525 |  |  |
| ENSBTAG00000011028 | ENSBTAG00000014526 |  |  |
| ENSBTAG00000011032 | ENSBTAG00000014529 |  |  |
| ENSBTAG00000011034 | ENSBTAG00000014530 |  |  |
| ENSBTAG00000011037 | ENSBTAG00000014534 |  |  |
| ENSBTAG00000011038 | ENSBTAG00000014536 |  |  |
| ENSBTAG00000011042 | ENSBTAG00000014538 |  |  |
| ENSBTAG00000011043 | ENSBTAG00000014540 |  |  |
| ENSBTAG00000011044 | ENSBTAG00000014541 |  |  |
| ENSBTAG00000011046 | ENSBTAG00000014546 |  |  |
| ENSBTAG00000011048 | ENSBTAG00000014550 |  |  |
| ENSBTAG00000011059 | ENSBTAG00000014551 |  |  |
| ENSBTAG00000011062 | ENSBTAG00000014553 |  |  |
| ENSBTAG00000011064 | ENSBTAG00000014554 |  |  |
| ENSBTAG00000011070 | ENSBTAG00000014558 |  |  |
| ENSBTAG00000011071 | ENSBTAG00000014560 |  |  |
| ENSBTAG00000011072 | ENSBTAG00000014561 |  |  |
| ENSBTAG00000011075 | ENSBTAG00000014576 |  |  |
| ENSBTAG00000011076 | ENSBTAG00000014579 |  |  |
| ENSBTAG00000011079 | ENSBTAG00000014581 |  |  |
| ENSBTAG00000011082 | ENSBTAG00000014583 |  |  |
| ENSBTAG00000011087 | ENSBTAG00000014592 |  |  |
| ENSBTAG00000011095 | ENSBTAG00000014598 |  |  |
| ENSBTAG00000011100 | ENSBTAG00000014601 |  |  |
| ENSBTAG00000011101 | ENSBTAG00000014602 |  |  |
| ENSBTAG00000011102 | ENSBTAG00000014603 |  |  |
| ENSBTAG00000011103 | ENSBTAG00000014608 |  |  |
| ENSBTAG00000011104 | ENSBTAG00000014611 |  |  |
| ENSBTAG00000011105 | ENSBTAG00000014612 |  |  |
| ENSBTAG00000011108 | ENSBTAG00000014619 |  |  |
| ENSBTAG00000011111 | ENSBTAG00000014623 |  |  |
| ENSBTAG00000011115 | ENSBTAG00000014628 |  |  |
| ENSBTAG00000011116 | ENSBTAG00000014633 |  |  |
| ENSBTAG00000011121 | ENSBTAG00000014643 |  |  |
| ENSBTAG00000011124 | ENSBTAG00000014644 |  |  |
| ENSBTAG00000011125 | ENSBTAG00000014653 |  |  |
| ENSBTAG00000011126 | ENSBTAG00000014655 |  |  |
| ENSBTAG00000011129 | ENSBTAG00000014659 |  |  |
| ENSBTAG00000011131 | ENSBTAG00000014665 |  |  |
| ENSBTAG00000011133 | ENSBTAG00000014675 |  |  |
| ENSBTAG00000011135 | ENSBTAG00000014677 |  |  |
| ENSBTAG00000011136 | ENSBTAG00000014679 |  |  |
| ENSBTAG00000011138 | ENSBTAG00000014689 |  |  |
| ENSBTAG00000011139 | ENSBTAG00000014691 |  |  |
| ENSBTAG00000011143 | ENSBTAG00000014692 |  |  |
| ENSBTAG00000011145 | ENSBTAG00000014698 |  |  |
| ENSBTAG00000011146 | ENSBTAG00000014699 |  |  |
| ENSBTAG00000011147 | ENSBTAG00000014700 |  |  |
| ENSBTAG00000011149 | ENSBTAG00000014711 |  |  |
| ENSBTAG00000011150 | ENSBTAG00000014714 |  |  |
| ENSBTAG00000011154 | ENSBTAG00000014719 |  |  |
| ENSBTAG00000011170 | ENSBTAG00000014725 |  |  |
| ENSBTAG00000011171 | ENSBTAG00000014726 |  |  |
| ENSBTAG00000011176 | ENSBTAG00000014728 |  |  |
| ENSBTAG00000011179 | ENSBTAG00000014729 |  |  |
| ENSBTAG00000011180 | ENSBTAG00000014733 |  |  |
| ENSBTAG00000011182 | ENSBTAG00000014734 |  |  |
| ENSBTAG00000011184 | ENSBTAG00000014738 |  |  |
| ENSBTAG00000011186 | ENSBTAG00000014750 |  |  |
| ENSBTAG00000011187 | ENSBTAG00000014758 |  |  |
| ENSBTAG00000011189 | ENSBTAG00000014766 |  |  |
| ENSBTAG00000011190 | ENSBTAG00000014769 |  |  |
| ENSBTAG00000011193 | ENSBTAG00000014772 |  |  |
| ENSBTAG00000011196 | ENSBTAG00000014773 |  |  |
| ENSBTAG00000011197 | ENSBTAG00000014777 |  |  |
| ENSBTAG00000011198 | ENSBTAG00000014791 |  |  |
| ENSBTAG00000011200 | ENSBTAG00000014792 |  |  |
| ENSBTAG00000011204 | ENSBTAG00000014794 |  |  |
| ENSBTAG00000011206 | ENSBTAG00000014804 |  |  |
| ENSBTAG00000011207 | ENSBTAG00000014805 |  |  |
| ENSBTAG00000011209 | ENSBTAG00000014806 |  |  |
| ENSBTAG00000011214 | ENSBTAG00000014807 |  |  |
| ENSBTAG00000011217 | ENSBTAG00000014809 |  |  |
| ENSBTAG00000011224 | ENSBTAG00000014820 |  |  |
| ENSBTAG00000011225 | ENSBTAG00000014821 |  |  |
| ENSBTAG00000011227 | ENSBTAG00000014822 |  |  |
| ENSBTAG00000011228 | ENSBTAG00000014825 |  |  |
| ENSBTAG00000011233 | ENSBTAG00000014829 |  |  |
| ENSBTAG00000011238 | ENSBTAG00000014830 |  |  |
| ENSBTAG00000011239 | ENSBTAG00000014831 |  |  |
| ENSBTAG00000011241 | ENSBTAG00000014832 |  |  |
| ENSBTAG00000011243 | ENSBTAG00000014835 |  |  |
| ENSBTAG00000011245 | ENSBTAG00000014838 |  |  |
| ENSBTAG00000011246 | ENSBTAG00000014841 |  |  |
| ENSBTAG00000011247 | ENSBTAG00000014847 |  |  |
| ENSBTAG00000011250 | ENSBTAG00000014850 |  |  |
| ENSBTAG00000011256 | ENSBTAG00000014858 |  |  |
| ENSBTAG00000011257 | ENSBTAG00000014861 |  |  |
| ENSBTAG00000011258 | ENSBTAG00000014872 |  |  |
| ENSBTAG00000011263 | ENSBTAG00000014874 |  |  |
| ENSBTAG00000011266 | ENSBTAG00000014877 |  |  |
| ENSBTAG00000011268 | ENSBTAG00000014879 |  |  |
| ENSBTAG00000011274 | ENSBTAG00000014881 |  |  |
| ENSBTAG00000011275 | ENSBTAG00000014883 |  |  |
| ENSBTAG00000011278 | ENSBTAG00000014884 |  |  |
| ENSBTAG00000011284 | ENSBTAG00000014890 |  |  |
| ENSBTAG00000011285 | ENSBTAG00000014891 |  |  |
| ENSBTAG00000011291 | ENSBTAG00000014911 |  |  |
| ENSBTAG00000011304 | ENSBTAG00000014912 |  |  |
| ENSBTAG00000011307 | ENSBTAG00000014915 |  |  |
| ENSBTAG00000011311 | ENSBTAG00000014916 |  |  |
| ENSBTAG00000011313 | ENSBTAG00000014917 |  |  |
| ENSBTAG00000011316 | ENSBTAG00000014922 |  |  |
| ENSBTAG00000011319 | ENSBTAG00000014930 |  |  |
| ENSBTAG00000011322 | ENSBTAG00000014936 |  |  |
| ENSBTAG00000011325 | ENSBTAG00000014940 |  |  |
| ENSBTAG00000011327 | ENSBTAG00000014943 |  |  |
| ENSBTAG00000011328 | ENSBTAG00000014958 |  |  |
| ENSBTAG00000011338 | ENSBTAG00000014960 |  |  |
| ENSBTAG00000011339 | ENSBTAG00000014969 |  |  |
| ENSBTAG00000011340 | ENSBTAG00000014972 |  |  |
| ENSBTAG00000011344 | ENSBTAG00000014974 |  |  |
| ENSBTAG00000011350 | ENSBTAG00000014981 |  |  |
| ENSBTAG00000011354 | ENSBTAG00000015000 |  |  |
| ENSBTAG00000011356 | ENSBTAG00000015008 |  |  |
| ENSBTAG00000011358 | ENSBTAG00000015012 |  |  |
| ENSBTAG00000011359 | ENSBTAG00000015013 |  |  |
| ENSBTAG00000011366 | ENSBTAG00000015014 |  |  |
| ENSBTAG00000011374 | ENSBTAG00000015015 |  |  |
| ENSBTAG00000011375 | ENSBTAG00000015016 |  |  |
| ENSBTAG00000011381 | ENSBTAG00000015017 |  |  |
| ENSBTAG00000011382 | ENSBTAG00000015019 |  |  |
| ENSBTAG00000011383 | ENSBTAG00000015023 |  |  |
| ENSBTAG00000011387 | ENSBTAG00000015025 |  |  |
| ENSBTAG00000011389 | ENSBTAG00000015026 |  |  |
| ENSBTAG00000011390 | ENSBTAG00000015032 |  |  |
| ENSBTAG00000011392 | ENSBTAG00000015036 |  |  |
| ENSBTAG00000011394 | ENSBTAG00000015038 |  |  |
| ENSBTAG00000011395 | ENSBTAG00000015041 |  |  |
| ENSBTAG00000011396 | ENSBTAG00000015043 |  |  |
| ENSBTAG00000011397 | ENSBTAG00000015046 |  |  |
| ENSBTAG00000011399 | ENSBTAG00000015053 |  |  |
| ENSBTAG00000011400 | ENSBTAG00000015056 |  |  |
| ENSBTAG00000011401 | ENSBTAG00000015066 |  |  |
| ENSBTAG00000011403 | ENSBTAG00000015069 |  |  |
| ENSBTAG00000011406 | ENSBTAG00000015073 |  |  |
| ENSBTAG00000011409 | ENSBTAG00000015076 |  |  |
| ENSBTAG00000011411 | ENSBTAG00000015080 |  |  |
| ENSBTAG00000011414 | ENSBTAG00000015083 |  |  |
| ENSBTAG00000011415 | ENSBTAG00000015085 |  |  |
| ENSBTAG00000011416 | ENSBTAG00000015086 |  |  |
| ENSBTAG00000011417 | ENSBTAG00000015090 |  |  |
| ENSBTAG00000011419 | ENSBTAG00000015092 |  |  |
| ENSBTAG00000011421 | ENSBTAG00000015097 |  |  |
| ENSBTAG00000011424 | ENSBTAG00000015104 |  |  |
| ENSBTAG00000011425 | ENSBTAG00000015105 |  |  |
| ENSBTAG00000011427 | ENSBTAG00000015106 |  |  |
| ENSBTAG00000011429 | ENSBTAG00000015107 |  |  |
| ENSBTAG00000011431 | ENSBTAG00000015109 |  |  |
| ENSBTAG00000011433 | ENSBTAG00000015113 |  |  |
| ENSBTAG00000011435 | ENSBTAG00000015114 |  |  |
| ENSBTAG00000011445 | ENSBTAG00000015127 |  |  |
| ENSBTAG00000011451 | ENSBTAG00000015130 |  |  |
| ENSBTAG00000011454 | ENSBTAG00000015132 |  |  |
| ENSBTAG00000011455 | ENSBTAG00000015133 |  |  |
| ENSBTAG00000011458 | ENSBTAG00000015136 |  |  |
| ENSBTAG00000011461 | ENSBTAG00000015144 |  |  |
| ENSBTAG00000011463 | ENSBTAG00000015145 |  |  |
| ENSBTAG00000011465 | ENSBTAG00000015146 |  |  |
| ENSBTAG00000011467 | ENSBTAG00000015151 |  |  |
| ENSBTAG00000011473 | ENSBTAG00000015154 |  |  |
| ENSBTAG00000011476 | ENSBTAG00000015155 |  |  |
| ENSBTAG00000011479 | ENSBTAG00000015169 |  |  |
| ENSBTAG00000011480 | ENSBTAG00000015177 |  |  |
| ENSBTAG00000011481 | ENSBTAG00000015181 |  |  |
| ENSBTAG00000011482 | ENSBTAG00000015184 |  |  |
| ENSBTAG00000011484 | ENSBTAG00000015186 |  |  |
| ENSBTAG00000011487 | ENSBTAG00000015188 |  |  |
| ENSBTAG00000011488 | ENSBTAG00000015195 |  |  |
| ENSBTAG00000011490 | ENSBTAG00000015200 |  |  |
| ENSBTAG00000011491 | ENSBTAG00000015202 |  |  |
| ENSBTAG00000011495 | ENSBTAG00000015204 |  |  |
| ENSBTAG00000011498 | ENSBTAG00000015205 |  |  |
| ENSBTAG00000011500 | ENSBTAG00000015209 |  |  |
| ENSBTAG00000011505 | ENSBTAG00000015212 |  |  |
| ENSBTAG00000011507 | ENSBTAG00000015214 |  |  |
| ENSBTAG00000011509 | ENSBTAG00000015217 |  |  |
| ENSBTAG00000011514 | ENSBTAG00000015220 |  |  |
| ENSBTAG00000011516 | ENSBTAG00000015222 |  |  |
| ENSBTAG00000011517 | ENSBTAG00000015225 |  |  |
| ENSBTAG00000011528 | ENSBTAG00000015230 |  |  |
| ENSBTAG00000011530 | ENSBTAG00000015232 |  |  |
| ENSBTAG00000011531 | ENSBTAG00000015238 |  |  |
| ENSBTAG00000011533 | ENSBTAG00000015240 |  |  |
| ENSBTAG00000011539 | ENSBTAG00000015248 |  |  |
| ENSBTAG00000011540 | ENSBTAG00000015249 |  |  |
| ENSBTAG00000011541 | ENSBTAG00000015251 |  |  |
| ENSBTAG00000011543 | ENSBTAG00000015253 |  |  |
| ENSBTAG00000011544 | ENSBTAG00000015255 |  |  |
| ENSBTAG00000011545 | ENSBTAG00000015268 |  |  |
| ENSBTAG00000011551 | ENSBTAG00000015272 |  |  |
| ENSBTAG00000011553 | ENSBTAG00000015276 |  |  |
| ENSBTAG00000011556 | ENSBTAG00000015278 |  |  |
| ENSBTAG00000011563 | ENSBTAG00000015286 |  |  |
| ENSBTAG00000011571 | ENSBTAG00000015290 |  |  |
| ENSBTAG00000011582 | ENSBTAG00000015294 |  |  |
| ENSBTAG00000011583 | ENSBTAG00000015297 |  |  |
| ENSBTAG00000011584 | ENSBTAG00000015301 |  |  |
| ENSBTAG00000011585 | ENSBTAG00000015308 |  |  |
| ENSBTAG00000011586 | ENSBTAG00000015309 |  |  |
| ENSBTAG00000011587 | ENSBTAG00000015311 |  |  |
| ENSBTAG00000011588 | ENSBTAG00000015312 |  |  |
| ENSBTAG00000011591 | ENSBTAG00000015316 |  |  |
| ENSBTAG00000011593 | ENSBTAG00000015321 |  |  |
| ENSBTAG00000011596 | ENSBTAG00000015326 |  |  |
| ENSBTAG00000011598 | ENSBTAG00000015327 |  |  |
| ENSBTAG00000011602 | ENSBTAG00000015334 |  |  |
| ENSBTAG00000011608 | ENSBTAG00000015335 |  |  |
| ENSBTAG00000011611 | ENSBTAG00000015336 |  |  |
| ENSBTAG00000011613 | ENSBTAG00000015337 |  |  |
| ENSBTAG00000011616 | ENSBTAG00000015338 |  |  |
| ENSBTAG00000011617 | ENSBTAG00000015339 |  |  |
| ENSBTAG00000011620 | ENSBTAG00000015340 |  |  |
| ENSBTAG00000011623 | ENSBTAG00000015348 |  |  |
| ENSBTAG00000011626 | ENSBTAG00000015350 |  |  |
| ENSBTAG00000011628 | ENSBTAG00000015351 |  |  |
| ENSBTAG00000011632 | ENSBTAG00000015352 |  |  |
| ENSBTAG00000011634 | ENSBTAG00000015354 |  |  |
| ENSBTAG00000011635 | ENSBTAG00000015356 |  |  |
| ENSBTAG00000011639 | ENSBTAG00000015358 |  |  |
| ENSBTAG00000011642 | ENSBTAG00000015363 |  |  |
| ENSBTAG00000011644 | ENSBTAG00000015366 |  |  |
| ENSBTAG00000011647 | ENSBTAG00000015369 |  |  |
| ENSBTAG00000011649 | ENSBTAG00000015375 |  |  |
| ENSBTAG00000011658 | ENSBTAG00000015377 |  |  |
| ENSBTAG00000011660 | ENSBTAG00000015379 |  |  |
| ENSBTAG00000011666 | ENSBTAG00000015381 |  |  |
| ENSBTAG00000011689 | ENSBTAG00000015384 |  |  |
| ENSBTAG00000011692 | ENSBTAG00000015387 |  |  |
| ENSBTAG00000011694 | ENSBTAG00000015390 |  |  |
| ENSBTAG00000011698 | ENSBTAG00000015394 |  |  |
| ENSBTAG00000011700 | ENSBTAG00000015401 |  |  |
| ENSBTAG00000011709 | ENSBTAG00000015406 |  |  |
| ENSBTAG00000011713 | ENSBTAG00000015413 |  |  |
| ENSBTAG00000011715 | ENSBTAG00000015424 |  |  |
| ENSBTAG00000011717 | ENSBTAG00000015428 |  |  |
| ENSBTAG00000011726 | ENSBTAG00000015432 |  |  |
| ENSBTAG00000011727 | ENSBTAG00000015436 |  |  |
| ENSBTAG00000011729 | ENSBTAG00000015437 |  |  |
| ENSBTAG00000011730 | ENSBTAG00000015438 |  |  |
| ENSBTAG00000011732 | ENSBTAG00000015450 |  |  |
| ENSBTAG00000011733 | ENSBTAG00000015457 |  |  |
| ENSBTAG00000011736 | ENSBTAG00000015459 |  |  |
| ENSBTAG00000011738 | ENSBTAG00000015460 |  |  |
| ENSBTAG00000011740 | ENSBTAG00000015466 |  |  |
| ENSBTAG00000011741 | ENSBTAG00000015478 |  |  |
| ENSBTAG00000011752 | ENSBTAG00000015482 |  |  |
| ENSBTAG00000011757 | ENSBTAG00000015487 |  |  |
| ENSBTAG00000011760 | ENSBTAG00000015497 |  |  |
| ENSBTAG00000011762 | ENSBTAG00000015498 |  |  |
| ENSBTAG00000011763 | ENSBTAG00000015513 |  |  |
| ENSBTAG00000011765 | ENSBTAG00000015520 |  |  |
| ENSBTAG00000011766 | ENSBTAG00000015527 |  |  |
| ENSBTAG00000011767 | ENSBTAG00000015529 |  |  |
| ENSBTAG00000011770 | ENSBTAG00000015532 |  |  |
| ENSBTAG00000011779 | ENSBTAG00000015534 |  |  |
| ENSBTAG00000011783 | ENSBTAG00000015536 |  |  |
| ENSBTAG00000011785 | ENSBTAG00000015538 |  |  |
| ENSBTAG00000011787 | ENSBTAG00000015543 |  |  |
| ENSBTAG00000011788 | ENSBTAG00000015546 |  |  |
| ENSBTAG00000011789 | ENSBTAG00000015549 |  |  |
| ENSBTAG00000011793 | ENSBTAG00000015559 |  |  |
| ENSBTAG00000011795 | ENSBTAG00000015567 |  |  |
| ENSBTAG00000011796 | ENSBTAG00000015572 |  |  |
| ENSBTAG00000011798 | ENSBTAG00000015580 |  |  |
| ENSBTAG00000011800 | ENSBTAG00000015581 |  |  |
| ENSBTAG00000011802 | ENSBTAG00000015582 |  |  |
| ENSBTAG00000011805 | ENSBTAG00000015590 |  |  |
| ENSBTAG00000011808 | ENSBTAG00000015591 |  |  |
| ENSBTAG00000011809 | ENSBTAG00000015596 |  |  |
| ENSBTAG00000011810 | ENSBTAG00000015602 |  |  |
| ENSBTAG00000011812 | ENSBTAG00000015604 |  |  |
| ENSBTAG00000011813 | ENSBTAG00000015607 |  |  |
| ENSBTAG00000011815 | ENSBTAG00000015611 |  |  |
| ENSBTAG00000011818 | ENSBTAG00000015612 |  |  |
| ENSBTAG00000011822 | ENSBTAG00000015614 |  |  |
| ENSBTAG00000011823 | ENSBTAG00000015615 |  |  |
| ENSBTAG00000011824 | ENSBTAG00000015625 |  |  |
| ENSBTAG00000011825 | ENSBTAG00000015637 |  |  |
| ENSBTAG00000011826 | ENSBTAG00000015644 |  |  |
| ENSBTAG00000011831 | ENSBTAG00000015648 |  |  |
| ENSBTAG00000011832 | ENSBTAG00000015656 |  |  |
| ENSBTAG00000011837 | ENSBTAG00000015659 |  |  |
| ENSBTAG00000011839 | ENSBTAG00000015669 |  |  |
| ENSBTAG00000011843 | ENSBTAG00000015681 |  |  |
| ENSBTAG00000011847 | ENSBTAG00000015683 |  |  |
| ENSBTAG00000011851 | ENSBTAG00000015686 |  |  |
| ENSBTAG00000011854 | ENSBTAG00000015694 |  |  |
| ENSBTAG00000011860 | ENSBTAG00000015700 |  |  |
| ENSBTAG00000011861 | ENSBTAG00000015708 |  |  |
| ENSBTAG00000011865 | ENSBTAG00000015713 |  |  |
| ENSBTAG00000011866 | ENSBTAG00000015716 |  |  |
| ENSBTAG00000011869 | ENSBTAG00000015717 |  |  |
| ENSBTAG00000011872 | ENSBTAG00000015718 |  |  |
| ENSBTAG00000011876 | ENSBTAG00000015719 |  |  |
| ENSBTAG00000011879 | ENSBTAG00000015723 |  |  |
| ENSBTAG00000011880 | ENSBTAG00000015724 |  |  |
| ENSBTAG00000011881 | ENSBTAG00000015727 |  |  |
| ENSBTAG00000011885 | ENSBTAG00000015728 |  |  |
| ENSBTAG00000011887 | ENSBTAG00000015731 |  |  |
| ENSBTAG00000011892 | ENSBTAG00000015732 |  |  |
| ENSBTAG00000011893 | ENSBTAG00000015735 |  |  |
| ENSBTAG00000011894 | ENSBTAG00000015738 |  |  |
| ENSBTAG00000011895 | ENSBTAG00000015742 |  |  |
| ENSBTAG00000011898 | ENSBTAG00000015744 |  |  |
| ENSBTAG00000011902 | ENSBTAG00000015745 |  |  |
| ENSBTAG00000011905 | ENSBTAG00000015751 |  |  |
| ENSBTAG00000011909 | ENSBTAG00000015757 |  |  |
| ENSBTAG00000011910 | ENSBTAG00000015758 |  |  |
| ENSBTAG00000011911 | ENSBTAG00000015763 |  |  |
| ENSBTAG00000011912 | ENSBTAG00000015764 |  |  |
| ENSBTAG00000011916 | ENSBTAG00000015767 |  |  |
| ENSBTAG00000011917 | ENSBTAG00000015769 |  |  |
| ENSBTAG00000011937 | ENSBTAG00000015778 |  |  |
| ENSBTAG00000011939 | ENSBTAG00000015780 |  |  |
| ENSBTAG00000011944 | ENSBTAG00000015781 |  |  |
| ENSBTAG00000011946 | ENSBTAG00000015786 |  |  |
| ENSBTAG00000011951 | ENSBTAG00000015788 |  |  |
| ENSBTAG00000011952 | ENSBTAG00000015796 |  |  |
| ENSBTAG00000011953 | ENSBTAG00000015801 |  |  |
| ENSBTAG00000011957 | ENSBTAG00000015802 |  |  |
| ENSBTAG00000011960 | ENSBTAG00000015804 |  |  |
| ENSBTAG00000011964 | ENSBTAG00000015806 |  |  |
| ENSBTAG00000011966 | ENSBTAG00000015817 |  |  |
| ENSBTAG00000011970 | ENSBTAG00000015818 |  |  |
| ENSBTAG00000011982 | ENSBTAG00000015821 |  |  |
| ENSBTAG00000011986 | ENSBTAG00000015834 |  |  |
| ENSBTAG00000011990 | ENSBTAG00000015837 |  |  |
| ENSBTAG00000011991 | ENSBTAG00000015839 |  |  |
| ENSBTAG00000011994 | ENSBTAG00000015841 |  |  |
| ENSBTAG00000011997 | ENSBTAG00000015853 |  |  |
| ENSBTAG00000012004 | ENSBTAG00000015856 |  |  |
| ENSBTAG00000012007 | ENSBTAG00000015857 |  |  |
| ENSBTAG00000012012 | ENSBTAG00000015858 |  |  |
| ENSBTAG00000012014 | ENSBTAG00000015860 |  |  |
| ENSBTAG00000012020 | ENSBTAG00000015868 |  |  |
| ENSBTAG00000012030 | ENSBTAG00000015874 |  |  |
| ENSBTAG00000012034 | ENSBTAG00000015878 |  |  |
| ENSBTAG00000012036 | ENSBTAG00000015879 |  |  |
| ENSBTAG00000012039 | ENSBTAG00000015885 |  |  |
| ENSBTAG00000012046 | ENSBTAG00000015888 |  |  |
| ENSBTAG00000012048 | ENSBTAG00000015889 |  |  |
| ENSBTAG00000012049 | ENSBTAG00000015898 |  |  |
| ENSBTAG00000012050 | ENSBTAG00000015900 |  |  |
| ENSBTAG00000012057 | ENSBTAG00000015901 |  |  |
| ENSBTAG00000012058 | ENSBTAG00000015902 |  |  |
| ENSBTAG00000012059 | ENSBTAG00000015909 |  |  |
| ENSBTAG00000012063 | ENSBTAG00000015910 |  |  |
| ENSBTAG00000012066 | ENSBTAG00000015920 |  |  |
| ENSBTAG00000012069 | ENSBTAG00000015924 |  |  |
| ENSBTAG00000012071 | ENSBTAG00000015925 |  |  |
| ENSBTAG00000012073 | ENSBTAG00000015930 |  |  |
| ENSBTAG00000012074 | ENSBTAG00000015935 |  |  |
| ENSBTAG00000012077 | ENSBTAG00000015936 |  |  |
| ENSBTAG00000012078 | ENSBTAG00000015943 |  |  |
| ENSBTAG00000012081 | ENSBTAG00000015945 |  |  |
| ENSBTAG00000012086 | ENSBTAG00000015946 |  |  |
| ENSBTAG00000012094 | ENSBTAG00000015951 |  |  |
| ENSBTAG00000012095 | ENSBTAG00000015958 |  |  |
| ENSBTAG00000012096 | ENSBTAG00000015961 |  |  |
| ENSBTAG00000012100 | ENSBTAG00000015965 |  |  |
| ENSBTAG00000012103 | ENSBTAG00000015972 |  |  |
| ENSBTAG00000012104 | ENSBTAG00000015973 |  |  |
| ENSBTAG00000012106 | ENSBTAG00000015979 |  |  |
| ENSBTAG00000012112 | ENSBTAG00000015981 |  |  |
| ENSBTAG00000012117 | ENSBTAG00000015982 |  |  |
| ENSBTAG00000012119 | ENSBTAG00000015986 |  |  |
| ENSBTAG00000012121 | ENSBTAG00000015991 |  |  |
| ENSBTAG00000012124 | ENSBTAG00000015994 |  |  |
| ENSBTAG00000012125 | ENSBTAG00000015996 |  |  |
| ENSBTAG00000012126 | ENSBTAG00000016000 |  |  |
| ENSBTAG00000012139 | ENSBTAG00000016004 |  |  |
| ENSBTAG00000012140 | ENSBTAG00000016007 |  |  |
| ENSBTAG00000012141 | ENSBTAG00000016010 |  |  |
| ENSBTAG00000012142 | ENSBTAG00000016026 |  |  |
| ENSBTAG00000012144 | ENSBTAG00000016028 |  |  |
| ENSBTAG00000012147 | ENSBTAG00000016030 |  |  |
| ENSBTAG00000012152 | ENSBTAG00000016037 |  |  |
| ENSBTAG00000012156 | ENSBTAG00000016043 |  |  |
| ENSBTAG00000012159 | ENSBTAG00000016045 |  |  |
| ENSBTAG00000012163 | ENSBTAG00000016060 |  |  |
| ENSBTAG00000012170 | ENSBTAG00000016061 |  |  |
| ENSBTAG00000012176 | ENSBTAG00000016063 |  |  |
| ENSBTAG00000012177 | ENSBTAG00000016077 |  |  |
| ENSBTAG00000012182 | ENSBTAG00000016085 |  |  |
| ENSBTAG00000012189 | ENSBTAG00000016092 |  |  |
| ENSBTAG00000012194 | ENSBTAG00000016094 |  |  |
| ENSBTAG00000012197 | ENSBTAG00000016096 |  |  |
| ENSBTAG00000012199 | ENSBTAG00000016098 |  |  |
| ENSBTAG00000012211 | ENSBTAG00000016107 |  |  |
| ENSBTAG00000012213 | ENSBTAG00000016109 |  |  |
| ENSBTAG00000012215 | ENSBTAG00000016121 |  |  |
| ENSBTAG00000012217 | ENSBTAG00000016124 |  |  |
| ENSBTAG00000012219 | ENSBTAG00000016128 |  |  |
| ENSBTAG00000012222 | ENSBTAG00000016137 |  |  |
| ENSBTAG00000012223 | ENSBTAG00000016147 |  |  |
| ENSBTAG00000012228 | ENSBTAG00000016149 |  |  |
| ENSBTAG00000012229 | ENSBTAG00000016152 |  |  |
| ENSBTAG00000012232 | ENSBTAG00000016156 |  |  |
| ENSBTAG00000012235 | ENSBTAG00000016165 |  |  |
| ENSBTAG00000012237 | ENSBTAG00000016168 |  |  |
| ENSBTAG00000012239 | ENSBTAG00000016169 |  |  |
| ENSBTAG00000012242 | ENSBTAG00000016191 |  |  |
| ENSBTAG00000012247 | ENSBTAG00000016199 |  |  |
| ENSBTAG00000012249 | ENSBTAG00000016206 |  |  |
| ENSBTAG00000012250 | ENSBTAG00000016208 |  |  |
| ENSBTAG00000012253 | ENSBTAG00000016209 |  |  |
| ENSBTAG00000012259 | ENSBTAG00000016217 |  |  |
| ENSBTAG00000012261 | ENSBTAG00000016218 |  |  |
| ENSBTAG00000012262 | ENSBTAG00000016220 |  |  |
| ENSBTAG00000012267 | ENSBTAG00000016223 |  |  |
| ENSBTAG00000012271 | ENSBTAG00000016225 |  |  |
| ENSBTAG00000012272 | ENSBTAG00000016228 |  |  |
| ENSBTAG00000012273 | ENSBTAG00000016231 |  |  |
| ENSBTAG00000012275 | ENSBTAG00000016235 |  |  |
| ENSBTAG00000012280 | ENSBTAG00000016240 |  |  |
| ENSBTAG00000012284 | ENSBTAG00000016242 |  |  |
| ENSBTAG00000012285 | ENSBTAG00000016243 |  |  |
| ENSBTAG00000012289 | ENSBTAG00000016252 |  |  |
| ENSBTAG00000012291 | ENSBTAG00000016253 |  |  |
| ENSBTAG00000012293 | ENSBTAG00000016254 |  |  |
| ENSBTAG00000012295 | ENSBTAG00000016255 |  |  |
| ENSBTAG00000012305 | ENSBTAG00000016260 |  |  |
| ENSBTAG00000012307 | ENSBTAG00000016263 |  |  |
| ENSBTAG00000012314 | ENSBTAG00000016264 |  |  |
| ENSBTAG00000012317 | ENSBTAG00000016265 |  |  |
| ENSBTAG00000012319 | ENSBTAG00000016271 |  |  |
| ENSBTAG00000012320 | ENSBTAG00000016275 |  |  |
| ENSBTAG00000012328 | ENSBTAG00000016276 |  |  |
| ENSBTAG00000012332 | ENSBTAG00000016277 |  |  |
| ENSBTAG00000012334 | ENSBTAG00000016282 |  |  |
| ENSBTAG00000012342 | ENSBTAG00000016287 |  |  |
| ENSBTAG00000012343 | ENSBTAG00000016293 |  |  |
| ENSBTAG00000012347 | ENSBTAG00000016294 |  |  |
| ENSBTAG00000012348 | ENSBTAG00000016295 |  |  |
| ENSBTAG00000012349 | ENSBTAG00000016296 |  |  |
| ENSBTAG00000012350 | ENSBTAG00000016298 |  |  |
| ENSBTAG00000012352 | ENSBTAG00000016302 |  |  |
| ENSBTAG00000012353 | ENSBTAG00000016305 |  |  |
| ENSBTAG00000012355 | ENSBTAG00000016307 |  |  |
| ENSBTAG00000012361 | ENSBTAG00000016309 |  |  |
| ENSBTAG00000012363 | ENSBTAG00000016311 |  |  |
| ENSBTAG00000012365 | ENSBTAG00000016315 |  |  |
| ENSBTAG00000012366 | ENSBTAG00000016320 |  |  |
| ENSBTAG00000012374 | ENSBTAG00000016323 |  |  |
| ENSBTAG00000012375 | ENSBTAG00000016327 |  |  |
| ENSBTAG00000012380 | ENSBTAG00000016328 |  |  |
| ENSBTAG00000012382 | ENSBTAG00000016336 |  |  |
| ENSBTAG00000012383 | ENSBTAG00000016337 |  |  |
| ENSBTAG00000012384 | ENSBTAG00000016341 |  |  |
| ENSBTAG00000012385 | ENSBTAG00000016343 |  |  |
| ENSBTAG00000012387 | ENSBTAG00000016344 |  |  |
| ENSBTAG00000012390 | ENSBTAG00000016349 |  |  |
| ENSBTAG00000012394 | ENSBTAG00000016350 |  |  |
| ENSBTAG00000012397 | ENSBTAG00000016352 |  |  |
| ENSBTAG00000012403 | ENSBTAG00000016354 |  |  |
| ENSBTAG00000012405 | ENSBTAG00000016360 |  |  |
| ENSBTAG00000012406 | ENSBTAG00000016363 |  |  |
| ENSBTAG00000012409 | ENSBTAG00000016367 |  |  |
| ENSBTAG00000012411 | ENSBTAG00000016369 |  |  |
| ENSBTAG00000012412 | ENSBTAG00000016377 |  |  |
| ENSBTAG00000012416 | ENSBTAG00000016378 |  |  |
| ENSBTAG00000012417 | ENSBTAG00000016384 |  |  |
| ENSBTAG00000012426 | ENSBTAG00000016387 |  |  |
| ENSBTAG00000012432 | ENSBTAG00000016389 |  |  |
| ENSBTAG00000012433 | ENSBTAG00000016396 |  |  |
| ENSBTAG00000012439 | ENSBTAG00000016397 |  |  |
| ENSBTAG00000012442 | ENSBTAG00000016398 |  |  |
| ENSBTAG00000012443 | ENSBTAG00000016399 |  |  |
| ENSBTAG00000012444 | ENSBTAG00000016407 |  |  |
| ENSBTAG00000012446 | ENSBTAG00000016412 |  |  |
| ENSBTAG00000012447 | ENSBTAG00000016413 |  |  |
| ENSBTAG00000012448 | ENSBTAG00000016415 |  |  |
| ENSBTAG00000012450 | ENSBTAG00000016427 |  |  |
| ENSBTAG00000012451 | ENSBTAG00000016442 |  |  |
| ENSBTAG00000012454 | ENSBTAG00000016445 |  |  |
| ENSBTAG00000012456 | ENSBTAG00000016450 |  |  |
| ENSBTAG00000012458 | ENSBTAG00000016456 |  |  |
| ENSBTAG00000012460 | ENSBTAG00000016457 |  |  |
| ENSBTAG00000012461 | ENSBTAG00000016465 |  |  |
| ENSBTAG00000012463 | ENSBTAG00000016467 |  |  |
| ENSBTAG00000012465 | ENSBTAG00000016473 |  |  |
| ENSBTAG00000012467 | ENSBTAG00000016481 |  |  |
| ENSBTAG00000012470 | ENSBTAG00000016486 |  |  |
| ENSBTAG00000012471 | ENSBTAG00000016494 |  |  |
| ENSBTAG00000012476 | ENSBTAG00000016501 |  |  |
| ENSBTAG00000012480 | ENSBTAG00000016502 |  |  |
| ENSBTAG00000012481 | ENSBTAG00000016505 |  |  |
| ENSBTAG00000012489 | ENSBTAG00000016506 |  |  |
| ENSBTAG00000012491 | ENSBTAG00000016508 |  |  |
| ENSBTAG00000012495 | ENSBTAG00000016511 |  |  |
| ENSBTAG00000012496 | ENSBTAG00000016514 |  |  |
| ENSBTAG00000012498 | ENSBTAG00000016516 |  |  |
| ENSBTAG00000012500 | ENSBTAG00000016519 |  |  |
| ENSBTAG00000012501 | ENSBTAG00000016522 |  |  |
| ENSBTAG00000012504 | ENSBTAG00000016525 |  |  |
| ENSBTAG00000012507 | ENSBTAG00000016527 |  |  |
| ENSBTAG00000012509 | ENSBTAG00000016529 |  |  |
| ENSBTAG00000012510 | ENSBTAG00000016532 |  |  |
| ENSBTAG00000012511 | ENSBTAG00000016533 |  |  |
| ENSBTAG00000012512 | ENSBTAG00000016541 |  |  |
| ENSBTAG00000012514 | ENSBTAG00000016542 |  |  |
| ENSBTAG00000012518 | ENSBTAG00000016546 |  |  |
| ENSBTAG00000012519 | ENSBTAG00000016549 |  |  |
| ENSBTAG00000012526 | ENSBTAG00000016551 |  |  |
| ENSBTAG00000012534 | ENSBTAG00000016558 |  |  |
| ENSBTAG00000012535 | ENSBTAG00000016562 |  |  |
| ENSBTAG00000012541 | ENSBTAG00000016563 |  |  |
| ENSBTAG00000012544 | ENSBTAG00000016567 |  |  |
| ENSBTAG00000012545 | ENSBTAG00000016571 |  |  |
| ENSBTAG00000012552 | ENSBTAG00000016578 |  |  |
| ENSBTAG00000012557 | ENSBTAG00000016591 |  |  |
| ENSBTAG00000012560 | ENSBTAG00000016593 |  |  |
| ENSBTAG00000012561 | ENSBTAG00000016595 |  |  |
| ENSBTAG00000012582 | ENSBTAG00000016599 |  |  |
| ENSBTAG00000012584 | ENSBTAG00000016609 |  |  |
| ENSBTAG00000012585 | ENSBTAG00000016613 |  |  |
| ENSBTAG00000012586 | ENSBTAG00000016615 |  |  |
| ENSBTAG00000012587 | ENSBTAG00000016618 |  |  |
| ENSBTAG00000012594 | ENSBTAG00000016622 |  |  |
| ENSBTAG00000012595 | ENSBTAG00000016627 |  |  |
| ENSBTAG00000012596 | ENSBTAG00000016635 |  |  |
| ENSBTAG00000012604 | ENSBTAG00000016640 |  |  |
| ENSBTAG00000012606 | ENSBTAG00000016648 |  |  |
| ENSBTAG00000012608 | ENSBTAG00000016650 |  |  |
| ENSBTAG00000012619 | ENSBTAG00000016651 |  |  |
| ENSBTAG00000012620 | ENSBTAG00000016661 |  |  |
| ENSBTAG00000012622 | ENSBTAG00000016662 |  |  |
| ENSBTAG00000012623 | ENSBTAG00000016663 |  |  |
| ENSBTAG00000012626 | ENSBTAG00000016664 |  |  |
| ENSBTAG00000012629 | ENSBTAG00000016666 |  |  |
| ENSBTAG00000012630 | ENSBTAG00000016676 |  |  |
| ENSBTAG00000012632 | ENSBTAG00000016677 |  |  |
| ENSBTAG00000012634 | ENSBTAG00000016679 |  |  |
| ENSBTAG00000012637 | ENSBTAG00000016680 |  |  |
| ENSBTAG00000012642 | ENSBTAG00000016683 |  |  |
| ENSBTAG00000012652 | ENSBTAG00000016694 |  |  |
| ENSBTAG00000012654 | ENSBTAG00000016696 |  |  |
| ENSBTAG00000012656 | ENSBTAG00000016698 |  |  |
| ENSBTAG00000012659 | ENSBTAG00000016703 |  |  |
| ENSBTAG00000012664 | ENSBTAG00000016704 |  |  |
| ENSBTAG00000012671 | ENSBTAG00000016707 |  |  |
| ENSBTAG00000012672 | ENSBTAG00000016708 |  |  |
| ENSBTAG00000012673 | ENSBTAG00000016709 |  |  |
| ENSBTAG00000012674 | ENSBTAG00000016712 |  |  |
| ENSBTAG00000012675 | ENSBTAG00000016720 |  |  |
| ENSBTAG00000012676 | ENSBTAG00000016723 |  |  |
| ENSBTAG00000012677 | ENSBTAG00000016724 |  |  |
| ENSBTAG00000012678 | ENSBTAG00000016725 |  |  |
| ENSBTAG00000012684 | ENSBTAG00000016728 |  |  |
| ENSBTAG00000012688 | ENSBTAG00000016730 |  |  |
| ENSBTAG00000012692 | ENSBTAG00000016731 |  |  |
| ENSBTAG00000012697 | ENSBTAG00000016738 |  |  |
| ENSBTAG00000012698 | ENSBTAG00000016744 |  |  |
| ENSBTAG00000012699 | ENSBTAG00000016751 |  |  |
| ENSBTAG00000012700 | ENSBTAG00000016762 |  |  |
| ENSBTAG00000012702 | ENSBTAG00000016763 |  |  |
| ENSBTAG00000012703 | ENSBTAG00000016766 |  |  |
| ENSBTAG00000012704 | ENSBTAG00000016769 |  |  |
| ENSBTAG00000012705 | ENSBTAG00000016770 |  |  |
| ENSBTAG00000012719 | ENSBTAG00000016771 |  |  |
| ENSBTAG00000012721 | ENSBTAG00000016779 |  |  |
| ENSBTAG00000012723 | ENSBTAG00000016782 |  |  |
| ENSBTAG00000012724 | ENSBTAG00000016791 |  |  |
| ENSBTAG00000012725 | ENSBTAG00000016800 |  |  |
| ENSBTAG00000012728 | ENSBTAG00000016804 |  |  |
| ENSBTAG00000012737 | ENSBTAG00000016805 |  |  |
| ENSBTAG00000012739 | ENSBTAG00000016818 |  |  |
| ENSBTAG00000012744 | ENSBTAG00000016821 |  |  |
| ENSBTAG00000012746 | ENSBTAG00000016823 |  |  |
| ENSBTAG00000012749 | ENSBTAG00000016826 |  |  |
| ENSBTAG00000012751 | ENSBTAG00000016827 |  |  |
| ENSBTAG00000012753 | ENSBTAG00000016828 |  |  |
| ENSBTAG00000012757 | ENSBTAG00000016830 |  |  |
| ENSBTAG00000012758 | ENSBTAG00000016835 |  |  |
| ENSBTAG00000012761 | ENSBTAG00000016841 |  |  |
| ENSBTAG00000012762 | ENSBTAG00000016845 |  |  |
| ENSBTAG00000012768 | ENSBTAG00000016846 |  |  |
| ENSBTAG00000012771 | ENSBTAG00000016847 |  |  |
| ENSBTAG00000012774 | ENSBTAG00000016848 |  |  |
| ENSBTAG00000012777 | ENSBTAG00000016849 |  |  |
| ENSBTAG00000012778 | ENSBTAG00000016867 |  |  |
| ENSBTAG00000012781 | ENSBTAG00000016869 |  |  |
| ENSBTAG00000012785 | ENSBTAG00000016873 |  |  |
| ENSBTAG00000012790 | ENSBTAG00000016874 |  |  |
| ENSBTAG00000012792 | ENSBTAG00000016878 |  |  |
| ENSBTAG00000012797 | ENSBTAG00000016881 |  |  |
| ENSBTAG00000012801 | ENSBTAG00000016882 |  |  |
| ENSBTAG00000012803 | ENSBTAG00000016885 |  |  |
| ENSBTAG00000012804 | ENSBTAG00000016887 |  |  |
| ENSBTAG00000012805 | ENSBTAG00000016890 |  |  |
| ENSBTAG00000012808 | ENSBTAG00000016896 |  |  |
| ENSBTAG00000012817 | ENSBTAG00000016900 |  |  |
| ENSBTAG00000012818 | ENSBTAG00000016906 |  |  |
| ENSBTAG00000012820 | ENSBTAG00000016908 |  |  |
| ENSBTAG00000012823 | ENSBTAG00000016910 |  |  |
| ENSBTAG00000012826 | ENSBTAG00000016913 |  |  |
| ENSBTAG00000012827 | ENSBTAG00000016918 |  |  |
| ENSBTAG00000012829 | ENSBTAG00000016924 |  |  |
| ENSBTAG00000012830 | ENSBTAG00000016932 |  |  |
| ENSBTAG00000012833 | ENSBTAG00000016933 |  |  |
| ENSBTAG00000012837 | ENSBTAG00000016948 |  |  |
| ENSBTAG00000012838 | ENSBTAG00000016956 |  |  |
| ENSBTAG00000012844 | ENSBTAG00000016961 |  |  |
| ENSBTAG00000012845 | ENSBTAG00000016963 |  |  |
| ENSBTAG00000012848 | ENSBTAG00000016968 |  |  |
| ENSBTAG00000012849 | ENSBTAG00000016969 |  |  |
| ENSBTAG00000012850 | ENSBTAG00000016973 |  |  |
| ENSBTAG00000012851 | ENSBTAG00000016977 |  |  |
| ENSBTAG00000012854 | ENSBTAG00000016979 |  |  |
| ENSBTAG00000012855 | ENSBTAG00000016980 |  |  |
| ENSBTAG00000012857 | ENSBTAG00000016984 |  |  |
| ENSBTAG00000012858 | ENSBTAG00000016988 |  |  |
| ENSBTAG00000012860 | ENSBTAG00000016998 |  |  |
| ENSBTAG00000012863 | ENSBTAG00000017002 |  |  |
| ENSBTAG00000012865 | ENSBTAG00000017007 |  |  |
| ENSBTAG00000012873 | ENSBTAG00000017016 |  |  |
| ENSBTAG00000012878 | ENSBTAG00000017017 |  |  |
| ENSBTAG00000012884 | ENSBTAG00000017019 |  |  |
| ENSBTAG00000012885 | ENSBTAG00000017024 |  |  |
| ENSBTAG00000012887 | ENSBTAG00000017028 |  |  |
| ENSBTAG00000012889 | ENSBTAG00000017038 |  |  |
| ENSBTAG00000012890 | ENSBTAG00000017056 |  |  |
| ENSBTAG00000012898 | ENSBTAG00000017060 |  |  |
| ENSBTAG00000012900 | ENSBTAG00000017061 |  |  |
| ENSBTAG00000012902 | ENSBTAG00000017064 |  |  |
| ENSBTAG00000012904 | ENSBTAG00000017067 |  |  |
| ENSBTAG00000012908 | ENSBTAG00000017068 |  |  |
| ENSBTAG00000012912 | ENSBTAG00000017069 |  |  |
| ENSBTAG00000012918 | ENSBTAG00000017071 |  |  |
| ENSBTAG00000012919 | ENSBTAG00000017072 |  |  |
| ENSBTAG00000012921 | ENSBTAG00000017075 |  |  |
| ENSBTAG00000012922 | ENSBTAG00000017079 |  |  |
| ENSBTAG00000012925 | ENSBTAG00000017083 |  |  |
| ENSBTAG00000012927 | ENSBTAG00000017086 |  |  |
| ENSBTAG00000012929 | ENSBTAG00000017087 |  |  |
| ENSBTAG00000012931 | ENSBTAG00000017091 |  |  |
| ENSBTAG00000012936 | ENSBTAG00000017094 |  |  |
| ENSBTAG00000012937 | ENSBTAG00000017115 |  |  |
| ENSBTAG00000012938 | ENSBTAG00000017116 |  |  |
| ENSBTAG00000012939 | ENSBTAG00000017118 |  |  |
| ENSBTAG00000012940 | ENSBTAG00000017125 |  |  |
| ENSBTAG00000012941 | ENSBTAG00000017129 |  |  |
| ENSBTAG00000012943 | ENSBTAG00000017132 |  |  |
| ENSBTAG00000012945 | ENSBTAG00000017133 |  |  |
| ENSBTAG00000012957 | ENSBTAG00000017135 |  |  |
| ENSBTAG00000012961 | ENSBTAG00000017139 |  |  |
| ENSBTAG00000012962 | ENSBTAG00000017141 |  |  |
| ENSBTAG00000012966 | ENSBTAG00000017147 |  |  |
| ENSBTAG00000012968 | ENSBTAG00000017158 |  |  |
| ENSBTAG00000012969 | ENSBTAG00000017162 |  |  |
| ENSBTAG00000012972 | ENSBTAG00000017165 |  |  |
| ENSBTAG00000012975 | ENSBTAG00000017179 |  |  |
| ENSBTAG00000012976 | ENSBTAG00000017181 |  |  |
| ENSBTAG00000012979 | ENSBTAG00000017184 |  |  |
| ENSBTAG00000012980 | ENSBTAG00000017187 |  |  |
| ENSBTAG00000012981 | ENSBTAG00000017188 |  |  |
| ENSBTAG00000012982 | ENSBTAG00000017200 |  |  |
| ENSBTAG00000012985 | ENSBTAG00000017213 |  |  |
| ENSBTAG00000012987 | ENSBTAG00000017218 |  |  |
| ENSBTAG00000012988 | ENSBTAG00000017223 |  |  |
| ENSBTAG00000012990 | ENSBTAG00000017225 |  |  |
| ENSBTAG00000012991 | ENSBTAG00000017239 |  |  |
| ENSBTAG00000012994 | ENSBTAG00000017243 |  |  |
| ENSBTAG00000012995 | ENSBTAG00000017256 |  |  |
| ENSBTAG00000012996 | ENSBTAG00000017263 |  |  |
| ENSBTAG00000012997 | ENSBTAG00000017265 |  |  |
| ENSBTAG00000013004 | ENSBTAG00000017266 |  |  |
| ENSBTAG00000013007 | ENSBTAG00000017267 |  |  |
| ENSBTAG00000013009 | ENSBTAG00000017275 |  |  |
| ENSBTAG00000013010 | ENSBTAG00000017279 |  |  |
| ENSBTAG00000013011 | ENSBTAG00000017283 |  |  |
| ENSBTAG00000013014 | ENSBTAG00000017284 |  |  |
| ENSBTAG00000013016 | ENSBTAG00000017287 |  |  |
| ENSBTAG00000013017 | ENSBTAG00000017310 |  |  |
| ENSBTAG00000013018 | ENSBTAG00000017313 |  |  |
| ENSBTAG00000013030 | ENSBTAG00000017318 |  |  |
| ENSBTAG00000013031 | ENSBTAG00000017321 |  |  |
| ENSBTAG00000013032 | ENSBTAG00000017325 |  |  |
| ENSBTAG00000013033 | ENSBTAG00000017326 |  |  |
| ENSBTAG00000013035 | ENSBTAG00000017329 |  |  |
| ENSBTAG00000013038 | ENSBTAG00000017339 |  |  |
| ENSBTAG00000013039 | ENSBTAG00000017346 |  |  |
| ENSBTAG00000013042 | ENSBTAG00000017349 |  |  |
| ENSBTAG00000013043 | ENSBTAG00000017354 |  |  |
| ENSBTAG00000013044 | ENSBTAG00000017361 |  |  |
| ENSBTAG00000013045 | ENSBTAG00000017362 |  |  |
| ENSBTAG00000013047 | ENSBTAG00000017363 |  |  |
| ENSBTAG00000013050 | ENSBTAG00000017366 |  |  |
| ENSBTAG00000013060 | ENSBTAG00000017367 |  |  |
| ENSBTAG00000013063 | ENSBTAG00000017368 |  |  |
| ENSBTAG00000013066 | ENSBTAG00000017369 |  |  |
| ENSBTAG00000013068 | ENSBTAG00000017375 |  |  |
| ENSBTAG00000013069 | ENSBTAG00000017382 |  |  |
| ENSBTAG00000013070 | ENSBTAG00000017393 |  |  |
| ENSBTAG00000013072 | ENSBTAG00000017395 |  |  |
| ENSBTAG00000013074 | ENSBTAG00000017397 |  |  |
| ENSBTAG00000013076 | ENSBTAG00000017401 |  |  |
| ENSBTAG00000013078 | ENSBTAG00000017402 |  |  |
| ENSBTAG00000013079 | ENSBTAG00000017407 |  |  |
| ENSBTAG00000013081 | ENSBTAG00000017409 |  |  |
| ENSBTAG00000013084 | ENSBTAG00000017411 |  |  |
| ENSBTAG00000013093 | ENSBTAG00000017420 |  |  |
| ENSBTAG00000013095 | ENSBTAG00000017421 |  |  |
| ENSBTAG00000013096 | ENSBTAG00000017423 |  |  |
| ENSBTAG00000013098 | ENSBTAG00000017424 |  |  |
| ENSBTAG00000013099 | ENSBTAG00000017425 |  |  |
| ENSBTAG00000013100 | ENSBTAG00000017426 |  |  |
| ENSBTAG00000013105 | ENSBTAG00000017435 |  |  |
| ENSBTAG00000013108 | ENSBTAG00000017436 |  |  |
| ENSBTAG00000013109 | ENSBTAG00000017437 |  |  |
| ENSBTAG00000013111 | ENSBTAG00000017439 |  |  |
| ENSBTAG00000013112 | ENSBTAG00000017443 |  |  |
| ENSBTAG00000013113 | ENSBTAG00000017452 |  |  |
| ENSBTAG00000013114 | ENSBTAG00000017458 |  |  |
| ENSBTAG00000013118 | ENSBTAG00000017459 |  |  |
| ENSBTAG00000013125 | ENSBTAG00000017461 |  |  |
| ENSBTAG00000013126 | ENSBTAG00000017465 |  |  |
| ENSBTAG00000013127 | ENSBTAG00000017478 |  |  |
| ENSBTAG00000013128 | ENSBTAG00000017482 |  |  |
| ENSBTAG00000013131 | ENSBTAG00000017490 |  |  |
| ENSBTAG00000013133 | ENSBTAG00000017492 |  |  |
| ENSBTAG00000013142 | ENSBTAG00000017508 |  |  |
| ENSBTAG00000013143 | ENSBTAG00000017512 |  |  |
| ENSBTAG00000013145 | ENSBTAG00000017517 |  |  |
| ENSBTAG00000013152 | ENSBTAG00000017524 |  |  |
| ENSBTAG00000013155 | ENSBTAG00000017525 |  |  |
| ENSBTAG00000013159 | ENSBTAG00000017527 |  |  |
| ENSBTAG00000013166 | ENSBTAG00000017537 |  |  |
| ENSBTAG00000013167 | ENSBTAG00000017540 |  |  |
| ENSBTAG00000013168 | ENSBTAG00000017545 |  |  |
| ENSBTAG00000013169 | ENSBTAG00000017547 |  |  |
| ENSBTAG00000013173 | ENSBTAG00000017549 |  |  |
| ENSBTAG00000013174 | ENSBTAG00000017550 |  |  |
| ENSBTAG00000013177 | ENSBTAG00000017557 |  |  |
| ENSBTAG00000013178 | ENSBTAG00000017560 |  |  |
| ENSBTAG00000013183 | ENSBTAG00000017566 |  |  |
| ENSBTAG00000013184 | ENSBTAG00000017569 |  |  |
| ENSBTAG00000013185 | ENSBTAG00000017582 |  |  |
| ENSBTAG00000013187 | ENSBTAG00000017592 |  |  |
| ENSBTAG00000013191 | ENSBTAG00000017593 |  |  |
| ENSBTAG00000013192 | ENSBTAG00000017602 |  |  |
| ENSBTAG00000013197 | ENSBTAG00000017604 |  |  |
| ENSBTAG00000013201 | ENSBTAG00000017605 |  |  |
| ENSBTAG00000013204 | ENSBTAG00000017613 |  |  |
| ENSBTAG00000013205 | ENSBTAG00000017616 |  |  |
| ENSBTAG00000013211 | ENSBTAG00000017622 |  |  |
| ENSBTAG00000013213 | ENSBTAG00000017636 |  |  |
| ENSBTAG00000013215 | ENSBTAG00000017639 |  |  |
| ENSBTAG00000013218 | ENSBTAG00000017641 |  |  |
| ENSBTAG00000013224 | ENSBTAG00000017647 |  |  |
| ENSBTAG00000013226 | ENSBTAG00000017652 |  |  |
| ENSBTAG00000013227 | ENSBTAG00000017655 |  |  |
| ENSBTAG00000013231 | ENSBTAG00000017656 |  |  |
| ENSBTAG00000013235 | ENSBTAG00000017660 |  |  |
| ENSBTAG00000013240 | ENSBTAG00000017661 |  |  |
| ENSBTAG00000013242 | ENSBTAG00000017676 |  |  |
| ENSBTAG00000013244 | ENSBTAG00000017680 |  |  |
| ENSBTAG00000013245 | ENSBTAG00000017681 |  |  |
| ENSBTAG00000013249 | ENSBTAG00000017683 |  |  |
| ENSBTAG00000013259 | ENSBTAG00000017689 |  |  |
| ENSBTAG00000013270 | ENSBTAG00000017690 |  |  |
| ENSBTAG00000013271 | ENSBTAG00000017694 |  |  |
| ENSBTAG00000013273 | ENSBTAG00000017713 |  |  |
| ENSBTAG00000013277 | ENSBTAG00000017718 |  |  |
| ENSBTAG00000013279 | ENSBTAG00000017719 |  |  |
| ENSBTAG00000013282 | ENSBTAG00000017729 |  |  |
| ENSBTAG00000013284 | ENSBTAG00000017738 |  |  |
| ENSBTAG00000013287 | ENSBTAG00000017739 |  |  |
| ENSBTAG00000013290 | ENSBTAG00000017740 |  |  |
| ENSBTAG00000013291 | ENSBTAG00000017747 |  |  |
| ENSBTAG00000013292 | ENSBTAG00000017748 |  |  |
| ENSBTAG00000013294 | ENSBTAG00000017767 |  |  |
| ENSBTAG00000013298 | ENSBTAG00000017776 |  |  |
| ENSBTAG00000013301 | ENSBTAG00000017779 |  |  |
| ENSBTAG00000013303 | ENSBTAG00000017780 |  |  |
| ENSBTAG00000013308 | ENSBTAG00000017781 |  |  |
| ENSBTAG00000013309 | ENSBTAG00000017797 |  |  |
| ENSBTAG00000013314 | ENSBTAG00000017799 |  |  |
| ENSBTAG00000013316 | ENSBTAG00000017801 |  |  |
| ENSBTAG00000013317 | ENSBTAG00000017804 |  |  |
| ENSBTAG00000013319 | ENSBTAG00000017805 |  |  |
| ENSBTAG00000013320 | ENSBTAG00000017809 |  |  |
| ENSBTAG00000013322 | ENSBTAG00000017810 |  |  |
| ENSBTAG00000013329 | ENSBTAG00000017811 |  |  |
| ENSBTAG00000013330 | ENSBTAG00000017812 |  |  |
| ENSBTAG00000013333 | ENSBTAG00000017814 |  |  |
| ENSBTAG00000013334 | ENSBTAG00000017824 |  |  |
| ENSBTAG00000013336 | ENSBTAG00000017825 |  |  |
| ENSBTAG00000013337 | ENSBTAG00000017831 |  |  |
| ENSBTAG00000013341 | ENSBTAG00000017833 |  |  |
| ENSBTAG00000013346 | ENSBTAG00000017834 |  |  |
| ENSBTAG00000013353 | ENSBTAG00000017835 |  |  |
| ENSBTAG00000013362 | ENSBTAG00000017839 |  |  |
| ENSBTAG00000013363 | ENSBTAG00000017840 |  |  |
| ENSBTAG00000013366 | ENSBTAG00000017846 |  |  |
| ENSBTAG00000013367 | ENSBTAG00000017847 |  |  |
| ENSBTAG00000013368 | ENSBTAG00000017850 |  |  |
| ENSBTAG00000013371 | ENSBTAG00000017852 |  |  |
| ENSBTAG00000013387 | ENSBTAG00000017855 |  |  |
| ENSBTAG00000013391 | ENSBTAG00000017856 |  |  |
| ENSBTAG00000013400 | ENSBTAG00000017860 |  |  |
| ENSBTAG00000013401 | ENSBTAG00000017863 |  |  |
| ENSBTAG00000013402 | ENSBTAG00000017864 |  |  |
| ENSBTAG00000013405 | ENSBTAG00000017866 |  |  |
| ENSBTAG00000013406 | ENSBTAG00000017867 |  |  |
| ENSBTAG00000013407 | ENSBTAG00000017868 |  |  |
| ENSBTAG00000013411 | ENSBTAG00000017869 |  |  |
| ENSBTAG00000013414 | ENSBTAG00000017872 |  |  |
| ENSBTAG00000013416 | ENSBTAG00000017873 |  |  |
| ENSBTAG00000013419 | ENSBTAG00000017875 |  |  |
| ENSBTAG00000013422 | ENSBTAG00000017877 |  |  |
| ENSBTAG00000013425 | ENSBTAG00000017889 |  |  |
| ENSBTAG00000013426 | ENSBTAG00000017919 |  |  |
| ENSBTAG00000013436 | ENSBTAG00000017922 |  |  |
| ENSBTAG00000013439 | ENSBTAG00000017932 |  |  |
| ENSBTAG00000013442 | ENSBTAG00000017942 |  |  |
| ENSBTAG00000013443 | ENSBTAG00000017943 |  |  |
| ENSBTAG00000013449 | ENSBTAG00000017956 |  |  |
| ENSBTAG00000013454 | ENSBTAG00000017958 |  |  |
| ENSBTAG00000013456 | ENSBTAG00000017967 |  |  |
| ENSBTAG00000013462 | ENSBTAG00000017970 |  |  |
| ENSBTAG00000013464 | ENSBTAG00000017992 |  |  |
| ENSBTAG00000013472 | ENSBTAG00000017996 |  |  |
| ENSBTAG00000013473 | ENSBTAG00000017999 |  |  |
| ENSBTAG00000013478 | ENSBTAG00000018000 |  |  |
| ENSBTAG00000013479 | ENSBTAG00000018011 |  |  |
| ENSBTAG00000013481 | ENSBTAG00000018015 |  |  |
| ENSBTAG00000013483 | ENSBTAG00000018016 |  |  |
| ENSBTAG00000013488 | ENSBTAG00000018020 |  |  |
| ENSBTAG00000013492 | ENSBTAG00000018025 |  |  |
| ENSBTAG00000013495 | ENSBTAG00000018026 |  |  |
| ENSBTAG00000013496 | ENSBTAG00000018033 |  |  |
| ENSBTAG00000013498 | ENSBTAG00000018036 |  |  |
| ENSBTAG00000013499 | ENSBTAG00000018044 |  |  |
| ENSBTAG00000013501 | ENSBTAG00000018046 |  |  |
| ENSBTAG00000013502 | ENSBTAG00000018048 |  |  |
| ENSBTAG00000013507 | ENSBTAG00000018049 |  |  |
| ENSBTAG00000013510 | ENSBTAG00000018050 |  |  |
| ENSBTAG00000013513 | ENSBTAG00000018053 |  |  |
| ENSBTAG00000013515 | ENSBTAG00000018063 |  |  |
| ENSBTAG00000013523 | ENSBTAG00000018065 |  |  |
| ENSBTAG00000013525 | ENSBTAG00000018067 |  |  |
| ENSBTAG00000013526 | ENSBTAG00000018071 |  |  |
| ENSBTAG00000013527 | ENSBTAG00000018072 |  |  |
| ENSBTAG00000013528 | ENSBTAG00000018085 |  |  |
| ENSBTAG00000013531 | ENSBTAG00000018094 |  |  |
| ENSBTAG00000013533 | ENSBTAG00000018097 |  |  |
| ENSBTAG00000013534 | ENSBTAG00000018101 |  |  |
| ENSBTAG00000013535 | ENSBTAG00000018114 |  |  |
| ENSBTAG00000013538 | ENSBTAG00000018115 |  |  |
| ENSBTAG00000013541 | ENSBTAG00000018116 |  |  |
| ENSBTAG00000013544 | ENSBTAG00000018119 |  |  |
| ENSBTAG00000013545 | ENSBTAG00000018123 |  |  |
| ENSBTAG00000013550 | ENSBTAG00000018126 |  |  |
| ENSBTAG00000013555 | ENSBTAG00000018131 |  |  |
| ENSBTAG00000013556 | ENSBTAG00000018134 |  |  |
| ENSBTAG00000013557 | ENSBTAG00000018146 |  |  |
| ENSBTAG00000013569 | ENSBTAG00000018152 |  |  |
| ENSBTAG00000013573 | ENSBTAG00000018153 |  |  |
| ENSBTAG00000013577 | ENSBTAG00000018156 |  |  |
| ENSBTAG00000013579 | ENSBTAG00000018158 |  |  |
| ENSBTAG00000013580 | ENSBTAG00000018159 |  |  |
| ENSBTAG00000013588 | ENSBTAG00000018160 |  |  |
| ENSBTAG00000013589 | ENSBTAG00000018161 |  |  |
| ENSBTAG00000013593 | ENSBTAG00000018164 |  |  |
| ENSBTAG00000013596 | ENSBTAG00000018176 |  |  |
| ENSBTAG00000013602 | ENSBTAG00000018179 |  |  |
| ENSBTAG00000013606 | ENSBTAG00000018186 |  |  |
| ENSBTAG00000013607 | ENSBTAG00000018196 |  |  |
| ENSBTAG00000013611 | ENSBTAG00000018202 |  |  |
| ENSBTAG00000013612 | ENSBTAG00000018207 |  |  |
| ENSBTAG00000013614 | ENSBTAG00000018213 |  |  |
| ENSBTAG00000013615 | ENSBTAG00000018214 |  |  |
| ENSBTAG00000013620 | ENSBTAG00000018216 |  |  |
| ENSBTAG00000013621 | ENSBTAG00000018218 |  |  |
| ENSBTAG00000013623 | ENSBTAG00000018223 |  |  |
| ENSBTAG00000013627 | ENSBTAG00000018227 |  |  |
| ENSBTAG00000013628 | ENSBTAG00000018236 |  |  |
| ENSBTAG00000013629 | ENSBTAG00000018238 |  |  |
| ENSBTAG00000013631 | ENSBTAG00000018239 |  |  |
| ENSBTAG00000013642 | ENSBTAG00000018240 |  |  |
| ENSBTAG00000013645 | ENSBTAG00000018245 |  |  |
| ENSBTAG00000013648 | ENSBTAG00000018247 |  |  |
| ENSBTAG00000013652 | ENSBTAG00000018252 |  |  |
| ENSBTAG00000013653 | ENSBTAG00000018253 |  |  |
| ENSBTAG00000013662 | ENSBTAG00000018254 |  |  |
| ENSBTAG00000013666 | ENSBTAG00000018256 |  |  |
| ENSBTAG00000013669 | ENSBTAG00000018257 |  |  |
| ENSBTAG00000013670 | ENSBTAG00000018258 |  |  |
| ENSBTAG00000013671 | ENSBTAG00000018261 |  |  |
| ENSBTAG00000013674 | ENSBTAG00000018262 |  |  |
| ENSBTAG00000013675 | ENSBTAG00000018263 |  |  |
| ENSBTAG00000013677 | ENSBTAG00000018271 |  |  |
| ENSBTAG00000013685 | ENSBTAG00000018281 |  |  |
| ENSBTAG00000013688 | ENSBTAG00000018285 |  |  |
| ENSBTAG00000013689 | ENSBTAG00000018286 |  |  |
| ENSBTAG00000013698 | ENSBTAG00000018287 |  |  |
| ENSBTAG00000013699 | ENSBTAG00000018289 |  |  |
| ENSBTAG00000013705 | ENSBTAG00000018291 |  |  |
| ENSBTAG00000013720 | ENSBTAG00000018292 |  |  |
| ENSBTAG00000013721 | ENSBTAG00000018295 |  |  |
| ENSBTAG00000013724 | ENSBTAG00000018299 |  |  |
| ENSBTAG00000013727 | ENSBTAG00000018300 |  |  |
| ENSBTAG00000013730 | ENSBTAG00000018313 |  |  |
| ENSBTAG00000013732 | ENSBTAG00000018317 |  |  |
| ENSBTAG00000013734 | ENSBTAG00000018318 |  |  |
| ENSBTAG00000013735 | ENSBTAG00000018321 |  |  |
| ENSBTAG00000013740 | ENSBTAG00000018322 |  |  |
| ENSBTAG00000013741 | ENSBTAG00000018339 |  |  |
| ENSBTAG00000013745 | ENSBTAG00000018345 |  |  |
| ENSBTAG00000013749 | ENSBTAG00000018347 |  |  |
| ENSBTAG00000013750 | ENSBTAG00000018348 |  |  |
| ENSBTAG00000013753 | ENSBTAG00000018361 |  |  |
| ENSBTAG00000013755 | ENSBTAG00000018362 |  |  |
| ENSBTAG00000013757 | ENSBTAG00000018363 |  |  |
| ENSBTAG00000013761 | ENSBTAG00000018365 |  |  |
| ENSBTAG00000013763 | ENSBTAG00000018372 |  |  |
| ENSBTAG00000013766 | ENSBTAG00000018373 |  |  |
| ENSBTAG00000013768 | ENSBTAG00000018374 |  |  |
| ENSBTAG00000013774 | ENSBTAG00000018381 |  |  |
| ENSBTAG00000013776 | ENSBTAG00000018386 |  |  |
| ENSBTAG00000013778 | ENSBTAG00000018391 |  |  |
| ENSBTAG00000013781 | ENSBTAG00000018400 |  |  |
| ENSBTAG00000013782 | ENSBTAG00000018402 |  |  |
| ENSBTAG00000013784 | ENSBTAG00000018406 |  |  |
| ENSBTAG00000013790 | ENSBTAG00000018413 |  |  |
| ENSBTAG00000013798 | ENSBTAG00000018417 |  |  |
| ENSBTAG00000013812 | ENSBTAG00000018421 |  |  |
| ENSBTAG00000013813 | ENSBTAG00000018422 |  |  |
| ENSBTAG00000013819 | ENSBTAG00000018423 |  |  |
| ENSBTAG00000013825 | ENSBTAG00000018427 |  |  |
| ENSBTAG00000013831 | ENSBTAG00000018432 |  |  |
| ENSBTAG00000013832 | ENSBTAG00000018436 |  |  |
| ENSBTAG00000013834 | ENSBTAG00000018446 |  |  |
| ENSBTAG00000013836 | ENSBTAG00000018452 |  |  |
| ENSBTAG00000013842 | ENSBTAG00000018464 |  |  |
| ENSBTAG00000013843 | ENSBTAG00000018465 |  |  |
| ENSBTAG00000013848 | ENSBTAG00000018467 |  |  |
| ENSBTAG00000013849 | ENSBTAG00000018474 |  |  |
| ENSBTAG00000013851 | ENSBTAG00000018482 |  |  |
| ENSBTAG00000013854 | ENSBTAG00000018488 |  |  |
| ENSBTAG00000013855 | ENSBTAG00000018493 |  |  |
| ENSBTAG00000013858 | ENSBTAG00000018497 |  |  |
| ENSBTAG00000013860 | ENSBTAG00000018499 |  |  |
| ENSBTAG00000013863 | ENSBTAG00000018513 |  |  |
| ENSBTAG00000013867 | ENSBTAG00000018517 |  |  |
| ENSBTAG00000013869 | ENSBTAG00000018522 |  |  |
| ENSBTAG00000013879 | ENSBTAG00000018538 |  |  |
| ENSBTAG00000013880 | ENSBTAG00000018540 |  |  |
| ENSBTAG00000013884 | ENSBTAG00000018548 |  |  |
| ENSBTAG00000013885 | ENSBTAG00000018556 |  |  |
| ENSBTAG00000013889 | ENSBTAG00000018557 |  |  |
| ENSBTAG00000013895 | ENSBTAG00000018562 |  |  |
| ENSBTAG00000013901 | ENSBTAG00000018563 |  |  |
| ENSBTAG00000013907 | ENSBTAG00000018564 |  |  |
| ENSBTAG00000013910 | ENSBTAG00000018566 |  |  |
| ENSBTAG00000013912 | ENSBTAG00000018569 |  |  |
| ENSBTAG00000013914 | ENSBTAG00000018572 |  |  |
| ENSBTAG00000013915 | ENSBTAG00000018576 |  |  |
| ENSBTAG00000013916 | ENSBTAG00000018578 |  |  |
| ENSBTAG00000013918 | ENSBTAG00000018579 |  |  |
| ENSBTAG00000013919 | ENSBTAG00000018581 |  |  |
| ENSBTAG00000013922 | ENSBTAG00000018588 |  |  |
| ENSBTAG00000013923 | ENSBTAG00000018589 |  |  |
| ENSBTAG00000013924 | ENSBTAG00000018604 |  |  |
| ENSBTAG00000013926 | ENSBTAG00000018607 |  |  |
| ENSBTAG00000013928 | ENSBTAG00000018610 |  |  |
| ENSBTAG00000013929 | ENSBTAG00000018616 |  |  |
| ENSBTAG00000013932 | ENSBTAG00000018622 |  |  |
| ENSBTAG00000013935 | ENSBTAG00000018625 |  |  |
| ENSBTAG00000013941 | ENSBTAG00000018631 |  |  |
| ENSBTAG00000013946 | ENSBTAG00000018637 |  |  |
| ENSBTAG00000013949 | ENSBTAG00000018646 |  |  |
| ENSBTAG00000013952 | ENSBTAG00000018647 |  |  |
| ENSBTAG00000013955 | ENSBTAG00000018650 |  |  |
| ENSBTAG00000013956 | ENSBTAG00000018653 |  |  |
| ENSBTAG00000013957 | ENSBTAG00000018656 |  |  |
| ENSBTAG00000013960 | ENSBTAG00000018661 |  |  |
| ENSBTAG00000013971 | ENSBTAG00000018663 |  |  |
| ENSBTAG00000013974 | ENSBTAG00000018667 |  |  |
| ENSBTAG00000013980 | ENSBTAG00000018671 |  |  |
| ENSBTAG00000013981 | ENSBTAG00000018688 |  |  |
| ENSBTAG00000013982 | ENSBTAG00000018690 |  |  |
| ENSBTAG00000013985 | ENSBTAG00000018691 |  |  |
| ENSBTAG00000013988 | ENSBTAG00000018694 |  |  |
| ENSBTAG00000013991 | ENSBTAG00000018697 |  |  |
| ENSBTAG00000013996 | ENSBTAG00000018699 |  |  |
| ENSBTAG00000013999 | ENSBTAG00000018703 |  |  |
| ENSBTAG00000014002 | ENSBTAG00000018706 |  |  |
| ENSBTAG00000014005 | ENSBTAG00000018707 |  |  |
| ENSBTAG00000014006 | ENSBTAG00000018711 |  |  |
| ENSBTAG00000014007 | ENSBTAG00000018715 |  |  |
| ENSBTAG00000014011 | ENSBTAG00000018717 |  |  |
| ENSBTAG00000014012 | ENSBTAG00000018722 |  |  |
| ENSBTAG00000014015 | ENSBTAG00000018723 |  |  |
| ENSBTAG00000014017 | ENSBTAG00000018725 |  |  |
| ENSBTAG00000014021 | ENSBTAG00000018729 |  |  |
| ENSBTAG00000014023 | ENSBTAG00000018732 |  |  |
| ENSBTAG00000014024 | ENSBTAG00000018739 |  |  |
| ENSBTAG00000014029 | ENSBTAG00000018742 |  |  |
| ENSBTAG00000014030 | ENSBTAG00000018743 |  |  |
| ENSBTAG00000014032 | ENSBTAG00000018745 |  |  |
| ENSBTAG00000014038 | ENSBTAG00000018746 |  |  |
| ENSBTAG00000014041 | ENSBTAG00000018749 |  |  |
| ENSBTAG00000014042 | ENSBTAG00000018753 |  |  |
| ENSBTAG00000014043 | ENSBTAG00000018761 |  |  |
| ENSBTAG00000014044 | ENSBTAG00000018770 |  |  |
| ENSBTAG00000014046 | ENSBTAG00000018772 |  |  |
| ENSBTAG00000014048 | ENSBTAG00000018773 |  |  |
| ENSBTAG00000014053 | ENSBTAG00000018783 |  |  |
| ENSBTAG00000014054 | ENSBTAG00000018795 |  |  |
| ENSBTAG00000014057 | ENSBTAG00000018796 |  |  |
| ENSBTAG00000014059 | ENSBTAG00000018802 |  |  |
| ENSBTAG00000014060 | ENSBTAG00000018803 |  |  |
| ENSBTAG00000014063 | ENSBTAG00000018804 |  |  |
| ENSBTAG00000014064 | ENSBTAG00000018808 |  |  |
| ENSBTAG00000014068 | ENSBTAG00000018809 |  |  |
| ENSBTAG00000014069 | ENSBTAG00000018824 |  |  |
| ENSBTAG00000014076 | ENSBTAG00000018829 |  |  |
| ENSBTAG00000014078 | ENSBTAG00000018833 |  |  |
| ENSBTAG00000014079 | ENSBTAG00000018834 |  |  |
| ENSBTAG00000014083 | ENSBTAG00000018848 |  |  |
| ENSBTAG00000014089 | ENSBTAG00000018851 |  |  |
| ENSBTAG00000014090 | ENSBTAG00000018852 |  |  |
| ENSBTAG00000014091 | ENSBTAG00000018854 |  |  |
| ENSBTAG00000014092 | ENSBTAG00000018857 |  |  |
| ENSBTAG00000014093 | ENSBTAG00000018859 |  |  |
| ENSBTAG00000014094 | ENSBTAG00000018863 |  |  |
| ENSBTAG00000014099 | ENSBTAG00000018873 |  |  |
| ENSBTAG00000014102 | ENSBTAG00000018879 |  |  |
| ENSBTAG00000014103 | ENSBTAG00000018880 |  |  |
| ENSBTAG00000014106 | ENSBTAG00000018882 |  |  |
| ENSBTAG00000014113 | ENSBTAG00000018889 |  |  |
| ENSBTAG00000014118 | ENSBTAG00000018893 |  |  |
| ENSBTAG00000014119 | ENSBTAG00000018894 |  |  |
| ENSBTAG00000014122 | ENSBTAG00000018897 |  |  |
| ENSBTAG00000014124 | ENSBTAG00000018905 |  |  |
| ENSBTAG00000014127 | ENSBTAG00000018914 |  |  |
| ENSBTAG00000014132 | ENSBTAG00000018921 |  |  |
| ENSBTAG00000014136 | ENSBTAG00000018925 |  |  |
| ENSBTAG00000014140 | ENSBTAG00000018935 |  |  |
| ENSBTAG00000014141 | ENSBTAG00000018936 |  |  |
| ENSBTAG00000014143 | ENSBTAG00000018937 |  |  |
| ENSBTAG00000014147 | ENSBTAG00000018938 |  |  |
| ENSBTAG00000014149 | ENSBTAG00000018941 |  |  |
| ENSBTAG00000014150 | ENSBTAG00000018942 |  |  |
| ENSBTAG00000014151 | ENSBTAG00000018945 |  |  |
| ENSBTAG00000014156 | ENSBTAG00000018948 |  |  |
| ENSBTAG00000014158 | ENSBTAG00000018969 |  |  |
| ENSBTAG00000014159 | ENSBTAG00000018971 |  |  |
| ENSBTAG00000014161 | ENSBTAG00000018973 |  |  |
| ENSBTAG00000014166 | ENSBTAG00000018975 |  |  |
| ENSBTAG00000014171 | ENSBTAG00000018979 |  |  |
| ENSBTAG00000014172 | ENSBTAG00000018984 |  |  |
| ENSBTAG00000014175 | ENSBTAG00000018989 |  |  |
| ENSBTAG00000014176 | ENSBTAG00000018991 |  |  |
| ENSBTAG00000014177 | ENSBTAG00000018996 |  |  |
| ENSBTAG00000014179 | ENSBTAG00000018999 |  |  |
| ENSBTAG00000014181 | ENSBTAG00000019002 |  |  |
| ENSBTAG00000014182 | ENSBTAG00000019008 |  |  |
| ENSBTAG00000014187 | ENSBTAG00000019011 |  |  |
| ENSBTAG00000014191 | ENSBTAG00000019014 |  |  |
| ENSBTAG00000014197 | ENSBTAG00000019017 |  |  |
| ENSBTAG00000014199 | ENSBTAG00000019020 |  |  |
| ENSBTAG00000014204 | ENSBTAG00000019023 |  |  |
| ENSBTAG00000014215 | ENSBTAG00000019024 |  |  |
| ENSBTAG00000014217 | ENSBTAG00000019026 |  |  |
| ENSBTAG00000014218 | ENSBTAG00000019027 |  |  |
| ENSBTAG00000014220 | ENSBTAG00000019029 |  |  |
| ENSBTAG00000014227 | ENSBTAG00000019037 |  |  |
| ENSBTAG00000014228 | ENSBTAG00000019044 |  |  |
| ENSBTAG00000014229 | ENSBTAG00000019045 |  |  |
| ENSBTAG00000014232 | ENSBTAG00000019052 |  |  |
| ENSBTAG00000014234 | ENSBTAG00000019060 |  |  |
| ENSBTAG00000014235 | ENSBTAG00000019061 |  |  |
| ENSBTAG00000014237 | ENSBTAG00000019065 |  |  |
| ENSBTAG00000014238 | ENSBTAG00000019066 |  |  |
| ENSBTAG00000014239 | ENSBTAG00000019067 |  |  |
| ENSBTAG00000014246 | ENSBTAG00000019069 |  |  |
| ENSBTAG00000014249 | ENSBTAG00000019070 |  |  |
| ENSBTAG00000014251 | ENSBTAG00000019071 |  |  |
| ENSBTAG00000014253 | ENSBTAG00000019073 |  |  |
| ENSBTAG00000014255 | ENSBTAG00000019075 |  |  |
| ENSBTAG00000014259 | ENSBTAG00000019076 |  |  |
| ENSBTAG00000014261 | ENSBTAG00000019079 |  |  |
| ENSBTAG00000014262 | ENSBTAG00000019080 |  |  |
| ENSBTAG00000014269 | ENSBTAG00000019081 |  |  |
| ENSBTAG00000014270 | ENSBTAG00000019082 |  |  |
| ENSBTAG00000014272 | ENSBTAG00000019090 |  |  |
| ENSBTAG00000014273 | ENSBTAG00000019092 |  |  |
| ENSBTAG00000014284 | ENSBTAG00000019094 |  |  |
| ENSBTAG00000014287 | ENSBTAG00000019097 |  |  |
| ENSBTAG00000014288 | ENSBTAG00000019105 |  |  |
| ENSBTAG00000014291 | ENSBTAG00000019107 |  |  |
| ENSBTAG00000014294 | ENSBTAG00000019115 |  |  |
| ENSBTAG00000014295 | ENSBTAG00000019116 |  |  |
| ENSBTAG00000014299 | ENSBTAG00000019124 |  |  |
| ENSBTAG00000014302 | ENSBTAG00000019125 |  |  |
| ENSBTAG00000014306 | ENSBTAG00000019129 |  |  |
| ENSBTAG00000014312 | ENSBTAG00000019130 |  |  |
| ENSBTAG00000014314 | ENSBTAG00000019131 |  |  |
| ENSBTAG00000014315 | ENSBTAG00000019132 |  |  |
| ENSBTAG00000014316 | ENSBTAG00000019136 |  |  |
| ENSBTAG00000014318 | ENSBTAG00000019138 |  |  |
| ENSBTAG00000014324 | ENSBTAG00000019139 |  |  |
| ENSBTAG00000014325 | ENSBTAG00000019140 |  |  |
| ENSBTAG00000014326 | ENSBTAG00000019143 |  |  |
| ENSBTAG00000014328 | ENSBTAG00000019146 |  |  |
| ENSBTAG00000014333 | ENSBTAG00000019150 |  |  |
| ENSBTAG00000014335 | ENSBTAG00000019153 |  |  |
| ENSBTAG00000014340 | ENSBTAG00000019157 |  |  |
| ENSBTAG00000014345 | ENSBTAG00000019164 |  |  |
| ENSBTAG00000014349 | ENSBTAG00000019167 |  |  |
| ENSBTAG00000014353 | ENSBTAG00000019175 |  |  |
| ENSBTAG00000014354 | ENSBTAG00000019177 |  |  |
| ENSBTAG00000014358 | ENSBTAG00000019180 |  |  |
| ENSBTAG00000014359 | ENSBTAG00000019181 |  |  |
| ENSBTAG00000014366 | ENSBTAG00000019182 |  |  |
| ENSBTAG00000014367 | ENSBTAG00000019183 |  |  |
| ENSBTAG00000014368 | ENSBTAG00000019184 |  |  |
| ENSBTAG00000014373 | ENSBTAG00000019187 |  |  |
| ENSBTAG00000014374 | ENSBTAG00000019192 |  |  |
| ENSBTAG00000014375 | ENSBTAG00000019194 |  |  |
| ENSBTAG00000014376 | ENSBTAG00000019209 |  |  |
| ENSBTAG00000014377 | ENSBTAG00000019210 |  |  |
| ENSBTAG00000014380 | ENSBTAG00000019211 |  |  |
| ENSBTAG00000014381 | ENSBTAG00000019213 |  |  |
| ENSBTAG00000014382 | ENSBTAG00000019215 |  |  |
| ENSBTAG00000014384 | ENSBTAG00000019218 |  |  |
| ENSBTAG00000014388 | ENSBTAG00000019225 |  |  |
| ENSBTAG00000014393 | ENSBTAG00000019229 |  |  |
| ENSBTAG00000014395 | ENSBTAG00000019235 |  |  |
| ENSBTAG00000014396 | ENSBTAG00000019241 |  |  |
| ENSBTAG00000014401 | ENSBTAG00000019242 |  |  |
| ENSBTAG00000014411 | ENSBTAG00000019246 |  |  |
| ENSBTAG00000014415 | ENSBTAG00000019251 |  |  |
| ENSBTAG00000014417 | ENSBTAG00000019255 |  |  |
| ENSBTAG00000014420 | ENSBTAG00000019256 |  |  |
| ENSBTAG00000014421 | ENSBTAG00000019262 |  |  |
| ENSBTAG00000014422 | ENSBTAG00000019265 |  |  |
| ENSBTAG00000014423 | ENSBTAG00000019267 |  |  |
| ENSBTAG00000014424 | ENSBTAG00000019271 |  |  |
| ENSBTAG00000014426 | ENSBTAG00000019273 |  |  |
| ENSBTAG00000014432 | ENSBTAG00000019275 |  |  |
| ENSBTAG00000014433 | ENSBTAG00000019277 |  |  |
| ENSBTAG00000014434 | ENSBTAG00000019285 |  |  |
| ENSBTAG00000014435 | ENSBTAG00000019290 |  |  |
| ENSBTAG00000014438 | ENSBTAG00000019291 |  |  |
| ENSBTAG00000014441 | ENSBTAG00000019297 |  |  |
| ENSBTAG00000014442 | ENSBTAG00000019300 |  |  |
| ENSBTAG00000014443 | ENSBTAG00000019309 |  |  |
| ENSBTAG00000014447 | ENSBTAG00000019312 |  |  |
| ENSBTAG00000014448 | ENSBTAG00000019314 |  |  |
| ENSBTAG00000014450 | ENSBTAG00000019315 |  |  |
| ENSBTAG00000014451 | ENSBTAG00000019322 |  |  |
| ENSBTAG00000014455 | ENSBTAG00000019334 |  |  |
| ENSBTAG00000014458 | ENSBTAG00000019336 |  |  |
| ENSBTAG00000014459 | ENSBTAG00000019341 |  |  |
| ENSBTAG00000014460 | ENSBTAG00000019343 |  |  |
| ENSBTAG00000014463 | ENSBTAG00000019345 |  |  |
| ENSBTAG00000014465 | ENSBTAG00000019347 |  |  |
| ENSBTAG00000014466 | ENSBTAG00000019353 |  |  |
| ENSBTAG00000014468 | ENSBTAG00000019368 |  |  |
| ENSBTAG00000014469 | ENSBTAG00000019369 |  |  |
| ENSBTAG00000014470 | ENSBTAG00000019371 |  |  |
| ENSBTAG00000014471 | ENSBTAG00000019376 |  |  |
| ENSBTAG00000014476 | ENSBTAG00000019379 |  |  |
| ENSBTAG00000014479 | ENSBTAG00000019382 |  |  |
| ENSBTAG00000014485 | ENSBTAG00000019387 |  |  |
| ENSBTAG00000014486 | ENSBTAG00000019404 |  |  |
| ENSBTAG00000014491 | ENSBTAG00000019406 |  |  |
| ENSBTAG00000014492 | ENSBTAG00000019412 |  |  |
| ENSBTAG00000014494 | ENSBTAG00000019416 |  |  |
| ENSBTAG00000014495 | ENSBTAG00000019417 |  |  |
| ENSBTAG00000014501 | ENSBTAG00000019423 |  |  |
| ENSBTAG00000014503 | ENSBTAG00000019425 |  |  |
| ENSBTAG00000014505 | ENSBTAG00000019428 |  |  |
| ENSBTAG00000014511 | ENSBTAG00000019434 |  |  |
| ENSBTAG00000014514 | ENSBTAG00000019436 |  |  |
| ENSBTAG00000014515 | ENSBTAG00000019443 |  |  |
| ENSBTAG00000014518 | ENSBTAG00000019448 |  |  |
| ENSBTAG00000014520 | ENSBTAG00000019458 |  |  |
| ENSBTAG00000014521 | ENSBTAG00000019459 |  |  |
| ENSBTAG00000014522 | ENSBTAG00000019461 |  |  |
| ENSBTAG00000014523 | ENSBTAG00000019463 |  |  |
| ENSBTAG00000014525 | ENSBTAG00000019465 |  |  |
| ENSBTAG00000014526 | ENSBTAG00000019468 |  |  |
| ENSBTAG00000014529 | ENSBTAG00000019470 |  |  |
| ENSBTAG00000014530 | ENSBTAG00000019471 |  |  |
| ENSBTAG00000014534 | ENSBTAG00000019479 |  |  |
| ENSBTAG00000014536 | ENSBTAG00000019480 |  |  |
| ENSBTAG00000014538 | ENSBTAG00000019486 |  |  |
| ENSBTAG00000014540 | ENSBTAG00000019488 |  |  |
| ENSBTAG00000014541 | ENSBTAG00000019492 |  |  |
| ENSBTAG00000014546 | ENSBTAG00000019495 |  |  |
| ENSBTAG00000014550 | ENSBTAG00000019496 |  |  |
| ENSBTAG00000014551 | ENSBTAG00000019497 |  |  |
| ENSBTAG00000014553 | ENSBTAG00000019498 |  |  |
| ENSBTAG00000014554 | ENSBTAG00000019500 |  |  |
| ENSBTAG00000014558 | ENSBTAG00000019501 |  |  |
| ENSBTAG00000014560 | ENSBTAG00000019506 |  |  |
| ENSBTAG00000014561 | ENSBTAG00000019508 |  |  |
| ENSBTAG00000014575 | ENSBTAG00000019509 |  |  |
| ENSBTAG00000014576 | ENSBTAG00000019510 |  |  |
| ENSBTAG00000014579 | ENSBTAG00000019518 |  |  |
| ENSBTAG00000014581 | ENSBTAG00000019522 |  |  |
| ENSBTAG00000014583 | ENSBTAG00000019524 |  |  |
| ENSBTAG00000014588 | ENSBTAG00000019526 |  |  |
| ENSBTAG00000014589 | ENSBTAG00000019533 |  |  |
| ENSBTAG00000014592 | ENSBTAG00000019534 |  |  |
| ENSBTAG00000014596 | ENSBTAG00000019536 |  |  |
| ENSBTAG00000014598 | ENSBTAG00000019537 |  |  |
| ENSBTAG00000014601 | ENSBTAG00000019545 |  |  |
| ENSBTAG00000014602 | ENSBTAG00000019548 |  |  |
| ENSBTAG00000014603 | ENSBTAG00000019552 |  |  |
| ENSBTAG00000014605 | ENSBTAG00000019557 |  |  |
| ENSBTAG00000014608 | ENSBTAG00000019563 |  |  |
| ENSBTAG00000014611 | ENSBTAG00000019565 |  |  |
| ENSBTAG00000014612 | ENSBTAG00000019567 |  |  |
| ENSBTAG00000014614 | ENSBTAG00000019569 |  |  |
| ENSBTAG00000014619 | ENSBTAG00000019574 |  |  |
| ENSBTAG00000014620 | ENSBTAG00000019577 |  |  |
| ENSBTAG00000014623 | ENSBTAG00000019581 |  |  |
| ENSBTAG00000014628 | ENSBTAG00000019585 |  |  |
| ENSBTAG00000014633 | ENSBTAG00000019589 |  |  |
| ENSBTAG00000014643 | ENSBTAG00000019591 |  |  |
| ENSBTAG00000014644 | ENSBTAG00000019592 |  |  |
| ENSBTAG00000014649 | ENSBTAG00000019593 |  |  |
| ENSBTAG00000014651 | ENSBTAG00000019600 |  |  |
| ENSBTAG00000014653 | ENSBTAG00000019603 |  |  |
| ENSBTAG00000014655 | ENSBTAG00000019604 |  |  |
| ENSBTAG00000014659 | ENSBTAG00000019608 |  |  |
| ENSBTAG00000014665 | ENSBTAG00000019611 |  |  |
| ENSBTAG00000014670 | ENSBTAG00000019612 |  |  |
| ENSBTAG00000014674 | ENSBTAG00000019621 |  |  |
| ENSBTAG00000014675 | ENSBTAG00000019625 |  |  |
| ENSBTAG00000014677 | ENSBTAG00000019627 |  |  |
| ENSBTAG00000014678 | ENSBTAG00000019630 |  |  |
| ENSBTAG00000014679 | ENSBTAG00000019636 |  |  |
| ENSBTAG00000014685 | ENSBTAG00000019639 |  |  |
| ENSBTAG00000014687 | ENSBTAG00000019645 |  |  |
| ENSBTAG00000014689 | ENSBTAG00000019651 |  |  |
| ENSBTAG00000014691 | ENSBTAG00000019652 |  |  |
| ENSBTAG00000014692 | ENSBTAG00000019658 |  |  |
| ENSBTAG00000014693 | ENSBTAG00000019660 |  |  |
| ENSBTAG00000014698 | ENSBTAG00000019662 |  |  |
| ENSBTAG00000014699 | ENSBTAG00000019663 |  |  |
| ENSBTAG00000014700 | ENSBTAG00000019665 |  |  |
| ENSBTAG00000014711 | ENSBTAG00000019672 |  |  |
| ENSBTAG00000014712 | ENSBTAG00000019675 |  |  |
| ENSBTAG00000014714 | ENSBTAG00000019676 |  |  |
| ENSBTAG00000014719 | ENSBTAG00000019680 |  |  |
| ENSBTAG00000014724 | ENSBTAG00000019684 |  |  |
| ENSBTAG00000014725 | ENSBTAG00000019685 |  |  |
| ENSBTAG00000014726 | ENSBTAG00000019686 |  |  |
| ENSBTAG00000014728 | ENSBTAG00000019692 |  |  |
| ENSBTAG00000014729 | ENSBTAG00000019694 |  |  |
| ENSBTAG00000014730 | ENSBTAG00000019703 |  |  |
| ENSBTAG00000014733 | ENSBTAG00000019704 |  |  |
| ENSBTAG00000014734 | ENSBTAG00000019707 |  |  |
| ENSBTAG00000014738 | ENSBTAG00000019708 |  |  |
| ENSBTAG00000014741 | ENSBTAG00000019712 |  |  |
| ENSBTAG00000014744 | ENSBTAG00000019716 |  |  |
| ENSBTAG00000014750 | ENSBTAG00000019722 |  |  |
| ENSBTAG00000014758 | ENSBTAG00000019723 |  |  |
| ENSBTAG00000014759 | ENSBTAG00000019729 |  |  |
| ENSBTAG00000014762 | ENSBTAG00000019733 |  |  |
| ENSBTAG00000014766 | ENSBTAG00000019741 |  |  |
| ENSBTAG00000014769 | ENSBTAG00000019742 |  |  |
| ENSBTAG00000014772 | ENSBTAG00000019750 |  |  |
| ENSBTAG00000014773 | ENSBTAG00000019754 |  |  |
| ENSBTAG00000014775 | ENSBTAG00000019755 |  |  |
| ENSBTAG00000014777 | ENSBTAG00000019767 |  |  |
| ENSBTAG00000014791 | ENSBTAG00000019772 |  |  |
| ENSBTAG00000014792 | ENSBTAG00000019779 |  |  |
| ENSBTAG00000014794 | ENSBTAG00000019781 |  |  |
| ENSBTAG00000014798 | ENSBTAG00000019782 |  |  |
| ENSBTAG00000014799 | ENSBTAG00000019783 |  |  |
| ENSBTAG00000014803 | ENSBTAG00000019794 |  |  |
| ENSBTAG00000014804 | ENSBTAG00000019798 |  |  |
| ENSBTAG00000014805 | ENSBTAG00000019803 |  |  |
| ENSBTAG00000014806 | ENSBTAG00000019807 |  |  |
| ENSBTAG00000014807 | ENSBTAG00000019808 |  |  |
| ENSBTAG00000014809 | ENSBTAG00000019810 |  |  |
| ENSBTAG00000014813 | ENSBTAG00000019811 |  |  |
| ENSBTAG00000014820 | ENSBTAG00000019812 |  |  |
| ENSBTAG00000014821 | ENSBTAG00000019818 |  |  |
| ENSBTAG00000014822 | ENSBTAG00000019830 |  |  |
| ENSBTAG00000014825 | ENSBTAG00000019832 |  |  |
| ENSBTAG00000014829 | ENSBTAG00000019836 |  |  |
| ENSBTAG00000014831 | ENSBTAG00000019838 |  |  |
| ENSBTAG00000014832 | ENSBTAG00000019841 |  |  |
| ENSBTAG00000014835 | ENSBTAG00000019845 |  |  |
| ENSBTAG00000014838 | ENSBTAG00000019846 |  |  |
| ENSBTAG00000014841 | ENSBTAG00000019848 |  |  |
| ENSBTAG00000014847 | ENSBTAG00000019852 |  |  |
| ENSBTAG00000014849 | ENSBTAG00000019854 |  |  |
| ENSBTAG00000014850 | ENSBTAG00000019855 |  |  |
| ENSBTAG00000014858 | ENSBTAG00000019857 |  |  |
| ENSBTAG00000014861 | ENSBTAG00000019859 |  |  |
| ENSBTAG00000014863 | ENSBTAG00000019869 |  |  |
| ENSBTAG00000014872 | ENSBTAG00000019870 |  |  |
| ENSBTAG00000014877 | ENSBTAG00000019872 |  |  |
| ENSBTAG00000014879 | ENSBTAG00000019876 |  |  |
| ENSBTAG00000014881 | ENSBTAG00000019877 |  |  |
| ENSBTAG00000014883 | ENSBTAG00000019883 |  |  |
| ENSBTAG00000014884 | ENSBTAG00000019886 |  |  |
| ENSBTAG00000014886 | ENSBTAG00000019887 |  |  |
| ENSBTAG00000014890 | ENSBTAG00000019889 |  |  |
| ENSBTAG00000014891 | ENSBTAG00000019892 |  |  |
| ENSBTAG00000014897 | ENSBTAG00000019906 |  |  |
| ENSBTAG00000014898 | ENSBTAG00000019907 |  |  |
| ENSBTAG00000014903 | ENSBTAG00000019910 |  |  |
| ENSBTAG00000014904 | ENSBTAG00000019911 |  |  |
| ENSBTAG00000014911 | ENSBTAG00000019914 |  |  |
| ENSBTAG00000014912 | ENSBTAG00000019915 |  |  |
| ENSBTAG00000014915 | ENSBTAG00000019924 |  |  |
| ENSBTAG00000014916 | ENSBTAG00000019926 |  |  |
| ENSBTAG00000014917 | ENSBTAG00000019930 |  |  |
| ENSBTAG00000014918 | ENSBTAG00000019933 |  |  |
| ENSBTAG00000014921 | ENSBTAG00000019937 |  |  |
| ENSBTAG00000014922 | ENSBTAG00000019944 |  |  |
| ENSBTAG00000014923 | ENSBTAG00000019948 |  |  |
| ENSBTAG00000014926 | ENSBTAG00000019950 |  |  |
| ENSBTAG00000014930 | ENSBTAG00000019952 |  |  |
| ENSBTAG00000014931 | ENSBTAG00000019953 |  |  |
| ENSBTAG00000014936 | ENSBTAG00000019954 |  |  |
| ENSBTAG00000014940 | ENSBTAG00000019956 |  |  |
| ENSBTAG00000014943 | ENSBTAG00000019958 |  |  |
| ENSBTAG00000014944 | ENSBTAG00000019963 |  |  |
| ENSBTAG00000014958 | ENSBTAG00000019967 |  |  |
| ENSBTAG00000014960 | ENSBTAG00000019972 |  |  |
| ENSBTAG00000014966 | ENSBTAG00000019984 |  |  |
| ENSBTAG00000014969 | ENSBTAG00000019986 |  |  |
| ENSBTAG00000014970 | ENSBTAG00000019987 |  |  |
| ENSBTAG00000014972 | ENSBTAG00000019988 |  |  |
| ENSBTAG00000014974 | ENSBTAG00000020014 |  |  |
| ENSBTAG00000014975 | ENSBTAG00000020015 |  |  |
| ENSBTAG00000014981 | ENSBTAG00000020017 |  |  |
| ENSBTAG00000014992 | ENSBTAG00000020018 |  |  |
| ENSBTAG00000014995 | ENSBTAG00000020022 |  |  |
| ENSBTAG00000015000 | ENSBTAG00000020030 |  |  |
| ENSBTAG00000015004 | ENSBTAG00000020031 |  |  |
| ENSBTAG00000015005 | ENSBTAG00000020034 |  |  |
| ENSBTAG00000015008 | ENSBTAG00000020035 |  |  |
| ENSBTAG00000015009 | ENSBTAG00000020037 |  |  |
| ENSBTAG00000015013 | ENSBTAG00000020042 |  |  |
| ENSBTAG00000015014 | ENSBTAG00000020043 |  |  |
| ENSBTAG00000015015 | ENSBTAG00000020048 |  |  |
| ENSBTAG00000015016 | ENSBTAG00000020051 |  |  |
| ENSBTAG00000015017 | ENSBTAG00000020054 |  |  |
| ENSBTAG00000015019 | ENSBTAG00000020060 |  |  |
| ENSBTAG00000015023 | ENSBTAG00000020066 |  |  |
| ENSBTAG00000015025 | ENSBTAG00000020067 |  |  |
| ENSBTAG00000015026 | ENSBTAG00000020072 |  |  |
| ENSBTAG00000015032 | ENSBTAG00000020073 |  |  |
| ENSBTAG00000015034 | ENSBTAG00000020074 |  |  |
| ENSBTAG00000015036 | ENSBTAG00000020079 |  |  |
| ENSBTAG00000015038 | ENSBTAG00000020084 |  |  |
| ENSBTAG00000015040 | ENSBTAG00000020087 |  |  |
| ENSBTAG00000015041 | ENSBTAG00000020090 |  |  |
| ENSBTAG00000015042 | ENSBTAG00000020097 |  |  |
| ENSBTAG00000015043 | ENSBTAG00000020099 |  |  |
| ENSBTAG00000015044 | ENSBTAG00000020106 |  |  |
| ENSBTAG00000015046 | ENSBTAG00000020115 |  |  |
| ENSBTAG00000015047 | ENSBTAG00000020116 |  |  |
| ENSBTAG00000015053 | ENSBTAG00000020123 |  |  |
| ENSBTAG00000015056 | ENSBTAG00000020126 |  |  |
| ENSBTAG00000015060 | ENSBTAG00000020127 |  |  |
| ENSBTAG00000015061 | ENSBTAG00000020136 |  |  |
| ENSBTAG00000015066 | ENSBTAG00000020147 |  |  |
| ENSBTAG00000015069 | ENSBTAG00000020148 |  |  |
| ENSBTAG00000015073 | ENSBTAG00000020149 |  |  |
| ENSBTAG00000015076 | ENSBTAG00000020153 |  |  |
| ENSBTAG00000015080 | ENSBTAG00000020160 |  |  |
| ENSBTAG00000015081 | ENSBTAG00000020172 |  |  |
| ENSBTAG00000015083 | ENSBTAG00000020173 |  |  |
| ENSBTAG00000015085 | ENSBTAG00000020174 |  |  |
| ENSBTAG00000015086 | ENSBTAG00000020180 |  |  |
| ENSBTAG00000015090 | ENSBTAG00000020183 |  |  |
| ENSBTAG00000015092 | ENSBTAG00000020190 |  |  |
| ENSBTAG00000015097 | ENSBTAG00000020192 |  |  |
| ENSBTAG00000015100 | ENSBTAG00000020193 |  |  |
| ENSBTAG00000015103 | ENSBTAG00000020194 |  |  |
| ENSBTAG00000015104 | ENSBTAG00000020199 |  |  |
| ENSBTAG00000015105 | ENSBTAG00000020202 |  |  |
| ENSBTAG00000015106 | ENSBTAG00000020218 |  |  |
| ENSBTAG00000015107 | ENSBTAG00000020219 |  |  |
| ENSBTAG00000015109 | ENSBTAG00000020227 |  |  |
| ENSBTAG00000015113 | ENSBTAG00000020229 |  |  |
| ENSBTAG00000015114 | ENSBTAG00000020232 |  |  |
| ENSBTAG00000015116 | ENSBTAG00000020233 |  |  |
| ENSBTAG00000015118 | ENSBTAG00000020237 |  |  |
| ENSBTAG00000015119 | ENSBTAG00000020244 |  |  |
| ENSBTAG00000015125 | ENSBTAG00000020250 |  |  |
| ENSBTAG00000015127 | ENSBTAG00000020252 |  |  |
| ENSBTAG00000015129 | ENSBTAG00000020257 |  |  |
| ENSBTAG00000015130 | ENSBTAG00000020262 |  |  |
| ENSBTAG00000015131 | ENSBTAG00000020269 |  |  |
| ENSBTAG00000015132 | ENSBTAG00000020270 |  |  |
| ENSBTAG00000015133 | ENSBTAG00000020272 |  |  |
| ENSBTAG00000015136 | ENSBTAG00000020273 |  |  |
| ENSBTAG00000015142 | ENSBTAG00000020282 |  |  |
| ENSBTAG00000015144 | ENSBTAG00000020294 |  |  |
| ENSBTAG00000015145 | ENSBTAG00000020296 |  |  |
| ENSBTAG00000015151 | ENSBTAG00000020297 |  |  |
| ENSBTAG00000015154 | ENSBTAG00000020303 |  |  |
| ENSBTAG00000015155 | ENSBTAG00000020307 |  |  |
| ENSBTAG00000015158 | ENSBTAG00000020308 |  |  |
| ENSBTAG00000015163 | ENSBTAG00000020311 |  |  |
| ENSBTAG00000015169 | ENSBTAG00000020314 |  |  |
| ENSBTAG00000015171 | ENSBTAG00000020318 |  |  |
| ENSBTAG00000015177 | ENSBTAG00000020321 |  |  |
| ENSBTAG00000015181 | ENSBTAG00000020327 |  |  |
| ENSBTAG00000015184 | ENSBTAG00000020330 |  |  |
| ENSBTAG00000015186 | ENSBTAG00000020334 |  |  |
| ENSBTAG00000015188 | ENSBTAG00000020340 |  |  |
| ENSBTAG00000015192 | ENSBTAG00000020342 |  |  |
| ENSBTAG00000015195 | ENSBTAG00000020344 |  |  |
| ENSBTAG00000015200 | ENSBTAG00000020345 |  |  |
| ENSBTAG00000015202 | ENSBTAG00000020355 |  |  |
| ENSBTAG00000015205 | ENSBTAG00000020356 |  |  |
| ENSBTAG00000015209 | ENSBTAG00000020357 |  |  |
| ENSBTAG00000015212 | ENSBTAG00000020374 |  |  |
| ENSBTAG00000015214 | ENSBTAG00000020375 |  |  |
| ENSBTAG00000015217 | ENSBTAG00000020376 |  |  |
| ENSBTAG00000015220 | ENSBTAG00000020381 |  |  |
| ENSBTAG00000015221 | ENSBTAG00000020382 |  |  |
| ENSBTAG00000015222 | ENSBTAG00000020387 |  |  |
| ENSBTAG00000015225 | ENSBTAG00000020390 |  |  |
| ENSBTAG00000015228 | ENSBTAG00000020393 |  |  |
| ENSBTAG00000015229 | ENSBTAG00000020394 |  |  |
| ENSBTAG00000015230 | ENSBTAG00000020395 |  |  |
| ENSBTAG00000015232 | ENSBTAG00000020404 |  |  |
| ENSBTAG00000015235 | ENSBTAG00000020407 |  |  |
| ENSBTAG00000015238 | ENSBTAG00000020412 |  |  |
| ENSBTAG00000015240 | ENSBTAG00000020413 |  |  |
| ENSBTAG00000015241 | ENSBTAG00000020417 |  |  |
| ENSBTAG00000015248 | ENSBTAG00000020418 |  |  |
| ENSBTAG00000015249 | ENSBTAG00000020421 |  |  |
| ENSBTAG00000015251 | ENSBTAG00000020423 |  |  |
| ENSBTAG00000015253 | ENSBTAG00000020427 |  |  |
| ENSBTAG00000015254 | ENSBTAG00000020431 |  |  |
| ENSBTAG00000015255 | ENSBTAG00000020432 |  |  |
| ENSBTAG00000015266 | ENSBTAG00000020434 |  |  |
| ENSBTAG00000015267 | ENSBTAG00000020441 |  |  |
| ENSBTAG00000015268 | ENSBTAG00000020446 |  |  |
| ENSBTAG00000015272 | ENSBTAG00000020449 |  |  |
| ENSBTAG00000015276 | ENSBTAG00000020454 |  |  |
| ENSBTAG00000015278 | ENSBTAG00000020455 |  |  |
| ENSBTAG00000015280 | ENSBTAG00000020458 |  |  |
| ENSBTAG00000015286 | ENSBTAG00000020465 |  |  |
| ENSBTAG00000015294 | ENSBTAG00000020475 |  |  |
| ENSBTAG00000015297 | ENSBTAG00000020481 |  |  |
| ENSBTAG00000015301 | ENSBTAG00000020482 |  |  |
| ENSBTAG00000015304 | ENSBTAG00000020494 |  |  |
| ENSBTAG00000015308 | ENSBTAG00000020495 |  |  |
| ENSBTAG00000015309 | ENSBTAG00000020496 |  |  |
| ENSBTAG00000015311 | ENSBTAG00000020504 |  |  |
| ENSBTAG00000015312 | ENSBTAG00000020518 |  |  |
| ENSBTAG00000015313 | ENSBTAG00000020520 |  |  |
| ENSBTAG00000015316 | ENSBTAG00000020527 |  |  |
| ENSBTAG00000015321 | ENSBTAG00000020540 |  |  |
| ENSBTAG00000015326 | ENSBTAG00000020541 |  |  |
| ENSBTAG00000015327 | ENSBTAG00000020542 |  |  |
| ENSBTAG00000015329 | ENSBTAG00000020543 |  |  |
| ENSBTAG00000015334 | ENSBTAG00000020544 |  |  |
| ENSBTAG00000015335 | ENSBTAG00000020551 |  |  |
| ENSBTAG00000015336 | ENSBTAG00000020563 |  |  |
| ENSBTAG00000015337 | ENSBTAG00000020567 |  |  |
| ENSBTAG00000015338 | ENSBTAG00000020570 |  |  |
| ENSBTAG00000015339 | ENSBTAG00000020573 |  |  |
| ENSBTAG00000015340 | ENSBTAG00000020578 |  |  |
| ENSBTAG00000015343 | ENSBTAG00000020595 |  |  |
| ENSBTAG00000015345 | ENSBTAG00000020602 |  |  |
| ENSBTAG00000015348 | ENSBTAG00000020605 |  |  |
| ENSBTAG00000015350 | ENSBTAG00000020608 |  |  |
| ENSBTAG00000015351 | ENSBTAG00000020611 |  |  |
| ENSBTAG00000015352 | ENSBTAG00000020616 |  |  |
| ENSBTAG00000015354 | ENSBTAG00000020626 |  |  |
| ENSBTAG00000015356 | ENSBTAG00000020630 |  |  |
| ENSBTAG00000015358 | ENSBTAG00000020632 |  |  |
| ENSBTAG00000015362 | ENSBTAG00000020633 |  |  |
| ENSBTAG00000015363 | ENSBTAG00000020634 |  |  |
| ENSBTAG00000015366 | ENSBTAG00000020635 |  |  |
| ENSBTAG00000015368 | ENSBTAG00000020638 |  |  |
| ENSBTAG00000015369 | ENSBTAG00000020642 |  |  |
| ENSBTAG00000015371 | ENSBTAG00000020644 |  |  |
| ENSBTAG00000015375 | ENSBTAG00000020645 |  |  |
| ENSBTAG00000015377 | ENSBTAG00000020647 |  |  |
| ENSBTAG00000015378 | ENSBTAG00000020648 |  |  |
| ENSBTAG00000015379 | ENSBTAG00000020649 |  |  |
| ENSBTAG00000015381 | ENSBTAG00000020650 |  |  |
| ENSBTAG00000015384 | ENSBTAG00000020655 |  |  |
| ENSBTAG00000015385 | ENSBTAG00000020663 |  |  |
| ENSBTAG00000015387 | ENSBTAG00000020664 |  |  |
| ENSBTAG00000015390 | ENSBTAG00000020674 |  |  |
| ENSBTAG00000015394 | ENSBTAG00000020679 |  |  |
| ENSBTAG00000015401 | ENSBTAG00000020688 |  |  |
| ENSBTAG00000015413 | ENSBTAG00000020689 |  |  |
| ENSBTAG00000015414 | ENSBTAG00000020699 |  |  |
| ENSBTAG00000015416 | ENSBTAG00000020704 |  |  |
| ENSBTAG00000015424 | ENSBTAG00000020705 |  |  |
| ENSBTAG00000015428 | ENSBTAG00000020709 |  |  |
| ENSBTAG00000015432 | ENSBTAG00000020710 |  |  |
| ENSBTAG00000015436 | ENSBTAG00000020713 |  |  |
| ENSBTAG00000015437 | ENSBTAG00000020725 |  |  |
| ENSBTAG00000015438 | ENSBTAG00000020726 |  |  |
| ENSBTAG00000015450 | ENSBTAG00000020731 |  |  |
| ENSBTAG00000015457 | ENSBTAG00000020735 |  |  |
| ENSBTAG00000015459 | ENSBTAG00000020736 |  |  |
| ENSBTAG00000015460 | ENSBTAG00000020739 |  |  |
| ENSBTAG00000015466 | ENSBTAG00000020747 |  |  |
| ENSBTAG00000015478 | ENSBTAG00000020755 |  |  |
| ENSBTAG00000015482 | ENSBTAG00000020758 |  |  |
| ENSBTAG00000015487 | ENSBTAG00000020761 |  |  |
| ENSBTAG00000015497 | ENSBTAG00000020766 |  |  |
| ENSBTAG00000015498 | ENSBTAG00000020769 |  |  |
| ENSBTAG00000015505 | ENSBTAG00000020773 |  |  |
| ENSBTAG00000015513 | ENSBTAG00000020780 |  |  |
| ENSBTAG00000015519 | ENSBTAG00000020781 |  |  |
| ENSBTAG00000015520 | ENSBTAG00000020783 |  |  |
| ENSBTAG00000015525 | ENSBTAG00000020791 |  |  |
| ENSBTAG00000015527 | ENSBTAG00000020793 |  |  |
| ENSBTAG00000015529 | ENSBTAG00000020796 |  |  |
| ENSBTAG00000015532 | ENSBTAG00000020810 |  |  |
| ENSBTAG00000015534 | ENSBTAG00000020815 |  |  |
| ENSBTAG00000015536 | ENSBTAG00000020824 |  |  |
| ENSBTAG00000015538 | ENSBTAG00000020829 |  |  |
| ENSBTAG00000015543 | ENSBTAG00000020831 |  |  |
| ENSBTAG00000015546 | ENSBTAG00000020836 |  |  |
| ENSBTAG00000015547 | ENSBTAG00000020844 |  |  |
| ENSBTAG00000015549 | ENSBTAG00000020848 |  |  |
| ENSBTAG00000015550 | ENSBTAG00000020853 |  |  |
| ENSBTAG00000015559 | ENSBTAG00000020859 |  |  |
| ENSBTAG00000015567 | ENSBTAG00000020860 |  |  |
| ENSBTAG00000015572 | ENSBTAG00000020865 |  |  |
| ENSBTAG00000015580 | ENSBTAG00000020873 |  |  |
| ENSBTAG00000015581 | ENSBTAG00000020878 |  |  |
| ENSBTAG00000015582 | ENSBTAG00000020880 |  |  |
| ENSBTAG00000015590 | ENSBTAG00000020883 |  |  |
| ENSBTAG00000015591 | ENSBTAG00000020886 |  |  |
| ENSBTAG00000015596 | ENSBTAG00000020892 |  |  |
| ENSBTAG00000015602 | ENSBTAG00000020894 |  |  |
| ENSBTAG00000015604 | ENSBTAG00000020896 |  |  |
| ENSBTAG00000015607 | ENSBTAG00000020898 |  |  |
| ENSBTAG00000015611 | ENSBTAG00000020907 |  |  |
| ENSBTAG00000015612 | ENSBTAG00000020908 |  |  |
| ENSBTAG00000015614 | ENSBTAG00000020914 |  |  |
| ENSBTAG00000015615 | ENSBTAG00000020921 |  |  |
| ENSBTAG00000015625 | ENSBTAG00000020931 |  |  |
| ENSBTAG00000015628 | ENSBTAG00000020933 |  |  |
| ENSBTAG00000015630 | ENSBTAG00000020934 |  |  |
| ENSBTAG00000015636 | ENSBTAG00000020936 |  |  |
| ENSBTAG00000015637 | ENSBTAG00000020937 |  |  |
| ENSBTAG00000015639 | ENSBTAG00000020939 |  |  |
| ENSBTAG00000015642 | ENSBTAG00000020940 |  |  |
| ENSBTAG00000015644 | ENSBTAG00000020942 |  |  |
| ENSBTAG00000015649 | ENSBTAG00000020956 |  |  |
| ENSBTAG00000015655 | ENSBTAG00000020958 |  |  |
| ENSBTAG00000015656 | ENSBTAG00000020963 |  |  |
| ENSBTAG00000015659 | ENSBTAG00000020964 |  |  |
| ENSBTAG00000015669 | ENSBTAG00000020968 |  |  |
| ENSBTAG00000015678 | ENSBTAG00000020969 |  |  |
| ENSBTAG00000015681 | ENSBTAG00000020973 |  |  |
| ENSBTAG00000015683 | ENSBTAG00000020979 |  |  |
| ENSBTAG00000015686 | ENSBTAG00000020980 |  |  |
| ENSBTAG00000015694 | ENSBTAG00000020984 |  |  |
| ENSBTAG00000015700 | ENSBTAG00000020989 |  |  |
| ENSBTAG00000015702 | ENSBTAG00000020994 |  |  |
| ENSBTAG00000015704 | ENSBTAG00000020996 |  |  |
| ENSBTAG00000015708 | ENSBTAG00000020999 |  |  |
| ENSBTAG00000015710 | ENSBTAG00000021002 |  |  |
| ENSBTAG00000015711 | ENSBTAG00000021009 |  |  |
| ENSBTAG00000015713 | ENSBTAG00000021013 |  |  |
| ENSBTAG00000015716 | ENSBTAG00000021019 |  |  |
| ENSBTAG00000015717 | ENSBTAG00000021025 |  |  |
| ENSBTAG00000015718 | ENSBTAG00000021035 |  |  |
| ENSBTAG00000015719 | ENSBTAG00000021046 |  |  |
| ENSBTAG00000015721 | ENSBTAG00000021048 |  |  |
| ENSBTAG00000015723 | ENSBTAG00000021065 |  |  |
| ENSBTAG00000015724 | ENSBTAG00000021067 |  |  |
| ENSBTAG00000015727 | ENSBTAG00000021069 |  |  |
| ENSBTAG00000015728 | ENSBTAG00000021073 |  |  |
| ENSBTAG00000015731 | ENSBTAG00000021076 |  |  |
| ENSBTAG00000015735 | ENSBTAG00000021077 |  |  |
| ENSBTAG00000015738 | ENSBTAG00000021078 |  |  |
| ENSBTAG00000015739 | ENSBTAG00000021082 |  |  |
| ENSBTAG00000015743 | ENSBTAG00000021083 |  |  |
| ENSBTAG00000015744 | ENSBTAG00000021091 |  |  |
| ENSBTAG00000015745 | ENSBTAG00000021100 |  |  |
| ENSBTAG00000015751 | ENSBTAG00000021102 |  |  |
| ENSBTAG00000015757 | ENSBTAG00000021103 |  |  |
| ENSBTAG00000015758 | ENSBTAG00000021105 |  |  |
| ENSBTAG00000015761 | ENSBTAG00000021107 |  |  |
| ENSBTAG00000015763 | ENSBTAG00000021115 |  |  |
| ENSBTAG00000015764 | ENSBTAG00000021116 |  |  |
| ENSBTAG00000015767 | ENSBTAG00000021119 |  |  |
| ENSBTAG00000015769 | ENSBTAG00000021120 |  |  |
| ENSBTAG00000015771 | ENSBTAG00000021121 |  |  |
| ENSBTAG00000015776 | ENSBTAG00000021130 |  |  |
| ENSBTAG00000015778 | ENSBTAG00000021131 |  |  |
| ENSBTAG00000015780 | ENSBTAG00000021134 |  |  |
| ENSBTAG00000015781 | ENSBTAG00000021140 |  |  |
| ENSBTAG00000015784 | ENSBTAG00000021141 |  |  |
| ENSBTAG00000015786 | ENSBTAG00000021158 |  |  |
| ENSBTAG00000015788 | ENSBTAG00000021162 |  |  |
| ENSBTAG00000015789 | ENSBTAG00000021164 |  |  |
| ENSBTAG00000015796 | ENSBTAG00000021165 |  |  |
| ENSBTAG00000015801 | ENSBTAG00000021172 |  |  |
| ENSBTAG00000015802 | ENSBTAG00000021176 |  |  |
| ENSBTAG00000015804 | ENSBTAG00000021180 |  |  |
| ENSBTAG00000015806 | ENSBTAG00000021181 |  |  |
| ENSBTAG00000015807 | ENSBTAG00000021187 |  |  |
| ENSBTAG00000015810 | ENSBTAG00000021190 |  |  |
| ENSBTAG00000015817 | ENSBTAG00000021191 |  |  |
| ENSBTAG00000015818 | ENSBTAG00000021196 |  |  |
| ENSBTAG00000015820 | ENSBTAG00000021202 |  |  |
| ENSBTAG00000015821 | ENSBTAG00000021205 |  |  |
| ENSBTAG00000015834 | ENSBTAG00000021209 |  |  |
| ENSBTAG00000015837 | ENSBTAG00000021210 |  |  |
| ENSBTAG00000015839 | ENSBTAG00000021211 |  |  |
| ENSBTAG00000015841 | ENSBTAG00000021214 |  |  |
| ENSBTAG00000015853 | ENSBTAG00000021216 |  |  |
| ENSBTAG00000015856 | ENSBTAG00000021217 |  |  |
| ENSBTAG00000015857 | ENSBTAG00000021219 |  |  |
| ENSBTAG00000015858 | ENSBTAG00000021223 |  |  |
| ENSBTAG00000015860 | ENSBTAG00000021230 |  |  |
| ENSBTAG00000015868 | ENSBTAG00000021231 |  |  |
| ENSBTAG00000015874 | ENSBTAG00000021232 |  |  |
| ENSBTAG00000015878 | ENSBTAG00000021240 |  |  |
| ENSBTAG00000015879 | ENSBTAG00000021242 |  |  |
| ENSBTAG00000015880 | ENSBTAG00000021246 |  |  |
| ENSBTAG00000015885 | ENSBTAG00000021248 |  |  |
| ENSBTAG00000015889 | ENSBTAG00000021251 |  |  |
| ENSBTAG00000015891 | ENSBTAG00000021252 |  |  |
| ENSBTAG00000015893 | ENSBTAG00000021254 |  |  |
| ENSBTAG00000015895 | ENSBTAG00000021260 |  |  |
| ENSBTAG00000015898 | ENSBTAG00000021262 |  |  |
| ENSBTAG00000015900 | ENSBTAG00000021263 |  |  |
| ENSBTAG00000015901 | ENSBTAG00000021275 |  |  |
| ENSBTAG00000015902 | ENSBTAG00000021282 |  |  |
| ENSBTAG00000015908 | ENSBTAG00000021286 |  |  |
| ENSBTAG00000015909 | ENSBTAG00000021287 |  |  |
| ENSBTAG00000015910 | ENSBTAG00000021289 |  |  |
| ENSBTAG00000015915 | ENSBTAG00000021293 |  |  |
| ENSBTAG00000015917 | ENSBTAG00000021294 |  |  |
| ENSBTAG00000015920 | ENSBTAG00000021302 |  |  |
| ENSBTAG00000015924 | ENSBTAG00000021303 |  |  |
| ENSBTAG00000015925 | ENSBTAG00000021307 |  |  |
| ENSBTAG00000015931 | ENSBTAG00000021313 |  |  |
| ENSBTAG00000015935 | ENSBTAG00000021318 |  |  |
| ENSBTAG00000015936 | ENSBTAG00000021321 |  |  |
| ENSBTAG00000015938 | ENSBTAG00000021323 |  |  |
| ENSBTAG00000015944 | ENSBTAG00000021325 |  |  |
| ENSBTAG00000015945 | ENSBTAG00000021334 |  |  |
| ENSBTAG00000015946 | ENSBTAG00000021337 |  |  |
| ENSBTAG00000015955 | ENSBTAG00000021338 |  |  |
| ENSBTAG00000015958 | ENSBTAG00000021339 |  |  |
| ENSBTAG00000015961 | ENSBTAG00000021341 |  |  |
| ENSBTAG00000015962 | ENSBTAG00000021342 |  |  |
| ENSBTAG00000015965 | ENSBTAG00000021351 |  |  |
| ENSBTAG00000015972 | ENSBTAG00000021361 |  |  |
| ENSBTAG00000015973 | ENSBTAG00000021364 |  |  |
| ENSBTAG00000015976 | ENSBTAG00000021372 |  |  |
| ENSBTAG00000015977 | ENSBTAG00000021373 |  |  |
| ENSBTAG00000015979 | ENSBTAG00000021374 |  |  |
| ENSBTAG00000015980 | ENSBTAG00000021377 |  |  |
| ENSBTAG00000015981 | ENSBTAG00000021392 |  |  |
| ENSBTAG00000015982 | ENSBTAG00000021397 |  |  |
| ENSBTAG00000015984 | ENSBTAG00000021398 |  |  |
| ENSBTAG00000015986 | ENSBTAG00000021410 |  |  |
| ENSBTAG00000015994 | ENSBTAG00000021417 |  |  |
| ENSBTAG00000015996 | ENSBTAG00000021424 |  |  |
| ENSBTAG00000016000 | ENSBTAG00000021426 |  |  |
| ENSBTAG00000016004 | ENSBTAG00000021427 |  |  |
| ENSBTAG00000016005 | ENSBTAG00000021428 |  |  |
| ENSBTAG00000016007 | ENSBTAG00000021430 |  |  |
| ENSBTAG00000016010 | ENSBTAG00000021434 |  |  |
| ENSBTAG00000016013 | ENSBTAG00000021435 |  |  |
| ENSBTAG00000016026 | ENSBTAG00000021444 |  |  |
| ENSBTAG00000016028 | ENSBTAG00000021445 |  |  |
| ENSBTAG00000016030 | ENSBTAG00000021452 |  |  |
| ENSBTAG00000016032 | ENSBTAG00000021455 |  |  |
| ENSBTAG00000016033 | ENSBTAG00000021456 |  |  |
| ENSBTAG00000016037 | ENSBTAG00000021457 |  |  |
| ENSBTAG00000016041 | ENSBTAG00000021461 |  |  |
| ENSBTAG00000016042 | ENSBTAG00000021462 |  |  |
| ENSBTAG00000016043 | ENSBTAG00000021469 |  |  |
| ENSBTAG00000016045 | ENSBTAG00000021471 |  |  |
| ENSBTAG00000016050 | ENSBTAG00000021480 |  |  |
| ENSBTAG00000016053 | ENSBTAG00000021482 |  |  |
| ENSBTAG00000016057 | ENSBTAG00000021490 |  |  |
| ENSBTAG00000016060 | ENSBTAG00000021491 |  |  |
| ENSBTAG00000016061 | ENSBTAG00000021499 |  |  |
| ENSBTAG00000016063 | ENSBTAG00000021506 |  |  |
| ENSBTAG00000016071 | ENSBTAG00000021508 |  |  |
| ENSBTAG00000016073 | ENSBTAG00000021514 |  |  |
| ENSBTAG00000016075 | ENSBTAG00000021517 |  |  |
| ENSBTAG00000016077 | ENSBTAG00000021518 |  |  |
| ENSBTAG00000016078 | ENSBTAG00000021521 |  |  |
| ENSBTAG00000016080 | ENSBTAG00000021523 |  |  |
| ENSBTAG00000016081 | ENSBTAG00000021537 |  |  |
| ENSBTAG00000016085 | ENSBTAG00000021540 |  |  |
| ENSBTAG00000016091 | ENSBTAG00000021543 |  |  |
| ENSBTAG00000016092 | ENSBTAG00000021549 |  |  |
| ENSBTAG00000016093 | ENSBTAG00000021556 |  |  |
| ENSBTAG00000016094 | ENSBTAG00000021558 |  |  |
| ENSBTAG00000016096 | ENSBTAG00000021573 |  |  |
| ENSBTAG00000016098 | ENSBTAG00000021574 |  |  |
| ENSBTAG00000016101 | ENSBTAG00000021581 |  |  |
| ENSBTAG00000016103 | ENSBTAG00000021583 |  |  |
| ENSBTAG00000016105 | ENSBTAG00000021586 |  |  |
| ENSBTAG00000016109 | ENSBTAG00000021587 |  |  |
| ENSBTAG00000016121 | ENSBTAG00000021588 |  |  |
| ENSBTAG00000016124 | ENSBTAG00000021591 |  |  |
| ENSBTAG00000016128 | ENSBTAG00000021592 |  |  |
| ENSBTAG00000016133 | ENSBTAG00000021596 |  |  |
| ENSBTAG00000016137 | ENSBTAG00000021602 |  |  |
| ENSBTAG00000016147 | ENSBTAG00000021604 |  |  |
| ENSBTAG00000016149 | ENSBTAG00000021609 |  |  |
| ENSBTAG00000016152 | ENSBTAG00000021614 |  |  |
| ENSBTAG00000016156 | ENSBTAG00000021615 |  |  |
| ENSBTAG00000016165 | ENSBTAG00000021620 |  |  |
| ENSBTAG00000016168 | ENSBTAG00000021630 |  |  |
| ENSBTAG00000016169 | ENSBTAG00000021632 |  |  |
| ENSBTAG00000016170 | ENSBTAG00000021633 |  |  |
| ENSBTAG00000016174 | ENSBTAG00000021653 |  |  |
| ENSBTAG00000016175 | ENSBTAG00000021654 |  |  |
| ENSBTAG00000016197 | ENSBTAG00000021656 |  |  |
| ENSBTAG00000016199 | ENSBTAG00000021658 |  |  |
| ENSBTAG00000016206 | ENSBTAG00000021663 |  |  |
| ENSBTAG00000016208 | ENSBTAG00000021664 |  |  |
| ENSBTAG00000016209 | ENSBTAG00000021672 |  |  |
| ENSBTAG00000016210 | ENSBTAG00000021678 |  |  |
| ENSBTAG00000016211 | ENSBTAG00000021680 |  |  |
| ENSBTAG00000016213 | ENSBTAG00000021685 |  |  |
| ENSBTAG00000016217 | ENSBTAG00000021691 |  |  |
| ENSBTAG00000016218 | ENSBTAG00000021694 |  |  |
| ENSBTAG00000016220 | ENSBTAG00000021697 |  |  |
| ENSBTAG00000016223 | ENSBTAG00000021699 |  |  |
| ENSBTAG00000016225 | ENSBTAG00000021707 |  |  |
| ENSBTAG00000016228 | ENSBTAG00000021709 |  |  |
| ENSBTAG00000016231 | ENSBTAG00000021715 |  |  |
| ENSBTAG00000016235 | ENSBTAG00000021721 |  |  |
| ENSBTAG00000016236 | ENSBTAG00000021723 |  |  |
| ENSBTAG00000016240 | ENSBTAG00000021725 |  |  |
| ENSBTAG00000016242 | ENSBTAG00000021728 |  |  |
| ENSBTAG00000016243 | ENSBTAG00000021731 |  |  |
| ENSBTAG00000016251 | ENSBTAG00000021739 |  |  |
| ENSBTAG00000016252 | ENSBTAG00000021743 |  |  |
| ENSBTAG00000016253 | ENSBTAG00000021745 |  |  |
| ENSBTAG00000016254 | ENSBTAG00000021746 |  |  |
| ENSBTAG00000016255 | ENSBTAG00000021751 |  |  |
| ENSBTAG00000016260 | ENSBTAG00000021756 |  |  |
| ENSBTAG00000016263 | ENSBTAG00000021759 |  |  |
| ENSBTAG00000016264 | ENSBTAG00000021761 |  |  |
| ENSBTAG00000016265 | ENSBTAG00000021762 |  |  |
| ENSBTAG00000016271 | ENSBTAG00000021766 |  |  |
| ENSBTAG00000016275 | ENSBTAG00000021767 |  |  |
| ENSBTAG00000016276 | ENSBTAG00000021768 |  |  |
| ENSBTAG00000016277 | ENSBTAG00000021769 |  |  |
| ENSBTAG00000016282 | ENSBTAG00000021771 |  |  |
| ENSBTAG00000016287 | ENSBTAG00000021772 |  |  |
| ENSBTAG00000016293 | ENSBTAG00000021775 |  |  |
| ENSBTAG00000016294 | ENSBTAG00000021778 |  |  |
| ENSBTAG00000016295 | ENSBTAG00000021779 |  |  |
| ENSBTAG00000016296 | ENSBTAG00000021780 |  |  |
| ENSBTAG00000016298 | ENSBTAG00000021796 |  |  |
| ENSBTAG00000016299 | ENSBTAG00000021805 |  |  |
| ENSBTAG00000016302 | ENSBTAG00000021810 |  |  |
| ENSBTAG00000016305 | ENSBTAG00000021815 |  |  |
| ENSBTAG00000016307 | ENSBTAG00000021818 |  |  |
| ENSBTAG00000016309 | ENSBTAG00000021819 |  |  |
| ENSBTAG00000016311 | ENSBTAG00000021820 |  |  |
| ENSBTAG00000016315 | ENSBTAG00000021827 |  |  |
| ENSBTAG00000016316 | ENSBTAG00000021830 |  |  |
| ENSBTAG00000016320 | ENSBTAG00000021836 |  |  |
| ENSBTAG00000016323 | ENSBTAG00000021837 |  |  |
| ENSBTAG00000016326 | ENSBTAG00000021838 |  |  |
| ENSBTAG00000016327 | ENSBTAG00000021845 |  |  |
| ENSBTAG00000016328 | ENSBTAG00000021849 |  |  |
| ENSBTAG00000016336 | ENSBTAG00000021850 |  |  |
| ENSBTAG00000016337 | ENSBTAG00000021851 |  |  |
| ENSBTAG00000016341 | ENSBTAG00000021856 |  |  |
| ENSBTAG00000016343 | ENSBTAG00000021858 |  |  |
| ENSBTAG00000016344 | ENSBTAG00000021864 |  |  |
| ENSBTAG00000016349 | ENSBTAG00000021869 |  |  |
| ENSBTAG00000016350 | ENSBTAG00000021872 |  |  |
| ENSBTAG00000016352 | ENSBTAG00000021879 |  |  |
| ENSBTAG00000016354 | ENSBTAG00000021880 |  |  |
| ENSBTAG00000016357 | ENSBTAG00000021883 |  |  |
| ENSBTAG00000016360 | ENSBTAG00000021884 |  |  |
| ENSBTAG00000016363 | ENSBTAG00000021886 |  |  |
| ENSBTAG00000016366 | ENSBTAG00000021887 |  |  |
| ENSBTAG00000016367 | ENSBTAG00000021900 |  |  |
| ENSBTAG00000016369 | ENSBTAG00000021902 |  |  |
| ENSBTAG00000016377 | ENSBTAG00000021903 |  |  |
| ENSBTAG00000016378 | ENSBTAG00000021905 |  |  |
| ENSBTAG00000016384 | ENSBTAG00000021910 |  |  |
| ENSBTAG00000016387 | ENSBTAG00000021911 |  |  |
| ENSBTAG00000016389 | ENSBTAG00000021916 |  |  |
| ENSBTAG00000016396 | ENSBTAG00000021918 |  |  |
| ENSBTAG00000016397 | ENSBTAG00000021919 |  |  |
| ENSBTAG00000016398 | ENSBTAG00000021921 |  |  |
| ENSBTAG00000016399 | ENSBTAG00000021922 |  |  |
| ENSBTAG00000016407 | ENSBTAG00000021923 |  |  |
| ENSBTAG00000016411 | ENSBTAG00000021927 |  |  |
| ENSBTAG00000016412 | ENSBTAG00000021931 |  |  |
| ENSBTAG00000016413 | ENSBTAG00000021934 |  |  |
| ENSBTAG00000016415 | ENSBTAG00000021939 |  |  |
| ENSBTAG00000016420 | ENSBTAG00000021940 |  |  |
| ENSBTAG00000016424 | ENSBTAG00000021943 |  |  |
| ENSBTAG00000016427 | ENSBTAG00000021945 |  |  |
| ENSBTAG00000016432 | ENSBTAG00000021948 |  |  |
| ENSBTAG00000016441 | ENSBTAG00000021949 |  |  |
| ENSBTAG00000016442 | ENSBTAG00000021951 |  |  |
| ENSBTAG00000016445 | ENSBTAG00000021955 |  |  |
| ENSBTAG00000016450 | ENSBTAG00000021956 |  |  |
| ENSBTAG00000016451 | ENSBTAG00000021957 |  |  |
| ENSBTAG00000016456 | ENSBTAG00000021962 |  |  |
| ENSBTAG00000016457 | ENSBTAG00000021964 |  |  |
| ENSBTAG00000016465 | ENSBTAG00000021965 |  |  |
| ENSBTAG00000016467 | ENSBTAG00000021967 |  |  |
| ENSBTAG00000016469 | ENSBTAG00000021969 |  |  |
| ENSBTAG00000016472 | ENSBTAG00000021972 |  |  |
| ENSBTAG00000016473 | ENSBTAG00000021975 |  |  |
| ENSBTAG00000016481 | ENSBTAG00000021976 |  |  |
| ENSBTAG00000016486 | ENSBTAG00000021977 |  |  |
| ENSBTAG00000016494 | ENSBTAG00000021980 |  |  |
| ENSBTAG00000016501 | ENSBTAG00000021981 |  |  |
| ENSBTAG00000016502 | ENSBTAG00000021987 |  |  |
| ENSBTAG00000016505 | ENSBTAG00000021992 |  |  |
| ENSBTAG00000016506 | ENSBTAG00000021993 |  |  |
| ENSBTAG00000016508 | ENSBTAG00000021997 |  |  |
| ENSBTAG00000016511 | ENSBTAG00000022003 |  |  |
| ENSBTAG00000016514 | ENSBTAG00000022004 |  |  |
| ENSBTAG00000016516 | ENSBTAG00000022013 |  |  |
| ENSBTAG00000016519 | ENSBTAG00000022020 |  |  |
| ENSBTAG00000016522 | ENSBTAG00000022022 |  |  |
| ENSBTAG00000016523 | ENSBTAG00000022032 |  |  |
| ENSBTAG00000016524 | ENSBTAG00000022044 |  |  |
| ENSBTAG00000016527 | ENSBTAG00000022067 |  |  |
| ENSBTAG00000016529 | ENSBTAG00000022069 |  |  |
| ENSBTAG00000016532 | ENSBTAG00000022109 |  |  |
| ENSBTAG00000016533 | ENSBTAG00000022114 |  |  |
| ENSBTAG00000016541 | ENSBTAG00000022120 |  |  |
| ENSBTAG00000016542 | ENSBTAG00000022155 |  |  |
| ENSBTAG00000016546 | ENSBTAG00000022160 |  |  |
| ENSBTAG00000016549 | ENSBTAG00000022169 |  |  |
| ENSBTAG00000016551 | ENSBTAG00000022238 |  |  |
| ENSBTAG00000016552 | ENSBTAG00000022288 |  |  |
| ENSBTAG00000016557 | ENSBTAG00000022292 |  |  |
| ENSBTAG00000016561 | ENSBTAG00000022314 |  |  |
| ENSBTAG00000016562 | ENSBTAG00000022329 |  |  |
| ENSBTAG00000016563 | ENSBTAG00000022360 |  |  |
| ENSBTAG00000016567 | ENSBTAG00000022382 |  |  |
| ENSBTAG00000016571 | ENSBTAG00000022394 |  |  |
| ENSBTAG00000016572 | ENSBTAG00000022395 |  |  |
| ENSBTAG00000016573 | ENSBTAG00000022396 |  |  |
| ENSBTAG00000016577 | ENSBTAG00000022449 |  |  |
| ENSBTAG00000016578 | ENSBTAG00000022471 |  |  |
| ENSBTAG00000016581 | ENSBTAG00000022509 |  |  |
| ENSBTAG00000016591 | ENSBTAG00000022530 |  |  |
| ENSBTAG00000016593 | ENSBTAG00000022539 |  |  |
| ENSBTAG00000016594 | ENSBTAG00000022564 |  |  |
| ENSBTAG00000016595 | ENSBTAG00000022570 |  |  |
| ENSBTAG00000016596 | ENSBTAG00000022580 |  |  |
| ENSBTAG00000016599 | ENSBTAG00000022588 |  |  |
| ENSBTAG00000016609 | ENSBTAG00000022590 |  |  |
| ENSBTAG00000016612 | ENSBTAG00000022622 |  |  |
| ENSBTAG00000016613 | ENSBTAG00000022632 |  |  |
| ENSBTAG00000016615 | ENSBTAG00000022656 |  |  |
| ENSBTAG00000016618 | ENSBTAG00000022681 |  |  |
| ENSBTAG00000016619 | ENSBTAG00000022721 |  |  |
| ENSBTAG00000016622 | ENSBTAG00000022731 |  |  |
| ENSBTAG00000016627 | ENSBTAG00000022741 |  |  |
| ENSBTAG00000016633 | ENSBTAG00000022775 |  |  |
| ENSBTAG00000016635 | ENSBTAG00000022808 |  |  |
| ENSBTAG00000016640 | ENSBTAG00000022813 |  |  |
| ENSBTAG00000016648 | ENSBTAG00000022829 |  |  |
| ENSBTAG00000016650 | ENSBTAG00000022890 |  |  |
| ENSBTAG00000016651 | ENSBTAG00000022917 |  |  |
| ENSBTAG00000016656 | ENSBTAG00000022938 |  |  |
| ENSBTAG00000016661 | ENSBTAG00000022960 |  |  |
| ENSBTAG00000016662 | ENSBTAG00000022991 |  |  |
| ENSBTAG00000016663 | ENSBTAG00000023007 |  |  |
| ENSBTAG00000016664 | ENSBTAG00000023018 |  |  |
| ENSBTAG00000016666 | ENSBTAG00000023026 |  |  |
| ENSBTAG00000016667 | ENSBTAG00000023028 |  |  |
| ENSBTAG00000016676 | ENSBTAG00000023032 |  |  |
| ENSBTAG00000016679 | ENSBTAG00000023144 |  |  |
| ENSBTAG00000016680 | ENSBTAG00000023169 |  |  |
| ENSBTAG00000016683 | ENSBTAG00000023179 |  |  |
| ENSBTAG00000016691 | ENSBTAG00000023198 |  |  |
| ENSBTAG00000016694 | ENSBTAG00000023218 |  |  |
| ENSBTAG00000016696 | ENSBTAG00000023259 |  |  |
| ENSBTAG00000016698 | ENSBTAG00000023374 |  |  |
| ENSBTAG00000016703 | ENSBTAG00000023415 |  |  |
| ENSBTAG00000016704 | ENSBTAG00000023416 |  |  |
| ENSBTAG00000016707 | ENSBTAG00000023417 |  |  |
| ENSBTAG00000016708 | ENSBTAG00000023419 |  |  |
| ENSBTAG00000016709 | ENSBTAG00000023434 |  |  |
| ENSBTAG00000016716 | ENSBTAG00000023453 |  |  |
| ENSBTAG00000016720 | ENSBTAG00000023462 |  |  |
| ENSBTAG00000016723 | ENSBTAG00000023523 |  |  |
| ENSBTAG00000016724 | ENSBTAG00000023551 |  |  |
| ENSBTAG00000016725 | ENSBTAG00000023600 |  |  |
| ENSBTAG00000016730 | ENSBTAG00000023628 |  |  |
| ENSBTAG00000016731 | ENSBTAG00000023718 |  |  |
| ENSBTAG00000016738 | ENSBTAG00000023730 |  |  |
| ENSBTAG00000016740 | ENSBTAG00000023731 |  |  |
| ENSBTAG00000016742 | ENSBTAG00000023736 |  |  |
| ENSBTAG00000016744 | ENSBTAG00000023744 |  |  |
| ENSBTAG00000016747 | ENSBTAG00000023776 |  |  |
| ENSBTAG00000016751 | ENSBTAG00000023784 |  |  |
| ENSBTAG00000016758 | ENSBTAG00000023795 |  |  |
| ENSBTAG00000016762 | ENSBTAG00000023806 |  |  |
| ENSBTAG00000016763 | ENSBTAG00000023814 |  |  |
| ENSBTAG00000016764 | ENSBTAG00000023831 |  |  |
| ENSBTAG00000016765 | ENSBTAG00000023846 |  |  |
| ENSBTAG00000016766 | ENSBTAG00000023847 |  |  |
| ENSBTAG00000016769 | ENSBTAG00000023929 |  |  |
| ENSBTAG00000016770 | ENSBTAG00000023938 |  |  |
| ENSBTAG00000016771 | ENSBTAG00000023939 |  |  |
| ENSBTAG00000016775 | ENSBTAG00000023947 |  |  |
| ENSBTAG00000016776 | ENSBTAG00000023963 |  |  |
| ENSBTAG00000016779 | ENSBTAG00000023976 |  |  |
| ENSBTAG00000016782 | ENSBTAG00000023989 |  |  |
| ENSBTAG00000016783 | ENSBTAG00000024000 |  |  |
| ENSBTAG00000016791 | ENSBTAG00000024015 |  |  |
| ENSBTAG00000016797 | ENSBTAG00000024027 |  |  |
| ENSBTAG00000016800 | ENSBTAG00000024042 |  |  |
| ENSBTAG00000016801 | ENSBTAG00000024058 |  |  |
| ENSBTAG00000016804 | ENSBTAG00000024086 |  |  |
| ENSBTAG00000016805 | ENSBTAG00000024091 |  |  |
| ENSBTAG00000016810 | ENSBTAG00000024096 |  |  |
| ENSBTAG00000016818 | ENSBTAG00000024107 |  |  |
| ENSBTAG00000016819 | ENSBTAG00000024157 |  |  |
| ENSBTAG00000016821 | ENSBTAG00000024162 |  |  |
| ENSBTAG00000016823 | ENSBTAG00000024169 |  |  |
| ENSBTAG00000016826 | ENSBTAG00000024219 |  |  |
| ENSBTAG00000016827 | ENSBTAG00000024240 |  |  |
| ENSBTAG00000016828 | ENSBTAG00000024255 |  |  |
| ENSBTAG00000016830 | ENSBTAG00000024291 |  |  |
| ENSBTAG00000016833 | ENSBTAG00000024378 |  |  |
| ENSBTAG00000016838 | ENSBTAG00000024379 |  |  |
| ENSBTAG00000016841 | ENSBTAG00000024381 |  |  |
| ENSBTAG00000016844 | ENSBTAG00000024406 |  |  |
| ENSBTAG00000016845 | ENSBTAG00000024443 |  |  |
| ENSBTAG00000016846 | ENSBTAG00000024470 |  |  |
| ENSBTAG00000016847 | ENSBTAG00000024482 |  |  |
| ENSBTAG00000016848 | ENSBTAG00000024485 |  |  |
| ENSBTAG00000016849 | ENSBTAG00000024539 |  |  |
| ENSBTAG00000016855 | ENSBTAG00000024561 |  |  |
| ENSBTAG00000016857 | ENSBTAG00000024608 |  |  |
| ENSBTAG00000016864 | ENSBTAG00000024772 |  |  |
| ENSBTAG00000016867 | ENSBTAG00000024787 |  |  |
| ENSBTAG00000016869 | ENSBTAG00000024801 |  |  |
| ENSBTAG00000016873 | ENSBTAG00000024803 |  |  |
| ENSBTAG00000016874 | ENSBTAG00000024822 |  |  |
| ENSBTAG00000016878 | ENSBTAG00000024849 |  |  |
| ENSBTAG00000016881 | ENSBTAG00000024889 |  |  |
| ENSBTAG00000016882 | ENSBTAG00000024909 |  |  |
| ENSBTAG00000016885 | ENSBTAG00000024918 |  |  |
| ENSBTAG00000016887 | ENSBTAG00000024928 |  |  |
| ENSBTAG00000016890 | ENSBTAG00000024942 |  |  |
| ENSBTAG00000016891 | ENSBTAG00000024947 |  |  |
| ENSBTAG00000016896 | ENSBTAG00000024957 |  |  |
| ENSBTAG00000016900 | ENSBTAG00000024958 |  |  |
| ENSBTAG00000016904 | ENSBTAG00000024974 |  |  |
| ENSBTAG00000016906 | ENSBTAG00000024983 |  |  |
| ENSBTAG00000016908 | ENSBTAG00000025005 |  |  |
| ENSBTAG00000016910 | ENSBTAG00000025009 |  |  |
| ENSBTAG00000016913 | ENSBTAG00000025028 |  |  |
| ENSBTAG00000016918 | ENSBTAG00000025029 |  |  |
| ENSBTAG00000016924 | ENSBTAG00000025035 |  |  |
| ENSBTAG00000016926 | ENSBTAG00000025046 |  |  |
| ENSBTAG00000016932 | ENSBTAG00000025062 |  |  |
| ENSBTAG00000016933 | ENSBTAG00000025099 |  |  |
| ENSBTAG00000016939 | ENSBTAG00000025126 |  |  |
| ENSBTAG00000016948 | ENSBTAG00000025136 |  |  |
| ENSBTAG00000016950 | ENSBTAG00000025148 |  |  |
| ENSBTAG00000016952 | ENSBTAG00000025181 |  |  |
| ENSBTAG00000016956 | ENSBTAG00000025182 |  |  |
| ENSBTAG00000016957 | ENSBTAG00000025183 |  |  |
| ENSBTAG00000016959 | ENSBTAG00000025191 |  |  |
| ENSBTAG00000016961 | ENSBTAG00000025192 |  |  |
| ENSBTAG00000016963 | ENSBTAG00000025212 |  |  |
| ENSBTAG00000016968 | ENSBTAG00000025213 |  |  |
| ENSBTAG00000016969 | ENSBTAG00000025242 |  |  |
| ENSBTAG00000016973 | ENSBTAG00000025246 |  |  |
| ENSBTAG00000016977 | ENSBTAG00000025250 |  |  |
| ENSBTAG00000016980 | ENSBTAG00000025263 |  |  |
| ENSBTAG00000016984 | ENSBTAG00000025266 |  |  |
| ENSBTAG00000016988 | ENSBTAG00000025297 |  |  |
| ENSBTAG00000016991 | ENSBTAG00000025311 |  |  |
| ENSBTAG00000016995 | ENSBTAG00000025313 |  |  |
| ENSBTAG00000016998 | ENSBTAG00000025320 |  |  |
| ENSBTAG00000016999 | ENSBTAG00000025340 |  |  |
| ENSBTAG00000017001 | ENSBTAG00000025372 |  |  |
| ENSBTAG00000017002 | ENSBTAG00000025400 |  |  |
| ENSBTAG00000017007 | ENSBTAG00000025402 |  |  |
| ENSBTAG00000017015 | ENSBTAG00000025405 |  |  |
| ENSBTAG00000017016 | ENSBTAG00000025410 |  |  |
| ENSBTAG00000017017 | ENSBTAG00000025413 |  |  |
| ENSBTAG00000017019 | ENSBTAG00000025434 |  |  |
| ENSBTAG00000017024 | ENSBTAG00000025443 |  |  |
| ENSBTAG00000017028 | ENSBTAG00000025450 |  |  |
| ENSBTAG00000017035 | ENSBTAG00000025462 |  |  |
| ENSBTAG00000017038 | ENSBTAG00000025471 |  |  |
| ENSBTAG00000017040 | ENSBTAG00000025485 |  |  |
| ENSBTAG00000017042 | ENSBTAG00000025494 |  |  |
| ENSBTAG00000017053 | ENSBTAG00000025502 |  |  |
| ENSBTAG00000017056 | ENSBTAG00000025554 |  |  |
| ENSBTAG00000017060 | ENSBTAG00000025589 |  |  |
| ENSBTAG00000017061 | ENSBTAG00000025606 |  |  |
| ENSBTAG00000017063 | ENSBTAG00000025612 |  |  |
| ENSBTAG00000017064 | ENSBTAG00000025632 |  |  |
| ENSBTAG00000017067 | ENSBTAG00000025642 |  |  |
| ENSBTAG00000017068 | ENSBTAG00000025644 |  |  |
| ENSBTAG00000017069 | ENSBTAG00000025664 |  |  |
| ENSBTAG00000017071 | ENSBTAG00000025669 |  |  |
| ENSBTAG00000017072 | ENSBTAG00000025752 |  |  |
| ENSBTAG00000017075 | ENSBTAG00000025762 |  |  |
| ENSBTAG00000017078 | ENSBTAG00000025782 |  |  |
| ENSBTAG00000017079 | ENSBTAG00000025817 |  |  |
| ENSBTAG00000017082 | ENSBTAG00000025837 |  |  |
| ENSBTAG00000017086 | ENSBTAG00000025853 |  |  |
| ENSBTAG00000017087 | ENSBTAG00000025859 |  |  |
| ENSBTAG00000017091 | ENSBTAG00000025898 |  |  |
| ENSBTAG00000017094 | ENSBTAG00000025903 |  |  |
| ENSBTAG00000017098 | ENSBTAG00000025920 |  |  |
| ENSBTAG00000017104 | ENSBTAG00000025931 |  |  |
| ENSBTAG00000017115 | ENSBTAG00000025942 |  |  |
| ENSBTAG00000017116 | ENSBTAG00000026003 |  |  |
| ENSBTAG00000017118 | ENSBTAG00000026004 |  |  |
| ENSBTAG00000017122 | ENSBTAG00000026067 |  |  |
| ENSBTAG00000017125 | ENSBTAG00000026080 |  |  |
| ENSBTAG00000017128 | ENSBTAG00000026088 |  |  |
| ENSBTAG00000017129 | ENSBTAG00000026111 |  |  |
| ENSBTAG00000017132 | ENSBTAG00000026114 |  |  |
| ENSBTAG00000017133 | ENSBTAG00000026172 |  |  |
| ENSBTAG00000017135 | ENSBTAG00000026192 |  |  |
| ENSBTAG00000017139 | ENSBTAG00000026199 |  |  |
| ENSBTAG00000017141 | ENSBTAG00000026236 |  |  |
| ENSBTAG00000017143 | ENSBTAG00000026242 |  |  |
| ENSBTAG00000017147 | ENSBTAG00000026260 |  |  |
| ENSBTAG00000017155 | ENSBTAG00000026263 |  |  |
| ENSBTAG00000017158 | ENSBTAG00000026275 |  |  |
| ENSBTAG00000017160 | ENSBTAG00000026278 |  |  |
| ENSBTAG00000017162 | ENSBTAG00000026283 |  |  |
| ENSBTAG00000017165 | ENSBTAG00000026290 |  |  |
| ENSBTAG00000017179 | ENSBTAG00000026323 |  |  |
| ENSBTAG00000017183 | ENSBTAG00000026326 |  |  |
| ENSBTAG00000017184 | ENSBTAG00000026369 |  |  |
| ENSBTAG00000017187 | ENSBTAG00000026403 |  |  |
| ENSBTAG00000017188 | ENSBTAG00000026429 |  |  |
| ENSBTAG00000017189 | ENSBTAG00000026481 |  |  |
| ENSBTAG00000017196 | ENSBTAG00000026585 |  |  |
| ENSBTAG00000017198 | ENSBTAG00000026586 |  |  |
| ENSBTAG00000017200 | ENSBTAG00000026604 |  |  |
| ENSBTAG00000017209 | ENSBTAG00000026624 |  |  |
| ENSBTAG00000017213 | ENSBTAG00000026626 |  |  |
| ENSBTAG00000017218 | ENSBTAG00000026637 |  |  |
| ENSBTAG00000017220 | ENSBTAG00000026660 |  |  |
| ENSBTAG00000017223 | ENSBTAG00000026684 |  |  |
| ENSBTAG00000017225 | ENSBTAG00000026696 |  |  |
| ENSBTAG00000017239 | ENSBTAG00000026708 |  |  |
| ENSBTAG00000017240 | ENSBTAG00000026748 |  |  |
| ENSBTAG00000017242 | ENSBTAG00000026753 |  |  |
| ENSBTAG00000017243 | ENSBTAG00000026769 |  |  |
| ENSBTAG00000017244 | ENSBTAG00000026779 |  |  |
| ENSBTAG00000017249 | ENSBTAG00000026792 |  |  |
| ENSBTAG00000017251 | ENSBTAG00000026819 |  |  |
| ENSBTAG00000017255 | ENSBTAG00000026825 |  |  |
| ENSBTAG00000017256 | ENSBTAG00000026829 |  |  |
| ENSBTAG00000017263 | ENSBTAG00000026836 |  |  |
| ENSBTAG00000017265 | ENSBTAG00000026880 |  |  |
| ENSBTAG00000017266 | ENSBTAG00000026915 |  |  |
| ENSBTAG00000017267 | ENSBTAG00000026916 |  |  |
| ENSBTAG00000017268 | ENSBTAG00000026919 |  |  |
| ENSBTAG00000017275 | ENSBTAG00000026963 |  |  |
| ENSBTAG00000017277 | ENSBTAG00000026993 |  |  |
| ENSBTAG00000017279 | ENSBTAG00000026995 |  |  |
| ENSBTAG00000017283 | ENSBTAG00000027017 |  |  |
| ENSBTAG00000017284 | ENSBTAG00000027051 |  |  |
| ENSBTAG00000017287 | ENSBTAG00000027064 |  |  |
| ENSBTAG00000017296 | ENSBTAG00000027074 |  |  |
| ENSBTAG00000017310 | ENSBTAG00000027126 |  |  |
| ENSBTAG00000017313 | ENSBTAG00000027134 |  |  |
| ENSBTAG00000017318 | ENSBTAG00000027151 |  |  |
| ENSBTAG00000017321 | ENSBTAG00000027159 |  |  |
| ENSBTAG00000017325 | ENSBTAG00000027173 |  |  |
| ENSBTAG00000017326 | ENSBTAG00000027205 |  |  |
| ENSBTAG00000017328 | ENSBTAG00000027316 |  |  |
| ENSBTAG00000017329 | ENSBTAG00000027317 |  |  |
| ENSBTAG00000017339 | ENSBTAG00000027320 |  |  |
| ENSBTAG00000017346 | ENSBTAG00000027348 |  |  |
| ENSBTAG00000017349 | ENSBTAG00000027387 |  |  |
| ENSBTAG00000017354 | ENSBTAG00000027412 |  |  |
| ENSBTAG00000017357 | ENSBTAG00000027425 |  |  |
| ENSBTAG00000017361 | ENSBTAG00000027431 |  |  |
| ENSBTAG00000017362 | ENSBTAG00000027442 |  |  |
| ENSBTAG00000017363 | ENSBTAG00000027446 |  |  |
| ENSBTAG00000017366 | ENSBTAG00000027453 |  |  |
| ENSBTAG00000017367 | ENSBTAG00000027516 |  |  |
| ENSBTAG00000017368 | ENSBTAG00000027557 |  |  |
| ENSBTAG00000017369 | ENSBTAG00000027569 |  |  |
| ENSBTAG00000017371 | ENSBTAG00000027625 |  |  |
| ENSBTAG00000017373 | ENSBTAG00000027626 |  |  |
| ENSBTAG00000017375 | ENSBTAG00000027630 |  |  |
| ENSBTAG00000017379 | ENSBTAG00000027655 |  |  |
| ENSBTAG00000017380 | ENSBTAG00000027665 |  |  |
| ENSBTAG00000017381 | ENSBTAG00000027722 |  |  |
| ENSBTAG00000017382 | ENSBTAG00000027728 |  |  |
| ENSBTAG00000017388 | ENSBTAG00000027764 |  |  |
| ENSBTAG00000017390 | ENSBTAG00000027775 |  |  |
| ENSBTAG00000017393 | ENSBTAG00000027809 |  |  |
| ENSBTAG00000017395 | ENSBTAG00000027832 |  |  |
| ENSBTAG00000017397 | ENSBTAG00000027879 |  |  |
| ENSBTAG00000017401 | ENSBTAG00000027916 |  |  |
| ENSBTAG00000017402 | ENSBTAG00000027924 |  |  |
| ENSBTAG00000017404 | ENSBTAG00000027937 |  |  |
| ENSBTAG00000017405 | ENSBTAG00000027991 |  |  |
| ENSBTAG00000017407 | ENSBTAG00000030162 |  |  |
| ENSBTAG00000017409 | ENSBTAG00000030170 |  |  |
| ENSBTAG00000017411 | ENSBTAG00000030172 |  |  |
| ENSBTAG00000017419 | ENSBTAG00000030175 |  |  |
| ENSBTAG00000017420 | ENSBTAG00000030189 |  |  |
| ENSBTAG00000017421 | ENSBTAG00000030209 |  |  |
| ENSBTAG00000017423 | ENSBTAG00000030227 |  |  |
| ENSBTAG00000017424 | ENSBTAG00000030255 |  |  |
| ENSBTAG00000017425 | ENSBTAG00000030258 |  |  |
| ENSBTAG00000017426 | ENSBTAG00000030274 |  |  |
| ENSBTAG00000017429 | ENSBTAG00000030282 |  |  |
| ENSBTAG00000017435 | ENSBTAG00000030286 |  |  |
| ENSBTAG00000017436 | ENSBTAG00000030333 |  |  |
| ENSBTAG00000017437 | ENSBTAG00000030335 |  |  |
| ENSBTAG00000017439 | ENSBTAG00000030340 |  |  |
| ENSBTAG00000017442 | ENSBTAG00000030366 |  |  |
| ENSBTAG00000017443 | ENSBTAG00000030367 |  |  |
| ENSBTAG00000017448 | ENSBTAG00000030369 |  |  |
| ENSBTAG00000017450 | ENSBTAG00000030393 |  |  |
| ENSBTAG00000017451 | ENSBTAG00000030403 |  |  |
| ENSBTAG00000017452 | ENSBTAG00000030424 |  |  |
| ENSBTAG00000017455 | ENSBTAG00000030425 |  |  |
| ENSBTAG00000017458 | ENSBTAG00000030434 |  |  |
| ENSBTAG00000017459 | ENSBTAG00000030435 |  |  |
| ENSBTAG00000017461 | ENSBTAG00000030474 |  |  |
| ENSBTAG00000017465 | ENSBTAG00000030483 |  |  |
| ENSBTAG00000017468 | ENSBTAG00000030503 |  |  |
| ENSBTAG00000017478 | ENSBTAG00000030523 |  |  |
| ENSBTAG00000017490 | ENSBTAG00000030529 |  |  |
| ENSBTAG00000017492 | ENSBTAG00000030539 |  |  |
| ENSBTAG00000017505 | ENSBTAG00000030542 |  |  |
| ENSBTAG00000017508 | ENSBTAG00000030557 |  |  |
| ENSBTAG00000017509 | ENSBTAG00000030559 |  |  |
| ENSBTAG00000017512 | ENSBTAG00000030584 |  |  |
| ENSBTAG00000017517 | ENSBTAG00000030587 |  |  |
| ENSBTAG00000017524 | ENSBTAG00000030589 |  |  |
| ENSBTAG00000017527 | ENSBTAG00000030591 |  |  |
| ENSBTAG00000017529 | ENSBTAG00000030593 |  |  |
| ENSBTAG00000017537 | ENSBTAG00000030599 |  |  |
| ENSBTAG00000017540 | ENSBTAG00000030623 |  |  |
| ENSBTAG00000017541 | ENSBTAG00000030629 |  |  |
| ENSBTAG00000017545 | ENSBTAG00000030632 |  |  |
| ENSBTAG00000017547 | ENSBTAG00000030646 |  |  |
| ENSBTAG00000017549 | ENSBTAG00000030650 |  |  |
| ENSBTAG00000017550 | ENSBTAG00000030674 |  |  |
| ENSBTAG00000017557 | ENSBTAG00000030675 |  |  |
| ENSBTAG00000017560 | ENSBTAG00000030683 |  |  |
| ENSBTAG00000017564 | ENSBTAG00000030686 |  |  |
| ENSBTAG00000017565 | ENSBTAG00000030706 |  |  |
| ENSBTAG00000017566 | ENSBTAG00000030711 |  |  |
| ENSBTAG00000017569 | ENSBTAG00000030718 |  |  |
| ENSBTAG00000017571 | ENSBTAG00000030749 |  |  |
| ENSBTAG00000017573 | ENSBTAG00000030769 |  |  |
| ENSBTAG00000017582 | ENSBTAG00000030805 |  |  |
| ENSBTAG00000017584 | ENSBTAG00000030809 |  |  |
| ENSBTAG00000017592 | ENSBTAG00000030817 |  |  |
| ENSBTAG00000017593 | ENSBTAG00000030824 |  |  |
| ENSBTAG00000017599 | ENSBTAG00000030836 |  |  |
| ENSBTAG00000017602 | ENSBTAG00000030864 |  |  |
| ENSBTAG00000017604 | ENSBTAG00000030881 |  |  |
| ENSBTAG00000017605 | ENSBTAG00000030897 |  |  |
| ENSBTAG00000017611 | ENSBTAG00000030898 |  |  |
| ENSBTAG00000017613 | ENSBTAG00000030913 |  |  |
| ENSBTAG00000017616 | ENSBTAG00000030930 |  |  |
| ENSBTAG00000017618 | ENSBTAG00000030932 |  |  |
| ENSBTAG00000017622 | ENSBTAG00000030951 |  |  |
| ENSBTAG00000017624 | ENSBTAG00000030956 |  |  |
| ENSBTAG00000017631 | ENSBTAG00000030960 |  |  |
| ENSBTAG00000017633 | ENSBTAG00000030965 |  |  |
| ENSBTAG00000017636 | ENSBTAG00000030966 |  |  |
| ENSBTAG00000017639 | ENSBTAG00000030977 |  |  |
| ENSBTAG00000017647 | ENSBTAG00000031012 |  |  |
| ENSBTAG00000017652 | ENSBTAG00000031014 |  |  |
| ENSBTAG00000017655 | ENSBTAG00000031069 |  |  |
| ENSBTAG00000017656 | ENSBTAG00000031135 |  |  |
| ENSBTAG00000017659 | ENSBTAG00000031146 |  |  |
| ENSBTAG00000017660 | ENSBTAG00000031165 |  |  |
| ENSBTAG00000017661 | ENSBTAG00000031178 |  |  |
| ENSBTAG00000017662 | ENSBTAG00000031184 |  |  |
| ENSBTAG00000017665 | ENSBTAG00000031194 |  |  |
| ENSBTAG00000017676 | ENSBTAG00000031231 |  |  |
| ENSBTAG00000017677 | ENSBTAG00000031246 |  |  |
| ENSBTAG00000017679 | ENSBTAG00000031252 |  |  |
| ENSBTAG00000017680 | ENSBTAG00000031267 |  |  |
| ENSBTAG00000017681 | ENSBTAG00000031295 |  |  |
| ENSBTAG00000017683 | ENSBTAG00000031309 |  |  |
| ENSBTAG00000017685 | ENSBTAG00000031327 |  |  |
| ENSBTAG00000017689 | ENSBTAG00000031335 |  |  |
| ENSBTAG00000017690 | ENSBTAG00000031340 |  |  |
| ENSBTAG00000017694 | ENSBTAG00000031348 |  |  |
| ENSBTAG00000017695 | ENSBTAG00000031355 |  |  |
| ENSBTAG00000017711 | ENSBTAG00000031358 |  |  |
| ENSBTAG00000017713 | ENSBTAG00000031377 |  |  |
| ENSBTAG00000017715 | ENSBTAG00000031397 |  |  |
| ENSBTAG00000017718 | ENSBTAG00000031430 |  |  |
| ENSBTAG00000017719 | ENSBTAG00000031433 |  |  |
| ENSBTAG00000017729 | ENSBTAG00000031435 |  |  |
| ENSBTAG00000017738 | ENSBTAG00000031441 |  |  |
| ENSBTAG00000017739 | ENSBTAG00000031447 |  |  |
| ENSBTAG00000017740 | ENSBTAG00000031468 |  |  |
| ENSBTAG00000017741 | ENSBTAG00000031473 |  |  |
| ENSBTAG00000017747 | ENSBTAG00000031497 |  |  |
| ENSBTAG00000017748 | ENSBTAG00000031500 |  |  |
| ENSBTAG00000017753 | ENSBTAG00000031503 |  |  |
| ENSBTAG00000017765 | ENSBTAG00000031544 |  |  |
| ENSBTAG00000017767 | ENSBTAG00000031548 |  |  |
| ENSBTAG00000017769 | ENSBTAG00000031553 |  |  |
| ENSBTAG00000017776 | ENSBTAG00000031561 |  |  |
| ENSBTAG00000017779 | ENSBTAG00000031572 |  |  |
| ENSBTAG00000017780 | ENSBTAG00000031575 |  |  |
| ENSBTAG00000017781 | ENSBTAG00000031590 |  |  |
| ENSBTAG00000017783 | ENSBTAG00000031598 |  |  |
| ENSBTAG00000017797 | ENSBTAG00000031614 |  |  |
| ENSBTAG00000017799 | ENSBTAG00000031647 |  |  |
| ENSBTAG00000017801 | ENSBTAG00000031656 |  |  |
| ENSBTAG00000017804 | ENSBTAG00000031679 |  |  |
| ENSBTAG00000017805 | ENSBTAG00000031686 |  |  |
| ENSBTAG00000017809 | ENSBTAG00000031697 |  |  |
| ENSBTAG00000017810 | ENSBTAG00000031701 |  |  |
| ENSBTAG00000017811 | ENSBTAG00000031704 |  |  |
| ENSBTAG00000017812 | ENSBTAG00000031707 |  |  |
| ENSBTAG00000017814 | ENSBTAG00000031709 |  |  |
| ENSBTAG00000017818 | ENSBTAG00000031715 |  |  |
| ENSBTAG00000017824 | ENSBTAG00000031718 |  |  |
| ENSBTAG00000017825 | ENSBTAG00000031731 |  |  |
| ENSBTAG00000017830 | ENSBTAG00000031747 |  |  |
| ENSBTAG00000017831 | ENSBTAG00000031749 |  |  |
| ENSBTAG00000017832 | ENSBTAG00000031765 |  |  |
| ENSBTAG00000017833 | ENSBTAG00000031774 |  |  |
| ENSBTAG00000017834 | ENSBTAG00000031777 |  |  |
| ENSBTAG00000017835 | ENSBTAG00000031778 |  |  |
| ENSBTAG00000017839 | ENSBTAG00000031785 |  |  |
| ENSBTAG00000017840 | ENSBTAG00000031789 |  |  |
| ENSBTAG00000017843 | ENSBTAG00000031792 |  |  |
| ENSBTAG00000017845 | ENSBTAG00000031795 |  |  |
| ENSBTAG00000017846 | ENSBTAG00000031806 |  |  |
| ENSBTAG00000017847 | ENSBTAG00000031824 |  |  |
| ENSBTAG00000017850 | ENSBTAG00000031829 |  |  |
| ENSBTAG00000017851 | ENSBTAG00000031845 |  |  |
| ENSBTAG00000017852 | ENSBTAG00000031851 |  |  |
| ENSBTAG00000017855 | ENSBTAG00000031861 |  |  |
| ENSBTAG00000017856 | ENSBTAG00000031863 |  |  |
| ENSBTAG00000017860 | ENSBTAG00000031871 |  |  |
| ENSBTAG00000017863 | ENSBTAG00000031875 |  |  |
| ENSBTAG00000017864 | ENSBTAG00000031885 |  |  |
| ENSBTAG00000017866 | ENSBTAG00000031886 |  |  |
| ENSBTAG00000017867 | ENSBTAG00000031890 |  |  |
| ENSBTAG00000017868 | ENSBTAG00000031895 |  |  |
| ENSBTAG00000017869 | ENSBTAG00000031898 |  |  |
| ENSBTAG00000017871 | ENSBTAG00000031916 |  |  |
| ENSBTAG00000017872 | ENSBTAG00000031919 |  |  |
| ENSBTAG00000017873 | ENSBTAG00000031981 |  |  |
| ENSBTAG00000017875 | ENSBTAG00000031998 |  |  |
| ENSBTAG00000017877 | ENSBTAG00000032014 |  |  |
| ENSBTAG00000017882 | ENSBTAG00000032018 |  |  |
| ENSBTAG00000017889 | ENSBTAG00000032021 |  |  |
| ENSBTAG00000017890 | ENSBTAG00000032026 |  |  |
| ENSBTAG00000017895 | ENSBTAG00000032047 |  |  |
| ENSBTAG00000017905 | ENSBTAG00000032055 |  |  |
| ENSBTAG00000017913 | ENSBTAG00000032059 |  |  |
| ENSBTAG00000017919 | ENSBTAG00000032068 |  |  |
| ENSBTAG00000017922 | ENSBTAG00000032071 |  |  |
| ENSBTAG00000017929 | ENSBTAG00000032084 |  |  |
| ENSBTAG00000017932 | ENSBTAG00000032087 |  |  |
| ENSBTAG00000017941 | ENSBTAG00000032089 |  |  |
| ENSBTAG00000017942 | ENSBTAG00000032092 |  |  |
| ENSBTAG00000017943 | ENSBTAG00000032097 |  |  |
| ENSBTAG00000017946 | ENSBTAG00000032137 |  |  |
| ENSBTAG00000017956 | ENSBTAG00000032148 |  |  |
| ENSBTAG00000017958 | ENSBTAG00000032152 |  |  |
| ENSBTAG00000017967 | ENSBTAG00000032163 |  |  |
| ENSBTAG00000017970 | ENSBTAG00000032166 |  |  |
| ENSBTAG00000017981 | ENSBTAG00000032200 |  |  |
| ENSBTAG00000017992 | ENSBTAG00000032234 |  |  |
| ENSBTAG00000017996 | ENSBTAG00000032247 |  |  |
| ENSBTAG00000017999 | ENSBTAG00000032288 |  |  |
| ENSBTAG00000018000 | ENSBTAG00000032331 |  |  |
| ENSBTAG00000018002 | ENSBTAG00000032340 |  |  |
| ENSBTAG00000018003 | ENSBTAG00000032369 |  |  |
| ENSBTAG00000018007 | ENSBTAG00000032372 |  |  |
| ENSBTAG00000018010 | ENSBTAG00000032396 |  |  |
| ENSBTAG00000018011 | ENSBTAG00000032424 |  |  |
| ENSBTAG00000018015 | ENSBTAG00000032425 |  |  |
| ENSBTAG00000018016 | ENSBTAG00000032427 |  |  |
| ENSBTAG00000018020 | ENSBTAG00000032433 |  |  |
| ENSBTAG00000018025 | ENSBTAG00000032453 |  |  |
| ENSBTAG00000018026 | ENSBTAG00000032477 |  |  |
| ENSBTAG00000018033 | ENSBTAG00000032509 |  |  |
| ENSBTAG00000018035 | ENSBTAG00000032515 |  |  |
| ENSBTAG00000018036 | ENSBTAG00000032518 |  |  |
| ENSBTAG00000018037 | ENSBTAG00000032538 |  |  |
| ENSBTAG00000018041 | ENSBTAG00000032548 |  |  |
| ENSBTAG00000018044 | ENSBTAG00000032576 |  |  |
| ENSBTAG00000018046 | ENSBTAG00000032591 |  |  |
| ENSBTAG00000018048 | ENSBTAG00000032617 |  |  |
| ENSBTAG00000018050 | ENSBTAG00000032640 |  |  |
| ENSBTAG00000018053 | ENSBTAG00000032642 |  |  |
| ENSBTAG00000018063 | ENSBTAG00000032651 |  |  |
| ENSBTAG00000018065 | ENSBTAG00000032660 |  |  |
| ENSBTAG00000018067 | ENSBTAG00000032680 |  |  |
| ENSBTAG00000018070 | ENSBTAG00000032704 |  |  |
| ENSBTAG00000018071 | ENSBTAG00000032738 |  |  |
| ENSBTAG00000018072 | ENSBTAG00000032763 |  |  |
| ENSBTAG00000018073 | ENSBTAG00000032821 |  |  |
| ENSBTAG00000018077 | ENSBTAG00000032829 |  |  |
| ENSBTAG00000018082 | ENSBTAG00000032844 |  |  |
| ENSBTAG00000018084 | ENSBTAG00000032848 |  |  |
| ENSBTAG00000018085 | ENSBTAG00000032852 |  |  |
| ENSBTAG00000018094 | ENSBTAG00000032884 |  |  |
| ENSBTAG00000018097 | ENSBTAG00000032902 |  |  |
| ENSBTAG00000018103 | ENSBTAG00000032914 |  |  |
| ENSBTAG00000018106 | ENSBTAG00000032951 |  |  |
| ENSBTAG00000018114 | ENSBTAG00000032961 |  |  |
| ENSBTAG00000018115 | ENSBTAG00000032962 |  |  |
| ENSBTAG00000018116 | ENSBTAG00000032964 |  |  |
| ENSBTAG00000018119 | ENSBTAG00000032982 |  |  |
| ENSBTAG00000018123 | ENSBTAG00000032995 |  |  |
| ENSBTAG00000018126 | ENSBTAG00000032996 |  |  |
| ENSBTAG00000018127 | ENSBTAG00000032997 |  |  |
| ENSBTAG00000018131 | ENSBTAG00000033008 |  |  |
| ENSBTAG00000018134 | ENSBTAG00000033015 |  |  |
| ENSBTAG00000018138 | ENSBTAG00000033056 |  |  |
| ENSBTAG00000018146 | ENSBTAG00000033078 |  |  |
| ENSBTAG00000018152 | ENSBTAG00000033080 |  |  |
| ENSBTAG00000018153 | ENSBTAG00000033117 |  |  |
| ENSBTAG00000018156 | ENSBTAG00000033136 |  |  |
| ENSBTAG00000018158 | ENSBTAG00000033160 |  |  |
| ENSBTAG00000018159 | ENSBTAG00000033170 |  |  |
| ENSBTAG00000018160 | ENSBTAG00000033174 |  |  |
| ENSBTAG00000018161 | ENSBTAG00000033186 |  |  |
| ENSBTAG00000018164 | ENSBTAG00000033190 |  |  |
| ENSBTAG00000018167 | ENSBTAG00000033214 |  |  |
| ENSBTAG00000018176 | ENSBTAG00000033217 |  |  |
| ENSBTAG00000018178 | ENSBTAG00000033218 |  |  |
| ENSBTAG00000018179 | ENSBTAG00000033220 |  |  |
| ENSBTAG00000018186 | ENSBTAG00000033221 |  |  |
| ENSBTAG00000018189 | ENSBTAG00000033225 |  |  |
| ENSBTAG00000018192 | ENSBTAG00000033255 |  |  |
| ENSBTAG00000018196 | ENSBTAG00000033278 |  |  |
| ENSBTAG00000018202 | ENSBTAG00000033290 |  |  |
| ENSBTAG00000018205 | ENSBTAG00000033292 |  |  |
| ENSBTAG00000018206 | ENSBTAG00000033298 |  |  |
| ENSBTAG00000018207 | ENSBTAG00000033304 |  |  |
| ENSBTAG00000018213 | ENSBTAG00000033319 |  |  |
| ENSBTAG00000018214 | ENSBTAG00000033326 |  |  |
| ENSBTAG00000018216 | ENSBTAG00000033331 |  |  |
| ENSBTAG00000018218 | ENSBTAG00000033333 |  |  |
| ENSBTAG00000018223 | ENSBTAG00000033334 |  |  |
| ENSBTAG00000018227 | ENSBTAG00000033339 |  |  |
| ENSBTAG00000018236 | ENSBTAG00000033345 |  |  |
| ENSBTAG00000018238 | ENSBTAG00000033367 |  |  |
| ENSBTAG00000018239 | ENSBTAG00000033395 |  |  |
| ENSBTAG00000018240 | ENSBTAG00000033396 |  |  |
| ENSBTAG00000018245 | ENSBTAG00000033397 |  |  |
| ENSBTAG00000018247 | ENSBTAG00000033413 |  |  |
| ENSBTAG00000018252 | ENSBTAG00000033423 |  |  |
| ENSBTAG00000018253 | ENSBTAG00000033441 |  |  |
| ENSBTAG00000018254 | ENSBTAG00000033453 |  |  |
| ENSBTAG00000018256 | ENSBTAG00000033460 |  |  |
| ENSBTAG00000018257 | ENSBTAG00000033486 |  |  |
| ENSBTAG00000018258 | ENSBTAG00000033504 |  |  |
| ENSBTAG00000018261 | ENSBTAG00000033510 |  |  |
| ENSBTAG00000018262 | ENSBTAG00000033535 |  |  |
| ENSBTAG00000018263 | ENSBTAG00000033543 |  |  |
| ENSBTAG00000018267 | ENSBTAG00000033603 |  |  |
| ENSBTAG00000018271 | ENSBTAG00000033621 |  |  |
| ENSBTAG00000018280 | ENSBTAG00000033677 |  |  |
| ENSBTAG00000018281 | ENSBTAG00000033679 |  |  |
| ENSBTAG00000018282 | ENSBTAG00000033690 |  |  |
| ENSBTAG00000018283 | ENSBTAG00000033699 |  |  |
| ENSBTAG00000018285 | ENSBTAG00000033731 |  |  |
| ENSBTAG00000018286 | ENSBTAG00000033747 |  |  |
| ENSBTAG00000018287 | ENSBTAG00000033803 |  |  |
| ENSBTAG00000018289 | ENSBTAG00000033835 |  |  |
| ENSBTAG00000018291 | ENSBTAG00000033902 |  |  |
| ENSBTAG00000018292 | ENSBTAG00000033983 |  |  |
| ENSBTAG00000018295 | ENSBTAG00000034069 |  |  |
| ENSBTAG00000018297 | ENSBTAG00000034106 |  |  |
| ENSBTAG00000018299 | ENSBTAG00000034139 |  |  |
| ENSBTAG00000018300 | ENSBTAG00000034147 |  |  |
| ENSBTAG00000018313 | ENSBTAG00000034154 |  |  |
| ENSBTAG00000018314 | ENSBTAG00000034184 |  |  |
| ENSBTAG00000018317 | ENSBTAG00000034185 |  |  |
| ENSBTAG00000018318 | ENSBTAG00000034192 |  |  |
| ENSBTAG00000018321 | ENSBTAG00000034255 |  |  |
| ENSBTAG00000018322 | ENSBTAG00000034295 |  |  |
| ENSBTAG00000018330 | ENSBTAG00000034347 |  |  |
| ENSBTAG00000018331 | ENSBTAG00000034366 |  |  |
| ENSBTAG00000018339 | ENSBTAG00000034384 |  |  |
| ENSBTAG00000018342 | ENSBTAG00000034436 |  |  |
| ENSBTAG00000018345 | ENSBTAG00000034441 |  |  |
| ENSBTAG00000018347 | ENSBTAG00000034442 |  |  |
| ENSBTAG00000018348 | ENSBTAG00000034495 |  |  |
| ENSBTAG00000018349 | ENSBTAG00000034529 |  |  |
| ENSBTAG00000018352 | ENSBTAG00000034531 |  |  |
| ENSBTAG00000018358 | ENSBTAG00000034598 |  |  |
| ENSBTAG00000018360 | ENSBTAG00000034633 |  |  |
| ENSBTAG00000018361 | ENSBTAG00000034659 |  |  |
| ENSBTAG00000018362 | ENSBTAG00000034676 |  |  |
| ENSBTAG00000018363 | ENSBTAG00000034691 |  |  |
| ENSBTAG00000018364 | ENSBTAG00000034776 |  |  |
| ENSBTAG00000018365 | ENSBTAG00000034823 |  |  |
| ENSBTAG00000018369 | ENSBTAG00000034827 |  |  |
| ENSBTAG00000018372 | ENSBTAG00000034850 |  |  |
| ENSBTAG00000018373 | ENSBTAG00000034869 |  |  |
| ENSBTAG00000018374 | ENSBTAG00000034875 |  |  |
| ENSBTAG00000018375 | ENSBTAG00000034883 |  |  |
| ENSBTAG00000018381 | ENSBTAG00000034885 |  |  |
| ENSBTAG00000018386 | ENSBTAG00000034905 |  |  |
| ENSBTAG00000018387 | ENSBTAG00000034936 |  |  |
| ENSBTAG00000018391 | ENSBTAG00000034939 |  |  |
| ENSBTAG00000018400 | ENSBTAG00000034952 |  |  |
| ENSBTAG00000018401 | ENSBTAG00000034978 |  |  |
| ENSBTAG00000018402 | ENSBTAG00000035012 |  |  |
| ENSBTAG00000018406 | ENSBTAG00000035054 |  |  |
| ENSBTAG00000018413 | ENSBTAG00000035064 |  |  |
| ENSBTAG00000018415 | ENSBTAG00000035083 |  |  |
| ENSBTAG00000018416 | ENSBTAG00000035129 |  |  |
| ENSBTAG00000018417 | ENSBTAG00000035144 |  |  |
| ENSBTAG00000018421 | ENSBTAG00000035174 |  |  |
| ENSBTAG00000018422 | ENSBTAG00000035175 |  |  |
| ENSBTAG00000018423 | ENSBTAG00000035226 |  |  |
| ENSBTAG00000018424 | ENSBTAG00000035244 |  |  |
| ENSBTAG00000018425 | ENSBTAG00000035247 |  |  |
| ENSBTAG00000018426 | ENSBTAG00000035265 |  |  |
| ENSBTAG00000018427 | ENSBTAG00000035286 |  |  |
| ENSBTAG00000018430 | ENSBTAG00000035319 |  |  |
| ENSBTAG00000018432 | ENSBTAG00000035323 |  |  |
| ENSBTAG00000018436 | ENSBTAG00000035556 |  |  |
| ENSBTAG00000018446 | ENSBTAG00000035643 |  |  |
| ENSBTAG00000018449 | ENSBTAG00000035785 |  |  |
| ENSBTAG00000018451 | ENSBTAG00000035844 |  |  |
| ENSBTAG00000018452 | ENSBTAG00000035858 |  |  |
| ENSBTAG00000018456 | ENSBTAG00000035868 |  |  |
| ENSBTAG00000018463 | ENSBTAG00000035907 |  |  |
| ENSBTAG00000018464 | ENSBTAG00000036009 |  |  |
| ENSBTAG00000018465 | ENSBTAG00000036222 |  |  |
| ENSBTAG00000018467 | ENSBTAG00000036260 |  |  |
| ENSBTAG00000018474 | ENSBTAG00000036262 |  |  |
| ENSBTAG00000018479 | ENSBTAG00000036297 |  |  |
| ENSBTAG00000018481 | ENSBTAG00000036298 |  |  |
| ENSBTAG00000018482 | ENSBTAG00000037375 |  |  |
| ENSBTAG00000018483 | ENSBTAG00000037389 |  |  |
| ENSBTAG00000018488 | ENSBTAG00000037465 |  |  |
| ENSBTAG00000018489 | ENSBTAG00000037470 |  |  |
| ENSBTAG00000018493 | ENSBTAG00000037483 |  |  |
| ENSBTAG00000018496 | ENSBTAG00000037489 |  |  |
| ENSBTAG00000018497 | ENSBTAG00000037533 |  |  |
| ENSBTAG00000018499 | ENSBTAG00000037539 |  |  |
| ENSBTAG00000018501 | ENSBTAG00000037559 |  |  |
| ENSBTAG00000018513 | ENSBTAG00000037566 |  |  |
| ENSBTAG00000018517 | ENSBTAG00000037571 |  |  |
| ENSBTAG00000018522 | ENSBTAG00000037602 |  |  |
| ENSBTAG00000018527 | ENSBTAG00000037605 |  |  |
| ENSBTAG00000018534 | ENSBTAG00000037640 |  |  |
| ENSBTAG00000018538 | ENSBTAG00000037673 |  |  |
| ENSBTAG00000018540 | ENSBTAG00000037686 |  |  |
| ENSBTAG00000018548 | ENSBTAG00000037687 |  |  |
| ENSBTAG00000018555 | ENSBTAG00000037702 |  |  |
| ENSBTAG00000018557 | ENSBTAG00000037703 |  |  |
| ENSBTAG00000018562 | ENSBTAG00000037728 |  |  |
| ENSBTAG00000018563 | ENSBTAG00000037729 |  |  |
| ENSBTAG00000018564 | ENSBTAG00000037735 |  |  |
| ENSBTAG00000018566 | ENSBTAG00000037765 |  |  |
| ENSBTAG00000018569 | ENSBTAG00000037778 |  |  |
| ENSBTAG00000018572 | ENSBTAG00000037791 |  |  |
| ENSBTAG00000018576 | ENSBTAG00000037795 |  |  |
| ENSBTAG00000018578 | ENSBTAG00000037799 |  |  |
| ENSBTAG00000018579 | ENSBTAG00000037800 |  |  |
| ENSBTAG00000018581 | ENSBTAG00000037804 |  |  |
| ENSBTAG00000018588 | ENSBTAG00000037813 |  |  |
| ENSBTAG00000018589 | ENSBTAG00000037819 |  |  |
| ENSBTAG00000018598 | ENSBTAG00000037826 |  |  |
| ENSBTAG00000018600 | ENSBTAG00000037844 |  |  |
| ENSBTAG00000018601 | ENSBTAG00000037856 |  |  |
| ENSBTAG00000018604 | ENSBTAG00000037964 |  |  |
| ENSBTAG00000018607 | ENSBTAG00000037972 |  |  |
| ENSBTAG00000018610 | ENSBTAG00000037980 |  |  |
| ENSBTAG00000018613 | ENSBTAG00000037986 |  |  |
| ENSBTAG00000018616 | ENSBTAG00000037988 |  |  |
| ENSBTAG00000018622 | ENSBTAG00000037989 |  |  |
| ENSBTAG00000018625 | ENSBTAG00000037991 |  |  |
| ENSBTAG00000018631 | ENSBTAG00000037996 |  |  |
| ENSBTAG00000018633 | ENSBTAG00000038025 |  |  |
| ENSBTAG00000018637 | ENSBTAG00000038033 |  |  |
| ENSBTAG00000018639 | ENSBTAG00000038047 |  |  |
| ENSBTAG00000018642 | ENSBTAG00000038050 |  |  |
| ENSBTAG00000018645 | ENSBTAG00000038058 |  |  |
| ENSBTAG00000018646 | ENSBTAG00000038062 |  |  |
| ENSBTAG00000018647 | ENSBTAG00000038079 |  |  |
| ENSBTAG00000018650 | ENSBTAG00000038085 |  |  |
| ENSBTAG00000018652 | ENSBTAG00000038093 |  |  |
| ENSBTAG00000018653 | ENSBTAG00000038104 |  |  |
| ENSBTAG00000018655 | ENSBTAG00000038112 |  |  |
| ENSBTAG00000018656 | ENSBTAG00000038115 |  |  |
| ENSBTAG00000018658 | ENSBTAG00000038116 |  |  |
| ENSBTAG00000018661 | ENSBTAG00000038117 |  |  |
| ENSBTAG00000018663 | ENSBTAG00000038131 |  |  |
| ENSBTAG00000018667 | ENSBTAG00000038132 |  |  |
| ENSBTAG00000018669 | ENSBTAG00000038149 |  |  |
| ENSBTAG00000018671 | ENSBTAG00000038151 |  |  |
| ENSBTAG00000018688 | ENSBTAG00000038156 |  |  |
| ENSBTAG00000018690 | ENSBTAG00000038173 |  |  |
| ENSBTAG00000018691 | ENSBTAG00000038180 |  |  |
| ENSBTAG00000018694 | ENSBTAG00000038186 |  |  |
| ENSBTAG00000018697 | ENSBTAG00000038189 |  |  |
| ENSBTAG00000018699 | ENSBTAG00000038195 |  |  |
| ENSBTAG00000018703 | ENSBTAG00000038221 |  |  |
| ENSBTAG00000018704 | ENSBTAG00000038228 |  |  |
| ENSBTAG00000018705 | ENSBTAG00000038235 |  |  |
| ENSBTAG00000018706 | ENSBTAG00000038238 |  |  |
| ENSBTAG00000018707 | ENSBTAG00000038263 |  |  |
| ENSBTAG00000018708 | ENSBTAG00000038286 |  |  |
| ENSBTAG00000018711 | ENSBTAG00000038291 |  |  |
| ENSBTAG00000018715 | ENSBTAG00000038312 |  |  |
| ENSBTAG00000018717 | ENSBTAG00000038325 |  |  |
| ENSBTAG00000018722 | ENSBTAG00000038333 |  |  |
| ENSBTAG00000018723 | ENSBTAG00000038340 |  |  |
| ENSBTAG00000018725 | ENSBTAG00000038347 |  |  |
| ENSBTAG00000018729 | ENSBTAG00000038375 |  |  |
| ENSBTAG00000018732 | ENSBTAG00000038381 |  |  |
| ENSBTAG00000018735 | ENSBTAG00000038384 |  |  |
| ENSBTAG00000018737 | ENSBTAG00000038386 |  |  |
| ENSBTAG00000018739 | ENSBTAG00000038415 |  |  |
| ENSBTAG00000018742 | ENSBTAG00000038461 |  |  |
| ENSBTAG00000018743 | ENSBTAG00000038462 |  |  |
| ENSBTAG00000018745 | ENSBTAG00000038464 |  |  |
| ENSBTAG00000018746 | ENSBTAG00000038477 |  |  |
| ENSBTAG00000018749 | ENSBTAG00000038480 |  |  |
| ENSBTAG00000018753 | ENSBTAG00000038488 |  |  |
| ENSBTAG00000018756 | ENSBTAG00000038495 |  |  |
| ENSBTAG00000018761 | ENSBTAG00000038496 |  |  |
| ENSBTAG00000018765 | ENSBTAG00000038497 |  |  |
| ENSBTAG00000018768 | ENSBTAG00000038536 |  |  |
| ENSBTAG00000018770 | ENSBTAG00000038540 |  |  |
| ENSBTAG00000018771 | ENSBTAG00000038577 |  |  |
| ENSBTAG00000018772 | ENSBTAG00000038595 |  |  |
| ENSBTAG00000018773 | ENSBTAG00000038619 |  |  |
| ENSBTAG00000018774 | ENSBTAG00000038635 |  |  |
| ENSBTAG00000018775 | ENSBTAG00000038640 |  |  |
| ENSBTAG00000018783 | ENSBTAG00000038652 |  |  |
| ENSBTAG00000018784 | ENSBTAG00000038660 |  |  |
| ENSBTAG00000018796 | ENSBTAG00000038662 |  |  |
| ENSBTAG00000018802 | ENSBTAG00000038674 |  |  |
| ENSBTAG00000018803 | ENSBTAG00000038696 |  |  |
| ENSBTAG00000018804 | ENSBTAG00000038698 |  |  |
| ENSBTAG00000018805 | ENSBTAG00000038700 |  |  |
| ENSBTAG00000018806 | ENSBTAG00000038706 |  |  |
| ENSBTAG00000018808 | ENSBTAG00000038737 |  |  |
| ENSBTAG00000018809 | ENSBTAG00000038738 |  |  |
| ENSBTAG00000018813 | ENSBTAG00000038783 |  |  |
| ENSBTAG00000018824 | ENSBTAG00000038795 |  |  |
| ENSBTAG00000018829 | ENSBTAG00000038806 |  |  |
| ENSBTAG00000018833 | ENSBTAG00000038810 |  |  |
| ENSBTAG00000018834 | ENSBTAG00000038815 |  |  |
| ENSBTAG00000018837 | ENSBTAG00000038865 |  |  |
| ENSBTAG00000018841 | ENSBTAG00000038866 |  |  |
| ENSBTAG00000018848 | ENSBTAG00000038869 |  |  |
| ENSBTAG00000018851 | ENSBTAG00000038878 |  |  |
| ENSBTAG00000018852 | ENSBTAG00000038888 |  |  |
| ENSBTAG00000018854 | ENSBTAG00000038893 |  |  |
| ENSBTAG00000018857 | ENSBTAG00000038896 |  |  |
| ENSBTAG00000018859 | ENSBTAG00000038904 |  |  |
| ENSBTAG00000018863 | ENSBTAG00000038910 |  |  |
| ENSBTAG00000018868 | ENSBTAG00000038929 |  |  |
| ENSBTAG00000018872 | ENSBTAG00000038931 |  |  |
| ENSBTAG00000018873 | ENSBTAG00000038949 |  |  |
| ENSBTAG00000018879 | ENSBTAG00000038955 |  |  |
| ENSBTAG00000018880 | ENSBTAG00000038966 |  |  |
| ENSBTAG00000018882 | ENSBTAG00000038974 |  |  |
| ENSBTAG00000018883 | ENSBTAG00000038979 |  |  |
| ENSBTAG00000018887 | ENSBTAG00000039015 |  |  |
| ENSBTAG00000018888 | ENSBTAG00000039037 |  |  |
| ENSBTAG00000018889 | ENSBTAG00000039046 |  |  |
| ENSBTAG00000018893 | ENSBTAG00000039050 |  |  |
| ENSBTAG00000018894 | ENSBTAG00000039055 |  |  |
| ENSBTAG00000018897 | ENSBTAG00000039068 |  |  |
| ENSBTAG00000018905 | ENSBTAG00000039071 |  |  |
| ENSBTAG00000018908 | ENSBTAG00000039077 |  |  |
| ENSBTAG00000018910 | ENSBTAG00000039090 |  |  |
| ENSBTAG00000018914 | ENSBTAG00000039105 |  |  |
| ENSBTAG00000018920 | ENSBTAG00000039129 |  |  |
| ENSBTAG00000018921 | ENSBTAG00000039153 |  |  |
| ENSBTAG00000018924 | ENSBTAG00000039161 |  |  |
| ENSBTAG00000018925 | ENSBTAG00000039163 |  |  |
| ENSBTAG00000018935 | ENSBTAG00000039172 |  |  |
| ENSBTAG00000018936 | ENSBTAG00000039212 |  |  |
| ENSBTAG00000018937 | ENSBTAG00000039231 |  |  |
| ENSBTAG00000018938 | ENSBTAG00000039246 |  |  |
| ENSBTAG00000018941 | ENSBTAG00000039275 |  |  |
| ENSBTAG00000018942 | ENSBTAG00000039287 |  |  |
| ENSBTAG00000018945 | ENSBTAG00000039307 |  |  |
| ENSBTAG00000018948 | ENSBTAG00000039313 |  |  |
| ENSBTAG00000018966 | ENSBTAG00000039325 |  |  |
| ENSBTAG00000018969 | ENSBTAG00000039326 |  |  |
| ENSBTAG00000018971 | ENSBTAG00000039335 |  |  |
| ENSBTAG00000018973 | ENSBTAG00000039340 |  |  |
| ENSBTAG00000018975 | ENSBTAG00000039355 |  |  |
| ENSBTAG00000018979 | ENSBTAG00000039362 |  |  |
| ENSBTAG00000018984 | ENSBTAG00000039366 |  |  |
| ENSBTAG00000018989 | ENSBTAG00000039374 |  |  |
| ENSBTAG00000018991 | ENSBTAG00000039384 |  |  |
| ENSBTAG00000018993 | ENSBTAG00000039391 |  |  |
| ENSBTAG00000018996 | ENSBTAG00000039415 |  |  |
| ENSBTAG00000018999 | ENSBTAG00000039425 |  |  |
| ENSBTAG00000019002 | ENSBTAG00000039435 |  |  |
| ENSBTAG00000019008 | ENSBTAG00000039462 |  |  |
| ENSBTAG00000019011 | ENSBTAG00000039483 |  |  |
| ENSBTAG00000019013 | ENSBTAG00000039493 |  |  |
| ENSBTAG00000019014 | ENSBTAG00000039509 |  |  |
| ENSBTAG00000019017 | ENSBTAG00000039523 |  |  |
| ENSBTAG00000019020 | ENSBTAG00000039529 |  |  |
| ENSBTAG00000019023 | ENSBTAG00000039540 |  |  |
| ENSBTAG00000019024 | ENSBTAG00000039552 |  |  |
| ENSBTAG00000019026 | ENSBTAG00000039571 |  |  |
| ENSBTAG00000019027 | ENSBTAG00000039573 |  |  |
| ENSBTAG00000019029 | ENSBTAG00000039574 |  |  |
| ENSBTAG00000019037 | ENSBTAG00000039591 |  |  |
| ENSBTAG00000019040 | ENSBTAG00000039597 |  |  |
| ENSBTAG00000019044 | ENSBTAG00000039599 |  |  |
| ENSBTAG00000019045 | ENSBTAG00000039602 |  |  |
| ENSBTAG00000019051 | ENSBTAG00000039618 |  |  |
| ENSBTAG00000019052 | ENSBTAG00000039620 |  |  |
| ENSBTAG00000019060 | ENSBTAG00000039634 |  |  |
| ENSBTAG00000019061 | ENSBTAG00000039635 |  |  |
| ENSBTAG00000019062 | ENSBTAG00000039657 |  |  |
| ENSBTAG00000019065 | ENSBTAG00000039658 |  |  |
| ENSBTAG00000019066 | ENSBTAG00000039684 |  |  |
| ENSBTAG00000019067 | ENSBTAG00000039686 |  |  |
| ENSBTAG00000019069 | ENSBTAG00000039688 |  |  |
| ENSBTAG00000019070 | ENSBTAG00000039695 |  |  |
| ENSBTAG00000019071 | ENSBTAG00000039705 |  |  |
| ENSBTAG00000019073 | ENSBTAG00000039714 |  |  |
| ENSBTAG00000019075 | ENSBTAG00000039718 |  |  |
| ENSBTAG00000019076 | ENSBTAG00000039727 |  |  |
| ENSBTAG00000019079 | ENSBTAG00000039731 |  |  |
| ENSBTAG00000019080 | ENSBTAG00000039732 |  |  |
| ENSBTAG00000019081 | ENSBTAG00000039740 |  |  |
| ENSBTAG00000019082 | ENSBTAG00000039794 |  |  |
| ENSBTAG00000019090 | ENSBTAG00000039803 |  |  |
| ENSBTAG00000019092 | ENSBTAG00000039812 |  |  |
| ENSBTAG00000019094 | ENSBTAG00000039815 |  |  |
| ENSBTAG00000019097 | ENSBTAG00000039819 |  |  |
| ENSBTAG00000019105 | ENSBTAG00000039820 |  |  |
| ENSBTAG00000019107 | ENSBTAG00000039839 |  |  |
| ENSBTAG00000019115 | ENSBTAG00000039851 |  |  |
| ENSBTAG00000019116 | ENSBTAG00000039855 |  |  |
| ENSBTAG00000019124 | ENSBTAG00000039891 |  |  |
| ENSBTAG00000019125 | ENSBTAG00000039916 |  |  |
| ENSBTAG00000019129 | ENSBTAG00000039928 |  |  |
| ENSBTAG00000019130 | ENSBTAG00000039950 |  |  |
| ENSBTAG00000019131 | ENSBTAG00000039951 |  |  |
| ENSBTAG00000019132 | ENSBTAG00000039958 |  |  |
| ENSBTAG00000019136 | ENSBTAG00000039968 |  |  |
| ENSBTAG00000019138 | ENSBTAG00000039991 |  |  |
| ENSBTAG00000019139 | ENSBTAG00000040005 |  |  |
| ENSBTAG00000019140 | ENSBTAG00000040006 |  |  |
| ENSBTAG00000019143 | ENSBTAG00000040028 |  |  |
| ENSBTAG00000019146 | ENSBTAG00000040031 |  |  |
| ENSBTAG00000019150 | ENSBTAG00000040034 |  |  |
| ENSBTAG00000019153 | ENSBTAG00000040043 |  |  |
| ENSBTAG00000019157 | ENSBTAG00000040055 |  |  |
| ENSBTAG00000019160 | ENSBTAG00000040058 |  |  |
| ENSBTAG00000019163 | ENSBTAG00000040065 |  |  |
| ENSBTAG00000019164 | ENSBTAG00000040082 |  |  |
| ENSBTAG00000019166 | ENSBTAG00000040131 |  |  |
| ENSBTAG00000019167 | ENSBTAG00000040169 |  |  |
| ENSBTAG00000019174 | ENSBTAG00000040188 |  |  |
| ENSBTAG00000019175 | ENSBTAG00000040193 |  |  |
| ENSBTAG00000019177 | ENSBTAG00000040199 |  |  |
| ENSBTAG00000019180 | ENSBTAG00000040202 |  |  |
| ENSBTAG00000019181 | ENSBTAG00000040244 |  |  |
| ENSBTAG00000019182 | ENSBTAG00000040295 |  |  |
| ENSBTAG00000019183 | ENSBTAG00000040296 |  |  |
| ENSBTAG00000019184 | ENSBTAG00000040321 |  |  |
| ENSBTAG00000019187 | ENSBTAG00000040323 |  |  |
| ENSBTAG00000019192 | ENSBTAG00000040333 |  |  |
| ENSBTAG00000019194 | ENSBTAG00000040336 |  |  |
| ENSBTAG00000019197 | ENSBTAG00000040340 |  |  |
| ENSBTAG00000019202 | ENSBTAG00000040361 |  |  |
| ENSBTAG00000019209 | ENSBTAG00000040367 |  |  |
| ENSBTAG00000019210 | ENSBTAG00000040368 |  |  |
| ENSBTAG00000019211 | ENSBTAG00000040381 |  |  |
| ENSBTAG00000019213 | ENSBTAG00000040392 |  |  |
| ENSBTAG00000019214 | ENSBTAG00000040422 |  |  |
| ENSBTAG00000019215 | ENSBTAG00000040432 |  |  |
| ENSBTAG00000019216 | ENSBTAG00000040435 |  |  |
| ENSBTAG00000019218 | ENSBTAG00000040460 |  |  |
| ENSBTAG00000019220 | ENSBTAG00000040490 |  |  |
| ENSBTAG00000019225 | ENSBTAG00000040512 |  |  |
| ENSBTAG00000019226 | ENSBTAG00000040551 |  |  |
| ENSBTAG00000019229 | ENSBTAG00000040569 |  |  |
| ENSBTAG00000019234 | ENSBTAG00000040575 |  |  |
| ENSBTAG00000019235 | ENSBTAG00000040580 |  |  |
| ENSBTAG00000019238 | ENSBTAG00000040586 |  |  |
| ENSBTAG00000019241 | ENSBTAG00000040598 |  |  |
| ENSBTAG00000019242 | ENSBTAG00000040607 |  |  |
| ENSBTAG00000019244 | ENSBTAG00000043553 |  |  |
| ENSBTAG00000019246 | ENSBTAG00000043581 |  |  |
| ENSBTAG00000019250 | ENSBTAG00000043951 |  |  |
| ENSBTAG00000019251 | ENSBTAG00000043954 |  |  |
| ENSBTAG00000019255 | ENSBTAG00000043961 |  |  |
| ENSBTAG00000019256 | ENSBTAG00000043972 |  |  |
| ENSBTAG00000019262 | ENSBTAG00000043981 |  |  |
| ENSBTAG00000019265 | ENSBTAG00000043993 |  |  |
| ENSBTAG00000019267 | ENSBTAG00000043994 |  |  |
| ENSBTAG00000019269 | ENSBTAG00000043996 |  |  |
| ENSBTAG00000019271 | ENSBTAG00000043999 |  |  |
| ENSBTAG00000019272 | ENSBTAG00000044007 |  |  |
| ENSBTAG00000019273 | ENSBTAG00000044015 |  |  |
| ENSBTAG00000019275 | ENSBTAG00000044018 |  |  |
| ENSBTAG00000019277 | ENSBTAG00000044029 |  |  |
| ENSBTAG00000019280 | ENSBTAG00000044043 |  |  |
| ENSBTAG00000019282 | ENSBTAG00000044053 |  |  |
| ENSBTAG00000019285 | ENSBTAG00000044056 |  |  |
| ENSBTAG00000019287 | ENSBTAG00000044058 |  |  |
| ENSBTAG00000019290 | ENSBTAG00000044063 |  |  |
| ENSBTAG00000019291 | ENSBTAG00000044075 |  |  |
| ENSBTAG00000019297 | ENSBTAG00000044092 |  |  |
| ENSBTAG00000019298 | ENSBTAG00000044097 |  |  |
| ENSBTAG00000019300 | ENSBTAG00000044099 |  |  |
| ENSBTAG00000019309 | ENSBTAG00000044100 |  |  |
| ENSBTAG00000019312 | ENSBTAG00000044106 |  |  |
| ENSBTAG00000019314 | ENSBTAG00000044123 |  |  |
| ENSBTAG00000019315 | ENSBTAG00000044129 |  |  |
| ENSBTAG00000019317 | ENSBTAG00000044135 |  |  |
| ENSBTAG00000019322 | ENSBTAG00000044150 |  |  |
| ENSBTAG00000019334 | ENSBTAG00000044169 |  |  |
| ENSBTAG00000019336 | ENSBTAG00000044171 |  |  |
| ENSBTAG00000019343 | ENSBTAG00000044175 |  |  |
| ENSBTAG00000019345 | ENSBTAG00000044178 |  |  |
| ENSBTAG00000019347 | ENSBTAG00000044184 |  |  |
| ENSBTAG00000019349 | ENSBTAG00000044185 |  |  |
| ENSBTAG00000019353 | ENSBTAG00000044194 |  |  |
| ENSBTAG00000019357 | ENSBTAG00000044202 |  |  |
| ENSBTAG00000019358 | ENSBTAG00000045510 |  |  |
| ENSBTAG00000019365 | ENSBTAG00000045520 |  |  |
| ENSBTAG00000019368 | ENSBTAG00000045538 |  |  |
| ENSBTAG00000019369 | ENSBTAG00000045548 |  |  |
| ENSBTAG00000019371 | ENSBTAG00000045550 |  |  |
| ENSBTAG00000019373 | ENSBTAG00000045566 |  |  |
| ENSBTAG00000019376 | ENSBTAG00000045578 |  |  |
| ENSBTAG00000019379 | ENSBTAG00000045582 |  |  |
| ENSBTAG00000019382 | ENSBTAG00000045588 |  |  |
| ENSBTAG00000019387 | ENSBTAG00000045592 |  |  |
| ENSBTAG00000019388 | ENSBTAG00000045604 |  |  |
| ENSBTAG00000019402 | ENSBTAG00000045628 |  |  |
| ENSBTAG00000019404 | ENSBTAG00000045649 |  |  |
| ENSBTAG00000019406 | ENSBTAG00000045653 |  |  |
| ENSBTAG00000019409 | ENSBTAG00000045678 |  |  |
| ENSBTAG00000019412 | ENSBTAG00000045697 |  |  |
| ENSBTAG00000019416 | ENSBTAG00000045704 |  |  |
| ENSBTAG00000019417 | ENSBTAG00000045728 |  |  |
| ENSBTAG00000019418 | ENSBTAG00000045742 |  |  |
| ENSBTAG00000019419 | ENSBTAG00000045744 |  |  |
| ENSBTAG00000019423 | ENSBTAG00000045748 |  |  |
| ENSBTAG00000019425 | ENSBTAG00000045750 |  |  |
| ENSBTAG00000019428 | ENSBTAG00000045757 |  |  |
| ENSBTAG00000019434 | ENSBTAG00000045762 |  |  |
| ENSBTAG00000019436 | ENSBTAG00000045773 |  |  |
| ENSBTAG00000019443 | ENSBTAG00000045779 |  |  |
| ENSBTAG00000019448 | ENSBTAG00000045785 |  |  |
| ENSBTAG00000019458 | ENSBTAG00000045794 |  |  |
| ENSBTAG00000019459 | ENSBTAG00000045805 |  |  |
| ENSBTAG00000019461 | ENSBTAG00000045828 |  |  |
| ENSBTAG00000019463 | ENSBTAG00000045834 |  |  |
| ENSBTAG00000019465 | ENSBTAG00000045896 |  |  |
| ENSBTAG00000019468 | ENSBTAG00000045902 |  |  |
| ENSBTAG00000019470 | ENSBTAG00000045929 |  |  |
| ENSBTAG00000019471 | ENSBTAG00000045931 |  |  |
| ENSBTAG00000019479 | ENSBTAG00000045943 |  |  |
| ENSBTAG00000019480 | ENSBTAG00000045967 |  |  |
| ENSBTAG00000019486 | ENSBTAG00000045969 |  |  |
| ENSBTAG00000019488 | ENSBTAG00000045971 |  |  |
| ENSBTAG00000019492 | ENSBTAG00000046014 |  |  |
| ENSBTAG00000019495 | ENSBTAG00000046019 |  |  |
| ENSBTAG00000019497 | ENSBTAG00000046031 |  |  |
| ENSBTAG00000019498 | ENSBTAG00000046037 |  |  |
| ENSBTAG00000019500 | ENSBTAG00000046046 |  |  |
| ENSBTAG00000019501 | ENSBTAG00000046054 |  |  |
| ENSBTAG00000019506 | ENSBTAG00000046073 |  |  |
| ENSBTAG00000019508 | ENSBTAG00000046075 |  |  |
| ENSBTAG00000019509 | ENSBTAG00000046080 |  |  |
| ENSBTAG00000019510 | ENSBTAG00000046084 |  |  |
| ENSBTAG00000019514 | ENSBTAG00000046089 |  |  |
| ENSBTAG00000019515 | ENSBTAG00000046117 |  |  |
| ENSBTAG00000019520 | ENSBTAG00000046121 |  |  |
| ENSBTAG00000019522 | ENSBTAG00000046160 |  |  |
| ENSBTAG00000019524 | ENSBTAG00000046172 |  |  |
| ENSBTAG00000019526 | ENSBTAG00000046218 |  |  |
| ENSBTAG00000019533 | ENSBTAG00000046248 |  |  |
| ENSBTAG00000019534 | ENSBTAG00000046250 |  |  |
| ENSBTAG00000019536 | ENSBTAG00000046257 |  |  |
| ENSBTAG00000019537 | ENSBTAG00000046264 |  |  |
| ENSBTAG00000019545 | ENSBTAG00000046273 |  |  |
| ENSBTAG00000019548 | ENSBTAG00000046282 |  |  |
| ENSBTAG00000019550 | ENSBTAG00000046303 |  |  |
| ENSBTAG00000019552 | ENSBTAG00000046308 |  |  |
| ENSBTAG00000019557 | ENSBTAG00000046321 |  |  |
| ENSBTAG00000019563 | ENSBTAG00000046339 |  |  |
| ENSBTAG00000019564 | ENSBTAG00000046385 |  |  |
| ENSBTAG00000019565 | ENSBTAG00000046389 |  |  |
| ENSBTAG00000019567 | ENSBTAG00000046391 |  |  |
| ENSBTAG00000019569 | ENSBTAG00000046415 |  |  |
| ENSBTAG00000019574 | ENSBTAG00000046450 |  |  |
| ENSBTAG00000019577 | ENSBTAG00000046456 |  |  |
| ENSBTAG00000019581 | ENSBTAG00000046478 |  |  |
| ENSBTAG00000019585 | ENSBTAG00000046484 |  |  |
| ENSBTAG00000019587 | ENSBTAG00000046486 |  |  |
| ENSBTAG00000019589 | ENSBTAG00000046493 |  |  |
| ENSBTAG00000019591 | ENSBTAG00000046500 |  |  |
| ENSBTAG00000019592 | ENSBTAG00000046511 |  |  |
| ENSBTAG00000019593 | ENSBTAG00000046520 |  |  |
| ENSBTAG00000019600 | ENSBTAG00000046533 |  |  |
| ENSBTAG00000019603 | ENSBTAG00000046542 |  |  |
| ENSBTAG00000019604 | ENSBTAG00000046544 |  |  |
| ENSBTAG00000019605 | ENSBTAG00000046545 |  |  |
| ENSBTAG00000019608 | ENSBTAG00000046561 |  |  |
| ENSBTAG00000019611 | ENSBTAG00000046580 |  |  |
| ENSBTAG00000019612 | ENSBTAG00000046584 |  |  |
| ENSBTAG00000019616 | ENSBTAG00000046586 |  |  |
| ENSBTAG00000019621 | ENSBTAG00000046588 |  |  |
| ENSBTAG00000019625 | ENSBTAG00000046607 |  |  |
| ENSBTAG00000019627 | ENSBTAG00000046612 |  |  |
| ENSBTAG00000019630 | ENSBTAG00000046628 |  |  |
| ENSBTAG00000019634 | ENSBTAG00000046644 |  |  |
| ENSBTAG00000019636 | ENSBTAG00000046670 |  |  |
| ENSBTAG00000019639 | ENSBTAG00000046724 |  |  |
| ENSBTAG00000019644 | ENSBTAG00000046730 |  |  |
| ENSBTAG00000019645 | ENSBTAG00000046746 |  |  |
| ENSBTAG00000019651 | ENSBTAG00000046750 |  |  |
| ENSBTAG00000019652 | ENSBTAG00000046773 |  |  |
| ENSBTAG00000019658 | ENSBTAG00000046797 |  |  |
| ENSBTAG00000019660 | ENSBTAG00000046840 |  |  |
| ENSBTAG00000019662 | ENSBTAG00000046846 |  |  |
| ENSBTAG00000019663 | ENSBTAG00000046886 |  |  |
| ENSBTAG00000019665 | ENSBTAG00000046918 |  |  |
| ENSBTAG00000019672 | ENSBTAG00000046919 |  |  |
| ENSBTAG00000019675 | ENSBTAG00000046936 |  |  |
| ENSBTAG00000019676 | ENSBTAG00000046939 |  |  |
| ENSBTAG00000019680 | ENSBTAG00000046959 |  |  |
| ENSBTAG00000019684 | ENSBTAG00000046977 |  |  |
| ENSBTAG00000019685 | ENSBTAG00000046979 |  |  |
| ENSBTAG00000019686 | ENSBTAG00000046981 |  |  |
| ENSBTAG00000019692 | ENSBTAG00000046996 |  |  |
| ENSBTAG00000019694 | ENSBTAG00000047009 |  |  |
| ENSBTAG00000019696 | ENSBTAG00000047027 |  |  |
| ENSBTAG00000019700 | ENSBTAG00000047054 |  |  |
| ENSBTAG00000019703 | ENSBTAG00000047085 |  |  |
| ENSBTAG00000019704 | ENSBTAG00000047088 |  |  |
| ENSBTAG00000019707 | ENSBTAG00000047139 |  |  |
| ENSBTAG00000019708 | ENSBTAG00000047148 |  |  |
| ENSBTAG00000019712 | ENSBTAG00000047168 |  |  |
| ENSBTAG00000019716 | ENSBTAG00000047169 |  |  |
| ENSBTAG00000019718 | ENSBTAG00000047202 |  |  |
| ENSBTAG00000019721 | ENSBTAG00000047203 |  |  |
| ENSBTAG00000019722 | ENSBTAG00000047225 |  |  |
| ENSBTAG00000019723 | ENSBTAG00000047249 |  |  |
| ENSBTAG00000019729 | ENSBTAG00000047278 |  |  |
| ENSBTAG00000019733 | ENSBTAG00000047287 |  |  |
| ENSBTAG00000019741 | ENSBTAG00000047303 |  |  |
| ENSBTAG00000019742 | ENSBTAG00000047314 |  |  |
| ENSBTAG00000019743 | ENSBTAG00000047330 |  |  |
| ENSBTAG00000019750 | ENSBTAG00000047331 |  |  |
| ENSBTAG00000019752 | ENSBTAG00000047339 |  |  |
| ENSBTAG00000019754 | ENSBTAG00000047347 |  |  |
| ENSBTAG00000019755 | ENSBTAG00000047406 |  |  |
| ENSBTAG00000019761 | ENSBTAG00000047412 |  |  |
| ENSBTAG00000019767 | ENSBTAG00000047418 |  |  |
| ENSBTAG00000019770 | ENSBTAG00000047434 |  |  |
| ENSBTAG00000019772 | ENSBTAG00000047448 |  |  |
| ENSBTAG00000019777 | ENSBTAG00000047450 |  |  |
| ENSBTAG00000019779 | ENSBTAG00000047491 |  |  |
| ENSBTAG00000019780 | ENSBTAG00000047493 |  |  |
| ENSBTAG00000019781 | ENSBTAG00000047499 |  |  |
| ENSBTAG00000019782 | ENSBTAG00000047502 |  |  |
| ENSBTAG00000019783 | ENSBTAG00000047510 |  |  |
| ENSBTAG00000019794 | ENSBTAG00000047520 |  |  |
| ENSBTAG00000019798 | ENSBTAG00000047531 |  |  |
| ENSBTAG00000019803 | ENSBTAG00000047551 |  |  |
| ENSBTAG00000019807 | ENSBTAG00000047586 |  |  |
| ENSBTAG00000019808 | ENSBTAG00000047591 |  |  |
| ENSBTAG00000019810 | ENSBTAG00000047595 |  |  |
| ENSBTAG00000019811 | ENSBTAG00000047599 |  |  |
| ENSBTAG00000019812 | ENSBTAG00000047605 |  |  |
| ENSBTAG00000019813 | ENSBTAG00000047606 |  |  |
| ENSBTAG00000019818 | ENSBTAG00000047628 |  |  |
| ENSBTAG00000019819 | ENSBTAG00000047635 |  |  |
| ENSBTAG00000019822 | ENSBTAG00000047638 |  |  |
| ENSBTAG00000019830 | ENSBTAG00000047658 |  |  |
| ENSBTAG00000019831 | ENSBTAG00000047690 |  |  |
| ENSBTAG00000019832 | ENSBTAG00000047694 |  |  |
| ENSBTAG00000019836 | ENSBTAG00000047706 |  |  |
| ENSBTAG00000019838 | ENSBTAG00000047708 |  |  |
| ENSBTAG00000019841 | ENSBTAG00000047719 |  |  |
| ENSBTAG00000019845 | ENSBTAG00000047739 |  |  |
| ENSBTAG00000019846 | ENSBTAG00000047756 |  |  |
| ENSBTAG00000019848 | ENSBTAG00000047760 |  |  |
| ENSBTAG00000019851 | ENSBTAG00000047766 |  |  |
| ENSBTAG00000019852 | ENSBTAG00000047780 |  |  |
| ENSBTAG00000019853 | ENSBTAG00000047781 |  |  |
| ENSBTAG00000019854 | ENSBTAG00000047786 |  |  |
| ENSBTAG00000019855 | ENSBTAG00000047806 |  |  |
| ENSBTAG00000019857 | ENSBTAG00000047837 |  |  |
| ENSBTAG00000019859 | ENSBTAG00000047857 |  |  |
| ENSBTAG00000019864 | ENSBTAG00000047876 |  |  |
| ENSBTAG00000019866 | ENSBTAG00000047880 |  |  |
| ENSBTAG00000019869 | ENSBTAG00000047909 |  |  |
| ENSBTAG00000019870 | ENSBTAG00000047919 |  |  |
| ENSBTAG00000019872 | ENSBTAG00000047944 |  |  |
| ENSBTAG00000019876 | ENSBTAG00000047958 |  |  |
| ENSBTAG00000019877 | ENSBTAG00000047991 |  |  |
| ENSBTAG00000019883 | ENSBTAG00000047998 |  |  |
| ENSBTAG00000019887 | ENSBTAG00000048053 |  |  |
| ENSBTAG00000019888 | ENSBTAG00000048071 |  |  |
| ENSBTAG00000019889 | ENSBTAG00000048077 |  |  |
| ENSBTAG00000019891 | ENSBTAG00000048098 |  |  |
| ENSBTAG00000019892 | ENSBTAG00000048107 |  |  |
| ENSBTAG00000019903 | ENSBTAG00000048125 |  |  |
| ENSBTAG00000019906 | ENSBTAG00000048151 |  |  |
| ENSBTAG00000019907 | ENSBTAG00000048155 |  |  |
| ENSBTAG00000019909 | ENSBTAG00000048157 |  |  |
| ENSBTAG00000019910 | ENSBTAG00000048173 |  |  |
| ENSBTAG00000019911 | ENSBTAG00000048184 |  |  |
| ENSBTAG00000019914 | ENSBTAG00000048195 |  |  |
| ENSBTAG00000019915 | ENSBTAG00000048225 |  |  |
| ENSBTAG00000019924 | ENSBTAG00000048237 |  |  |
| ENSBTAG00000019926 | ENSBTAG00000048273 |  |  |
| ENSBTAG00000019927 | ENSBTAG00000048286 |  |  |
| ENSBTAG00000019930 | ENSBTAG00000048296 |  |  |
| ENSBTAG00000019933 |  |  |  |
| ENSBTAG00000019937 |  |  |  |
| ENSBTAG00000019944 |  |  |  |
| ENSBTAG00000019947 |  |  |  |
| ENSBTAG00000019948 |  |  |  |
| ENSBTAG00000019950 |  |  |  |
| ENSBTAG00000019952 |  |  |  |
| ENSBTAG00000019953 |  |  |  |
| ENSBTAG00000019954 |  |  |  |
| ENSBTAG00000019956 |  |  |  |
| ENSBTAG00000019958 |  |  |  |
| ENSBTAG00000019960 |  |  |  |
| ENSBTAG00000019963 |  |  |  |
| ENSBTAG00000019967 |  |  |  |
| ENSBTAG00000019972 |  |  |  |
| ENSBTAG00000019980 |  |  |  |
| ENSBTAG00000019984 |  |  |  |
| ENSBTAG00000019986 |  |  |  |
| ENSBTAG00000019987 |  |  |  |
| ENSBTAG00000019988 |  |  |  |
| ENSBTAG00000020012 |  |  |  |
| ENSBTAG00000020014 |  |  |  |
| ENSBTAG00000020015 |  |  |  |
| ENSBTAG00000020017 |  |  |  |
| ENSBTAG00000020018 |  |  |  |
| ENSBTAG00000020022 |  |  |  |
| ENSBTAG00000020023 |  |  |  |
| ENSBTAG00000020026 |  |  |  |
| ENSBTAG00000020030 |  |  |  |
| ENSBTAG00000020031 |  |  |  |
| ENSBTAG00000020034 |  |  |  |
| ENSBTAG00000020035 |  |  |  |
| ENSBTAG00000020037 |  |  |  |
| ENSBTAG00000020042 |  |  |  |
| ENSBTAG00000020043 |  |  |  |
| ENSBTAG00000020046 |  |  |  |
| ENSBTAG00000020047 |  |  |  |
| ENSBTAG00000020048 |  |  |  |
| ENSBTAG00000020051 |  |  |  |
| ENSBTAG00000020054 |  |  |  |
| ENSBTAG00000020060 |  |  |  |
| ENSBTAG00000020061 |  |  |  |
| ENSBTAG00000020062 |  |  |  |
| ENSBTAG00000020066 |  |  |  |
| ENSBTAG00000020067 |  |  |  |
| ENSBTAG00000020070 |  |  |  |
| ENSBTAG00000020072 |  |  |  |
| ENSBTAG00000020073 |  |  |  |
| ENSBTAG00000020074 |  |  |  |
| ENSBTAG00000020079 |  |  |  |
| ENSBTAG00000020084 |  |  |  |
| ENSBTAG00000020087 |  |  |  |
| ENSBTAG00000020090 |  |  |  |
| ENSBTAG00000020097 |  |  |  |
| ENSBTAG00000020099 |  |  |  |
| ENSBTAG00000020106 |  |  |  |
| ENSBTAG00000020107 |  |  |  |
| ENSBTAG00000020115 |  |  |  |
| ENSBTAG00000020116 |  |  |  |
| ENSBTAG00000020119 |  |  |  |
| ENSBTAG00000020123 |  |  |  |
| ENSBTAG00000020126 |  |  |  |
| ENSBTAG00000020127 |  |  |  |
| ENSBTAG00000020136 |  |  |  |
| ENSBTAG00000020139 |  |  |  |
| ENSBTAG00000020148 |  |  |  |
| ENSBTAG00000020149 |  |  |  |
| ENSBTAG00000020153 |  |  |  |
| ENSBTAG00000020154 |  |  |  |
| ENSBTAG00000020160 |  |  |  |
| ENSBTAG00000020169 |  |  |  |
| ENSBTAG00000020172 |  |  |  |
| ENSBTAG00000020173 |  |  |  |
| ENSBTAG00000020174 |  |  |  |
| ENSBTAG00000020175 |  |  |  |
| ENSBTAG00000020180 |  |  |  |
| ENSBTAG00000020183 |  |  |  |
| ENSBTAG00000020184 |  |  |  |
| ENSBTAG00000020186 |  |  |  |
| ENSBTAG00000020190 |  |  |  |
| ENSBTAG00000020192 |  |  |  |
| ENSBTAG00000020193 |  |  |  |
| ENSBTAG00000020194 |  |  |  |
| ENSBTAG00000020199 |  |  |  |
| ENSBTAG00000020202 |  |  |  |
| ENSBTAG00000020205 |  |  |  |
| ENSBTAG00000020214 |  |  |  |
| ENSBTAG00000020218 |  |  |  |
| ENSBTAG00000020219 |  |  |  |
| ENSBTAG00000020223 |  |  |  |
| ENSBTAG00000020226 |  |  |  |
| ENSBTAG00000020227 |  |  |  |
| ENSBTAG00000020229 |  |  |  |
| ENSBTAG00000020232 |  |  |  |
| ENSBTAG00000020233 |  |  |  |
| ENSBTAG00000020236 |  |  |  |
| ENSBTAG00000020237 |  |  |  |
| ENSBTAG00000020244 |  |  |  |
| ENSBTAG00000020247 |  |  |  |
| ENSBTAG00000020249 |  |  |  |
| ENSBTAG00000020250 |  |  |  |
| ENSBTAG00000020252 |  |  |  |
| ENSBTAG00000020257 |  |  |  |
| ENSBTAG00000020262 |  |  |  |
| ENSBTAG00000020263 |  |  |  |
| ENSBTAG00000020269 |  |  |  |
| ENSBTAG00000020270 |  |  |  |
| ENSBTAG00000020272 |  |  |  |
| ENSBTAG00000020273 |  |  |  |
| ENSBTAG00000020281 |  |  |  |
| ENSBTAG00000020282 |  |  |  |
| ENSBTAG00000020285 |  |  |  |
| ENSBTAG00000020294 |  |  |  |
| ENSBTAG00000020296 |  |  |  |
| ENSBTAG00000020297 |  |  |  |
| ENSBTAG00000020303 |  |  |  |
| ENSBTAG00000020307 |  |  |  |
| ENSBTAG00000020308 |  |  |  |
| ENSBTAG00000020311 |  |  |  |
| ENSBTAG00000020314 |  |  |  |
| ENSBTAG00000020318 |  |  |  |
| ENSBTAG00000020319 |  |  |  |
| ENSBTAG00000020321 |  |  |  |
| ENSBTAG00000020327 |  |  |  |
| ENSBTAG00000020329 |  |  |  |
| ENSBTAG00000020330 |  |  |  |
| ENSBTAG00000020334 |  |  |  |
| ENSBTAG00000020342 |  |  |  |
| ENSBTAG00000020345 |  |  |  |
| ENSBTAG00000020346 |  |  |  |
| ENSBTAG00000020351 |  |  |  |
| ENSBTAG00000020355 |  |  |  |
| ENSBTAG00000020356 |  |  |  |
| ENSBTAG00000020357 |  |  |  |
| ENSBTAG00000020359 |  |  |  |
| ENSBTAG00000020371 |  |  |  |
| ENSBTAG00000020373 |  |  |  |
| ENSBTAG00000020374 |  |  |  |
| ENSBTAG00000020379 |  |  |  |
| ENSBTAG00000020381 |  |  |  |
| ENSBTAG00000020382 |  |  |  |
| ENSBTAG00000020387 |  |  |  |
| ENSBTAG00000020389 |  |  |  |
| ENSBTAG00000020390 |  |  |  |
| ENSBTAG00000020393 |  |  |  |
| ENSBTAG00000020394 |  |  |  |
| ENSBTAG00000020395 |  |  |  |
| ENSBTAG00000020404 |  |  |  |
| ENSBTAG00000020407 |  |  |  |
| ENSBTAG00000020412 |  |  |  |
| ENSBTAG00000020413 |  |  |  |
| ENSBTAG00000020417 |  |  |  |
| ENSBTAG00000020418 |  |  |  |
| ENSBTAG00000020421 |  |  |  |
| ENSBTAG00000020423 |  |  |  |
| ENSBTAG00000020427 |  |  |  |
| ENSBTAG00000020431 |  |  |  |
| ENSBTAG00000020432 |  |  |  |
| ENSBTAG00000020434 |  |  |  |
| ENSBTAG00000020441 |  |  |  |
| ENSBTAG00000020446 |  |  |  |
| ENSBTAG00000020449 |  |  |  |
| ENSBTAG00000020454 |  |  |  |
| ENSBTAG00000020455 |  |  |  |
| ENSBTAG00000020458 |  |  |  |
| ENSBTAG00000020465 |  |  |  |
| ENSBTAG00000020475 |  |  |  |
| ENSBTAG00000020477 |  |  |  |
| ENSBTAG00000020480 |  |  |  |
| ENSBTAG00000020481 |  |  |  |
| ENSBTAG00000020482 |  |  |  |
| ENSBTAG00000020484 |  |  |  |
| ENSBTAG00000020489 |  |  |  |
| ENSBTAG00000020494 |  |  |  |
| ENSBTAG00000020495 |  |  |  |
| ENSBTAG00000020496 |  |  |  |
| ENSBTAG00000020498 |  |  |  |
| ENSBTAG00000020504 |  |  |  |
| ENSBTAG00000020517 |  |  |  |
| ENSBTAG00000020518 |  |  |  |
| ENSBTAG00000020520 |  |  |  |
| ENSBTAG00000020525 |  |  |  |
| ENSBTAG00000020527 |  |  |  |
| ENSBTAG00000020530 |  |  |  |
| ENSBTAG00000020536 |  |  |  |
| ENSBTAG00000020538 |  |  |  |
| ENSBTAG00000020540 |  |  |  |
| ENSBTAG00000020541 |  |  |  |
| ENSBTAG00000020542 |  |  |  |
| ENSBTAG00000020543 |  |  |  |
| ENSBTAG00000020544 |  |  |  |
| ENSBTAG00000020551 |  |  |  |
| ENSBTAG00000020558 |  |  |  |
| ENSBTAG00000020567 |  |  |  |
| ENSBTAG00000020568 |  |  |  |
| ENSBTAG00000020570 |  |  |  |
| ENSBTAG00000020573 |  |  |  |
| ENSBTAG00000020575 |  |  |  |
| ENSBTAG00000020578 |  |  |  |
| ENSBTAG00000020589 |  |  |  |
| ENSBTAG00000020595 |  |  |  |
| ENSBTAG00000020597 |  |  |  |
| ENSBTAG00000020600 |  |  |  |
| ENSBTAG00000020602 |  |  |  |
| ENSBTAG00000020605 |  |  |  |
| ENSBTAG00000020607 |  |  |  |
| ENSBTAG00000020608 |  |  |  |
| ENSBTAG00000020610 |  |  |  |
| ENSBTAG00000020611 |  |  |  |
| ENSBTAG00000020612 |  |  |  |
| ENSBTAG00000020613 |  |  |  |
| ENSBTAG00000020616 |  |  |  |
| ENSBTAG00000020626 |  |  |  |
| ENSBTAG00000020630 |  |  |  |
| ENSBTAG00000020632 |  |  |  |
| ENSBTAG00000020634 |  |  |  |
| ENSBTAG00000020635 |  |  |  |
| ENSBTAG00000020638 |  |  |  |
| ENSBTAG00000020642 |  |  |  |
| ENSBTAG00000020644 |  |  |  |
| ENSBTAG00000020645 |  |  |  |
| ENSBTAG00000020647 |  |  |  |
| ENSBTAG00000020648 |  |  |  |
| ENSBTAG00000020649 |  |  |  |
| ENSBTAG00000020650 |  |  |  |
| ENSBTAG00000020655 |  |  |  |
| ENSBTAG00000020658 |  |  |  |
| ENSBTAG00000020663 |  |  |  |
| ENSBTAG00000020664 |  |  |  |
| ENSBTAG00000020674 |  |  |  |
| ENSBTAG00000020676 |  |  |  |
| ENSBTAG00000020679 |  |  |  |
| ENSBTAG00000020688 |  |  |  |
| ENSBTAG00000020689 |  |  |  |
| ENSBTAG00000020698 |  |  |  |
| ENSBTAG00000020699 |  |  |  |
| ENSBTAG00000020704 |  |  |  |
| ENSBTAG00000020705 |  |  |  |
| ENSBTAG00000020709 |  |  |  |
| ENSBTAG00000020710 |  |  |  |
| ENSBTAG00000020713 |  |  |  |
| ENSBTAG00000020714 |  |  |  |
| ENSBTAG00000020717 |  |  |  |
| ENSBTAG00000020720 |  |  |  |
| ENSBTAG00000020721 |  |  |  |
| ENSBTAG00000020725 |  |  |  |
| ENSBTAG00000020726 |  |  |  |
| ENSBTAG00000020731 |  |  |  |
| ENSBTAG00000020735 |  |  |  |
| ENSBTAG00000020736 |  |  |  |
| ENSBTAG00000020739 |  |  |  |
| ENSBTAG00000020747 |  |  |  |
| ENSBTAG00000020754 |  |  |  |
| ENSBTAG00000020755 |  |  |  |
| ENSBTAG00000020758 |  |  |  |
| ENSBTAG00000020760 |  |  |  |
| ENSBTAG00000020761 |  |  |  |
| ENSBTAG00000020766 |  |  |  |
| ENSBTAG00000020769 |  |  |  |
| ENSBTAG00000020773 |  |  |  |
| ENSBTAG00000020776 |  |  |  |
| ENSBTAG00000020777 |  |  |  |
| ENSBTAG00000020780 |  |  |  |
| ENSBTAG00000020781 |  |  |  |
| ENSBTAG00000020782 |  |  |  |
| ENSBTAG00000020783 |  |  |  |
| ENSBTAG00000020787 |  |  |  |
| ENSBTAG00000020791 |  |  |  |
| ENSBTAG00000020793 |  |  |  |
| ENSBTAG00000020796 |  |  |  |
| ENSBTAG00000020801 |  |  |  |
| ENSBTAG00000020810 |  |  |  |
| ENSBTAG00000020813 |  |  |  |
| ENSBTAG00000020824 |  |  |  |
| ENSBTAG00000020828 |  |  |  |
| ENSBTAG00000020829 |  |  |  |
| ENSBTAG00000020836 |  |  |  |
| ENSBTAG00000020837 |  |  |  |
| ENSBTAG00000020843 |  |  |  |
| ENSBTAG00000020844 |  |  |  |
| ENSBTAG00000020848 |  |  |  |
| ENSBTAG00000020850 |  |  |  |
| ENSBTAG00000020853 |  |  |  |
| ENSBTAG00000020858 |  |  |  |
| ENSBTAG00000020859 |  |  |  |
| ENSBTAG00000020860 |  |  |  |
| ENSBTAG00000020865 |  |  |  |
| ENSBTAG00000020872 |  |  |  |
| ENSBTAG00000020873 |  |  |  |
| ENSBTAG00000020878 |  |  |  |
| ENSBTAG00000020880 |  |  |  |
| ENSBTAG00000020883 |  |  |  |
| ENSBTAG00000020884 |  |  |  |
| ENSBTAG00000020886 |  |  |  |
| ENSBTAG00000020889 |  |  |  |
| ENSBTAG00000020892 |  |  |  |
| ENSBTAG00000020894 |  |  |  |
| ENSBTAG00000020895 |  |  |  |
| ENSBTAG00000020896 |  |  |  |
| ENSBTAG00000020898 |  |  |  |
| ENSBTAG00000020900 |  |  |  |
| ENSBTAG00000020904 |  |  |  |
| ENSBTAG00000020907 |  |  |  |
| ENSBTAG00000020908 |  |  |  |
| ENSBTAG00000020914 |  |  |  |
| ENSBTAG00000020916 |  |  |  |
| ENSBTAG00000020921 |  |  |  |
| ENSBTAG00000020924 |  |  |  |
| ENSBTAG00000020927 |  |  |  |
| ENSBTAG00000020931 |  |  |  |
| ENSBTAG00000020933 |  |  |  |
| ENSBTAG00000020934 |  |  |  |
| ENSBTAG00000020936 |  |  |  |
| ENSBTAG00000020937 |  |  |  |
| ENSBTAG00000020938 |  |  |  |
| ENSBTAG00000020939 |  |  |  |
| ENSBTAG00000020940 |  |  |  |
| ENSBTAG00000020942 |  |  |  |
| ENSBTAG00000020956 |  |  |  |
| ENSBTAG00000020958 |  |  |  |
| ENSBTAG00000020963 |  |  |  |
| ENSBTAG00000020964 |  |  |  |
| ENSBTAG00000020968 |  |  |  |
| ENSBTAG00000020969 |  |  |  |
| ENSBTAG00000020973 |  |  |  |
| ENSBTAG00000020975 |  |  |  |
| ENSBTAG00000020979 |  |  |  |
| ENSBTAG00000020980 |  |  |  |
| ENSBTAG00000020983 |  |  |  |
| ENSBTAG00000020984 |  |  |  |
| ENSBTAG00000020989 |  |  |  |
| ENSBTAG00000020990 |  |  |  |
| ENSBTAG00000020994 |  |  |  |
| ENSBTAG00000020996 |  |  |  |
| ENSBTAG00000020998 |  |  |  |
| ENSBTAG00000020999 |  |  |  |
| ENSBTAG00000021002 |  |  |  |
| ENSBTAG00000021009 |  |  |  |
| ENSBTAG00000021013 |  |  |  |
| ENSBTAG00000021019 |  |  |  |
| ENSBTAG00000021023 |  |  |  |
| ENSBTAG00000021025 |  |  |  |
| ENSBTAG00000021029 |  |  |  |
| ENSBTAG00000021035 |  |  |  |
| ENSBTAG00000021036 |  |  |  |
| ENSBTAG00000021041 |  |  |  |
| ENSBTAG00000021046 |  |  |  |
| ENSBTAG00000021048 |  |  |  |
| ENSBTAG00000021050 |  |  |  |
| ENSBTAG00000021065 |  |  |  |
| ENSBTAG00000021066 |  |  |  |
| ENSBTAG00000021067 |  |  |  |
| ENSBTAG00000021069 |  |  |  |
| ENSBTAG00000021072 |  |  |  |
| ENSBTAG00000021073 |  |  |  |
| ENSBTAG00000021076 |  |  |  |
| ENSBTAG00000021077 |  |  |  |
| ENSBTAG00000021078 |  |  |  |
| ENSBTAG00000021082 |  |  |  |
| ENSBTAG00000021083 |  |  |  |
| ENSBTAG00000021091 |  |  |  |
| ENSBTAG00000021092 |  |  |  |
| ENSBTAG00000021100 |  |  |  |
| ENSBTAG00000021102 |  |  |  |
| ENSBTAG00000021103 |  |  |  |
| ENSBTAG00000021105 |  |  |  |
| ENSBTAG00000021107 |  |  |  |
| ENSBTAG00000021115 |  |  |  |
| ENSBTAG00000021116 |  |  |  |
| ENSBTAG00000021119 |  |  |  |
| ENSBTAG00000021120 |  |  |  |
| ENSBTAG00000021121 |  |  |  |
| ENSBTAG00000021130 |  |  |  |
| ENSBTAG00000021131 |  |  |  |
| ENSBTAG00000021134 |  |  |  |
| ENSBTAG00000021140 |  |  |  |
| ENSBTAG00000021141 |  |  |  |
| ENSBTAG00000021143 |  |  |  |
| ENSBTAG00000021157 |  |  |  |
| ENSBTAG00000021158 |  |  |  |
| ENSBTAG00000021160 |  |  |  |
| ENSBTAG00000021162 |  |  |  |
| ENSBTAG00000021164 |  |  |  |
| ENSBTAG00000021165 |  |  |  |
| ENSBTAG00000021168 |  |  |  |
| ENSBTAG00000021174 |  |  |  |
| ENSBTAG00000021176 |  |  |  |
| ENSBTAG00000021180 |  |  |  |
| ENSBTAG00000021181 |  |  |  |
| ENSBTAG00000021187 |  |  |  |
| ENSBTAG00000021189 |  |  |  |
| ENSBTAG00000021190 |  |  |  |
| ENSBTAG00000021191 |  |  |  |
| ENSBTAG00000021196 |  |  |  |
| ENSBTAG00000021202 |  |  |  |
| ENSBTAG00000021205 |  |  |  |
| ENSBTAG00000021209 |  |  |  |
| ENSBTAG00000021210 |  |  |  |
| ENSBTAG00000021211 |  |  |  |
| ENSBTAG00000021214 |  |  |  |
| ENSBTAG00000021216 |  |  |  |
| ENSBTAG00000021217 |  |  |  |
| ENSBTAG00000021219 |  |  |  |
| ENSBTAG00000021222 |  |  |  |
| ENSBTAG00000021223 |  |  |  |
| ENSBTAG00000021226 |  |  |  |
| ENSBTAG00000021227 |  |  |  |
| ENSBTAG00000021230 |  |  |  |
| ENSBTAG00000021231 |  |  |  |
| ENSBTAG00000021232 |  |  |  |
| ENSBTAG00000021240 |  |  |  |
| ENSBTAG00000021242 |  |  |  |
| ENSBTAG00000021245 |  |  |  |
| ENSBTAG00000021246 |  |  |  |
| ENSBTAG00000021248 |  |  |  |
| ENSBTAG00000021249 |  |  |  |
| ENSBTAG00000021250 |  |  |  |
| ENSBTAG00000021251 |  |  |  |
| ENSBTAG00000021254 |  |  |  |
| ENSBTAG00000021260 |  |  |  |
| ENSBTAG00000021262 |  |  |  |
| ENSBTAG00000021263 |  |  |  |
| ENSBTAG00000021272 |  |  |  |
| ENSBTAG00000021275 |  |  |  |
| ENSBTAG00000021276 |  |  |  |
| ENSBTAG00000021282 |  |  |  |
| ENSBTAG00000021284 |  |  |  |
| ENSBTAG00000021286 |  |  |  |
| ENSBTAG00000021287 |  |  |  |
| ENSBTAG00000021288 |  |  |  |
| ENSBTAG00000021289 |  |  |  |
| ENSBTAG00000021292 |  |  |  |
| ENSBTAG00000021293 |  |  |  |
| ENSBTAG00000021294 |  |  |  |
| ENSBTAG00000021301 |  |  |  |
| ENSBTAG00000021302 |  |  |  |
| ENSBTAG00000021303 |  |  |  |
| ENSBTAG00000021304 |  |  |  |
| ENSBTAG00000021307 |  |  |  |
| ENSBTAG00000021313 |  |  |  |
| ENSBTAG00000021318 |  |  |  |
| ENSBTAG00000021321 |  |  |  |
| ENSBTAG00000021323 |  |  |  |
| ENSBTAG00000021325 |  |  |  |
| ENSBTAG00000021334 |  |  |  |
| ENSBTAG00000021337 |  |  |  |
| ENSBTAG00000021338 |  |  |  |
| ENSBTAG00000021339 |  |  |  |
| ENSBTAG00000021342 |  |  |  |
| ENSBTAG00000021351 |  |  |  |
| ENSBTAG00000021360 |  |  |  |
| ENSBTAG00000021361 |  |  |  |
| ENSBTAG00000021364 |  |  |  |
| ENSBTAG00000021372 |  |  |  |
| ENSBTAG00000021373 |  |  |  |
| ENSBTAG00000021374 |  |  |  |
| ENSBTAG00000021377 |  |  |  |
| ENSBTAG00000021392 |  |  |  |
| ENSBTAG00000021396 |  |  |  |
| ENSBTAG00000021397 |  |  |  |
| ENSBTAG00000021398 |  |  |  |
| ENSBTAG00000021410 |  |  |  |
| ENSBTAG00000021417 |  |  |  |
| ENSBTAG00000021424 |  |  |  |
| ENSBTAG00000021426 |  |  |  |
| ENSBTAG00000021427 |  |  |  |
| ENSBTAG00000021428 |  |  |  |
| ENSBTAG00000021430 |  |  |  |
| ENSBTAG00000021434 |  |  |  |
| ENSBTAG00000021435 |  |  |  |
| ENSBTAG00000021442 |  |  |  |
| ENSBTAG00000021444 |  |  |  |
| ENSBTAG00000021445 |  |  |  |
| ENSBTAG00000021452 |  |  |  |
| ENSBTAG00000021455 |  |  |  |
| ENSBTAG00000021456 |  |  |  |
| ENSBTAG00000021457 |  |  |  |
| ENSBTAG00000021461 |  |  |  |
| ENSBTAG00000021462 |  |  |  |
| ENSBTAG00000021469 |  |  |  |
| ENSBTAG00000021471 |  |  |  |
| ENSBTAG00000021480 |  |  |  |
| ENSBTAG00000021482 |  |  |  |
| ENSBTAG00000021483 |  |  |  |
| ENSBTAG00000021490 |  |  |  |
| ENSBTAG00000021491 |  |  |  |
| ENSBTAG00000021493 |  |  |  |
| ENSBTAG00000021497 |  |  |  |
| ENSBTAG00000021499 |  |  |  |
| ENSBTAG00000021501 |  |  |  |
| ENSBTAG00000021506 |  |  |  |
| ENSBTAG00000021508 |  |  |  |
| ENSBTAG00000021516 |  |  |  |
| ENSBTAG00000021517 |  |  |  |
| ENSBTAG00000021518 |  |  |  |
| ENSBTAG00000021521 |  |  |  |
| ENSBTAG00000021523 |  |  |  |
| ENSBTAG00000021526 |  |  |  |
| ENSBTAG00000021537 |  |  |  |
| ENSBTAG00000021540 |  |  |  |
| ENSBTAG00000021543 |  |  |  |
| ENSBTAG00000021549 |  |  |  |
| ENSBTAG00000021553 |  |  |  |
| ENSBTAG00000021556 |  |  |  |
| ENSBTAG00000021558 |  |  |  |
| ENSBTAG00000021565 |  |  |  |
| ENSBTAG00000021570 |  |  |  |
| ENSBTAG00000021573 |  |  |  |
| ENSBTAG00000021574 |  |  |  |
| ENSBTAG00000021576 |  |  |  |
| ENSBTAG00000021577 |  |  |  |
| ENSBTAG00000021580 |  |  |  |
| ENSBTAG00000021581 |  |  |  |
| ENSBTAG00000021583 |  |  |  |
| ENSBTAG00000021587 |  |  |  |
| ENSBTAG00000021588 |  |  |  |
| ENSBTAG00000021591 |  |  |  |
| ENSBTAG00000021592 |  |  |  |
| ENSBTAG00000021596 |  |  |  |
| ENSBTAG00000021602 |  |  |  |
| ENSBTAG00000021604 |  |  |  |
| ENSBTAG00000021606 |  |  |  |
| ENSBTAG00000021609 |  |  |  |
| ENSBTAG00000021611 |  |  |  |
| ENSBTAG00000021614 |  |  |  |
| ENSBTAG00000021620 |  |  |  |
| ENSBTAG00000021630 |  |  |  |
| ENSBTAG00000021632 |  |  |  |
| ENSBTAG00000021633 |  |  |  |
| ENSBTAG00000021634 |  |  |  |
| ENSBTAG00000021635 |  |  |  |
| ENSBTAG00000021652 |  |  |  |
| ENSBTAG00000021653 |  |  |  |
| ENSBTAG00000021654 |  |  |  |
| ENSBTAG00000021656 |  |  |  |
| ENSBTAG00000021657 |  |  |  |
| ENSBTAG00000021658 |  |  |  |
| ENSBTAG00000021663 |  |  |  |
| ENSBTAG00000021664 |  |  |  |
| ENSBTAG00000021672 |  |  |  |
| ENSBTAG00000021675 |  |  |  |
| ENSBTAG00000021678 |  |  |  |
| ENSBTAG00000021680 |  |  |  |
| ENSBTAG00000021685 |  |  |  |
| ENSBTAG00000021691 |  |  |  |
| ENSBTAG00000021694 |  |  |  |
| ENSBTAG00000021697 |  |  |  |
| ENSBTAG00000021699 |  |  |  |
| ENSBTAG00000021700 |  |  |  |
| ENSBTAG00000021705 |  |  |  |
| ENSBTAG00000021707 |  |  |  |
| ENSBTAG00000021709 |  |  |  |
| ENSBTAG00000021715 |  |  |  |
| ENSBTAG00000021721 |  |  |  |
| ENSBTAG00000021723 |  |  |  |
| ENSBTAG00000021725 |  |  |  |
| ENSBTAG00000021728 |  |  |  |
| ENSBTAG00000021731 |  |  |  |
| ENSBTAG00000021739 |  |  |  |
| ENSBTAG00000021741 |  |  |  |
| ENSBTAG00000021743 |  |  |  |
| ENSBTAG00000021744 |  |  |  |
| ENSBTAG00000021745 |  |  |  |
| ENSBTAG00000021746 |  |  |  |
| ENSBTAG00000021751 |  |  |  |
| ENSBTAG00000021756 |  |  |  |
| ENSBTAG00000021759 |  |  |  |
| ENSBTAG00000021761 |  |  |  |
| ENSBTAG00000021762 |  |  |  |
| ENSBTAG00000021766 |  |  |  |
| ENSBTAG00000021767 |  |  |  |
| ENSBTAG00000021768 |  |  |  |
| ENSBTAG00000021769 |  |  |  |
| ENSBTAG00000021771 |  |  |  |
| ENSBTAG00000021772 |  |  |  |
| ENSBTAG00000021775 |  |  |  |
| ENSBTAG00000021778 |  |  |  |
| ENSBTAG00000021779 |  |  |  |
| ENSBTAG00000021780 |  |  |  |
| ENSBTAG00000021781 |  |  |  |
| ENSBTAG00000021791 |  |  |  |
| ENSBTAG00000021795 |  |  |  |
| ENSBTAG00000021796 |  |  |  |
| ENSBTAG00000021799 |  |  |  |
| ENSBTAG00000021802 |  |  |  |
| ENSBTAG00000021805 |  |  |  |
| ENSBTAG00000021808 |  |  |  |
| ENSBTAG00000021810 |  |  |  |
| ENSBTAG00000021811 |  |  |  |
| ENSBTAG00000021813 |  |  |  |
| ENSBTAG00000021815 |  |  |  |
| ENSBTAG00000021818 |  |  |  |
| ENSBTAG00000021819 |  |  |  |
| ENSBTAG00000021820 |  |  |  |
| ENSBTAG00000021827 |  |  |  |
| ENSBTAG00000021829 |  |  |  |
| ENSBTAG00000021830 |  |  |  |
| ENSBTAG00000021836 |  |  |  |
| ENSBTAG00000021837 |  |  |  |
| ENSBTAG00000021838 |  |  |  |
| ENSBTAG00000021842 |  |  |  |
| ENSBTAG00000021844 |  |  |  |
| ENSBTAG00000021845 |  |  |  |
| ENSBTAG00000021849 |  |  |  |
| ENSBTAG00000021850 |  |  |  |
| ENSBTAG00000021851 |  |  |  |
| ENSBTAG00000021853 |  |  |  |
| ENSBTAG00000021856 |  |  |  |
| ENSBTAG00000021858 |  |  |  |
| ENSBTAG00000021859 |  |  |  |
| ENSBTAG00000021869 |  |  |  |
| ENSBTAG00000021872 |  |  |  |
| ENSBTAG00000021879 |  |  |  |
| ENSBTAG00000021880 |  |  |  |
| ENSBTAG00000021883 |  |  |  |
| ENSBTAG00000021884 |  |  |  |
| ENSBTAG00000021886 |  |  |  |
| ENSBTAG00000021887 |  |  |  |
| ENSBTAG00000021894 |  |  |  |
| ENSBTAG00000021896 |  |  |  |
| ENSBTAG00000021900 |  |  |  |
| ENSBTAG00000021901 |  |  |  |
| ENSBTAG00000021902 |  |  |  |
| ENSBTAG00000021903 |  |  |  |
| ENSBTAG00000021905 |  |  |  |
| ENSBTAG00000021910 |  |  |  |
| ENSBTAG00000021911 |  |  |  |
| ENSBTAG00000021912 |  |  |  |
| ENSBTAG00000021913 |  |  |  |
| ENSBTAG00000021916 |  |  |  |
| ENSBTAG00000021918 |  |  |  |
| ENSBTAG00000021919 |  |  |  |
| ENSBTAG00000021921 |  |  |  |
| ENSBTAG00000021922 |  |  |  |
| ENSBTAG00000021923 |  |  |  |
| ENSBTAG00000021927 |  |  |  |
| ENSBTAG00000021931 |  |  |  |
| ENSBTAG00000021934 |  |  |  |
| ENSBTAG00000021939 |  |  |  |
| ENSBTAG00000021940 |  |  |  |
| ENSBTAG00000021941 |  |  |  |
| ENSBTAG00000021943 |  |  |  |
| ENSBTAG00000021945 |  |  |  |
| ENSBTAG00000021948 |  |  |  |
| ENSBTAG00000021949 |  |  |  |
| ENSBTAG00000021951 |  |  |  |
| ENSBTAG00000021953 |  |  |  |
| ENSBTAG00000021955 |  |  |  |
| ENSBTAG00000021957 |  |  |  |
| ENSBTAG00000021958 |  |  |  |
| ENSBTAG00000021961 |  |  |  |
| ENSBTAG00000021962 |  |  |  |
| ENSBTAG00000021964 |  |  |  |
| ENSBTAG00000021965 |  |  |  |
| ENSBTAG00000021967 |  |  |  |
| ENSBTAG00000021969 |  |  |  |
| ENSBTAG00000021971 |  |  |  |
| ENSBTAG00000021975 |  |  |  |
| ENSBTAG00000021980 |  |  |  |
| ENSBTAG00000021981 |  |  |  |
| ENSBTAG00000021987 |  |  |  |
| ENSBTAG00000021988 |  |  |  |
| ENSBTAG00000021992 |  |  |  |
| ENSBTAG00000021993 |  |  |  |
| ENSBTAG00000021997 |  |  |  |
| ENSBTAG00000021999 |  |  |  |
| ENSBTAG00000022003 |  |  |  |
| ENSBTAG00000022004 |  |  |  |
| ENSBTAG00000022005 |  |  |  |
| ENSBTAG00000022009 |  |  |  |
| ENSBTAG00000022013 |  |  |  |
| ENSBTAG00000022020 |  |  |  |
| ENSBTAG00000022022 |  |  |  |
| ENSBTAG00000022027 |  |  |  |
| ENSBTAG00000022032 |  |  |  |
| ENSBTAG00000022044 |  |  |  |
| ENSBTAG00000022067 |  |  |  |
| ENSBTAG00000022069 |  |  |  |
| ENSBTAG00000022109 |  |  |  |
| ENSBTAG00000022114 |  |  |  |
| ENSBTAG00000022120 |  |  |  |
| ENSBTAG00000022155 |  |  |  |
| ENSBTAG00000022160 |  |  |  |
| ENSBTAG00000022167 |  |  |  |
| ENSBTAG00000022169 |  |  |  |
| ENSBTAG00000022209 |  |  |  |
| ENSBTAG00000022238 |  |  |  |
| ENSBTAG00000022242 |  |  |  |
| ENSBTAG00000022246 |  |  |  |
| ENSBTAG00000022278 |  |  |  |
| ENSBTAG00000022288 |  |  |  |
| ENSBTAG00000022292 |  |  |  |
| ENSBTAG00000022314 |  |  |  |
| ENSBTAG00000022329 |  |  |  |
| ENSBTAG00000022360 |  |  |  |
| ENSBTAG00000022382 |  |  |  |
| ENSBTAG00000022449 |  |  |  |
| ENSBTAG00000022471 |  |  |  |
| ENSBTAG00000022498 |  |  |  |
| ENSBTAG00000022509 |  |  |  |
| ENSBTAG00000022530 |  |  |  |
| ENSBTAG00000022539 |  |  |  |
| ENSBTAG00000022564 |  |  |  |
| ENSBTAG00000022570 |  |  |  |
| ENSBTAG00000022580 |  |  |  |
| ENSBTAG00000022588 |  |  |  |
| ENSBTAG00000022590 |  |  |  |
| ENSBTAG00000022598 |  |  |  |
| ENSBTAG00000022613 |  |  |  |
| ENSBTAG00000022622 |  |  |  |
| ENSBTAG00000022632 |  |  |  |
| ENSBTAG00000022656 |  |  |  |
| ENSBTAG00000022681 |  |  |  |
| ENSBTAG00000022699 |  |  |  |
| ENSBTAG00000022715 |  |  |  |
| ENSBTAG00000022721 |  |  |  |
| ENSBTAG00000022731 |  |  |  |
| ENSBTAG00000022741 |  |  |  |
| ENSBTAG00000022759 |  |  |  |
| ENSBTAG00000022775 |  |  |  |
| ENSBTAG00000022777 |  |  |  |
| ENSBTAG00000022808 |  |  |  |
| ENSBTAG00000022813 |  |  |  |
| ENSBTAG00000022825 |  |  |  |
| ENSBTAG00000022837 |  |  |  |
| ENSBTAG00000022890 |  |  |  |
| ENSBTAG00000022895 |  |  |  |
| ENSBTAG00000022917 |  |  |  |
| ENSBTAG00000022920 |  |  |  |
| ENSBTAG00000022938 |  |  |  |
| ENSBTAG00000022960 |  |  |  |
| ENSBTAG00000022989 |  |  |  |
| ENSBTAG00000022991 |  |  |  |
| ENSBTAG00000023007 |  |  |  |
| ENSBTAG00000023018 |  |  |  |
| ENSBTAG00000023026 |  |  |  |
| ENSBTAG00000023028 |  |  |  |
| ENSBTAG00000023032 |  |  |  |
| ENSBTAG00000023054 |  |  |  |
| ENSBTAG00000023073 |  |  |  |
| ENSBTAG00000023144 |  |  |  |
| ENSBTAG00000023169 |  |  |  |
| ENSBTAG00000023172 |  |  |  |
| ENSBTAG00000023179 |  |  |  |
| ENSBTAG00000023198 |  |  |  |
| ENSBTAG00000023218 |  |  |  |
| ENSBTAG00000023259 |  |  |  |
| ENSBTAG00000023283 |  |  |  |
| ENSBTAG00000023374 |  |  |  |
| ENSBTAG00000023412 |  |  |  |
| ENSBTAG00000023415 |  |  |  |
| ENSBTAG00000023416 |  |  |  |
| ENSBTAG00000023417 |  |  |  |
| ENSBTAG00000023419 |  |  |  |
| ENSBTAG00000023426 |  |  |  |
| ENSBTAG00000023452 |  |  |  |
| ENSBTAG00000023453 |  |  |  |
| ENSBTAG00000023462 |  |  |  |
| ENSBTAG00000023471 |  |  |  |
| ENSBTAG00000023523 |  |  |  |
| ENSBTAG00000023551 |  |  |  |
| ENSBTAG00000023600 |  |  |  |
| ENSBTAG00000023601 |  |  |  |
| ENSBTAG00000023628 |  |  |  |
| ENSBTAG00000023632 |  |  |  |
| ENSBTAG00000023635 |  |  |  |
| ENSBTAG00000023648 |  |  |  |
| ENSBTAG00000023666 |  |  |  |
| ENSBTAG00000023718 |  |  |  |
| ENSBTAG00000023730 |  |  |  |
| ENSBTAG00000023731 |  |  |  |
| ENSBTAG00000023734 |  |  |  |
| ENSBTAG00000023736 |  |  |  |
| ENSBTAG00000023744 |  |  |  |
| ENSBTAG00000023776 |  |  |  |
| ENSBTAG00000023784 |  |  |  |
| ENSBTAG00000023795 |  |  |  |
| ENSBTAG00000023806 |  |  |  |
| ENSBTAG00000023814 |  |  |  |
| ENSBTAG00000023831 |  |  |  |
| ENSBTAG00000023832 |  |  |  |
| ENSBTAG00000023846 |  |  |  |
| ENSBTAG00000023847 |  |  |  |
| ENSBTAG00000023851 |  |  |  |
| ENSBTAG00000023891 |  |  |  |
| ENSBTAG00000023929 |  |  |  |
| ENSBTAG00000023938 |  |  |  |
| ENSBTAG00000023939 |  |  |  |
| ENSBTAG00000023947 |  |  |  |
| ENSBTAG00000023963 |  |  |  |
| ENSBTAG00000023976 |  |  |  |
| ENSBTAG00000023986 |  |  |  |
| ENSBTAG00000023989 |  |  |  |
| ENSBTAG00000023997 |  |  |  |
| ENSBTAG00000024000 |  |  |  |
| ENSBTAG00000024015 |  |  |  |
| ENSBTAG00000024027 |  |  |  |
| ENSBTAG00000024042 |  |  |  |
| ENSBTAG00000024044 |  |  |  |
| ENSBTAG00000024058 |  |  |  |
| ENSBTAG00000024086 |  |  |  |
| ENSBTAG00000024091 |  |  |  |
| ENSBTAG00000024096 |  |  |  |
| ENSBTAG00000024107 |  |  |  |
| ENSBTAG00000024137 |  |  |  |
| ENSBTAG00000024157 |  |  |  |
| ENSBTAG00000024162 |  |  |  |
| ENSBTAG00000024169 |  |  |  |
| ENSBTAG00000024219 |  |  |  |
| ENSBTAG00000024240 |  |  |  |
| ENSBTAG00000024255 |  |  |  |
| ENSBTAG00000024275 |  |  |  |
| ENSBTAG00000024291 |  |  |  |
| ENSBTAG00000024378 |  |  |  |
| ENSBTAG00000024379 |  |  |  |
| ENSBTAG00000024381 |  |  |  |
| ENSBTAG00000024406 |  |  |  |
| ENSBTAG00000024443 |  |  |  |
| ENSBTAG00000024450 |  |  |  |
| ENSBTAG00000024470 |  |  |  |
| ENSBTAG00000024482 |  |  |  |
| ENSBTAG00000024485 |  |  |  |
| ENSBTAG00000024496 |  |  |  |
| ENSBTAG00000024509 |  |  |  |
| ENSBTAG00000024510 |  |  |  |
| ENSBTAG00000024539 |  |  |  |
| ENSBTAG00000024549 |  |  |  |
| ENSBTAG00000024582 |  |  |  |
| ENSBTAG00000024605 |  |  |  |
| ENSBTAG00000024608 |  |  |  |
| ENSBTAG00000024663 |  |  |  |
| ENSBTAG00000024683 |  |  |  |
| ENSBTAG00000024701 |  |  |  |
| ENSBTAG00000024708 |  |  |  |
| ENSBTAG00000024723 |  |  |  |
| ENSBTAG00000024772 |  |  |  |
| ENSBTAG00000024781 |  |  |  |
| ENSBTAG00000024787 |  |  |  |
| ENSBTAG00000024801 |  |  |  |
| ENSBTAG00000024803 |  |  |  |
| ENSBTAG00000024822 |  |  |  |
| ENSBTAG00000024826 |  |  |  |
| ENSBTAG00000024849 |  |  |  |
| ENSBTAG00000024884 |  |  |  |
| ENSBTAG00000024889 |  |  |  |
| ENSBTAG00000024909 |  |  |  |
| ENSBTAG00000024918 |  |  |  |
| ENSBTAG00000024928 |  |  |  |
| ENSBTAG00000024929 |  |  |  |
| ENSBTAG00000024942 |  |  |  |
| ENSBTAG00000024947 |  |  |  |
| ENSBTAG00000024957 |  |  |  |
| ENSBTAG00000024958 |  |  |  |
| ENSBTAG00000024960 |  |  |  |
| ENSBTAG00000024974 |  |  |  |
| ENSBTAG00000024983 |  |  |  |
| ENSBTAG00000025003 |  |  |  |
| ENSBTAG00000025005 |  |  |  |
| ENSBTAG00000025009 |  |  |  |
| ENSBTAG00000025028 |  |  |  |
| ENSBTAG00000025029 |  |  |  |
| ENSBTAG00000025035 |  |  |  |
| ENSBTAG00000025046 |  |  |  |
| ENSBTAG00000025062 |  |  |  |
| ENSBTAG00000025078 |  |  |  |
| ENSBTAG00000025088 |  |  |  |
| ENSBTAG00000025099 |  |  |  |
| ENSBTAG00000025126 |  |  |  |
| ENSBTAG00000025136 |  |  |  |
| ENSBTAG00000025148 |  |  |  |
| ENSBTAG00000025149 |  |  |  |
| ENSBTAG00000025181 |  |  |  |
| ENSBTAG00000025182 |  |  |  |
| ENSBTAG00000025183 |  |  |  |
| ENSBTAG00000025191 |  |  |  |
| ENSBTAG00000025192 |  |  |  |
| ENSBTAG00000025212 |  |  |  |
| ENSBTAG00000025213 |  |  |  |
| ENSBTAG00000025242 |  |  |  |
| ENSBTAG00000025246 |  |  |  |
| ENSBTAG00000025250 |  |  |  |
| ENSBTAG00000025263 |  |  |  |
| ENSBTAG00000025266 |  |  |  |
| ENSBTAG00000025297 |  |  |  |
| ENSBTAG00000025310 |  |  |  |
| ENSBTAG00000025311 |  |  |  |
| ENSBTAG00000025313 |  |  |  |
| ENSBTAG00000025320 |  |  |  |
| ENSBTAG00000025340 |  |  |  |
| ENSBTAG00000025358 |  |  |  |
| ENSBTAG00000025402 |  |  |  |
| ENSBTAG00000025403 |  |  |  |
| ENSBTAG00000025405 |  |  |  |
| ENSBTAG00000025410 |  |  |  |
| ENSBTAG00000025413 |  |  |  |
| ENSBTAG00000025425 |  |  |  |
| ENSBTAG00000025434 |  |  |  |
| ENSBTAG00000025442 |  |  |  |
| ENSBTAG00000025450 |  |  |  |
| ENSBTAG00000025462 |  |  |  |
| ENSBTAG00000025471 |  |  |  |
| ENSBTAG00000025485 |  |  |  |
| ENSBTAG00000025494 |  |  |  |
| ENSBTAG00000025502 |  |  |  |
| ENSBTAG00000025526 |  |  |  |
| ENSBTAG00000025531 |  |  |  |
| ENSBTAG00000025554 |  |  |  |
| ENSBTAG00000025589 |  |  |  |
| ENSBTAG00000025593 |  |  |  |
| ENSBTAG00000025606 |  |  |  |
| ENSBTAG00000025612 |  |  |  |
| ENSBTAG00000025622 |  |  |  |
| ENSBTAG00000025632 |  |  |  |
| ENSBTAG00000025642 |  |  |  |
| ENSBTAG00000025644 |  |  |  |
| ENSBTAG00000025663 |  |  |  |
| ENSBTAG00000025664 |  |  |  |
| ENSBTAG00000025669 |  |  |  |
| ENSBTAG00000025748 |  |  |  |
| ENSBTAG00000025752 |  |  |  |
| ENSBTAG00000025762 |  |  |  |
| ENSBTAG00000025775 |  |  |  |
| ENSBTAG00000025782 |  |  |  |
| ENSBTAG00000025788 |  |  |  |
| ENSBTAG00000025817 |  |  |  |
| ENSBTAG00000025830 |  |  |  |
| ENSBTAG00000025837 |  |  |  |
| ENSBTAG00000025853 |  |  |  |
| ENSBTAG00000025859 |  |  |  |
| ENSBTAG00000025898 |  |  |  |
| ENSBTAG00000025903 |  |  |  |
| ENSBTAG00000025920 |  |  |  |
| ENSBTAG00000025931 |  |  |  |
| ENSBTAG00000025942 |  |  |  |
| ENSBTAG00000026003 |  |  |  |
| ENSBTAG00000026004 |  |  |  |
| ENSBTAG00000026016 |  |  |  |
| ENSBTAG00000026067 |  |  |  |
| ENSBTAG00000026080 |  |  |  |
| ENSBTAG00000026088 |  |  |  |
| ENSBTAG00000026102 |  |  |  |
| ENSBTAG00000026111 |  |  |  |
| ENSBTAG00000026114 |  |  |  |
| ENSBTAG00000026172 |  |  |  |
| ENSBTAG00000026192 |  |  |  |
| ENSBTAG00000026199 |  |  |  |
| ENSBTAG00000026234 |  |  |  |
| ENSBTAG00000026236 |  |  |  |
| ENSBTAG00000026242 |  |  |  |
| ENSBTAG00000026260 |  |  |  |
| ENSBTAG00000026263 |  |  |  |
| ENSBTAG00000026275 |  |  |  |
| ENSBTAG00000026278 |  |  |  |
| ENSBTAG00000026283 |  |  |  |
| ENSBTAG00000026323 |  |  |  |
| ENSBTAG00000026326 |  |  |  |
| ENSBTAG00000026369 |  |  |  |
| ENSBTAG00000026375 |  |  |  |
| ENSBTAG00000026376 |  |  |  |
| ENSBTAG00000026394 |  |  |  |
| ENSBTAG00000026403 |  |  |  |
| ENSBTAG00000026415 |  |  |  |
| ENSBTAG00000026428 |  |  |  |
| ENSBTAG00000026429 |  |  |  |
| ENSBTAG00000026481 |  |  |  |
| ENSBTAG00000026519 |  |  |  |
| ENSBTAG00000026585 |  |  |  |
| ENSBTAG00000026586 |  |  |  |
| ENSBTAG00000026604 |  |  |  |
| ENSBTAG00000026624 |  |  |  |
| ENSBTAG00000026626 |  |  |  |
| ENSBTAG00000026637 |  |  |  |
| ENSBTAG00000026660 |  |  |  |
| ENSBTAG00000026676 |  |  |  |
| ENSBTAG00000026684 |  |  |  |
| ENSBTAG00000026696 |  |  |  |
| ENSBTAG00000026704 |  |  |  |
| ENSBTAG00000026708 |  |  |  |
| ENSBTAG00000026748 |  |  |  |
| ENSBTAG00000026753 |  |  |  |
| ENSBTAG00000026769 |  |  |  |
| ENSBTAG00000026779 |  |  |  |
| ENSBTAG00000026792 |  |  |  |
| ENSBTAG00000026812 |  |  |  |
| ENSBTAG00000026813 |  |  |  |
| ENSBTAG00000026819 |  |  |  |
| ENSBTAG00000026825 |  |  |  |
| ENSBTAG00000026829 |  |  |  |
| ENSBTAG00000026836 |  |  |  |
| ENSBTAG00000026880 |  |  |  |
| ENSBTAG00000026915 |  |  |  |
| ENSBTAG00000026916 |  |  |  |
| ENSBTAG00000026919 |  |  |  |
| ENSBTAG00000026963 |  |  |  |
| ENSBTAG00000026972 |  |  |  |
| ENSBTAG00000026977 |  |  |  |
| ENSBTAG00000026993 |  |  |  |
| ENSBTAG00000026994 |  |  |  |
| ENSBTAG00000026995 |  |  |  |
| ENSBTAG00000027017 |  |  |  |
| ENSBTAG00000027051 |  |  |  |
| ENSBTAG00000027059 |  |  |  |
| ENSBTAG00000027064 |  |  |  |
| ENSBTAG00000027069 |  |  |  |
| ENSBTAG00000027074 |  |  |  |
| ENSBTAG00000027126 |  |  |  |
| ENSBTAG00000027134 |  |  |  |
| ENSBTAG00000027151 |  |  |  |
| ENSBTAG00000027159 |  |  |  |
| ENSBTAG00000027162 |  |  |  |
| ENSBTAG00000027173 |  |  |  |
| ENSBTAG00000027205 |  |  |  |
| ENSBTAG00000027246 |  |  |  |
| ENSBTAG00000027316 |  |  |  |
| ENSBTAG00000027317 |  |  |  |
| ENSBTAG00000027320 |  |  |  |
| ENSBTAG00000027326 |  |  |  |
| ENSBTAG00000027337 |  |  |  |
| ENSBTAG00000027348 |  |  |  |
| ENSBTAG00000027387 |  |  |  |
| ENSBTAG00000027390 |  |  |  |
| ENSBTAG00000027407 |  |  |  |
| ENSBTAG00000027409 |  |  |  |
| ENSBTAG00000027412 |  |  |  |
| ENSBTAG00000027420 |  |  |  |
| ENSBTAG00000027425 |  |  |  |
| ENSBTAG00000027431 |  |  |  |
| ENSBTAG00000027442 |  |  |  |
| ENSBTAG00000027446 |  |  |  |
| ENSBTAG00000027453 |  |  |  |
| ENSBTAG00000027477 |  |  |  |
| ENSBTAG00000027506 |  |  |  |
| ENSBTAG00000027513 |  |  |  |
| ENSBTAG00000027516 |  |  |  |
| ENSBTAG00000027557 |  |  |  |
| ENSBTAG00000027569 |  |  |  |
| ENSBTAG00000027625 |  |  |  |
| ENSBTAG00000027626 |  |  |  |
| ENSBTAG00000027629 |  |  |  |
| ENSBTAG00000027654 |  |  |  |
| ENSBTAG00000027655 |  |  |  |
| ENSBTAG00000027665 |  |  |  |
| ENSBTAG00000027676 |  |  |  |
| ENSBTAG00000027694 |  |  |  |
| ENSBTAG00000027696 |  |  |  |
| ENSBTAG00000027713 |  |  |  |
| ENSBTAG00000027722 |  |  |  |
| ENSBTAG00000027728 |  |  |  |
| ENSBTAG00000027764 |  |  |  |
| ENSBTAG00000027766 |  |  |  |
| ENSBTAG00000027772 |  |  |  |
| ENSBTAG00000027775 |  |  |  |
| ENSBTAG00000027809 |  |  |  |
| ENSBTAG00000027832 |  |  |  |
| ENSBTAG00000027843 |  |  |  |
| ENSBTAG00000027879 |  |  |  |
| ENSBTAG00000027924 |  |  |  |
| ENSBTAG00000027937 |  |  |  |
| ENSBTAG00000027991 |  |  |  |
| ENSBTAG00000030162 |  |  |  |
| ENSBTAG00000030168 |  |  |  |
| ENSBTAG00000030169 |  |  |  |
| ENSBTAG00000030170 |  |  |  |
| ENSBTAG00000030172 |  |  |  |
| ENSBTAG00000030175 |  |  |  |
| ENSBTAG00000030179 |  |  |  |
| ENSBTAG00000030189 |  |  |  |
| ENSBTAG00000030190 |  |  |  |
| ENSBTAG00000030209 |  |  |  |
| ENSBTAG00000030210 |  |  |  |
| ENSBTAG00000030222 |  |  |  |
| ENSBTAG00000030227 |  |  |  |
| ENSBTAG00000030246 |  |  |  |
| ENSBTAG00000030255 |  |  |  |
| ENSBTAG00000030258 |  |  |  |
| ENSBTAG00000030282 |  |  |  |
| ENSBTAG00000030286 |  |  |  |
| ENSBTAG00000030302 |  |  |  |
| ENSBTAG00000030319 |  |  |  |
| ENSBTAG00000030333 |  |  |  |
| ENSBTAG00000030335 |  |  |  |
| ENSBTAG00000030340 |  |  |  |
| ENSBTAG00000030366 |  |  |  |
| ENSBTAG00000030367 |  |  |  |
| ENSBTAG00000030369 |  |  |  |
| ENSBTAG00000030393 |  |  |  |
| ENSBTAG00000030403 |  |  |  |
| ENSBTAG00000030423 |  |  |  |
| ENSBTAG00000030424 |  |  |  |
| ENSBTAG00000030425 |  |  |  |
| ENSBTAG00000030434 |  |  |  |
| ENSBTAG00000030435 |  |  |  |
| ENSBTAG00000030453 |  |  |  |
| ENSBTAG00000030474 |  |  |  |
| ENSBTAG00000030483 |  |  |  |
| ENSBTAG00000030503 |  |  |  |
| ENSBTAG00000030519 |  |  |  |
| ENSBTAG00000030523 |  |  |  |
| ENSBTAG00000030529 |  |  |  |
| ENSBTAG00000030539 |  |  |  |
| ENSBTAG00000030542 |  |  |  |
| ENSBTAG00000030557 |  |  |  |
| ENSBTAG00000030559 |  |  |  |
| ENSBTAG00000030566 |  |  |  |
| ENSBTAG00000030584 |  |  |  |
| ENSBTAG00000030587 |  |  |  |
| ENSBTAG00000030591 |  |  |  |
| ENSBTAG00000030593 |  |  |  |
| ENSBTAG00000030595 |  |  |  |
| ENSBTAG00000030599 |  |  |  |
| ENSBTAG00000030629 |  |  |  |
| ENSBTAG00000030632 |  |  |  |
| ENSBTAG00000030646 |  |  |  |
| ENSBTAG00000030648 |  |  |  |
| ENSBTAG00000030650 |  |  |  |
| ENSBTAG00000030674 |  |  |  |
| ENSBTAG00000030675 |  |  |  |
| ENSBTAG00000030683 |  |  |  |
| ENSBTAG00000030686 |  |  |  |
| ENSBTAG00000030690 |  |  |  |
| ENSBTAG00000030706 |  |  |  |
| ENSBTAG00000030711 |  |  |  |
| ENSBTAG00000030718 |  |  |  |
| ENSBTAG00000030744 |  |  |  |
| ENSBTAG00000030749 |  |  |  |
| ENSBTAG00000030769 |  |  |  |
| ENSBTAG00000030784 |  |  |  |
| ENSBTAG00000030805 |  |  |  |
| ENSBTAG00000030809 |  |  |  |
| ENSBTAG00000030817 |  |  |  |
| ENSBTAG00000030824 |  |  |  |
| ENSBTAG00000030836 |  |  |  |
| ENSBTAG00000030855 |  |  |  |
| ENSBTAG00000030864 |  |  |  |
| ENSBTAG00000030881 |  |  |  |
| ENSBTAG00000030897 |  |  |  |
| ENSBTAG00000030898 |  |  |  |
| ENSBTAG00000030913 |  |  |  |
| ENSBTAG00000030915 |  |  |  |
| ENSBTAG00000030920 |  |  |  |
| ENSBTAG00000030930 |  |  |  |
| ENSBTAG00000030932 |  |  |  |
| ENSBTAG00000030940 |  |  |  |
| ENSBTAG00000030951 |  |  |  |
| ENSBTAG00000030956 |  |  |  |
| ENSBTAG00000030960 |  |  |  |
| ENSBTAG00000030965 |  |  |  |
| ENSBTAG00000030966 |  |  |  |
| ENSBTAG00000030974 |  |  |  |
| ENSBTAG00000030976 |  |  |  |
| ENSBTAG00000030977 |  |  |  |
| ENSBTAG00000031012 |  |  |  |
| ENSBTAG00000031014 |  |  |  |
| ENSBTAG00000031041 |  |  |  |
| ENSBTAG00000031069 |  |  |  |
| ENSBTAG00000031135 |  |  |  |
| ENSBTAG00000031146 |  |  |  |
| ENSBTAG00000031165 |  |  |  |
| ENSBTAG00000031178 |  |  |  |
| ENSBTAG00000031184 |  |  |  |
| ENSBTAG00000031185 |  |  |  |
| ENSBTAG00000031188 |  |  |  |
| ENSBTAG00000031194 |  |  |  |
| ENSBTAG00000031231 |  |  |  |
| ENSBTAG00000031236 |  |  |  |
| ENSBTAG00000031246 |  |  |  |
| ENSBTAG00000031249 |  |  |  |
| ENSBTAG00000031252 |  |  |  |
| ENSBTAG00000031267 |  |  |  |
| ENSBTAG00000031287 |  |  |  |
| ENSBTAG00000031295 |  |  |  |
| ENSBTAG00000031309 |  |  |  |
| ENSBTAG00000031327 |  |  |  |
| ENSBTAG00000031335 |  |  |  |
| ENSBTAG00000031340 |  |  |  |
| ENSBTAG00000031348 |  |  |  |
| ENSBTAG00000031352 |  |  |  |
| ENSBTAG00000031355 |  |  |  |
| ENSBTAG00000031358 |  |  |  |
| ENSBTAG00000031376 |  |  |  |
| ENSBTAG00000031377 |  |  |  |
| ENSBTAG00000031383 |  |  |  |
| ENSBTAG00000031385 |  |  |  |
| ENSBTAG00000031397 |  |  |  |
| ENSBTAG00000031430 |  |  |  |
| ENSBTAG00000031432 |  |  |  |
| ENSBTAG00000031433 |  |  |  |
| ENSBTAG00000031435 |  |  |  |
| ENSBTAG00000031441 |  |  |  |
| ENSBTAG00000031444 |  |  |  |
| ENSBTAG00000031447 |  |  |  |
| ENSBTAG00000031461 |  |  |  |
| ENSBTAG00000031468 |  |  |  |
| ENSBTAG00000031473 |  |  |  |
| ENSBTAG00000031497 |  |  |  |
| ENSBTAG00000031500 |  |  |  |
| ENSBTAG00000031503 |  |  |  |
| ENSBTAG00000031524 |  |  |  |
| ENSBTAG00000031544 |  |  |  |
| ENSBTAG00000031548 |  |  |  |
| ENSBTAG00000031551 |  |  |  |
| ENSBTAG00000031553 |  |  |  |
| ENSBTAG00000031561 |  |  |  |
| ENSBTAG00000031567 |  |  |  |
| ENSBTAG00000031569 |  |  |  |
| ENSBTAG00000031572 |  |  |  |
| ENSBTAG00000031575 |  |  |  |
| ENSBTAG00000031590 |  |  |  |
| ENSBTAG00000031598 |  |  |  |
| ENSBTAG00000031599 |  |  |  |
| ENSBTAG00000031614 |  |  |  |
| ENSBTAG00000031632 |  |  |  |
| ENSBTAG00000031647 |  |  |  |
| ENSBTAG00000031656 |  |  |  |
| ENSBTAG00000031679 |  |  |  |
| ENSBTAG00000031686 |  |  |  |
| ENSBTAG00000031688 |  |  |  |
| ENSBTAG00000031697 |  |  |  |
| ENSBTAG00000031701 |  |  |  |
| ENSBTAG00000031704 |  |  |  |
| ENSBTAG00000031707 |  |  |  |
| ENSBTAG00000031709 |  |  |  |
| ENSBTAG00000031715 |  |  |  |
| ENSBTAG00000031716 |  |  |  |
| ENSBTAG00000031718 |  |  |  |
| ENSBTAG00000031725 |  |  |  |
| ENSBTAG00000031731 |  |  |  |
| ENSBTAG00000031747 |  |  |  |
| ENSBTAG00000031749 |  |  |  |
| ENSBTAG00000031765 |  |  |  |
| ENSBTAG00000031774 |  |  |  |
| ENSBTAG00000031777 |  |  |  |
| ENSBTAG00000031778 |  |  |  |
| ENSBTAG00000031785 |  |  |  |
| ENSBTAG00000031788 |  |  |  |
| ENSBTAG00000031789 |  |  |  |
| ENSBTAG00000031792 |  |  |  |
| ENSBTAG00000031795 |  |  |  |
| ENSBTAG00000031797 |  |  |  |
| ENSBTAG00000031806 |  |  |  |
| ENSBTAG00000031814 |  |  |  |
| ENSBTAG00000031824 |  |  |  |
| ENSBTAG00000031828 |  |  |  |
| ENSBTAG00000031829 |  |  |  |
| ENSBTAG00000031845 |  |  |  |
| ENSBTAG00000031846 |  |  |  |
| ENSBTAG00000031849 |  |  |  |
| ENSBTAG00000031851 |  |  |  |
| ENSBTAG00000031861 |  |  |  |
| ENSBTAG00000031863 |  |  |  |
| ENSBTAG00000031871 |  |  |  |
| ENSBTAG00000031873 |  |  |  |
| ENSBTAG00000031875 |  |  |  |
| ENSBTAG00000031885 |  |  |  |
| ENSBTAG00000031886 |  |  |  |
| ENSBTAG00000031890 |  |  |  |
| ENSBTAG00000031895 |  |  |  |
| ENSBTAG00000031898 |  |  |  |
| ENSBTAG00000031916 |  |  |  |
| ENSBTAG00000031919 |  |  |  |
| ENSBTAG00000031940 |  |  |  |
| ENSBTAG00000031941 |  |  |  |
| ENSBTAG00000031967 |  |  |  |
| ENSBTAG00000031981 |  |  |  |
| ENSBTAG00000031998 |  |  |  |
| ENSBTAG00000032014 |  |  |  |
| ENSBTAG00000032018 |  |  |  |
| ENSBTAG00000032021 |  |  |  |
| ENSBTAG00000032022 |  |  |  |
| ENSBTAG00000032026 |  |  |  |
| ENSBTAG00000032047 |  |  |  |
| ENSBTAG00000032051 |  |  |  |
| ENSBTAG00000032055 |  |  |  |
| ENSBTAG00000032059 |  |  |  |
| ENSBTAG00000032068 |  |  |  |
| ENSBTAG00000032071 |  |  |  |
| ENSBTAG00000032084 |  |  |  |
| ENSBTAG00000032087 |  |  |  |
| ENSBTAG00000032089 |  |  |  |
| ENSBTAG00000032092 |  |  |  |
| ENSBTAG00000032097 |  |  |  |
| ENSBTAG00000032132 |  |  |  |
| ENSBTAG00000032137 |  |  |  |
| ENSBTAG00000032148 |  |  |  |
| ENSBTAG00000032152 |  |  |  |
| ENSBTAG00000032163 |  |  |  |
| ENSBTAG00000032200 |  |  |  |
| ENSBTAG00000032223 |  |  |  |
| ENSBTAG00000032234 |  |  |  |
| ENSBTAG00000032236 |  |  |  |
| ENSBTAG00000032247 |  |  |  |
| ENSBTAG00000032260 |  |  |  |
| ENSBTAG00000032288 |  |  |  |
| ENSBTAG00000032304 |  |  |  |
| ENSBTAG00000032331 |  |  |  |
| ENSBTAG00000032340 |  |  |  |
| ENSBTAG00000032369 |  |  |  |
| ENSBTAG00000032372 |  |  |  |
| ENSBTAG00000032374 |  |  |  |
| ENSBTAG00000032396 |  |  |  |
| ENSBTAG00000032424 |  |  |  |
| ENSBTAG00000032425 |  |  |  |
| ENSBTAG00000032427 |  |  |  |
| ENSBTAG00000032433 |  |  |  |
| ENSBTAG00000032453 |  |  |  |
| ENSBTAG00000032477 |  |  |  |
| ENSBTAG00000032515 |  |  |  |
| ENSBTAG00000032518 |  |  |  |
| ENSBTAG00000032534 |  |  |  |
| ENSBTAG00000032548 |  |  |  |
| ENSBTAG00000032591 |  |  |  |
| ENSBTAG00000032617 |  |  |  |
| ENSBTAG00000032640 |  |  |  |
| ENSBTAG00000032642 |  |  |  |
| ENSBTAG00000032651 |  |  |  |
| ENSBTAG00000032660 |  |  |  |
| ENSBTAG00000032674 |  |  |  |
| ENSBTAG00000032680 |  |  |  |
| ENSBTAG00000032704 |  |  |  |
| ENSBTAG00000032705 |  |  |  |
| ENSBTAG00000032733 |  |  |  |
| ENSBTAG00000032738 |  |  |  |
| ENSBTAG00000032763 |  |  |  |
| ENSBTAG00000032775 |  |  |  |
| ENSBTAG00000032808 |  |  |  |
| ENSBTAG00000032819 |  |  |  |
| ENSBTAG00000032821 |  |  |  |
| ENSBTAG00000032829 |  |  |  |
| ENSBTAG00000032844 |  |  |  |
| ENSBTAG00000032848 |  |  |  |
| ENSBTAG00000032852 |  |  |  |
| ENSBTAG00000032884 |  |  |  |
| ENSBTAG00000032902 |  |  |  |
| ENSBTAG00000032908 |  |  |  |
| ENSBTAG00000032914 |  |  |  |
| ENSBTAG00000032918 |  |  |  |
| ENSBTAG00000032951 |  |  |  |
| ENSBTAG00000032961 |  |  |  |
| ENSBTAG00000032962 |  |  |  |
| ENSBTAG00000032964 |  |  |  |
| ENSBTAG00000032979 |  |  |  |
| ENSBTAG00000032982 |  |  |  |
| ENSBTAG00000032995 |  |  |  |
| ENSBTAG00000032996 |  |  |  |
| ENSBTAG00000032997 |  |  |  |
| ENSBTAG00000033008 |  |  |  |
| ENSBTAG00000033015 |  |  |  |
| ENSBTAG00000033032 |  |  |  |
| ENSBTAG00000033056 |  |  |  |
| ENSBTAG00000033076 |  |  |  |
| ENSBTAG00000033077 |  |  |  |
| ENSBTAG00000033078 |  |  |  |
| ENSBTAG00000033080 |  |  |  |
| ENSBTAG00000033096 |  |  |  |
| ENSBTAG00000033117 |  |  |  |
| ENSBTAG00000033136 |  |  |  |
| ENSBTAG00000033137 |  |  |  |
| ENSBTAG00000033160 |  |  |  |
| ENSBTAG00000033170 |  |  |  |
| ENSBTAG00000033174 |  |  |  |
| ENSBTAG00000033186 |  |  |  |
| ENSBTAG00000033190 |  |  |  |
| ENSBTAG00000033214 |  |  |  |
| ENSBTAG00000033217 |  |  |  |
| ENSBTAG00000033218 |  |  |  |
| ENSBTAG00000033220 |  |  |  |
| ENSBTAG00000033221 |  |  |  |
| ENSBTAG00000033222 |  |  |  |
| ENSBTAG00000033225 |  |  |  |
| ENSBTAG00000033255 |  |  |  |
| ENSBTAG00000033278 |  |  |  |
| ENSBTAG00000033290 |  |  |  |
| ENSBTAG00000033292 |  |  |  |
| ENSBTAG00000033298 |  |  |  |
| ENSBTAG00000033304 |  |  |  |
| ENSBTAG00000033313 |  |  |  |
| ENSBTAG00000033319 |  |  |  |
| ENSBTAG00000033326 |  |  |  |
| ENSBTAG00000033330 |  |  |  |
| ENSBTAG00000033331 |  |  |  |
| ENSBTAG00000033333 |  |  |  |
| ENSBTAG00000033334 |  |  |  |
| ENSBTAG00000033339 |  |  |  |
| ENSBTAG00000033345 |  |  |  |
| ENSBTAG00000033351 |  |  |  |
| ENSBTAG00000033367 |  |  |  |
| ENSBTAG00000033395 |  |  |  |
| ENSBTAG00000033396 |  |  |  |
| ENSBTAG00000033397 |  |  |  |
| ENSBTAG00000033412 |  |  |  |
| ENSBTAG00000033423 |  |  |  |
| ENSBTAG00000033441 |  |  |  |
| ENSBTAG00000033445 |  |  |  |
| ENSBTAG00000033453 |  |  |  |
| ENSBTAG00000033460 |  |  |  |
| ENSBTAG00000033464 |  |  |  |
| ENSBTAG00000033486 |  |  |  |
| ENSBTAG00000033504 |  |  |  |
| ENSBTAG00000033510 |  |  |  |
| ENSBTAG00000033529 |  |  |  |
| ENSBTAG00000033535 |  |  |  |
| ENSBTAG00000033543 |  |  |  |
| ENSBTAG00000033580 |  |  |  |
| ENSBTAG00000033603 |  |  |  |
| ENSBTAG00000033621 |  |  |  |
| ENSBTAG00000033677 |  |  |  |
| ENSBTAG00000033679 |  |  |  |
| ENSBTAG00000033680 |  |  |  |
| ENSBTAG00000033690 |  |  |  |
| ENSBTAG00000033699 |  |  |  |
| ENSBTAG00000033721 |  |  |  |
| ENSBTAG00000033731 |  |  |  |
| ENSBTAG00000033747 |  |  |  |
| ENSBTAG00000033803 |  |  |  |
| ENSBTAG00000033806 |  |  |  |
| ENSBTAG00000033835 |  |  |  |
| ENSBTAG00000033902 |  |  |  |
| ENSBTAG00000033961 |  |  |  |
| ENSBTAG00000033983 |  |  |  |
| ENSBTAG00000034069 |  |  |  |
| ENSBTAG00000034106 |  |  |  |
| ENSBTAG00000034138 |  |  |  |
| ENSBTAG00000034139 |  |  |  |
| ENSBTAG00000034147 |  |  |  |
| ENSBTAG00000034154 |  |  |  |
| ENSBTAG00000034184 |  |  |  |
| ENSBTAG00000034185 |  |  |  |
| ENSBTAG00000034192 |  |  |  |
| ENSBTAG00000034206 |  |  |  |
| ENSBTAG00000034255 |  |  |  |
| ENSBTAG00000034295 |  |  |  |
| ENSBTAG00000034323 |  |  |  |
| ENSBTAG00000034347 |  |  |  |
| ENSBTAG00000034366 |  |  |  |
| ENSBTAG00000034384 |  |  |  |
| ENSBTAG00000034385 |  |  |  |
| ENSBTAG00000034393 |  |  |  |
| ENSBTAG00000034402 |  |  |  |
| ENSBTAG00000034436 |  |  |  |
| ENSBTAG00000034441 |  |  |  |
| ENSBTAG00000034442 |  |  |  |
| ENSBTAG00000034495 |  |  |  |
| ENSBTAG00000034501 |  |  |  |
| ENSBTAG00000034519 |  |  |  |
| ENSBTAG00000034522 |  |  |  |
| ENSBTAG00000034529 |  |  |  |
| ENSBTAG00000034531 |  |  |  |
| ENSBTAG00000034586 |  |  |  |
| ENSBTAG00000034598 |  |  |  |
| ENSBTAG00000034613 |  |  |  |
| ENSBTAG00000034633 |  |  |  |
| ENSBTAG00000034645 |  |  |  |
| ENSBTAG00000034676 |  |  |  |
| ENSBTAG00000034689 |  |  |  |
| ENSBTAG00000034693 |  |  |  |
| ENSBTAG00000034776 |  |  |  |
| ENSBTAG00000034823 |  |  |  |
| ENSBTAG00000034827 |  |  |  |
| ENSBTAG00000034850 |  |  |  |
| ENSBTAG00000034867 |  |  |  |
| ENSBTAG00000034869 |  |  |  |
| ENSBTAG00000034875 |  |  |  |
| ENSBTAG00000034883 |  |  |  |
| ENSBTAG00000034885 |  |  |  |
| ENSBTAG00000034905 |  |  |  |
| ENSBTAG00000034936 |  |  |  |
| ENSBTAG00000034939 |  |  |  |
| ENSBTAG00000034952 |  |  |  |
| ENSBTAG00000034954 |  |  |  |
| ENSBTAG00000034963 |  |  |  |
| ENSBTAG00000034978 |  |  |  |
| ENSBTAG00000034985 |  |  |  |
| ENSBTAG00000035012 |  |  |  |
| ENSBTAG00000035018 |  |  |  |
| ENSBTAG00000035054 |  |  |  |
| ENSBTAG00000035064 |  |  |  |
| ENSBTAG00000035072 |  |  |  |
| ENSBTAG00000035083 |  |  |  |
| ENSBTAG00000035084 |  |  |  |
| ENSBTAG00000035129 |  |  |  |
| ENSBTAG00000035144 |  |  |  |
| ENSBTAG00000035174 |  |  |  |
| ENSBTAG00000035175 |  |  |  |
| ENSBTAG00000035226 |  |  |  |
| ENSBTAG00000035244 |  |  |  |
| ENSBTAG00000035247 |  |  |  |
| ENSBTAG00000035265 |  |  |  |
| ENSBTAG00000035286 |  |  |  |
| ENSBTAG00000035319 |  |  |  |
| ENSBTAG00000035323 |  |  |  |
| ENSBTAG00000035370 |  |  |  |
| ENSBTAG00000035556 |  |  |  |
| ENSBTAG00000035587 |  |  |  |
| ENSBTAG00000035643 |  |  |  |
| ENSBTAG00000035654 |  |  |  |
| ENSBTAG00000035696 |  |  |  |
| ENSBTAG00000035708 |  |  |  |
| ENSBTAG00000035785 |  |  |  |
| ENSBTAG00000035827 |  |  |  |
| ENSBTAG00000035844 |  |  |  |
| ENSBTAG00000035858 |  |  |  |
| ENSBTAG00000035868 |  |  |  |
| ENSBTAG00000035907 |  |  |  |
| ENSBTAG00000035995 |  |  |  |
| ENSBTAG00000035998 |  |  |  |
| ENSBTAG00000036028 |  |  |  |
| ENSBTAG00000036099 |  |  |  |
| ENSBTAG00000036101 |  |  |  |
| ENSBTAG00000036116 |  |  |  |
| ENSBTAG00000036183 |  |  |  |
| ENSBTAG00000036222 |  |  |  |
| ENSBTAG00000036224 |  |  |  |
| ENSBTAG00000036260 |  |  |  |
| ENSBTAG00000036262 |  |  |  |
| ENSBTAG00000036277 |  |  |  |
| ENSBTAG00000036297 |  |  |  |
| ENSBTAG00000036298 |  |  |  |
| ENSBTAG00000037375 |  |  |  |
| ENSBTAG00000037377 |  |  |  |
| ENSBTAG00000037383 |  |  |  |
| ENSBTAG00000037389 |  |  |  |
| ENSBTAG00000037400 |  |  |  |
| ENSBTAG00000037465 |  |  |  |
| ENSBTAG00000037470 |  |  |  |
| ENSBTAG00000037483 |  |  |  |
| ENSBTAG00000037489 |  |  |  |
| ENSBTAG00000037527 |  |  |  |
| ENSBTAG00000037533 |  |  |  |
| ENSBTAG00000037539 |  |  |  |
| ENSBTAG00000037549 |  |  |  |
| ENSBTAG00000037553 |  |  |  |
| ENSBTAG00000037558 |  |  |  |
| ENSBTAG00000037559 |  |  |  |
| ENSBTAG00000037560 |  |  |  |
| ENSBTAG00000037566 |  |  |  |
| ENSBTAG00000037571 |  |  |  |
| ENSBTAG00000037602 |  |  |  |
| ENSBTAG00000037605 |  |  |  |
| ENSBTAG00000037638 |  |  |  |
| ENSBTAG00000037640 |  |  |  |
| ENSBTAG00000037644 |  |  |  |
| ENSBTAG00000037673 |  |  |  |
| ENSBTAG00000037686 |  |  |  |
| ENSBTAG00000037687 |  |  |  |
| ENSBTAG00000037702 |  |  |  |
| ENSBTAG00000037703 |  |  |  |
| ENSBTAG00000037726 |  |  |  |
| ENSBTAG00000037728 |  |  |  |
| ENSBTAG00000037729 |  |  |  |
| ENSBTAG00000037735 |  |  |  |
| ENSBTAG00000037764 |  |  |  |
| ENSBTAG00000037765 |  |  |  |
| ENSBTAG00000037778 |  |  |  |
| ENSBTAG00000037784 |  |  |  |
| ENSBTAG00000037791 |  |  |  |
| ENSBTAG00000037795 |  |  |  |
| ENSBTAG00000037799 |  |  |  |
| ENSBTAG00000037800 |  |  |  |
| ENSBTAG00000037804 |  |  |  |
| ENSBTAG00000037811 |  |  |  |
| ENSBTAG00000037813 |  |  |  |
| ENSBTAG00000037819 |  |  |  |
| ENSBTAG00000037824 |  |  |  |
| ENSBTAG00000037826 |  |  |  |
| ENSBTAG00000037844 |  |  |  |
| ENSBTAG00000037856 |  |  |  |
| ENSBTAG00000037858 |  |  |  |
| ENSBTAG00000037883 |  |  |  |
| ENSBTAG00000037896 |  |  |  |
| ENSBTAG00000037917 |  |  |  |
| ENSBTAG00000037949 |  |  |  |
| ENSBTAG00000037964 |  |  |  |
| ENSBTAG00000037972 |  |  |  |
| ENSBTAG00000037980 |  |  |  |
| ENSBTAG00000037986 |  |  |  |
| ENSBTAG00000037988 |  |  |  |
| ENSBTAG00000037989 |  |  |  |
| ENSBTAG00000037991 |  |  |  |
| ENSBTAG00000037996 |  |  |  |
| ENSBTAG00000038011 |  |  |  |
| ENSBTAG00000038025 |  |  |  |
| ENSBTAG00000038033 |  |  |  |
| ENSBTAG00000038047 |  |  |  |
| ENSBTAG00000038050 |  |  |  |
| ENSBTAG00000038058 |  |  |  |
| ENSBTAG00000038062 |  |  |  |
| ENSBTAG00000038067 |  |  |  |
| ENSBTAG00000038074 |  |  |  |
| ENSBTAG00000038079 |  |  |  |
| ENSBTAG00000038085 |  |  |  |
| ENSBTAG00000038093 |  |  |  |
| ENSBTAG00000038104 |  |  |  |
| ENSBTAG00000038112 |  |  |  |
| ENSBTAG00000038115 |  |  |  |
| ENSBTAG00000038116 |  |  |  |
| ENSBTAG00000038117 |  |  |  |
| ENSBTAG00000038126 |  |  |  |
| ENSBTAG00000038131 |  |  |  |
| ENSBTAG00000038132 |  |  |  |
| ENSBTAG00000038139 |  |  |  |
| ENSBTAG00000038149 |  |  |  |
| ENSBTAG00000038151 |  |  |  |
| ENSBTAG00000038156 |  |  |  |
| ENSBTAG00000038173 |  |  |  |
| ENSBTAG00000038180 |  |  |  |
| ENSBTAG00000038181 |  |  |  |
| ENSBTAG00000038186 |  |  |  |
| ENSBTAG00000038189 |  |  |  |
| ENSBTAG00000038195 |  |  |  |
| ENSBTAG00000038215 |  |  |  |
| ENSBTAG00000038221 |  |  |  |
| ENSBTAG00000038228 |  |  |  |
| ENSBTAG00000038235 |  |  |  |
| ENSBTAG00000038238 |  |  |  |
| ENSBTAG00000038263 |  |  |  |
| ENSBTAG00000038268 |  |  |  |
| ENSBTAG00000038283 |  |  |  |
| ENSBTAG00000038286 |  |  |  |
| ENSBTAG00000038291 |  |  |  |
| ENSBTAG00000038312 |  |  |  |
| ENSBTAG00000038321 |  |  |  |
| ENSBTAG00000038325 |  |  |  |
| ENSBTAG00000038330 |  |  |  |
| ENSBTAG00000038333 |  |  |  |
| ENSBTAG00000038340 |  |  |  |
| ENSBTAG00000038347 |  |  |  |
| ENSBTAG00000038375 |  |  |  |
| ENSBTAG00000038381 |  |  |  |
| ENSBTAG00000038384 |  |  |  |
| ENSBTAG00000038386 |  |  |  |
| ENSBTAG00000038415 |  |  |  |
| ENSBTAG00000038438 |  |  |  |
| ENSBTAG00000038439 |  |  |  |
| ENSBTAG00000038461 |  |  |  |
| ENSBTAG00000038462 |  |  |  |
| ENSBTAG00000038464 |  |  |  |
| ENSBTAG00000038477 |  |  |  |
| ENSBTAG00000038480 |  |  |  |
| ENSBTAG00000038488 |  |  |  |
| ENSBTAG00000038495 |  |  |  |
| ENSBTAG00000038496 |  |  |  |
| ENSBTAG00000038497 |  |  |  |
| ENSBTAG00000038536 |  |  |  |
| ENSBTAG00000038577 |  |  |  |
| ENSBTAG00000038584 |  |  |  |
| ENSBTAG00000038619 |  |  |  |
| ENSBTAG00000038635 |  |  |  |
| ENSBTAG00000038640 |  |  |  |
| ENSBTAG00000038652 |  |  |  |
| ENSBTAG00000038660 |  |  |  |
| ENSBTAG00000038662 |  |  |  |
| ENSBTAG00000038674 |  |  |  |
| ENSBTAG00000038696 |  |  |  |
| ENSBTAG00000038698 |  |  |  |
| ENSBTAG00000038700 |  |  |  |
| ENSBTAG00000038706 |  |  |  |
| ENSBTAG00000038737 |  |  |  |
| ENSBTAG00000038738 |  |  |  |
| ENSBTAG00000038745 |  |  |  |
| ENSBTAG00000038756 |  |  |  |
| ENSBTAG00000038759 |  |  |  |
| ENSBTAG00000038770 |  |  |  |
| ENSBTAG00000038783 |  |  |  |
| ENSBTAG00000038795 |  |  |  |
| ENSBTAG00000038806 |  |  |  |
| ENSBTAG00000038810 |  |  |  |
| ENSBTAG00000038815 |  |  |  |
| ENSBTAG00000038831 |  |  |  |
| ENSBTAG00000038854 |  |  |  |
| ENSBTAG00000038865 |  |  |  |
| ENSBTAG00000038866 |  |  |  |
| ENSBTAG00000038869 |  |  |  |
| ENSBTAG00000038878 |  |  |  |
| ENSBTAG00000038888 |  |  |  |
| ENSBTAG00000038893 |  |  |  |
| ENSBTAG00000038896 |  |  |  |
| ENSBTAG00000038904 |  |  |  |
| ENSBTAG00000038910 |  |  |  |
| ENSBTAG00000038918 |  |  |  |
| ENSBTAG00000038920 |  |  |  |
| ENSBTAG00000038929 |  |  |  |
| ENSBTAG00000038931 |  |  |  |
| ENSBTAG00000038955 |  |  |  |
| ENSBTAG00000038966 |  |  |  |
| ENSBTAG00000038974 |  |  |  |
| ENSBTAG00000038979 |  |  |  |
| ENSBTAG00000038990 |  |  |  |
| ENSBTAG00000039015 |  |  |  |
| ENSBTAG00000039028 |  |  |  |
| ENSBTAG00000039046 |  |  |  |
| ENSBTAG00000039050 |  |  |  |
| ENSBTAG00000039055 |  |  |  |
| ENSBTAG00000039056 |  |  |  |
| ENSBTAG00000039068 |  |  |  |
| ENSBTAG00000039071 |  |  |  |
| ENSBTAG00000039075 |  |  |  |
| ENSBTAG00000039077 |  |  |  |
| ENSBTAG00000039080 |  |  |  |
| ENSBTAG00000039090 |  |  |  |
| ENSBTAG00000039091 |  |  |  |
| ENSBTAG00000039105 |  |  |  |
| ENSBTAG00000039122 |  |  |  |
| ENSBTAG00000039129 |  |  |  |
| ENSBTAG00000039153 |  |  |  |
| ENSBTAG00000039157 |  |  |  |
| ENSBTAG00000039161 |  |  |  |
| ENSBTAG00000039163 |  |  |  |
| ENSBTAG00000039172 |  |  |  |
| ENSBTAG00000039196 |  |  |  |
| ENSBTAG00000039212 |  |  |  |
| ENSBTAG00000039223 |  |  |  |
| ENSBTAG00000039231 |  |  |  |
| ENSBTAG00000039246 |  |  |  |
| ENSBTAG00000039275 |  |  |  |
| ENSBTAG00000039287 |  |  |  |
| ENSBTAG00000039302 |  |  |  |
| ENSBTAG00000039307 |  |  |  |
| ENSBTAG00000039313 |  |  |  |
| ENSBTAG00000039325 |  |  |  |
| ENSBTAG00000039326 |  |  |  |
| ENSBTAG00000039335 |  |  |  |
| ENSBTAG00000039340 |  |  |  |
| ENSBTAG00000039355 |  |  |  |
| ENSBTAG00000039366 |  |  |  |
| ENSBTAG00000039374 |  |  |  |
| ENSBTAG00000039384 |  |  |  |
| ENSBTAG00000039391 |  |  |  |
| ENSBTAG00000039397 |  |  |  |
| ENSBTAG00000039415 |  |  |  |
| ENSBTAG00000039425 |  |  |  |
| ENSBTAG00000039435 |  |  |  |
| ENSBTAG00000039456 |  |  |  |
| ENSBTAG00000039462 |  |  |  |
| ENSBTAG00000039483 |  |  |  |
| ENSBTAG00000039486 |  |  |  |
| ENSBTAG00000039493 |  |  |  |
| ENSBTAG00000039509 |  |  |  |
| ENSBTAG00000039513 |  |  |  |
| ENSBTAG00000039520 |  |  |  |
| ENSBTAG00000039523 |  |  |  |
| ENSBTAG00000039529 |  |  |  |
| ENSBTAG00000039540 |  |  |  |
| ENSBTAG00000039552 |  |  |  |
| ENSBTAG00000039556 |  |  |  |
| ENSBTAG00000039571 |  |  |  |
| ENSBTAG00000039573 |  |  |  |
| ENSBTAG00000039574 |  |  |  |
| ENSBTAG00000039581 |  |  |  |
| ENSBTAG00000039591 |  |  |  |
| ENSBTAG00000039597 |  |  |  |
| ENSBTAG00000039599 |  |  |  |
| ENSBTAG00000039602 |  |  |  |
| ENSBTAG00000039618 |  |  |  |
| ENSBTAG00000039620 |  |  |  |
| ENSBTAG00000039634 |  |  |  |
| ENSBTAG00000039635 |  |  |  |
| ENSBTAG00000039644 |  |  |  |
| ENSBTAG00000039657 |  |  |  |
| ENSBTAG00000039658 |  |  |  |
| ENSBTAG00000039684 |  |  |  |
| ENSBTAG00000039686 |  |  |  |
| ENSBTAG00000039688 |  |  |  |
| ENSBTAG00000039695 |  |  |  |
| ENSBTAG00000039696 |  |  |  |
| ENSBTAG00000039705 |  |  |  |
| ENSBTAG00000039714 |  |  |  |
| ENSBTAG00000039718 |  |  |  |
| ENSBTAG00000039727 |  |  |  |
| ENSBTAG00000039731 |  |  |  |
| ENSBTAG00000039732 |  |  |  |
| ENSBTAG00000039738 |  |  |  |
| ENSBTAG00000039740 |  |  |  |
| ENSBTAG00000039765 |  |  |  |
| ENSBTAG00000039770 |  |  |  |
| ENSBTAG00000039784 |  |  |  |
| ENSBTAG00000039787 |  |  |  |
| ENSBTAG00000039794 |  |  |  |
| ENSBTAG00000039803 |  |  |  |
| ENSBTAG00000039812 |  |  |  |
| ENSBTAG00000039815 |  |  |  |
| ENSBTAG00000039819 |  |  |  |
| ENSBTAG00000039820 |  |  |  |
| ENSBTAG00000039839 |  |  |  |
| ENSBTAG00000039851 |  |  |  |
| ENSBTAG00000039855 |  |  |  |
| ENSBTAG00000039886 |  |  |  |
| ENSBTAG00000039891 |  |  |  |
| ENSBTAG00000039916 |  |  |  |
| ENSBTAG00000039928 |  |  |  |
| ENSBTAG00000039950 |  |  |  |
| ENSBTAG00000039951 |  |  |  |
| ENSBTAG00000039958 |  |  |  |
| ENSBTAG00000039967 |  |  |  |
| ENSBTAG00000039968 |  |  |  |
| ENSBTAG00000039970 |  |  |  |
| ENSBTAG00000039991 |  |  |  |
| ENSBTAG00000040005 |  |  |  |
| ENSBTAG00000040006 |  |  |  |
| ENSBTAG00000040028 |  |  |  |
| ENSBTAG00000040031 |  |  |  |
| ENSBTAG00000040034 |  |  |  |
| ENSBTAG00000040043 |  |  |  |
| ENSBTAG00000040055 |  |  |  |
| ENSBTAG00000040058 |  |  |  |
| ENSBTAG00000040065 |  |  |  |
| ENSBTAG00000040082 |  |  |  |
| ENSBTAG00000040106 |  |  |  |
| ENSBTAG00000040111 |  |  |  |
| ENSBTAG00000040131 |  |  |  |
| ENSBTAG00000040147 |  |  |  |
| ENSBTAG00000040151 |  |  |  |
| ENSBTAG00000040169 |  |  |  |
| ENSBTAG00000040188 |  |  |  |
| ENSBTAG00000040190 |  |  |  |
| ENSBTAG00000040193 |  |  |  |
| ENSBTAG00000040199 |  |  |  |
| ENSBTAG00000040202 |  |  |  |
| ENSBTAG00000040208 |  |  |  |
| ENSBTAG00000040226 |  |  |  |
| ENSBTAG00000040244 |  |  |  |
| ENSBTAG00000040291 |  |  |  |
| ENSBTAG00000040295 |  |  |  |
| ENSBTAG00000040296 |  |  |  |
| ENSBTAG00000040316 |  |  |  |
| ENSBTAG00000040321 |  |  |  |
| ENSBTAG00000040323 |  |  |  |
| ENSBTAG00000040333 |  |  |  |
| ENSBTAG00000040336 |  |  |  |
| ENSBTAG00000040340 |  |  |  |
| ENSBTAG00000040360 |  |  |  |
| ENSBTAG00000040361 |  |  |  |
| ENSBTAG00000040367 |  |  |  |
| ENSBTAG00000040368 |  |  |  |
| ENSBTAG00000040381 |  |  |  |
| ENSBTAG00000040388 |  |  |  |
| ENSBTAG00000040392 |  |  |  |
| ENSBTAG00000040422 |  |  |  |
| ENSBTAG00000040432 |  |  |  |
| ENSBTAG00000040435 |  |  |  |
| ENSBTAG00000040460 |  |  |  |
| ENSBTAG00000040477 |  |  |  |
| ENSBTAG00000040490 |  |  |  |
| ENSBTAG00000040512 |  |  |  |
| ENSBTAG00000040569 |  |  |  |
| ENSBTAG00000040575 |  |  |  |
| ENSBTAG00000040580 |  |  |  |
| ENSBTAG00000040598 |  |  |  |
| ENSBTAG00000040602 |  |  |  |
| ENSBTAG00000040607 |  |  |  |
| ENSBTAG00000043553 |  |  |  |
| ENSBTAG00000043581 |  |  |  |
| ENSBTAG00000043951 |  |  |  |
| ENSBTAG00000043954 |  |  |  |
| ENSBTAG00000043961 |  |  |  |
| ENSBTAG00000043964 |  |  |  |
| ENSBTAG00000043972 |  |  |  |
| ENSBTAG00000043981 |  |  |  |
| ENSBTAG00000043989 |  |  |  |
| ENSBTAG00000043993 |  |  |  |
| ENSBTAG00000043994 |  |  |  |
| ENSBTAG00000043996 |  |  |  |
| ENSBTAG00000043999 |  |  |  |
| ENSBTAG00000044000 |  |  |  |
| ENSBTAG00000044007 |  |  |  |
| ENSBTAG00000044015 |  |  |  |
| ENSBTAG00000044019 |  |  |  |
| ENSBTAG00000044029 |  |  |  |
| ENSBTAG00000044032 |  |  |  |
| ENSBTAG00000044043 |  |  |  |
| ENSBTAG00000044046 |  |  |  |
| ENSBTAG00000044053 |  |  |  |
| ENSBTAG00000044056 |  |  |  |
| ENSBTAG00000044058 |  |  |  |
| ENSBTAG00000044062 |  |  |  |
| ENSBTAG00000044063 |  |  |  |
| ENSBTAG00000044068 |  |  |  |
| ENSBTAG00000044075 |  |  |  |
| ENSBTAG00000044092 |  |  |  |
| ENSBTAG00000044099 |  |  |  |
| ENSBTAG00000044100 |  |  |  |
| ENSBTAG00000044106 |  |  |  |
| ENSBTAG00000044123 |  |  |  |
| ENSBTAG00000044129 |  |  |  |
| ENSBTAG00000044135 |  |  |  |
| ENSBTAG00000044150 |  |  |  |
| ENSBTAG00000044169 |  |  |  |
| ENSBTAG00000044171 |  |  |  |
| ENSBTAG00000044175 |  |  |  |
| ENSBTAG00000044178 |  |  |  |
| ENSBTAG00000044183 |  |  |  |
| ENSBTAG00000044184 |  |  |  |
| ENSBTAG00000044185 |  |  |  |
| ENSBTAG00000044194 |  |  |  |
| ENSBTAG00000044202 |  |  |  |
| ENSBTAG00000044207 |  |  |  |
| ENSBTAG00000045510 |  |  |  |
| ENSBTAG00000045520 |  |  |  |
| ENSBTAG00000045535 |  |  |  |
| ENSBTAG00000045538 |  |  |  |
| ENSBTAG00000045548 |  |  |  |
| ENSBTAG00000045550 |  |  |  |
| ENSBTAG00000045566 |  |  |  |
| ENSBTAG00000045567 |  |  |  |
| ENSBTAG00000045578 |  |  |  |
| ENSBTAG00000045582 |  |  |  |
| ENSBTAG00000045588 |  |  |  |
| ENSBTAG00000045592 |  |  |  |
| ENSBTAG00000045604 |  |  |  |
| ENSBTAG00000045628 |  |  |  |
| ENSBTAG00000045649 |  |  |  |
| ENSBTAG00000045653 |  |  |  |
| ENSBTAG00000045664 |  |  |  |
| ENSBTAG00000045677 |  |  |  |
| ENSBTAG00000045678 |  |  |  |
| ENSBTAG00000045697 |  |  |  |
| ENSBTAG00000045704 |  |  |  |
| ENSBTAG00000045711 |  |  |  |
| ENSBTAG00000045728 |  |  |  |
| ENSBTAG00000045732 |  |  |  |
| ENSBTAG00000045742 |  |  |  |
| ENSBTAG00000045744 |  |  |  |
| ENSBTAG00000045748 |  |  |  |
| ENSBTAG00000045750 |  |  |  |
| ENSBTAG00000045754 |  |  |  |
| ENSBTAG00000045757 |  |  |  |
| ENSBTAG00000045762 |  |  |  |
| ENSBTAG00000045767 |  |  |  |
| ENSBTAG00000045773 |  |  |  |
| ENSBTAG00000045776 |  |  |  |
| ENSBTAG00000045779 |  |  |  |
| ENSBTAG00000045785 |  |  |  |
| ENSBTAG00000045794 |  |  |  |
| ENSBTAG00000045805 |  |  |  |
| ENSBTAG00000045828 |  |  |  |
| ENSBTAG00000045834 |  |  |  |
| ENSBTAG00000045857 |  |  |  |
| ENSBTAG00000045887 |  |  |  |
| ENSBTAG00000045895 |  |  |  |
| ENSBTAG00000045896 |  |  |  |
| ENSBTAG00000045901 |  |  |  |
| ENSBTAG00000045902 |  |  |  |
| ENSBTAG00000045904 |  |  |  |
| ENSBTAG00000045929 |  |  |  |
| ENSBTAG00000045931 |  |  |  |
| ENSBTAG00000045943 |  |  |  |
| ENSBTAG00000045946 |  |  |  |
| ENSBTAG00000045955 |  |  |  |
| ENSBTAG00000045967 |  |  |  |
| ENSBTAG00000045969 |  |  |  |
| ENSBTAG00000045971 |  |  |  |
| ENSBTAG00000045987 |  |  |  |
| ENSBTAG00000046005 |  |  |  |
| ENSBTAG00000046014 |  |  |  |
| ENSBTAG00000046019 |  |  |  |
| ENSBTAG00000046024 |  |  |  |
| ENSBTAG00000046031 |  |  |  |
| ENSBTAG00000046037 |  |  |  |
| ENSBTAG00000046046 |  |  |  |
| ENSBTAG00000046054 |  |  |  |
| ENSBTAG00000046073 |  |  |  |
| ENSBTAG00000046075 |  |  |  |
| ENSBTAG00000046080 |  |  |  |
| ENSBTAG00000046089 |  |  |  |
| ENSBTAG00000046092 |  |  |  |
| ENSBTAG00000046095 |  |  |  |
| ENSBTAG00000046117 |  |  |  |
| ENSBTAG00000046121 |  |  |  |
| ENSBTAG00000046158 |  |  |  |
| ENSBTAG00000046160 |  |  |  |
| ENSBTAG00000046166 |  |  |  |
| ENSBTAG00000046172 |  |  |  |
| ENSBTAG00000046173 |  |  |  |
| ENSBTAG00000046176 |  |  |  |
| ENSBTAG00000046218 |  |  |  |
| ENSBTAG00000046248 |  |  |  |
| ENSBTAG00000046250 |  |  |  |
| ENSBTAG00000046257 |  |  |  |
| ENSBTAG00000046264 |  |  |  |
| ENSBTAG00000046266 |  |  |  |
| ENSBTAG00000046273 |  |  |  |
| ENSBTAG00000046282 |  |  |  |
| ENSBTAG00000046286 |  |  |  |
| ENSBTAG00000046295 |  |  |  |
| ENSBTAG00000046303 |  |  |  |
| ENSBTAG00000046308 |  |  |  |
| ENSBTAG00000046338 |  |  |  |
| ENSBTAG00000046339 |  |  |  |
| ENSBTAG00000046358 |  |  |  |
| ENSBTAG00000046359 |  |  |  |
| ENSBTAG00000046375 |  |  |  |
| ENSBTAG00000046385 |  |  |  |
| ENSBTAG00000046389 |  |  |  |
| ENSBTAG00000046391 |  |  |  |
| ENSBTAG00000046394 |  |  |  |
| ENSBTAG00000046406 |  |  |  |
| ENSBTAG00000046415 |  |  |  |
| ENSBTAG00000046447 |  |  |  |
| ENSBTAG00000046456 |  |  |  |
| ENSBTAG00000046467 |  |  |  |
| ENSBTAG00000046478 |  |  |  |
| ENSBTAG00000046484 |  |  |  |
| ENSBTAG00000046485 |  |  |  |
| ENSBTAG00000046486 |  |  |  |
| ENSBTAG00000046493 |  |  |  |
| ENSBTAG00000046500 |  |  |  |
| ENSBTAG00000046511 |  |  |  |
| ENSBTAG00000046512 |  |  |  |
| ENSBTAG00000046519 |  |  |  |
| ENSBTAG00000046520 |  |  |  |
| ENSBTAG00000046526 |  |  |  |
| ENSBTAG00000046533 |  |  |  |
| ENSBTAG00000046542 |  |  |  |
| ENSBTAG00000046544 |  |  |  |
| ENSBTAG00000046545 |  |  |  |
| ENSBTAG00000046548 |  |  |  |
| ENSBTAG00000046561 |  |  |  |
| ENSBTAG00000046580 |  |  |  |
| ENSBTAG00000046584 |  |  |  |
| ENSBTAG00000046586 |  |  |  |
| ENSBTAG00000046587 |  |  |  |
| ENSBTAG00000046588 |  |  |  |
| ENSBTAG00000046607 |  |  |  |
| ENSBTAG00000046609 |  |  |  |
| ENSBTAG00000046612 |  |  |  |
| ENSBTAG00000046614 |  |  |  |
| ENSBTAG00000046628 |  |  |  |
| ENSBTAG00000046644 |  |  |  |
| ENSBTAG00000046653 |  |  |  |
| ENSBTAG00000046670 |  |  |  |
| ENSBTAG00000046672 |  |  |  |
| ENSBTAG00000046720 |  |  |  |
| ENSBTAG00000046724 |  |  |  |
| ENSBTAG00000046730 |  |  |  |
| ENSBTAG00000046746 |  |  |  |
| ENSBTAG00000046750 |  |  |  |
[truncated: 5,216 more chars]
