# Supplementary material for: Specific microRNA library of IFN-τ on bovine endometrial epithelial cells
Source: Oncotarget. 2017 Jun 14;8(37):61487–98. doi: 10.18632/oncotarget.18470 (PMC5617439; doi:10.18632/oncotarget.18470)
Supplement: Supplementary file 7 [file oncotarget-08-61487-s007.doc]

**Supplementary Table 8: GO enrichment of predicted targets (TS *vs.* CS group)**

| **GO_accession** | **Description** | **Type** | ***p*-Value** | **Gene numbers** |
| --- | --- | --- | --- | --- |
| GO:0048037 | cofactor binding | MF | 0.0003124 | 211 |
| GO:0050662 | coenzyme binding | MF | 0.0028157 | 165 |
| GO:0003824 | catalytic activity | MF | 0.0047086 | 3129 |
| GO:0044710 | single-organism metabolic process | BP | 0.0063085 | 1185 |
| GO:0043234 | protein complex | CC | 0.0063085 | 877 |
| GO:0071702 | organic substance transport | BP | 0.0063085 | 493 |
| GO:0030001 | metal ion transport | BP | 0.0073669 | 222 |
| GO:0005737 | cytoplasm | CC | 0.0073669 | 983 |
| GO:0008152 | metabolic process | BP | 0.0073669 | 3558 |
| GO:0006629 | lipid metabolic process | BP | 0.0073669 | 361 |
| GO:0006810 | transport | BP | 0.0073669 | 1234 |
| GO:0051234 | establishment of localization | BP | 0.0073669 | 1234 |
| GO:0044424 | intracellular part | CC | 0.0073669 | 1939 |
| GO:0046873 | Metal ion transmembrane transporter activity | MF | 0.0097236 | 219 |
| GO:0051179 | localization | BP | 0.01124 | 1282 |
| GO:0046915 | transition metal ion transmembrane transporter activity | MF | 0.012658 | 76 |
| GO:0002376 | immune system process | BP | 0.013484 | 18 |
| GO:0006812 | cation transport | BP | 0.016444 | 364 |
| GO:0007264 | small GTPase mediated signal transduction | BP | 0.01666 | 292 |
| GO:0032991 | macromolecular complex | CC | 0.018424 | 1073 |
| GO:0051649 | establishment of localization in cell | BP | 0.022233 | 405 |
| GO:0042612 | MHC class I protein complex | CC | 0.023506 | 4 |
| GO:0044422 | organelle part | CC | 0.024091 | 703 |
| GO:0008009 | chemokine activity | MF | 0.024091 | 25 |
| GO:0042379 | chemokine receptor binding | MF | 0.024091 | 25 |
| GO:0055114 | oxidation-reduction process | BP | 0.025999 | 525 |
| GO:0033036 | macromolecule localization | BP | 0.025999 | 445 |
| GO:0044237 | cellular metabolic process | BP | 0.025999 | 2774 |
| GO:0006633 | fatty acid biosynthetic process | BP | 0.025999 | 33 |
| GO:0051641 | cellular localization | BP | 0.025999 | 445 |
| GO:0072330 | monocarboxylic acid biosynthetic process | BP | 0.025999 | 36 |
| GO:0006631 | fatty acid metabolic process | BP | 0.025999 | 57 |
| GO:0044446 | intracellular organelle part | CC | 0.026508 | 696 |
| GO:0000041 | transition metal ion transport | BP | 0.030778 | 80 |
| GO:0009056 | catabolic process | BP | 0.037664 | 399 |
| GO:0005623 | cell | CC | 0.038234 | 2290 |
| GO:0044464 | cell part | CC | 0.038234 | 2290 |
| GO:0016491 | oxidoreductase activity | MF | 0.038627 | 556 |
| GO:0072511 | divalent inorganic cation transport | BP | 0.038843 | 81 |
| GO:0005381 | iron ion transmembrane transporter activity | MF | 0.038843 | 60 |
| GO:0043412 | macromolecule modification | BP | 0.038843 | 609 |
| GO:0015031 | protein transport | BP | 0.038843 | 372 |
| GO:0045184 | establishment of protein localization | BP | 0.038843 | 372 |
| GO:0016740 | transferase activity | MF | 0.038843 | 1112 |
| GO:0015093 | ferrous iron transmembrane transporter activity | MF | 0.039389 | 58 |
| GO:0015684 | ferrous iron transport | BP | 0.039389 | 58 |
| GO:0016903 | oxidoreductase activity, acting on the aldehyde or oxo group of donors | MF | 0.039389 | 30 |
| GO:1901564 | organonitrogen compound metabolic process | BP | 0.03953 | 544 |
| GO:0001664 | G-protein coupled receptor binding | MF | 0.041769 | 31 |
| GO:0048519 | negative regulation of biological process | BP | 0.041769 | 119 |
| GO:0046907 | intracellular transport | BP | 0.044143 | 365 |
| GO:0048523 | negative regulation of cellular process | BP | 0.044143 | 94 |
| GO:0032787 | monocarboxylic acid metabolic process | BP | 0.045431 | 65 |
| GO:0070838 | divalent metal ion transport | BP | 0.047565 | 73 |
| GO:0005488 | binding | MF | 0.047638 | 4785 |
| GO:0008104 | protein localization | BP | 0.048728 | 399 |

**GO enrichment of predicted targets (TT *vs.* CT group)**

| GO_accession | Description | Type | *p*-value | Gene numbers |
| --- | --- | --- | --- | --- |
| GO:0005737 | cytoplasm | CC | 0.014135 | 764 |
| GO:0071702 | organic substance transport | BP | 0.02133 | 391 |
| GO:0048523 | negative regulation of cellular process | BP | 0.022239 | 82 |
| GO:0044237 | cellular metabolic process | BP | 0.03521 | 2141 |
| GO:0051179 | localization | BP | 0.03521 | 1001 |
| GO:0048519 | negative regulation of biological process | BP | 0.03521 | 100 |
| GO:0006810 | transport | BP | 0.03521 | 961 |
| GO:0051234 | establishment of localization | BP | 0.03521 | 961 |
| GO:0006914 | autophagy | BP | 0.042554 | 90 |
| GO:0033036 | macromolecule localization | BP | 0.046469 | 354 |

MF: molecular function; BP: biological process; CC: cellular component.

*p*-value was adjusted using the Benjamini method.
